# Fluctuating Behavior and Ligand-Dependent Reactivity of Isocyanates with Bis(pentafulvene)titanium Complexes

**DOI:** 10.1021/acs.inorgchem.5c00947

**Published:** 2025-06-20

**Authors:** Marcel Eilers, Tobias Bötel, Kevin Schwitalla, Marc Schmidtmann, Rüdiger Beckhaus

**Affiliations:** † Institut für Chemie, 11233 Carl von Ossietzky Universität Oldenburg, D-26111 Oldenburg, Federal Republic of Germany; ‡ Institut für Anorganische Chemie, Technische Universität Graz, A-8010 Graz, Republic of Austria

## Abstract

Reactions of bis­(π-η^5^:σ-η^1^-pentafulvene)titanium complexes with isocyanates result in selective insertions of either one or two equivalents of RN=C=O into the two frustrated Ti–C_exo_ bonds under mild conditions. Depending on the pentafulvene ligand, K^1^ *O*- and K^1^ *N*-amidato titanium complexes are obtained and comprehensively characterized by single crystal X-ray diffraction and NMR and IR spectroscopy. In case of single insertion products, the reactivity of the second pentafulvene ligand is investigated with respect to E–H cleavage reactions (alcohols, amines) and insertion reactions of multiple-bond-containing substrates (ketones, nitriles). Transformations of the obtained K^1^ *N*- to the respective K^1^ *O*-amidato complexes are observed and investigated by DFT studies and variable temperature NMR experiments.

## Graphical abstract

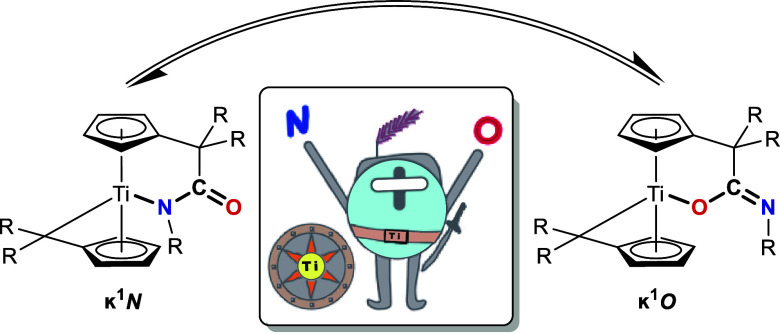

## Introduction

In addition to carbon dioxide, ketenes and carbodiimides, isocyanates represent a well-known class of heterocumulenes. The isocyanate group contains two double bonds, C=N and C=O, leading to a strong polarization due to the high electronegativities of the oxygen and nitrogen atoms, which result in different resonance structures and, consequently, high reactivity toward various substrates (Figure 1, top).^1^ One of the most significant industrial applications of isocyanates is the production of polyurethanes.^2,3^ Furthermore, isocyanates demonstrate a high degree of reactivity toward a variety of substrates in numerous reactions and are employed in the synthesis of organic compounds, for instance in the synthesis of carbodiimides.^4,5^ In transition metal chemistry, isocyanates exhibit a variety of binding modes, which have been the subject of extensive research over a considerable time period.^6,7^ The possibility of different coordination modes for N,O ligands is well-documented, with the corresponding ligand capable of binding to monometallic or bimetallic centers, thereby yielding bridging, chelating, monodentate, or allylic coordination modes.^8,9^

**Figure 1:**
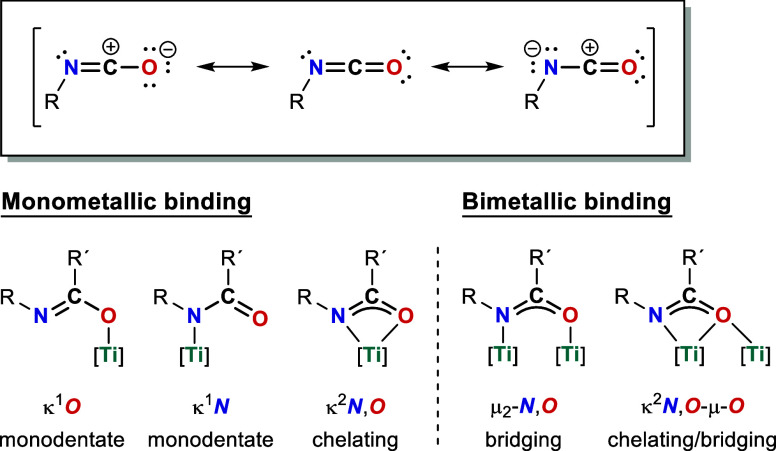
Resonance structures for isocyanates (top) and exemplified binding motives of N,O-ligands in titanium complexes (bottom).

As illustrated in Figure 1, a series of known binding modes are evident in the context of titanium complexes. Initial observations revealed K^1^ *O*- or K^1^ *N*-coordination modes, depending on the oxidation state and the steric demand of the ligands within the coordination sphere of the metal center.^10–17^ Less prevalent are bimetallic coordination modes featuring bridging ligands.^18^ Most common is the K^2^ *N*,*O* coordination mode obtained from insertion reactions of isocyanates into Ti–E bonds (E = C, N, O)^19,20^ or from deprotonation reactions and subsequent coordination to the metal center.^21^ These monoanionic N,O-chelating ligand titanium complexes have been found to be active in hydrofunctionalization and polymerization reactions.^21^ There are also examples for living polymerization reactions,^22,23^ chiral polymerization reactions,^24^ and catalytic applications for the synthesis of urea.^25^ In our group, first studies on the insertion reaction of isocyanates into Ti–C and Nb–C bonds have been published, which resulted in K^1^ *N*-amidato titanium^15^ and K^1^ *O*-amidato niobium^26^ complexes. Subsequent investigations on the reactions of M–C bonds with (hetero)­cumulenes demonstrated that bis­(π–η^5^:σ–η^1^-pentafulvene)­titanium complexes react with propa-1,2-diene to form chained η^3^-allyl complexes^27^ and with carbodiimides to K^1^ *N*-amidinato complexes.^28^ Given our previous work on pentafulvene complexes of group 4 and 5 metals and their exceptional behavior in E–H activations and multiple bond insertion reactions under ambient conditions,^26,29,30^ we herein report on the reaction of isocyanates with the respective bis­(π–η^5^:σ–η^1^-pentafulvene)­titanium complexes **1** and **2**.

## Results and Discussion

### Syntheses and Characterization of Insertion Pro-ducts

The reaction of **1** with equimolar amounts of isocyanate in *n*-hexane at room temperature resulted in an immediate color change from blue to green, and the selective formation of single products was verified by ^1^H NMR spectroscopy (Scheme 1). The reaction mixtures were stirred at room temperature for 16 h to achieve complete conversion and precipitation of the products.

**Scheme 1:**
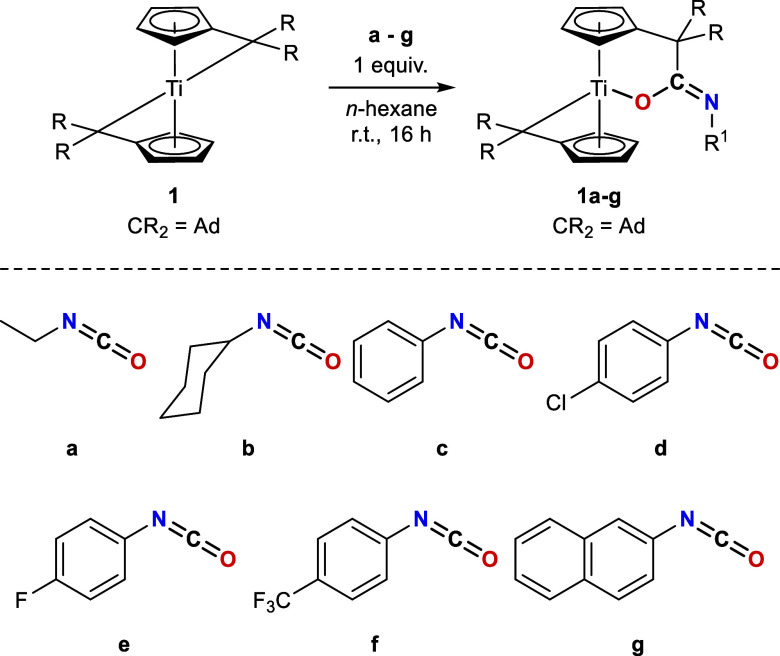
Reaction of Bis­(π–η^5^:σ–η^1^-Pentafulvene)­titanium Complex **1** with One Equivalent of Isocyanates **a**–**g** Results in the Formation of K^1^ *O*-Amidato Complexes **1a**–**g**

Independent of stoichiometry, only one of the two reactive pentafulvene ligands reacts with the isocyanates. Accordingly, the use of substoichiometric amounts of isocyanate will leave unreacted **1**, and the use of an excess will not lead to further reactions. The resulting green solids were isolated in moderate to good yields (49–93%), are air and moisture sensitive, insoluble in *n*-hexane, soluble in THF and toluene, and decompose upon heating above 171 °C. Complexes **1a**–**g** are comprehensively characterized by NMR-experiments. In the ^1^H NMR spectra, the adamantyl moieties give signals around ^1^H = 0.9–2.7 ppm with two additional multiplets that are significantly shifted to lower fields (e.g., for **1a**: ^1^H = 2.89–2.93 and 4.42–4.48 ppm). This is attributed to hydrogen interaction with the nitrogen atoms.^28^ One multiplet is found for each of the eight different protons of the chemically nonidentical pentafulvene ligands (^1^H = 3.81–7.14 ppm). Successful insertion is also indicated by the corresponding signals of the isocyanate substituents, which, in the case of alkyl substituents, also correlate in ^1^H,^15^N HMBC experiments to the nitrogen atom (^15^N = 233 ppm (**1a**) and 243 ppm (**1b**)). No ^15^N correlations were observed for aryl-substituted isocyanates. Of high diagnostic value are the ^13^C signals of the quaternary carbon atom of the incorporated isocyanate, which are summarized in Table 1. Since there are two double bonds in isocyanates, the results of ^13^C­{^1^H} NMR spectroscopy showed characteristic chemical shifts of around ^13^C = 174–177 ppm, indicating that the former carbonyl group is incorporated into the Ti–C_exo_ bond. The obtained chemical shifts are in good agreement with literature values, confirming the intact imide function.^15^ The corresponding IR *v*(C=N) frequencies are observed at around 1600 cm^–1^. Despite the tendency of electron-withdrawing substituents dictating a trend toward lower wavenumbers, **1e** deviates from this trend.

**Table 1:** Yields, Characteristic NMR and IR Signals of **1a**–**g**

complex	yield	^13^C{^1^H} O–C=N (ppm)	IR O–C=N (cm^–1^)
**1a**	93%	175.0	1615
**1b**	74%	174.0	1632
**1c**	49%	175.6	1615
**1d**	93%	176.3	1601
**1e**	83%	175.8	1624
**1f**	59%	177.2	1595
**1g**	72%	176.2	1625

The formation of the K^1^ *O*-amidato complexes was confirmed by means of single crystal X-ray diffraction. Dark green single crystals of **1a**, **1c**–**f** were obtained from benzene-*d*_6_ or toluene solutions by slow evaporation of the solvent at ambient temperature. For instance, the solid state structure of **1a** is depicted in Figure 2, while the structures of **1c**–**f** are provided in the Supporting Information. For complex **1a**, the insertion of the isocyanate into the Ti–C_exo_ bond results in the formation of an elongated C­(sp^3^)–C­(sp^2^) single bond (C21–C31 = 1.5439(7) Å) when compared to the literature value of 1.51 Å,^31^ with the central carbon atom of the isocyanate being sp^2^ hybridized. The C31–O1 bond (1.3516(6) Å) is in the range for a shortened single bond, and the C31–N1 bond (1.2752(7) Å) in the range for a C=N double bond when compared to the sum of the covalent radii (Σ*r*_cov_(C–O) = 1.38 Å; Σ*r*_cov_(C=N) = 1.27 Å).^32^ The Ti1–O1 bond length of 1.9350(4) Å is in the range for a typical Ti–O single bond (Σ*r*_cov_(Ti–O) = 1.99 Å).^33,34^ The insertion also results in the elongation of the Cp–C_exo_ bond from 1.454(3)/1.451(3) Å in **1** ^35^ to 1.5229(7) Å (C16–C21) due to the change in the hybridization of the C_exo_ carbon atom (C21). The remaining pentafulvene bond Ti1–C6 also got slightly elongated from 2.310(2)/2.320(2) Å to 2.3714(5) Å in the process. The solid state structures of **1c**–**f** show no significant differences in bond lengths (Table 3). NMR experiments performed at higher temperatures revealed no change in the coordination mode (Figure S4). In conclusion, the incorporation of various alkyl- and aryl-substituted isocyanates into the Ti–C_exo_ bond of **1** lead to the regioselective formation of K^1^ *O*-amidato complexes.

**Figure 2:**
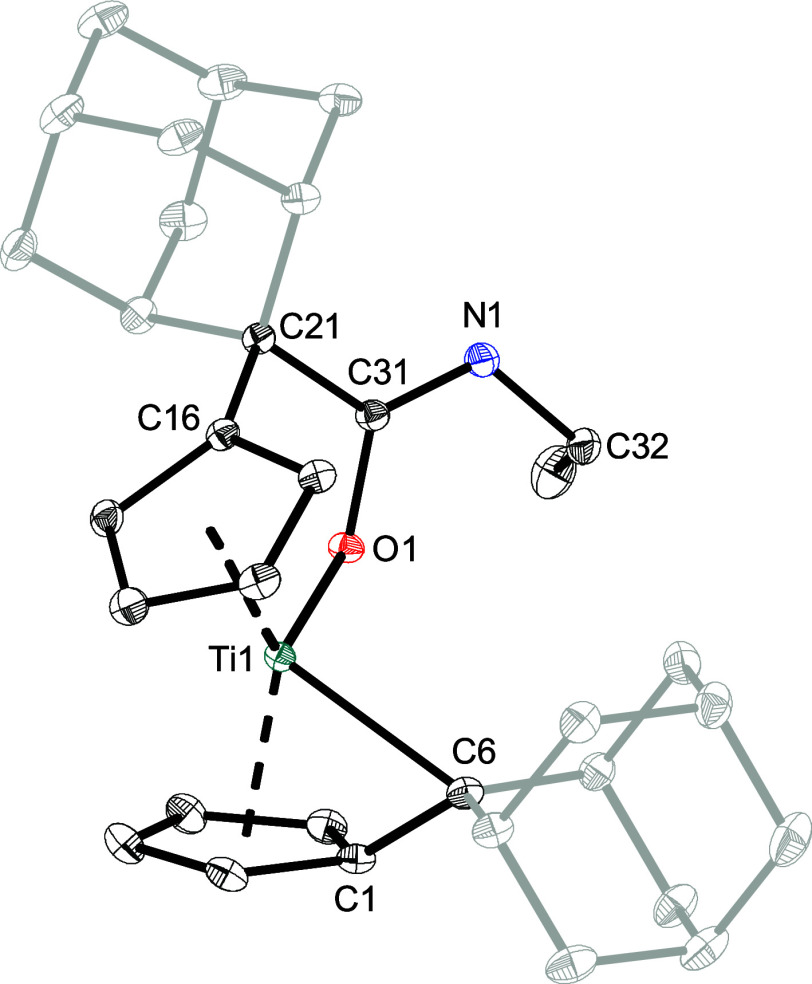
Molecular structure of **1a**. Thermal ellipsoids are drawn at the 50% probability level. Hydrogen atoms and solvent molecules are omitted for clarity.

**Table 2:** Yields, Characteristic NMR and IR Signals of **2a**–**c**

complex	yield	^13^C{^1^H} O=C–N(ppm)	IR O=C–N (cm^–1^)
**2a**	72%	193.6	1606
**2b**	74%	190.2	1602
**2c**	82%	190.2	1615

**Table 3:** Selected Bond Lengths (Å) of **1a** and **1c**–**f**

complex	Ti1–O1	Ti1–C6	C1–C6	C16–C21	C21–C31	C31–O1	C31–N1
**1a**	1.9350(4)	2.3714(5)	1.4420(7)	1.5229(7)	1.5439(7)	1.3516(6)	1.2752(7)
**1c**	1.9343(4)	2.3756(5)	1.4422(7)	1.5258(6)	1.5414(6)	1.3327(6)	1.2845(6)
**1d**	1.9245(10)	2.4029(15)	1.4387(19)	1.5238(19)	1.5411(18)	1.3338(16)	1.2814(16)
**1e**	1.9437(4)	2.3976(6)	1.4373(8)	1.5212(8)	1.5436(8)	1.3381(7)	1.2802(7)
**1f**	1.9317(8)	2.3829(11)	1.4403(15)	1.5230(14)	1.5420(14)	1.3340(13)	1.2848(13)

In contrast to the reactions of the bis­(π–η^5^:σ–η^1^-pentafulvene)­titanium complex **1** with equimolar amounts of isocyanates, only the substrates **a-c** selectively react with the *para*-toluene analogue **2** (see Scheme 2). Reactions with the substrates **d–g** resulted in mixtures of K^1^ *O*-amidato, K^1^ *N*-amidato, and double-inserted products.

**Scheme 2:**
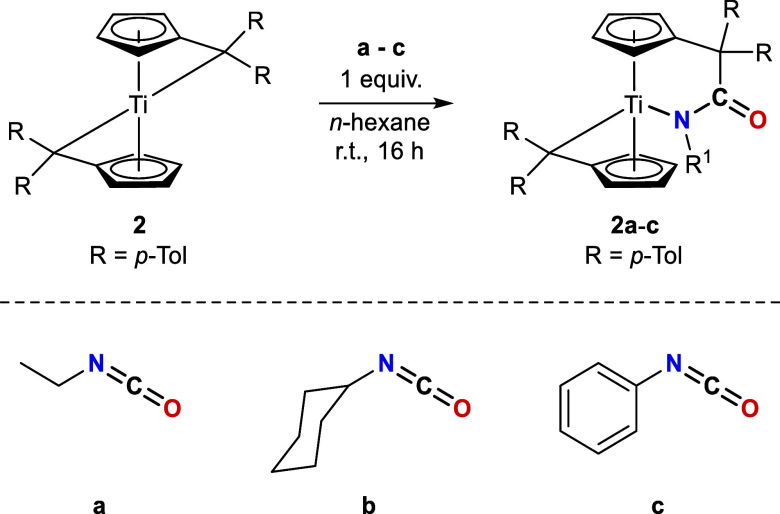
Reaction of Bis­(π–η^5^:σ–η^1^-Pentafulvene)titanium Complex **2** with One Equivalent of Isocyanates **a–c** Results in the Formation of K^1^ *N*-Amidato Complexes **2a**–**c**

In a manner analogous to **1**, the reaction mixtures were stirred at room temperature for 16 h to achieve complete conversion. After workup, the resulting solids are air and moisture sensitive, insoluble in *n*-hexane, soluble in THF and toluene, and decompose upon heating above 142 °C. **2a**–**c** are comprehensively characterized by NMR experiments. In the ^1^H NMR spectra, apart from the *para*-tolyl methyl singlets (^1^H = 2.00–2.20 ppm) and aryl signals (^1^H = 6.64–8.17 ppm), one multiplet is found for each of the eight different protons of the chemically nonidentical pentafulvene ligands (^1^H = 3.51–6.81 ppm). The successful insertion is also indicated by the corresponding signals of the isocyanate substituents, which, in the case of **2a**, also correlate in the ^1^H,^15^N HMBC experiment to the nitrogen atom (^15^N = 258 ppm). This corresponds to the observed ^15^N chemical shifts of complexes **1a** (^15^N = 243 ppm) and **1b** (^15^N = 233 ppm). However, a slight shift to lower fields is apparent, which is likely attributable to the different coordination mode. No ^15^N signals were observed for the cyclohexyl- and aryl-substituted isocyanates.

In contrast to **1a**–**g**, the ^13^C­{^1^H} NMR spectroscopy of **2a**–**c** demonstrate the presence of characteristic ^13^C chemical shifts for the quaternary carbon atom of the incorporated isocyanate group at ^13^C = 190–194 ppm (see Table 2). These chemical shifts are low field shifted and in good agreement with organic ketones, suggesting inverse regioselectivity, with the former imine group being incorporated into the Ti–C_exo_ bond, leaving the carbonyl function unreacted.^15^ The formation of K^1^ *N*-amidato complexes was confirmed by X-ray diffraction analysis. Dark green crystals of **2a**–**c** were obtained from benzene-*d*_6_ or toluene solutions by slow evaporation at ambient temperature. The solid state structure of **2a** is depicted in Figure 3, while the structures of **2b** and **2c** are provided in the Supporting Information.

**Figure 3:**
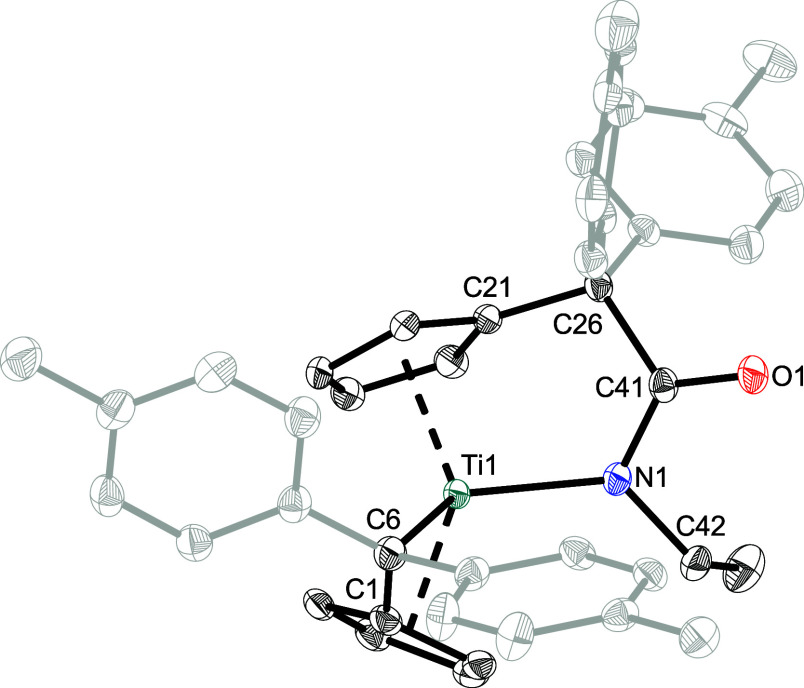
Molecular structure of **2a**. Thermal ellipsoids are drawn at the 50% probability level. Hydrogen atoms and solvent molecules are omitted for clarity.

The molecular structure of complex **2a** exhibits a Ti1–N1 bond length of 2.0741(10) Å, which is indicative of a typical single bond when compared to the sum of the covalent radii (Σ*r*_cov_(Ti–N) = 2.07 Å).^34^ However, this bond length is significantly elongated when compared to a similar complex (1.972(2) Å).^15^ The insertion of the isocyanate into the Ti–C_exo_ bond results in the formation of an elongated C­(sp^3^)–C­(sp^2^) single bond (C26–C41:1.5540(16) Å) when compared to the literature value of 1.51 Å,^31^ with the central carbon atom of the isocyanate being sp^2^ hybridized. The C41–N1 bond (1.3725(16) Å) is a shortened single bond and the C41–O1 bond (1.2315(15) Å) a shortened double bond when compared to the sum of the covalent radii (Σ*r*_cov_(C–N) = 1.46 Å; Σ*r*_cov_(C=O) = 1.24 Å).^32^ The insertion also results in the elongation of the Cp–C_exo_ bonds from 1.418(3)/1.423(3) Å^36^ to 1.5176(16) Å (C21–C26) due to the change in the hybridization of the C_exo_ carbon atom. The remaining pentafulvene bond Ti1–C6 got significantly elongated from 2.392(2)/2.408(2) Å to 2.5589(13) Å in the process, indicating a haptotropic shift of the Cp ring from η^5^:η^1^ to η^4^.^36^ The elongation of the Ti–C_exo_ bond is particularly evident in complex **2a**, as the extension of the bond length is less pronounced in complexes **2b** and **2c** (Table 5).

**Table 4:** Yields, Characteristic NMR and IR Signals of **3a**–**c**

complex	yield	^13^C{^1^H} O–C=N (ppm)	^13^C{^1^H} O=C–N (ppm)	IR C=N, C=O (cm^–1^)
**3a**	75%	174.9	190.6	1606, 1637
**3b**	50%	175.0	188.5	1602, 1630
**3c**	77%	175.5	191.7	1588, 1622

**Table 5:** Selected Bond Lengths (Å) of **2a**–**c**

complex	Ti1–N1	Ti1–C6	C1–C6	C21–C26	C26–C41	C41–N1	C41–O1
**2a**	2.0741(10)	2.5589(13)	1.4340(18)	1.5176(16)	1.5540(16)	1.3725(16)	1.2315(15)
**2b**	2.0908(8)	2.4726(10)	1.4419(13)	1.5144(12)	1.5564(12)	1.3715(11)	1.2355(11)
**2c**	2.0778(7)	2.4207(8)	1.4452(11)	1.5138(11)	1.5567(11)	1.3769(11)	1.2279(10)

Despite the variation in coordination observed between **2a**–**c** and **1a**–**g**, the IR bands observed at around 1600 cm^–1^ remain uncharacteristically shifted, hindering the reliable assignment of coordination via IR spectroscopy. In contrast to complex **1**, complex **2** also reacts with two equivalents of isocyanate, resulting in the insertion reaction of two molecules of the isocyanates into both Ti–C_exo_ bonds (Scheme 3), which was also observed in reactions with carbodiimides.^28^ This resulted in a color change of the reaction mixtures from green to yellow and the formation of single products was verified by ^1^H NMR spectroscopy. The resulting solutions were stirred for 16 h to achieve full conversion. Purification and drying under high vacuum afforded yellow (**3a,b**) or green (**3c**) solids in moderate isolated yields (72–82%). These complexes are less sensitive to air and moisture due to the functionalization of both pentafulvene ligands. They are soluble in toluene and THF and decompose on heating above 156 °C.

**Scheme 3:**
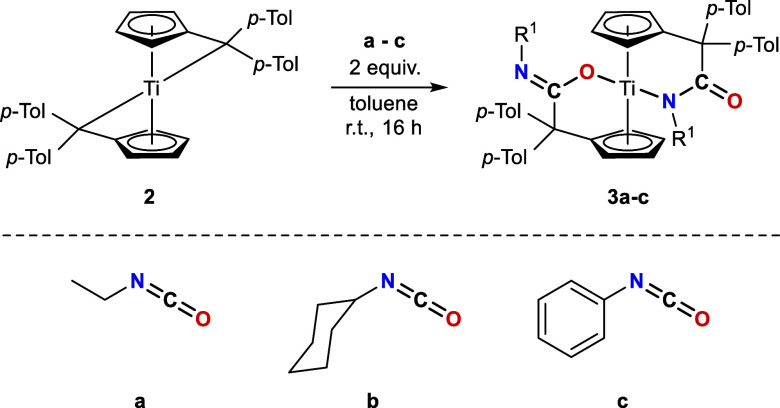
Reaction of **2** with Two Equivalents of Isocyanates **a**–**c** Results in the Formation of Complexes **3a**–**c**

In the ^1^H NMR spectra, apart from the *para*-tolyl methyl singlets (^1^H = 2.01–2.14 ppm) and aryl signals (^1^H = 6.89–7.96 ppm), one multiplet is found for each of the eight different protons of the chemically nonidentical pentafulvene ligands (^1^H = 4.82–6.55 ppm). This finding suggests an asymmetrically substituted structure, which is also indicated by the signals of the corresponding isocyanate substituents. Furthermore, the ^15^N values for the ethyl-substituted complex **3a** were determined through 2D ^1^H, ^15^N NMR experiments. The chemical shifts of the two nitrogen atoms are found at ^15^N = 244 and 248 ppm, which are almost identical. In the ^13^C­{^1^H} NMR spectra, both, the signals for the inserted carbonyl function (^13^C = 170 ppm) and the signals for the inserted imine function (^13^C = 190 ppm) of the isocyanate are observed (Table 4).

These findings indicate that the two equivalents of isocyanate have been inserted into the two fulvene bonds with different binding modes to the metal center, resulting in the formation of asymmetric structures.

This was also confirmed by X-ray diffraction analysis. Yellow-green crystals of **3a**–**c** were obtained from saturated solutions in benzene-*d*_6_ or toluene by means of slow evaporation at ambient temperatures. The solid state structure of **3a** is shown in Figure 4, while the structures of **3b** and **3c** are provided in the Supporting Information.

**Figure 4:**
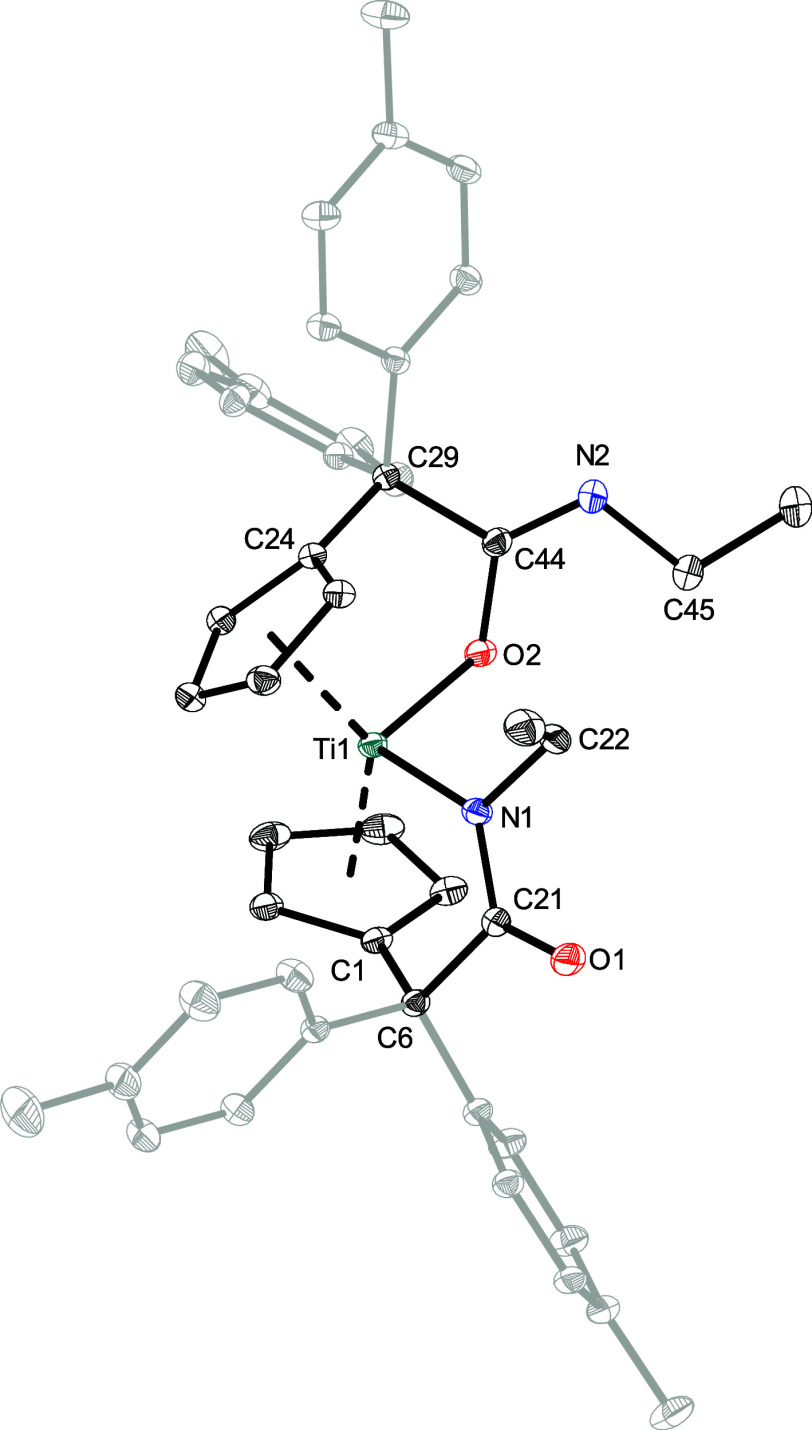
Molecular structure of **3a**. Thermal ellipsoids are drawn at the 50% probability level. Hydrogen atoms and solvent molecules are omitted for clarity.

The molecular structure of complex **3a** shows two isocyanates incorporated into the two pentafulvene ligands in an unsymmetrical manner. The newly formed Ti1–N1 (2.0750(8) Å) and Ti1–O1 (1.9171(7) Å) bonds are characterized as single bonds, and the newly formed C–C bonds (C6–C21 = 1.5552(13) Å, C29–C44 = 1.5442(13) Å) are slightly elongated single bonds. The former C=N and C=O double bonds of the isocyanate are now best described as shortened single bonds (C21–N1 = 1.3573(12) Å, C44–O2 = 1.3471(11) Å) while the respective C=N and C=O double bonds (C44–N2 = 1.2649(12) Å, C21–O1 = 1.2350(11) Å) remain intact.

Although not part of this work, it should also be feasible to synthesize mixed complexes in a stepwise fashion, as has been previously demonstrated for carbodiimides.^28^

### Reactivity of **1a** toward H-Acidic Substrates

We used the isolated mono­(π-η^5^:σ-η^1^-pentafulvene)titanium complex **1a** to investigate the remaining reactivity of the second pentafulvene function and also of the K^1^ *O*-amidato ligand in reactions with various H-acidic substrates, including amines and alcohols (Scheme 4).

**Scheme 4:**
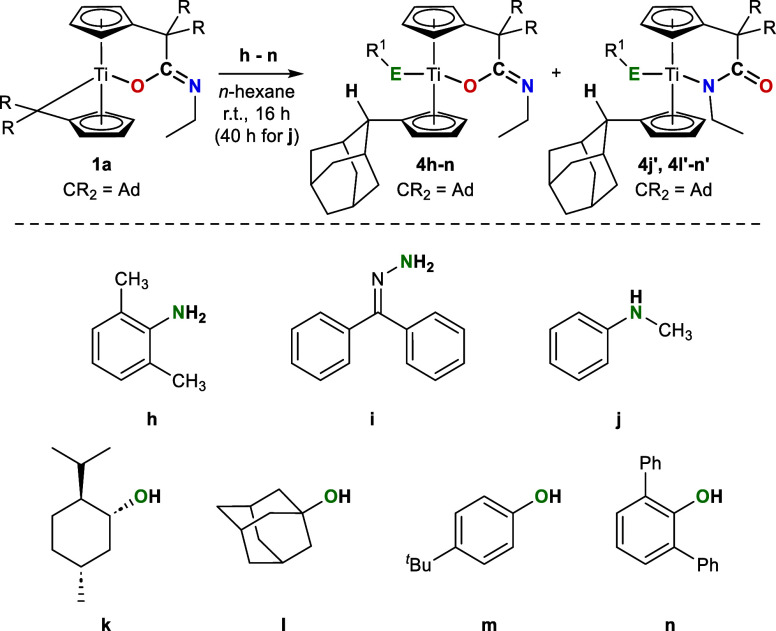
Reaction of **1a** with Amines (**h–j**) and Alcohols (**k–n**) Results in the Formation of K^1^ *O* Complexes **4** and K^1^ *N* Complexes **4′**

The reaction of **1a** with equimolar amounts of the amines **h–j** and alcohols **k–n** in *n*-hexane at room temperature resulted in a color change of the suspensions from green to red (amines) and yellow (alcohols) over a reaction time of 16 h (40 h for **j**) and the formation of colored precipitates was observed (the UV/vis spectra of all complexes are provided in the Supporting Information). The resulting complexes **4** were obtained in moderate to good yields (63–86%) and are soluble in toluene and THF. They are less sensitive to air and moisture and decompose upon heating to 163–197 °C (amines) and 177–216 °C (alcohols). ^1^H NMR spectroscopy revealed selective protonation of the C_exo_ position and coordination of the substrate to titanium in all cases. Sharp singlets for the C_exo_H protons are observed at ^1^H = 2.9–4.0 ppm, clearly distinguishable from the remaining adamantyl and Cp protons. In certain reactions, the formation of two distinct products was observed. In such cases, ^13^C­{^1^H} NMR spectra revealed that the amidato ligand can undergo a change in its coordination to the metal center, resulting in the formation of K^1^ *O*- and K^1^ *N*-amidato complexes in varying ratios depending on the substrate utilized (see Table 6).

**Table 6:** Yields, Characteristic NMR Signals and Rations of K^1^ *O* Complexes **4** and K^1^ *N* Complexes **4′**

complex	yield	^13^C{^1^H} O–C=N ppm	^13^C{^1^H}O=C–N (ppm)	ratio (NMR) 4:4′
**4h**	72%	177.1		1:0
**4i**	72%	173.9		1:0
**4j**/**4j′**	67%	176.3	187.2	9:1
**4k**	63%	176.6/177.2		1:0
**4l/4l′**	82%	175.6	188.0	1:1
**4m/4m′**	84%	175.2	190.7	1:7
**4n/4n′**	70%	175.9	191.6	1:33

The reactions of **1a** with the primary amines **h** and **i** resulted in the formation of complexes **4h** and **4i** as single products, as verified by ^1^H NMR spectroscopy. The ^13^C­{^1^H} chemical shifts indicate the selective formation of K^1^ *O*-amidato complexes, with the oxygen still coordinating to the titanium center. However, in the reaction of **1a** with the secondary amine *N*-methylaniline **j**, the formation of a byproduct in the ratio of 9:1 was observed. ^13^C NMR spectra revealed the formation of K^1^ *N*-amidato complex **4j′** as the minor product. By using analytically pure (−)-menthol **k** to protonate the C_exo_ of complex **1a**, the formation of two products is observed, but ^13^C­{^1^H} NMR spectroscopy revealed that both products are K^1^ *O* coordinated to the titanium center (^13^C­(NCO) = 176.6 and 177.2 ppm). The use of a chiral compound also made the prochiral titanium complex **1** chiral, resulting in the formation of a diastereomeric complex obtained as a racemic mixture (see Figure 5). Surprisingly, the formation of K^1^ *N*-amidato complexes was not observed in this reaction.

**Figure 5:**
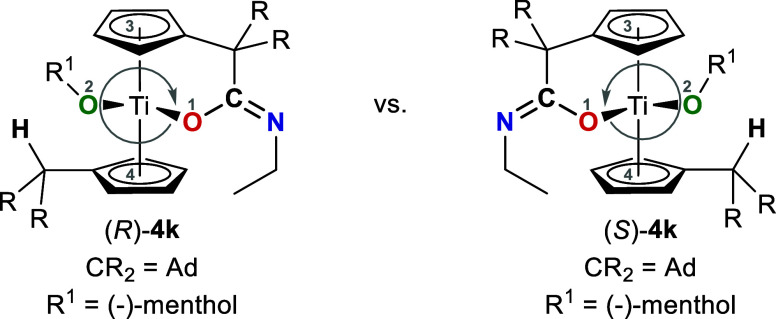
(R)- and (S)-isomers of complex **4k**.

The formation of side products was also observed in reactions of **1a** with aryl and alkyl substituted alcohols **k–n**. The reaction with 2-adamantanole resulted in a 1:1 mixture of the K^1^ *O*- and the K^1^ *N*-amidato complexes. The reaction of **1a** with the 4-*tert*-butylphenol resulted in a ratio of 1:7, favoring the formation of the K^1^ *N*-amidato complex. By reacting **1a** with 2,6-diphenylphenol as a sterically demanding substrate, the formation of the K^1^ *N*-amidato complex was observed as the main product in a ratio of 33:1 (Table 6). The formation of the complexes was confirmed by X-ray diffraction. Single crystals were obtained from saturated solutions of the products in benzene-*d*_6_ or toluene by slow evaporation of the solvents at ambient temperature. The solid state structures show either the K^1^ *O*-(**4h**, **4i**, **4k** and **4l**) or the K^1^ *N*-(**4m′** and **4n′**) binding mode (see Figure 6).

**Figure 6:**
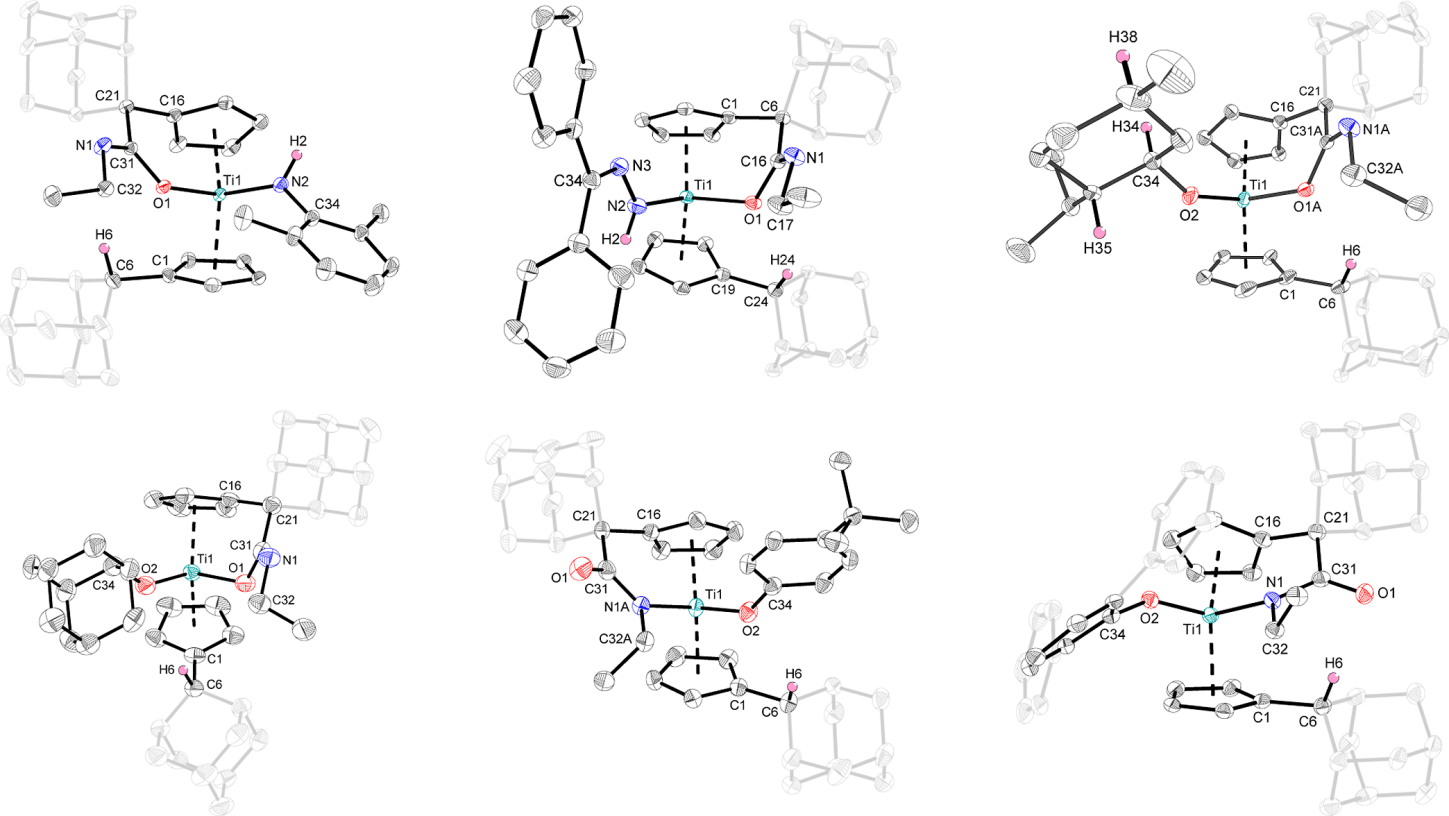
Molecular structures of the complexes **4h** (top left), **4i** (top middle), **4k** (top right), **4l** (bottom left), **4m′** (bottom middle) and **4n′** (bottom right). Thermal ellipsoids are drawn at the 50% probability level. Most hydrogen atoms, solvent molecules and disorders are omitted for clarity.

### Reactivity of **1a** toward Unsaturated Substrates

Bis­(π-η^5^:σ-η^1^-pentafulvene)­titanium complexes have also been shown to activate multiple-bond-containing substrates, such as ketones or nitriles.^28^ Therefore, we were interested in the reactions of **1a** with these compounds and whether the observed fluctuating behavior of the binding mode will also occur. The reaction of **1a** with equimolar amounts of the corresponding multiple-bond-containing substrate in *n*-hexane at ambient temperature resulted in a color change from green to yellow (**o**), orange (**p**) and pink (**q**) over a reaction time of 16 h. The formation of correspondingly colored precipitates was also observed (Scheme 5).

**Scheme 5:**
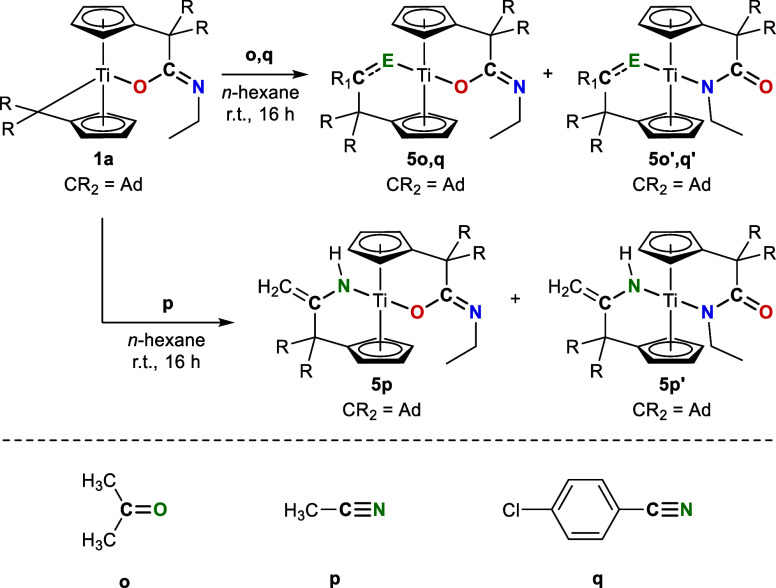
Reaction of **1a** with Unsaturated Substrates **o**, **p**, and **q** Results in the Formation of K^1^ *O* Complexes **5** and K^1^ *N* Complexes **5′**

The NMR spectra of the isolated complexes demonstrate the insertion of the substrates into the remaining pentafulvene bond of **1a**. As was previously observed for reactions with H-acidic substrates, the amidato ligand can change the coordination mode and, consequently, two products (**5o**–**q** and **5o′–q′**) are formed in each reaction. ^13^C­{^1^H} NMR spectra confirm the formation of K^1^ *O*- and K^1^ *N*-amidato complexes, in different ratios depending on the corresponding multiple-bond-containing substrate utilized (see Table 7). The reaction of **1a** with acetonitrile is accompanied by a 1,3 H-shift, resulting in the formation of the titana amido complexes **5p** and **5p′**.

**Table 7:** Yields, Characteristic NMR Signals and Ratios of K^1^ *O* Complexes **5** and K^1^ *N* Complexes **5′**

complex	yield	^13^C{^1^H} O–C=N (ppm)	^13^C{^1^H} O=C–N (ppm)	ratio (NMR)
**5o/5o′**	64%	176.2	191.2	7:1
**5p/5p′**	59%	173.9	190.6	2:1
**5q/5q′**	75%	172.3	191.3	1:1

The molecular structures of **5p′** and **5p**, as determined by single crystal X-ray diffraction, are depicted in Figure 7, and of **5o** and **5o′** in the Supporting Information.

**Figure 7:**
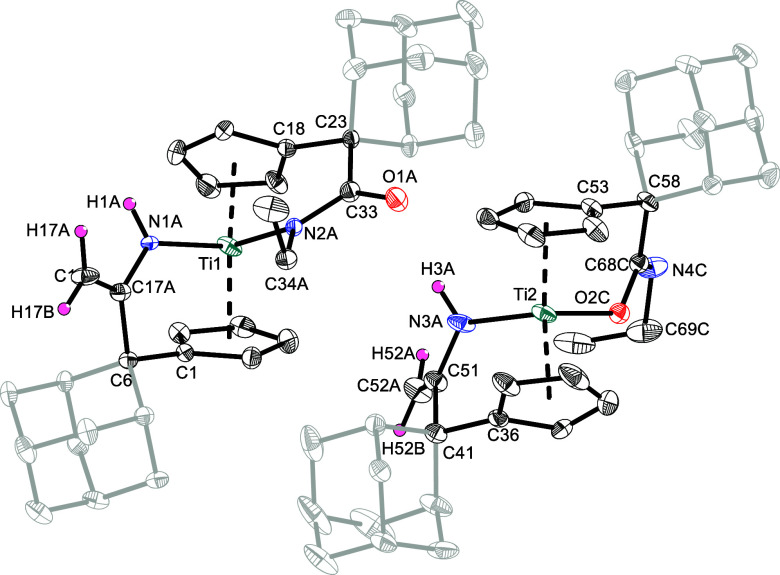
Molecular structures of **5p′** (left) and **5p** (right). Thermal ellipsoids are drawn at the 50% probability level. Hydrogen atoms (except for H1A, H3A, H17A, H17B, H52A, H52B) are omitted for clarity.

### DFT Calculations and Thermodynamic Stability

In order to rationalize the formation of isomers and the observed fluctuating behavior in the aforementioned reactions, the relative free energies (Δ*G*) of **1a**, **2a** and **4m′** were calculated based on optimized geometries calculated at the B3LYP/Def2-TZVP level of theory (see Figure 8).

**Figure 8:**
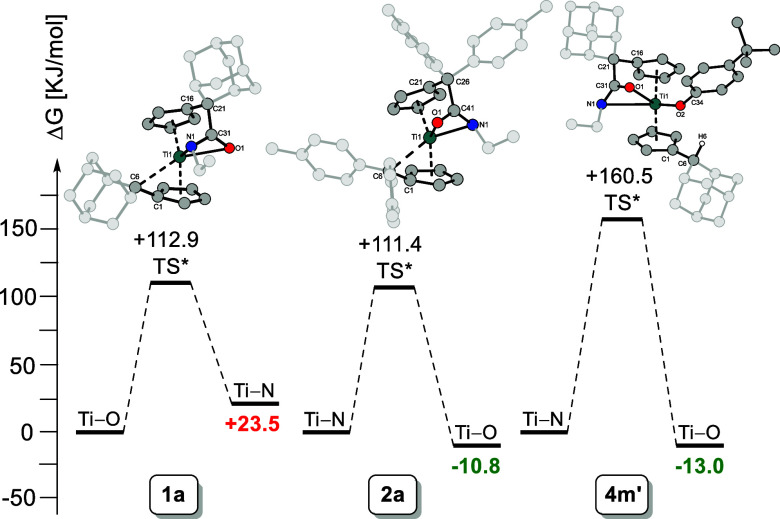
Calculated free energies of **1a**, **2a** and **4m′** and transition states (TS*) for the binding site exchange reaction. DFT calculations in toluene (B3LYP/Def2-TZVP).

The calculations demonstrate that the formation of **1a** (Ti–O) is thermodynamically favored. The Ti–N coordination (**1a′**) exhibits an increase in energy of +23.5 kJ/mol. The formation of **2a** (Ti–N) appears to be kinetically driven, with the Ti–O coordination (**2a′**) exhibiting a slight preference of −10.8 kJ/mol. The transition states for the exchange reactions in both cases are relatively high (+112.9 and +111.4 kJ/mol), therefore no change in the coordination mode is observed at ambient temperatures. For the functionalized complex **4m′**, the transformation from Ti–N to Ti–O coordination exhibits an even higher transition state (+160.5 kJ/mol), while the energy difference between the products is only minor (−13.0 kJ/mol).

Due to of the relatively high activation barriers, an attempt was made to convert the Ti–N coordinated complexes **2a**, **3a** and **4m′** to the thermodynamically favored Ti–O binding mode. This was achieved by heating corresponding toluene-*d*_8_ solutions of the complexes at elevated temperatures for designated periods of time (Scheme 6).

**Scheme 6:**
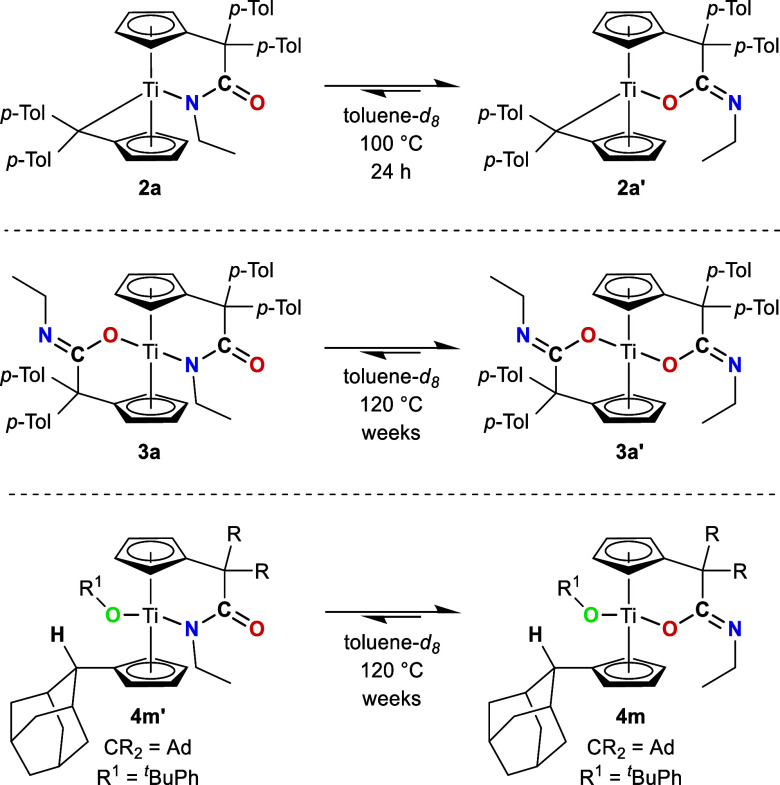
Temperature Induced Binding Site Exchange Reaction of Complexes **2a**, **3a** and **4m′**

In accordance with the calculated relative free energy profiles, the slow conversion to the respective K^1^ *O*-amidato complexes was observed; however, no full conversion was obtained, even at higher temperatures or for longer heating times. This is probably due to the high energy barriers and small energy differences of the starting materials and products (see Figure 8). The conversions were followed by ^1^H NMR spectroscopy. For instance, in the ^1^H NMR spectra of **2a**, new signals for the Ti–O coordinated form **2a′** were observed after heating the NMR sample for 2 h at 100 °C (see Figure 9). It was determined that the equilibrium between **2a** (*) and **2a′** (°) is achieved after heating for 24 h at 100 °C, resulting in a ratio of 1:2. As previously mentioned, a full conversion was not observed, even after heating the sample for additional days at 120 °C.

**Figure 9:**
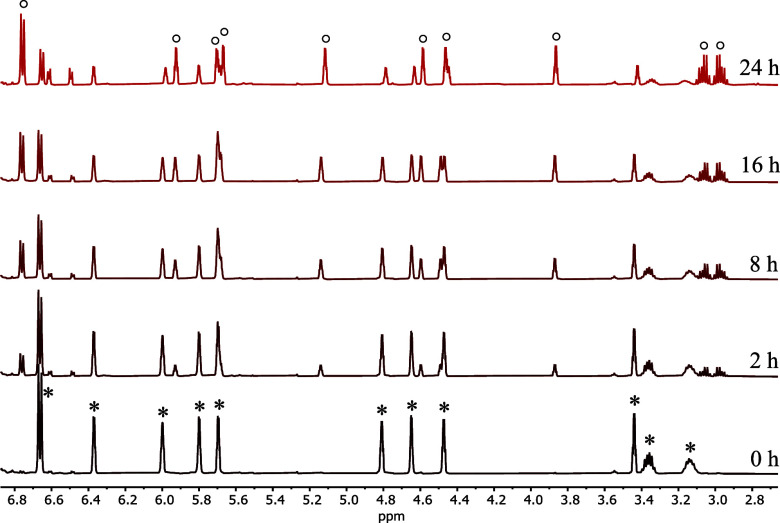
Excerpts from the ^1^H NMR spectra of **2a** (500 MHz, toluene-*d*_8_, at 305 K, **2a** = *, **2a′** = °) after heating the NMR-tube at 100 °C.

In the context of the binding site exchange reaction induced by unsaturated or H-acidic substrates, it is hypothesized that the initial step in this sequence is the coordination of the substrate to the metal center. This might result in a haptotropic shift of a Cp ring from η^5^:η^1^ to η^4^. The amidato ligand remains bound to the C_exo_ atom; however, it is capable of dissociation from the metal center, resulting in a chelating K^2^N,O transition state. Subsequently, the precoordinated substrate can react with the remaining pentafulvene ligand. For reactive and less sterically demanding substrates, the dissociation process does not appear to be essential. However, for sterically more demanding and less reactive substrates, the K^2^N,O coordination mode is more significant and enables a change of the binding mode from K^1^ *O* to K^1^ *N* (Scheme 7).

**Scheme 7:**
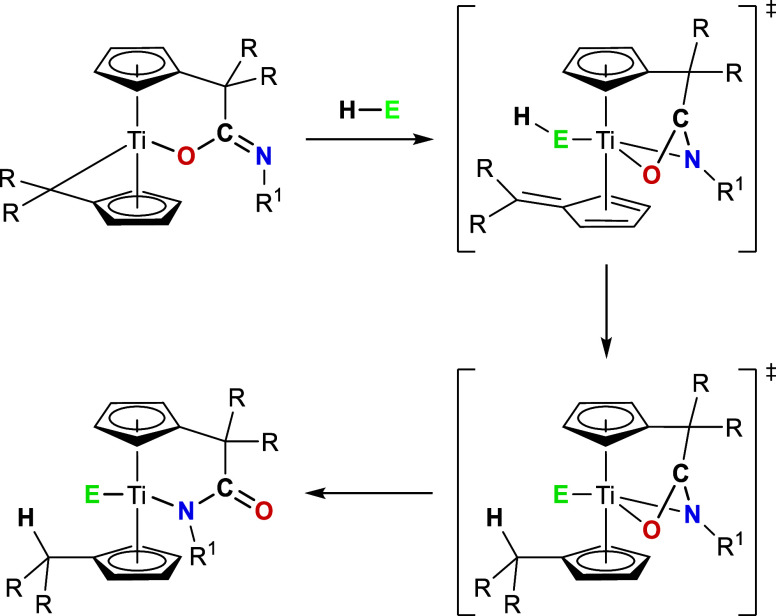
Proposed Mechanism for the Substrate Induced Binding Site Exchange Reaction, Exemplified for H-Acidic Substrates

## Summary and Conclusions

In this work, we have demonstrated the versatile chemistry of isocyanates in the coordination sphere of bis­(π–η^5^:σ–η^1^-pentafulvene)titanium complexes. Selective insertion reactions of both double bonds of the isocyanates into the polarized Ti–C_exo_ bonds were observed, which was found to be dependent on the pentafulvene ligand. In contrast to complex **1**, complex **2** reacts with two equivalents of isocyanates, resulting in mixed titanium K^1^ *O* and K^1^ *N* amidato complexes. The respective coordination modes were determined by ^13^C NMR spectroscopy and confirmed by X-ray diffraction. The additional functionalization of the second pentafulvene ligand of **1a** with H-acidic and unsaturated substrates has been shown to result in a change of coordination of the amidato ligand from K^1^ *O* to K^1^ *N* in different ratios depending on the substrates. The efficient, selective and stepwise functionalization of the two pentafulvene ligands under mild conditions with high yields demonstrates the flexibility of bis­(π–η^5^:σ–η^1^-pentafulvene)­complexes for the synthesis of functionalized complexes. Computational calculations were performed to confirm a conceivable conversion from the Ti–N to the thermodynamically favored Ti–O binding mode, involving a chelating transition state. However, these calculations also show high energy barriers for these reactions, a finding that is further supported by high temperature NMR experiments.

## Experimental Section

*Caution!* Extreme care should be taken both in the handling of the cryogen liquid nitrogen and its use in the Schlenk line trap to avoid the condensation of oxygen from air. All reactions were carried out under a dry nitrogen or argon atmosphere using standard Schlenk and glovebox techniques. Caution! Extreme care should be taken both in the handling of cryogen liquid nitrogen and its use in the Schlenk line trap to avoid the condensation of oxygen from air. Solvents were dried according to standard procedures over Na/K alloy with benzophenone as indicator and subsequently distilled and stored under a nitrogen atmosphere. NMR spectra were recorded on a Bruker AVANCE III 500 MHz spectrometer or a JEOL JNM-ECZL 500 MHz spectrometer. The IR spectra were recorded on a Bruker Tensor 27 spectrometer using an attenuated total reflection (ATR) method. Elemental analyses were carried out on a Euro EA 3000 Elemental Analyzer. Melting points were determined using a Mettler Toledo MP30. Further exact details of syntheses, crystallographic data, NMR, IR and UV/vis spectra are given in the Supporting Information.

## Supplementary Material



## Notes

Spectra and crystallographic details (PDF)
