# Supplementary material for: Fluctuating Behavior and Ligand-Dependent Reactivity of Isocyanates with Bis(pentafulvene)titanium Complexes
Source: Inorg Chem. 2025 Jun 20;64(26):12990–9. doi: 10.1021/acs.inorgchem.5c00947 (PMC12239070; doi:10.1021/acs.inorgchem.5c00947)
Supplement: Supplementary file 1 [file ic5c00947_si_001.pdf]

Supporting Information

Fluctuating Behavior and Ligand-Dependent Reactivity of Isocyanates with Bis(pentafulvene)titanium Complexes

Marcel Eilers[a], Tobias Bötel[a#], Kevin Schwitalla[a], Marc Schmidtmann[a] and Rüdiger Beckhaus[a*]

[a] *Institut für Chemie, Carl von Ossietzky Universität Oldenburg, D-26111 Oldenburg, Federal Republic of Germany*

[#] *Institut für Anorganische Chemie, Technische Universität Graz, A-8010 Graz, Republic of Austria*

E-mail: ruediger.beckhaus@uol.de

Table of Content

Experimental Section S1

NMR Spectra S29

IR Spectra S63

UV/vis Spectra S75

Crystallographic Data S87

Computational Part S107

References S112

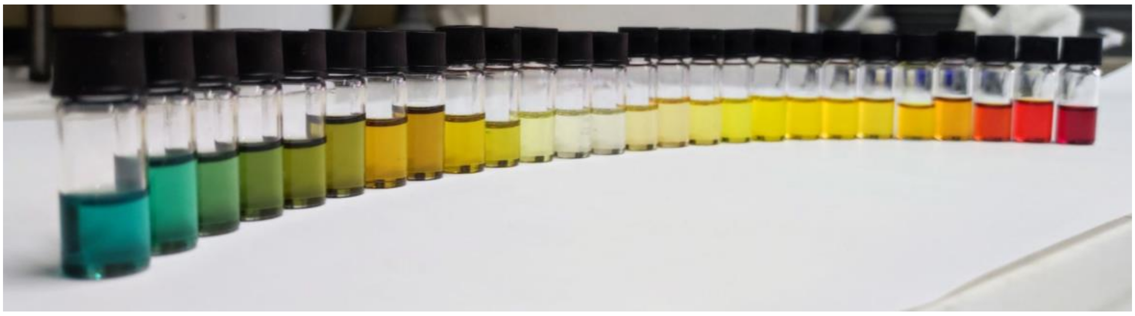

Experimental Section

General Considerations

Caution! Extreme care should be taken both in the handling of the cryogen liquid nitrogen and its use in the Schlenk line trap to avoid the condensation of oxygen from air. All reactions were carried out under a dry, oxygen-free nitrogen or argon atmosphere with strict exclusion of oxygen and moisture using standard Schlenk and glovebox (< 0.5 ppm O_2, < 0.05 ppm H_2O) techniques. Glassware was stored in an oven at 120 °C, evacuated, and purged before use. Complexes **1a** and **1b** were prepared according to published procedures.[1,2] Isocyanates were purchased from commercial suppliers and were used without further purification. Solvents were dried according to standard procedures over Na/K alloy with benzophenone as an indicator and subsequently distilled and stored under a nitrogen atmosphere. Solids were stored and weighed in a glovebox and dried under high vacuum prior to use. NMR spectra were recorded on a Bruker AVANCE III RMN 1Bay 500 MHz spectrometer or a JEOL JNM-ECZL 500 MHz spectrometer (^{1}H 500 MHz; ^{13}C 126 MHz; ^{15}N 51 MHz; ^{19}F 470 MHz). The NMR chemical shifts of ^{1}H and ^{13}C were referenced to residual protons of the solvent. Absolute values of the coupling constants (δ) are given in Hertz (Hz). Multiplicities of peaks are abbreviated as single (s), double (d), triplet (t), multiplet (m) or broad (br). Chemical shifts of ^{15}N are given from ^{1}H,^{15}N HMBC NMR experiments with nitromethane as external standard (378.9 ppm vs. NH_3). IR spectra were recorded on a Bruker Tensor 27 spectrometer using the attenuated total reflection (ATR) method. Melting points were determined using a Mettler Toledo MP30. Samples were prepared in a glovebox, prior sealed with silicone grease and then fused before measurement. High resolution mass spectra were measured on a Thermo Fisher Scientific DFS™ Magnetic Sector HRMS System spectrometer using either LIFDI or EI as the ionization method.

General Procedure (I) for the Synthesis of 1a-g and 2a-c

In a glove box, **1** (178 mg, 0.400 mmol) or **2** (226 mg, 0.400 mmol) was suspended in 5 mL of *n*-hexane. To these suspensions, one equivalent of the corresponding isocyanate (0.400 mmol, solids were dissolved in 1 mL of *n*-hexane) was added dropwise with a syringe at room temperature. The reaction mixtures immediately turned from blue to green and a greenish precipitate formed after a few minutes. The suspensions were stirred overnight (16 h) to accomplish full conversion and precipitation of the products. The mother liquors were then decanted, the residues were washed twice with 3 mL of *n*-hexane and the green powders and crystalline solids obtained were dried under high vacuum before being stored in a glovebox.

General Procedure (II) for the Synthesis of 3a-c

In a glove box, **2** (226 mg, 0.400 mmol) was dissolved in 5 mL of toluene. Two equivalents of the corresponding isocyanate (0.800 mmol, solids dissolved in 1 mL of toluene) were added dropwise with a syringe at room temperature. The reaction mixtures changed colour from green to yellow. The solutions were stirred overnight (16 h) to accomplish full conversion and then concentrated to 2 mL under reduced pressure before adding 5 mL of *n*-hexane to precipitate the products. After a further 3 h of stirring at room temperature, the mother liquors were decanted, the residues were washed twice with 3 mL of *n*-hexane and the yellow powders and crystalline solids obtained were dried under high vacuum before being stored in a glovebox.

Synthesis of 1a

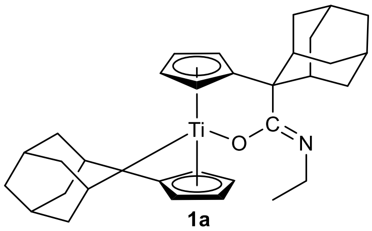

1a

Following the general procedure **(I)** but using 4.5 mmol for each substrate in 20 mL of *n*-hexane gave **1a** as a green powder (2.163 g, 4.195 mmol, 93%, mp: 171 °C (dec.)).

^{1}H NMR (500 MHz, C_6D_6, 305 K): 0.95-0.99 (m, 1 H, Ad–H), 1.23 (t, *J* = 7.2 Hz, 3 H, CH_3), 1.33-1.38 (m, 1 H, Ad–H), 1.58-1.62 (m, 1 H, Ad–H), 1.66-1.72 (m, 3 H, Ad–H), 1.74-1.86 (m, 7 H, Ad–H), 1.89-1.92 (m, 1 H, Ad–H), 1.94-1.98 (m, 2 H, Ad–H), 2.00-2.09 (m, 4 H, Ad–H), 2.14-2.17 (m, 1 H, Ad–H), 2.20-2.26 (m, 2 H, Ad–H), 2.33-2.38 (m, 1 H, Ad–H), 2.57-2.63 (m, 2 H, Ad–H), 2.89-2.98 (m, 2 H, Ad–CH, *N*–CH_2), 3.18 (dq, *J* = 12.9, 7.3 Hz, 1 H, *N*–CH_2), 3.91-3.94 (m, 1 H, Cp–H), 4.38-4.44 (m, 2 H, Ad–H, Cp–H), 4.51-4.54 (m, 1 H, Cp–H), 5.27-5.31 (m, 1 H, Cp–H), 5.72-5.76 (m, 1 H, Cp–H), 5.96-5.99 (m, 1 H, Cp–H), 6.03-6.07 (m, 1 H, Cp–H), 7.00-7.03 (m, 1 H, Cp–H) ppm.

^{13}C{^{1}H} NMR (126 MHz, C_6D_6, 305 K): 17.1 (CH_2–$\underline{C}H_3$), 28.3 (Ad–CH), 28.5 (Ad–CH), 28.9 (Ad–CH), 29.6 (Ad–CH), 33.1 (Ad–CH_2), 33.2 (Ad–CH), 33.5 (Ad–CH_2), 35.4 (Ad–CH), 35.5 (Ad–CH_2), 36.2 (Ad–CH), 36.3 (Ad–CH_2), 36.6 (Ad–CH), 37.0 (Ad–CH_2), 38.3 (Ad–CH_2), 38.4 (Ad–CH_2), 39.8 (Ad–CH_2), 41.3 ($\underline{C}H_2$–CH_3), 44.4 (Ad–CH_2), 45.4 (Ad–CH_2), 53.5 (Cp–C_{exo}), 103.4 (Cp–CH), 108.1 (Cp–CH), 108.3 (Cp–CH), 108.6 (Cp–CH), 111.2 (Cp–CH), 114.6 (Cp–CH), 116.4 (Cp–CH), 120.0 (Cp–CH), 122.6 (Cp–$\underline{C}_{exo}$–CNO), 128.3* (Cp–C_{ipso}), 140.9 (Cp–C_{ipso}), 175.0 (O–C=N) ppm. * = Overlap with solvent signal.

^{1}H,^{15}N HMBC NMR (51 MHz, 500 MHz, C_6D_6, 305 K): 232.9 ppm.

IR (ATR): ṽ = 2900 (m), 2848 (m), 1615 (m), 1571 (m), 1449 (m), 1098 (m), 1062 (m), 788 (m), 769 (s), 724 (m), 682 (m), 640 (m), 626 (m), 586 (m), 572 (m) cm^{-1}.

HRMS (LIFDI): calcd. for $C_{33}H_{41}ONTi$: m/z = 515.2662 [M^+]; found: m/z = 515.2672.

Synthesis of 1b

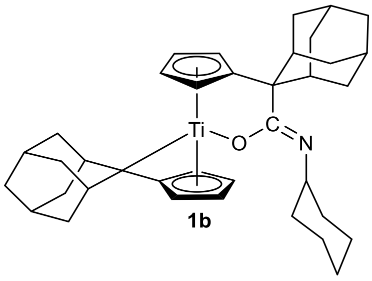

1b

Following the general procedure **(I)** gave **1b** as a green powder (167 mg, 0.296 mmol, 74%, mp: 206 °C (dec.)).

^{1}H NMR (500 MHz, C_6D_6, 305 K): 0.98-1.01 (m, 1 H, Ad–H), 1.29-1.37 (m, 3 H, Ad–H, Cy–CH_2), 1.38-1.44 (m, 2 H, Ad–H, Cy–CH_2), 1.51-1.59 (m, 2 H, Ad–H, Cy–CH_2), 1.61-1.65 (m, 1 H, Ad–H / Cy–CH_2), 1.66-1.73 (m, 4 H, Ad–H, Cy–CH_2), 1.74-1.79 (m, 4 H, Ad–H, Cy–CH_2), 1.80-1.82 (m, 3 H, Ad–H, Cy–CH_2), 1.83-1.93 (m, 4 H, Ad–H, Cy–CH_2), 1.97-2.20 (m, 2 H, Ad–H, Cy–CH_2), 2.02-2.06 (m, 2 H, Ad–H, Cy–CH_2), 2.09-2.16 (m, 2 H, Ad–H, Cy–CH_2), 2.19-2.21 (m, 1 H, Ad–H / Cy–CH_2), 2.22-2.28 (m, 2 H, Ad–H, Cy–CH_2), 2.40-2.45 (m, 1 H, Ad–H), 2.52-2.55 (m, 1 H, Ad–H), 2.58-2.63 (m, 1 H, Ad–H), 3.00-3.05 (m, 1 H, Ad–H), 3.19-3.23 (m, 1 H, Cy–CH), 3.92-3.95 (m, 1 H, Cp–H), 4.33-4.38 (m, 1 H, Ad–H), 4.42-4.46 (m, 1 H, Cp–H), 4.50-4.54 (m, 1 H, Cp–H), 5.27-5.31 (m, 1 H, Cp–H), 5.75-5.79 (m, 1 H, Cp–H), 5.99-6.02 (m, 1 H, Cp–H), 6.04-6.07 (m, 1 H, Cp–H), 7.01-7.03 (m, 1 H, Cp–H) ppm.

^{13}C{^{1}H} NMR (126 MHz, C_6D_6, 305 K): 25.3 (Cy–CH_2), 25.5 (Cy–CH_2), 26.88 (Cy–CH_2), 28.3 (Ad–CH), 28.5 (Ad–CH), 28.9 (Ad–CH), 29.6 (Ad–CH), 33.1 (Ad–CH_2), 33.3 (Ad–CH_2), 33.4 (Ad–CH), 34.2 (Cy–CH_2), 35.3 (Cy–CH_2), 35.4 (Ad–CH), 35.6 (Ad–CH_2), 36.1 (Ad–CH), 36.3 (Ad–CH_2), 36.7 (Ad–CH), 37.0 (Ad–CH_2), 38.4 (Ad–CH_2), 38.5 (Ad–CH_2), 39.9 (Ad–CH_2), 44.5 (Ad–CH_2), 45.4 (Ad–CH_2), 53.4 (Cp–C_{exo}), 54.8 (Cy–CH), 103.4 (Cp–CH), 108.1 (Cp–CH), 108.2 (Cp–CH), 108.5 (Cp–CH), 110.9 (Cp–CH), 114.7 (Cp–CH), 116.5 (Cp–CH), 119.7 (Cp–CH), 122.3 (Cp–$\underline{C}_{exo}$–CNO), 128.3* (Cp–C_{ipso}), 141.1 (Cp–C_{ipso}), 174.0 (O–C=N) ppm. * = Overlap with solvent signal.

^{1}H,^{15}N HMBC NMR (51 MHz, 500 MHz, C_6D_6, 305 K): 243.0 ppm.

IR (ATR): ṽ = 2903 (m), 2844 (m), 1632 (m), 1469 (m), 1446 (m), 1260 (m), 1202 (m), 1166 (m), 1141 (m), 1097 (m), 1078 (m), 1054 (m), 1037 (m), 812 (s), 776 (m), 761 (m), 692 (m), 583 (m), 530 (s) cm^{-1}.

HRMS (LIFDI): calcd. for $C_{37}H_{47}ONTi$: m/z = 569.3132 [M^+]; found: m/z = 569.3128.

Synthesis of 1c

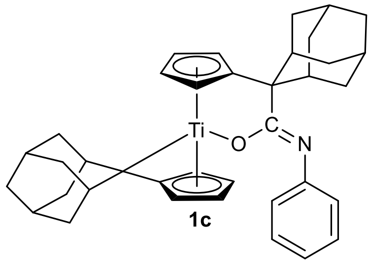

1c

Following the general procedure **(I)** gave **1c** as a green powder (110 mg, 0.195 mmol, 49%, mp: 196 °C (dec.)).

^{1}H NMR (500 MHz, C_6D_6, 305 K): 0.86-0.88 (m, 1 H, Ad–H), 1.14-1.19 (m, 1 H, Ad–H), 1.27-1.31 (m, 1 H, Ad–H), 1.41-1.46 (m, 1 H, Ad–H), 1.55-1.59 (m, 1 H, Ad–H), 1.70-1.76 (m, 6 H, Ad–H), 1.81-1.85 (m, 4 H, Ad–H), 1.91-1.99 (m, 4 H, Ad–H), 2.02-2.07 (m, 2 H, Ad–H), 2.13-2.18 (m, 2ii H, Ad–H), 2.25-2.30 (m, 1 H, Ad–H), 2.59-2.64 (m, 1 H, Ad–H), 2.65-2.69 (m, 1 H, Ad–H), 2.90-2.95 (m, 1 H, Ad–H), 3.85-3.87 (m, 1 H, Cp–H), 4.23-4.27 (m, 1 H, Ad–H), 4.37-4.39 (m, 1 H, Cp–H), 4.51-4.53 (m, 1 H, Cp–H), 5.23-5.25 (m, 1 H, Cp–H), 5.65-5.67 (m, 1 H, Cp–H), 6.01-6.02 (m, 1 H, Cp–H), 6.03-6.04 (m, 1 H, Cp–H), 6.88-6.91 (m, 1 H, Ph−H), 7.02-7.06 (m, 2 H, Ph−H), 7.11-7.14 (m, 2 H, Ph−H), 7.17* (m, 1 H, Cp–H) ppm. * = Overlap with solvent signal.

^{13}C{^{1}H} NMR (126 MHz, C_6D_6, 305 K): 28.1 (Ad–CH), 28.4 (Ad–CH), 28.8 (Ad–CH), 29.6 (Ad–CH), 33.2 (Ad–CH_2), 33.6 (Ad–CH), 33.7 (Ad–CH_2), 35.4 (Ad–CH, Ad–CH_2), 35.6 (Ad–CH), 35.8 (Ad–CH), 36.2 (Ad–CH_2), 36.9 (Ad–CH_2), 38.4 (Ad–CH_2), 38.8 (Ad–CH_2), 39.6 (Ad–CH_2), 44.4 (Ad–CH_2), 45.8 (Ad–CH_2), 54.0 (Cp–C_{exo}), 104.2 (Cp–CH), 108.4 (2 x Cp–CH), 108.6 (Cp–CH), 112.1 (Cp–CH), 114.6 (Cp–CH), 117.1 (Cp–CH), 120.5 (Cp–CH), 122.2 (Ph−CH), 124.0 (Ph−CH), 124.5 (Cp–C_{exo}–CNO), 127.9* (Ph−CH), 128.7 (Cp–C_{ipso}), 140.1 (Cp–C_{ipso}), 149.4 (Ph−C_q), 175.6 (O–C=N) ppm. * = Overlap with solvent signal.

^{1}H,^{15}N HMBC NMR (51 MHz, 500 MHz, C_6D_6, 305 K): Not detected.

IR (ATR): ṽ = 2901 (s), 2849 (m), 2360 (m), 2342 (m), 1615 (m), 1589 (m), 1499 (m), 1486 (m), 1448 (m), 1233 (m), 1180 (m), 1098 (m), 1062 (m), 852 (m), 813 (m), 813 (m), 773 (m), 750 (s), 722 (m), 691 (s), 642 (m), 580 (w) cm^{-1}.

HRMS (LIFDI): calcd. for $C_{37}H_{41}ONTi$: m/z = 563.2662 [M^+]; found: m/z = 563.2664.

Synthesis of 1d

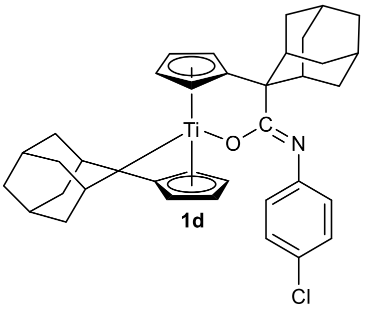

1d

Following the general procedure **(I)** gave **1d** as a green powder (223 mg, 0.373 mmol, 93%, mp: 222 °C (dec.)).

^{1}H NMR (500 MHz, C_6D_6, 305 K): 0.81-0.84 (m, 1 H, Ad–H), 1.02-1.07 (m, 1 H, Ad–H), 1.32-1.36 (m, 1 H, Ad–H), 1.53-1.57 (m, 1 H, Ad–H), 1.64-1.68 (m, 2 H, Ad–H), 1.70-1.75 (m, 6 H, Ad–H), 1.80-1.85 (m, 3 H, Ad–H), 1.88-1.93 (m, 3 H, Ad–H), 1.94-1.98 (m, 1 H, Ad–H), 1.99-2.02 (m, 2 H, Ad–H), 2.10-2.16 (m, 2 H, Ad–H), 2.22-2.26 (m, 1 H, Ad–H), 2.56-2.63 (m, 2 H, Ad–H), 2.75-2.80 (m, 1 H, Ad–H), 3.82-3.84 (m, 1 H, Cp–H), 4.12-4.17 (m, 1 H, Ad–H), 4.35-4.39 (m, 1 H, Cp–H), 4.51-4.52 (m, 1 H, Cp–H), 5.22-5.24 (m, 1 H, Cp–H), 5.61-5.62 (m, 1 H, Cp–H), 5.98-6.00 (m, 1 H, Cp–H), 6.01-6.02 (m, 1 H, Cp–H), 6.80-6.83 (m, 2 H, Ph–H), 7.07-7.10 (m, 2 H, Ph–H), 7.10-7.12 (m, 1 H, Cp–H) ppm.

^{13}C{^{1}H} NMR (126 MHz, C_6D_6, 305 K): 28.0 (Ad–CH), 28.4 (Ad–CH), 28.7 (Ad–CH), 29.5 (Ad–CH), 33.2 (Ad–CH_2), 33.5 (Ad–CH), 33.6 (Ad–CH_2), 35.3 (Ad–CH), 35.4 (Ad–CH), 35.7 (Ad–CH), 35.8 (Ad–CH), 36.1 (Ad–CH_2), 36.9 (Ad–CH_2), 38.2 (Ad–CH_2), 38.8 (Ad–CH_2), 39.5 (Ad–CH_2), 44.3 (Ad–CH_2), 45.8 (Ad–CH_2), 54.0 (Cp–C_{exo}), 104.3 (Cp–CH), 108.5 (Cp–CH), 108.6 (Cp–CH), 108.9 (Cp–CH), 112.4 (Cp–CH), 114.1 (Cp–CH), 117.1 (Cp–CH), 120.4 (Cp–CH), 124.9 (Cp–C_{exo}–CNO), 125.5 (Ph−CH), 127.3 (Ph−C_q), 127.9* (Ph−CH), 128.8 (Cp–C_{ipso}), 140.0 (Cp–C_{ipso}), 147.9 (Ph−C_q), 176.3 (O–C=N) ppm. * = Overlap with solvent signal.

^{1}H,^{15}N HMBC NMR (51 MHz, 500 MHz, C_6D_6, 305 K): Not detected.

IR (ATR): ṽ = 2899 (m), 2848 (m), 1601 (m), 1580 (m), 1485 (m), 1469 (m), 1448 (m), 1249 (m), 1230 (m), 1201 (m), 1184 (m), 1095 (m), 1062 (m), 1044 (m), 1010 (m), 992 (m), 809 (s), 786 (m), 774 (m), 760 (m), 690 (m), 627 (m), 544 (m), 510 (m) cm^{-1}.

HRMS (LIFDI): calcd. for $C_{37}H_{40}ClONTi$: m/z = 597.2272 [M^+]; found: m/z = 597.2267.

Synthesis of 1e

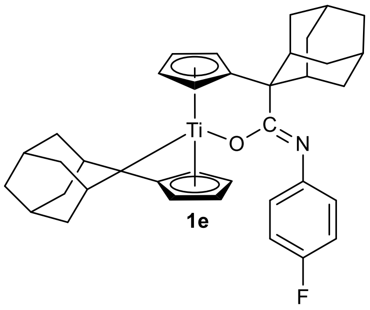

1e

Following the general procedure **(I)** gave **1e** as a green powder (194 mg, 0.333 mmol, 83%, mp: 180 °C (dec.)).

^{1}H NMR (500 MHz, C_6D_6, 305 K): 0.82-0.86 (m, 1 H, Ad–H), 1.05-1.10 (m, 1 H, Ad–H), 1.25-1.27 (m, 1 H, Ad–H), 1.32-1.37 (m, 1 H, Ad–H), 1.54-1.58 (m, 1 H, Ad–H), 1.66-1.69 (m, 2 H, Ad–H), 1.71-1.76 (m, 4 H, Ad–H), 1.79-1.84 (m, 4 H, Ad–H), 1.89-1.93 (m, 3 H, Ad–H), 1.95-1.98 (m, 1 H, Ad–H), 1.99-2.03 (m, 2 H, Ad–H), 2.11-2.17 (m, 2 H, Ad–H), 2.23-2.27 (m, 1 H, Ad–H), 2.57-2.66 (m, 2 H, Ad–H), 2.77-2.83 (m, 1 H, Ad–H), 3.82-3.84 (m, 1 H, Cp–H), 4.16-4.22 (m, 1 H, Ad–H), 4.35-4.39 (m, 1 H, Cp–H), 4.51-4.53 (m, 1 H, Cp–H), 5.22-5.25 (m, 1 H, Cp–H), 5.63-5.66 (m, 1 H, Cp–H), 6.00-6.04 (m, 2 H, 2 x Cp–H), 6.01-6.02 (m, 1 H, Cp–H), 6.76-6.82 (m, 2 H, Ph–H), 6.85-6.91 (Ph–H), 7.10-7.13 (m, 1 H, Cp–H) ppm.

^{13}C{^{1}H} NMR (126 MHz, C_6D_6, 305 K): 28.1 (Ad–CH), 28.4 (Ad–CH), 28.8 (Ad–CH), 29.5 (Ad–CH), 33.3 (Ad–CH_2), 33.5 (Ad–CH), 33.7 (Ad–CH_2), 35.3 (Ad–CH), 35.4 (Ad–CH), 35.7 (Ad–CH), 35.8 (Ad–CH), 36.1 (Ad–CH_2), 36.8 (Ad–CH_2), 38.3 (Ad–CH_2), 38.7 (Ad–CH_2), 39.5 (Ad–CH_2), 44.3 (Ad–CH_2), 45.8 (Ad–CH_2), 54.0 (Cp–C_{exo}), 104.3 (Cp–CH), 108.4 (Cp–CH), 108.6 (Cp–CH), 108.8 (Cp–CH), 112.3 (Cp–CH), 114.3 (d, J = 21.7 Hz, Ph–CH), 114.7 (Cp–CH), 117.0 (Cp–CH), 120.3 (Cp–CH), 124.7 (Cp–C_{exo}–CNO), 125.3 (d, J = 7.4 Hz, Ph−CH), 128.7 (Cp–C_{ipso}), 140.0 (Cp–C_{ipso}), 145.3 (d, J = 2.8 Hz, Ph−C_q), 159.1 (d, J = 239.7 Hz, Ph−CF), 175.8 (O–C=N) ppm.

^{1}H,^{15}N HMBC NMR (51 MHz, 500 MHz, C_6D_6, 305 K): Not detected.

^{19}F{^{1}H} NMR (470 MHz, C_6D_6, 305 K): -122.08 ppm (Ph–F).

IR (ATR): ṽ = 2906 (m), 2848 (m), 1624 (m), 1496 (m), 1468 (m), 1448 (m), 1226 (m), 1207 (m), 1195 (m), 1174 (m), 1095 (m), 1059 (m), 1040 (m), 989 (m), 831 (s), 816 (m), 796 (m), 775 (m), 580 (w), 532 (m), 51f6 (m) cm^{-1}.

HRMS (LIFDI): calcd. for $C_{37}H_{40}ONFTi$: m/z = 581.2568 [M^+]; found: m/z = 581.2577.

Synthesis of 1f

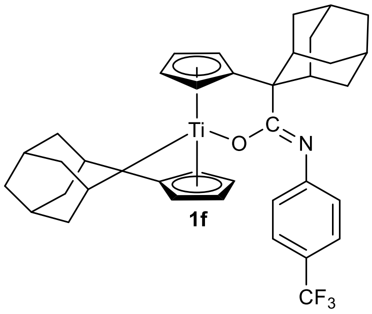

1f

Following the general procedure **(I)** gave **1f** as a green powder (148 mg, 0.234 mmol, 59%, mp: 196 °C (dec.)).

^{1}H NMR (500 MHz, C_6D_6, 305 K): 0.78-0.81 (m, 1 H, Ad–H), 0.97-1.02 (m, 1 H, Ad–H), 1.05-1.07 (m, 1 H, Ad–H), 1.27-1.32 (m, 1 H, Ad–H), 1.51-1.56 (m, 1 H, Ad–H), 1.60-1.64 (m, 1 H, Ad–H), 1.68-1.73 (m, 4 H, Ad–H), 1.74-1.84 (m, 5 H, Ad–H), 1.86-1.89 (m, 1 H, Ad–H), 1.90-1.98 (m, 4 H, Ad–H), 2.00-2.03 (m, 1 H, Ad–H), 2.07-2.12 (m, 2 H, Ad–H), 2.20-2.25 (m, 1 H, Ad–H), 2.57-2.63 (m, 2 H, Ad–H), 2.71-2.76 (m, 1 H, Ad–H), 3.80-3.83 (m, 1 H, Cp–H), 4.11-4.16 (m, 1 H, Ad–H), 4.35-4.39 (m, 1 H, Cp–H), 4.51-4.53 (m, 1 H, Cp–H), 5.22-5.25 (m, 1 H, Cp–H), 5.52-5.55 (m, 1 H, Cp–H), 5.97-6.00 (m, 1 H, Cp–H), 6.01-6.03 (m, 1 H, Cp–H), 6.86-6.90 (m, 2 H, Ph–H), 7.08-7.11 (m, 1 H, Cp–H) 7.33-7.37 (Ph–H) ppm.

^{13}C{^{1}H} NMR (126 MHz, C_6D_6, 305 K): 28.0 (Ad–CH), 28.3 (Ad–CH), 28.7 (Ad–CH), 29.4 (Ad–CH), 33.2 (Ad–CH_2), 33.4 (Ad–CH), 33.7 (Ad–CH_2), 35.3 (Ad–CH), 35.4 (Ad–CH), 35.6 (Ad–CH), 35.8 (Ad–CH), 36.1 (Ad–CH_2), 36.8 (Ad–CH_2), 38.2 (Ad–CH_2), 38.9 (Ad–CH_2), 39.4 (Ad–CH_2), 44.2 (Ad–CH_2), 45.8 (Ad–CH_2), 54.0 (Cp–C_{exo}), 104.3 (Cp–CH), 108.5 (Cp–CH), 108.8 (Cp–CH), 108.9 (Cp–CH), 112.6 (Cp–CH), 114.8 (Cp–CH), 117.2 (Cp–CH), 120.5 (Cp–CH), 124.1 (2 x Ph−CH), 125.0 (q, *J* = 4.0 Hz, 2 x Ph–CH), 125.2 (Cp–C_{exo}–CNO), 125.8 (q, *J* = 270.7 Hz, CF_3), 128.9 (Cp–C_{ipso}), 140.0 (Cp–C_{ipso}), 152.8 (Ph−C_q), 177.2 (O–C=N) ppm.

^{1}H,^{15}N HMBC NMR (51 MHz, 500 MHz, C_6D_6, 305 K): Not detected.

^{19}F{^{1}H} NMR (470 MHz, C_6D_6, 305 K): -61.07 (Ph–CF_3) ppm.

IR (ATR): ṽ = 2906 (m), 2849 (w), 1621 (w), 1595 (m), 1446 (w), 1324 (s), 1256 (w), 1235 (w), 1186 (m), 1105 (m), 1064 (m), 1043 (m), 816 (s), 791 (m) cm^{-1}.

HRMS (LIFDI): calcd. for $C_{38}H_{40}ONF_3Ti$: m/z = 631.2536 [M^+]; found: m/z = 631.2523.

Synthesis of 1g

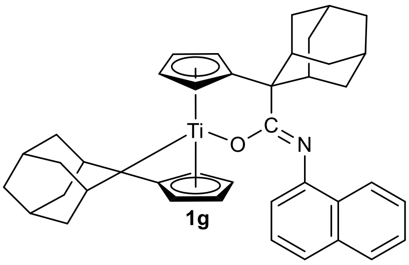

1g

Following the general procedure **(I)** gave **1g** as a green powder (177 mg, 0.290 mmol, 72%, mp: 186 °C (dec.)).

^{1}H NMR (500 MHz, C_6D_6, 305 K): 0.61-0.65 (m, 1 H, Ad–H), 0.78-0.84 (m, 2 H, Ad–H), 0.98-1.02 (m, 1 H, Ad–H), 1.27-1.32 (m, 2 H, Ad–H), 1.45-2.10 (m, 16 H, Ad–H), 2.16-2.19 (m, 1 H, Ad–H), 2.33-2.36 (m, 1 H, Ad–H), 2.60-2.65 (m, 1 H, Ad–H), 2.73-2.76 (m, 1 H, Ad–H), 3.08-3.12 (m, 1 H, Ad–H), 3.78-3.81 (m, 1 H, Cp–H), 4.26-4.30 (m, 1 H, Ad–H), 4.33-4.36 (m, 1 H, Cp–H), 4.49-4.52 (m, 1 H, Cp–H), 5.18-5.22 (m, 1 H, Cp–H), 5.40-5.43 (m, 1 H, Cp–H), 5.85-5.88 (m, 1 H, Cp–H), 6.05-6.08 (m, 1 H, Cp–H), 7.04-7.08 (m, 1 H, Naph–H), 7.13-7.16* (m, 1 H, Cp–H) 7.22-7.27 (m, 1 H, Naph–H), 7.28-7.33 (m, 1 H, Naph–H), 7.35-7.40 (m, 1 H, Naph–H), 7.41-7.44 (m, 1 H, Naph–H), 7.68-7.72 (m, 1 H, Naph–H), 8.42-8.45 (m, 1 H, Naph–H) ppm.

^{13}C{^{1}H} NMR (126 MHz, C_6D_6, 305 K): 28.0 (Ad–CH), 28.2 (Ad–CH), 28.8 (Ad–CH), 29.4 (Ad–CH), 33.4 (Ad–CH_2), 33.7 (Ad–CH), 33.8 (Ad–CH_2), 35.3 (Ad–CH), 35.6 (Ad–CH), 35.7 (Ad–CH), 35.9 (Ad–CH), 36.0 (Ad–CH_2), 36.9 (Ad–CH_2), 38.0 (Ad–CH_2), 38.2 (Ad–CH_2), 39.6 (Ad–CH_2), 44.2 (Ad–CH_2), 45.7 (Ad–CH_2), 54.5 (Cp–C_{exo}), 104.3 (Cp–CH), 108.5 (Cp–CH), 108.6 (Cp–CH), 108.6 (Cp–CH), 112.1 (Cp–CH), 114.7 (Cp–CH), 117.1 (Cp–CH), 118.2 (Naph–CH), 120.7 (Cp–CH), 122.2 (Naph–CH), 124.5 (Naph–CH), 124.7 (Cp–C_{exo}–CNO), 125.4 (Naph–CH), 125.5 (Naph–CH), 125.7 (Naph–CH), 128.0 (Naph–CH), 128.9 (Cp–C_{ipso}), 130.1 (Naph–C_q), 134.8 (Naph–C_q), 140.2 (Cp–C_{ipso}), 145.8 (Naph–C_q), 176.2 (O–C=N) ppm.

^{1}H,^{15}N HMBC NMR (51 MHz, 500 MHz, C_6D_6, 305 K): Not detected.

IR (ATR): ṽ = 2899 (m), 2848 (m), 1625 (m), 1469 (m), 1469 (m), 1446 (m), 1200 (m), 1175 (m), 1097 (m), 1060 (m), 1041 (m), 986 (m), 973 (w), 808 (s), 775 (m), 762 (m), 689 (m), 514 (m) cm^{-1}.

HRMS (LIFDI): calcd. for $C_{41}H_{43}ONTi$: m/z = 613.2819 [M^+]; found: m/z = 613.2798.

Synthesis of 2a

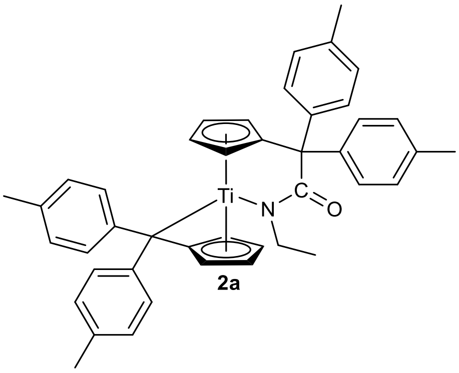

Following the general procedure **(I)** gave **2a** as a green powder (172 mg, 0.271 mmol, 68%, mp: 157 °C (dec.)).

^{1}H NMR (500 MHz, C_6D_6, 305 K): 0.74 (t, *J* = 6.9 Hz, 3 H, N–CH_2–$C\underline{H}_3$), 2.01 (s, 3 H, *p*Tol–CH_3), 2.11 (s, 6 H, *p*Tol–CH_3), 2.12 (s, 3 H, *p*Tol–CH_3), 3.17 (dq, *J* = 12.9 Hz, 7.2 Hz, 1 H, N–$C\underline{H}_2$–CH_3), 3.47 (dq, *J* = 12.8 Hz, 7.1 Hz, 1 H, N–$C\underline{H}_2$–CH_3), 3.51-3.53 (m, 1 H, Cp–H), 4.48-4.50 (m, 1 H, Cp–H), 4.67-4.69 (m, 1 H, Cp–H), 4.77-4.79 (m, 1 H, Cp–H), 5.73-5.76 (m, 1 H, Cp–H), 5.84-5.87 (m, 1 H, Cp–H), 5.99-6.02 (m, 1 H, Cp–H), 6.45-6.48 (m, 1 H, Cp–H), 6.67-6.70 (m, 2 H, *p*Tol–CH), 6.96-7.00 (m, 4 H, *p*Tol–CH), 7.05-7.10 (m, 4 H, *p*Tol–CH), 7.12-7.14 (m, 2 H, *p*Tol–CH), 7.78-7.82 (m, 2 H, *p*Tol–CH), 8.14-8.17 (m, 2 H, *p*Tol–CH) ppm.

^{13}C{^{1}H} NMR (126 MHz, C_6D_6, 305 K): 16.2 (N–CH_2–$\underline{C}H_3$), 20.85 (*p*Tol–CH_3), 20.92 (*p*Tol–CH_3), 21.01 (*p*Tol–CH_3), 21.04 (*p*Tol–CH_3), 49.4 (N–$\underline{C}H_2$–CH_3), 63.0 (Cp–C_{exo}), 108.0 (Cp–CH), 108.2 (Cp–CH), 109.7 (Cp–CH), 110.3 (Cp–CH), 113.0 (Cp–CH), 115.2 (Cp–CH), 117.9 (Cp–CH), 119.7 (Cp–CH), 120.1 (Cp–C_{exo}), 126.1 (*p*Tol–CH), 126.7 (Cp–C_{ipso}), 128.7 (*p*Tol–CH), 128.76 (*p*Tol–CH), 128.81 (*p*Tol–CH), 130.5 (*p*Tol–CH), 130.6 (*p*Tol–CH), 131.5 (*p*Tol–CH), 134.7 (Cp–C_{ipso}), 135.0 (*p*Tol–C_q), 136.1 (*p*Tol–C_q), 136.3 (*p*Tol–C_q), 136.6 (*p*Tol–C_q), 140.6 (*p*Tol–C_q), 141.5 (*p*Tol–C_q), 143.1 (2 x *p*Tol–C_q), 193.5 (N–C=O) ppm.

^{1}H,^{15}N HMBC NMR (51 MHz, 500 MHz, C_6D_6, 305 K): 257.8 (N–C=O) ppm.

IR (ATR): ṽ = 3023 (w), 2961 (w), 2919 (w), 2867 (w), 1606 (m), 1585 (m), 1532 (m), 1510 (m), 1446 (m), 1361 (m), 1085 (m), 1044 (m), 1021 (m), 823 (m), 810 (s), 787 (s), 773 (s), 764 (m), 725 (m), 675 (m), 636 (m), 586 (m), 576 (m), 554 (m), 512 (m) cm^{-1}.

HRMS (EI): calcd. for $C_{43}H_{41}ONTi$: m/z = 635.2662 [M^+]; found: m/z = 635.2664.

Synthesis of 2b

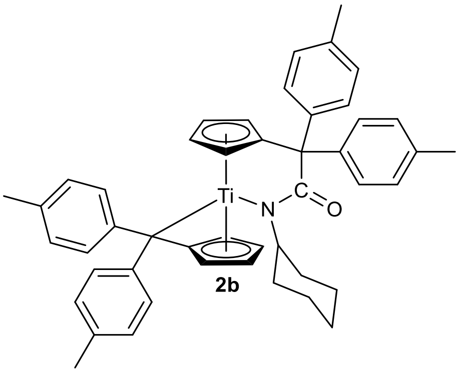

2b

Following the general procedure **(I)** gave **2b** as a green powder (171 mg, 0.248 mmol, 62%, mp: 142 °C (dec.)).

^{1}H NMR (500 MHz, C_6D_6, 305 K): 0.62-0.72 (m, 1 H, Cy–CH_2), 0.80-0.86 (m, 1 H, Cy–CH_2), 0.90-1.00 (m, 2 H, Cy–CH_2), 1.16-1.22 (m, 1 H, Cy–CH_2), 1.43-1.49 (m, 1 H, Cy–CH_2), 1.55-1.62 (m, 1 H, Cy–CH_2), 1.68-1.74 (m, 1 H, Cy–CH_2), 2.04 (s, 3 H, *p*Tol–CH_3), 2.11 (s, 3 H, *p*Tol–CH_3), 2.12 (s, 3 H, *p*Tol–CH_3), 2.14 (s, 3 H, *p*Tol–CH_3), 2.49-2.56 (m, 1 H, Cy–CH), 2.75-2.86 (m, 1 H, Cy–CH_2), 4.03-4.06 (m, 1 H, Cp–H), 4.40-4.43 (m, 1 H, Cp–H), 4.70-4.73 (m, 1 H, Cp–H), 4.74-4.77 (m, 1 H, Cp–H), 5.56-5.59 (m, 1 H, Cp–H), 5.83-5.86 (m, 1 H, Cp–H), 6.13-6.16 (m, 1 H, Cp–H), 6.59-6.62 (m, 1 H, Cp–H), 6.72-6.76 (m, 2 H, *p*Tol–CH), 6.94-6.98 (m, 2 H, *p*Tol–CH), 6.99-7.02 (m, 2 H, *p*Tol–CH), 7.02-7.05 (m, 2 H, *p*Tol–CH), 7.14-7.16 (m, 2 H, *p*Tol–CH), 7.17-7.18 (m, 2 H, *p*Tol–CH), 7.84-7.87 (m, 2 H, *p*Tol–CH), 7.98-8.02 (m, 2 H, *p*Tol–CH) ppm.

^{13}C{^{1}H} NMR (126 MHz, C_6D_6, 305 K): 20.87 (*p*Tol–CH_3), 20.93 (*p*Tol–CH_3), 21.01 (*p*Tol–CH_3), 21.04 (*p*Tol–CH_3), 26.3 (Cy–CH_2), 27.2 (Cy–CH_2), 27.8 (Cy–CH_2), 30.4 (Cy–CH_2), 31.4 (Cy–CH_2), 63.3 (Cp–C_{exo}), 68.1 (Cy–CH), 106.7 (Cp–CH), 109.4 (Cp–CH), 110.3 (Cp–CH), 110.6 (Cp–CH), 113.1 (Cp–CH), 116.2 (Cp–CH), 117.8 (Cp–CH), 119.98 (Cp–C_{exo}), 120.04 (Cp–CH), 126.2 (*p*Tol–CH), 126.5 (Cp–C_{ipso}), 128.6 (*p*Tol–CH), 128.7 (*p*Tol–CH), 128.8 (*p*Tol–CH), 129.6 (*p*Tol–CH), 130.3 (*p*Tol–CH), 130.7 (*p*Tol–CH), 131.1 (Cp–C_{ipso}), 134.3 (*p*Tol–C_q), 135.2 (*p*Tol–C_q), 135.8 (*p*Tol–C_q), 136.2 (*p*Tol–C_q), 136.7 (*p*Tol–C_q), 140.3 (*p*Tol–C_q), 141.4 (*p*Tol–C_q), 142.9 (*p*Tol–C_q), 143.7 (*p*Tol–C_q), 190.2 (N–C=O) ppm.

^{1}H,^{15}N HMBC NMR (51 MHz, 500 MHz, C_6D_6, 305 K): Not detected.

IR (ATR): $\tilde{\nu}$ = 3092 (w), 3022 (w), 2919 (w), 2848 (w), 1602 (s), 1506 (m), 1460 (m), 1452 (m), 1249 (m), 1218 (m), 1193 (m), 1057 (m), 1047 (m), 1022 (m), 950 (m), 830 (m), 810 (s), 803 (s), 792 (s), 779 (s), 764 (m), 744 (s), 732 (m), 676 (m), 572 (m), 562 (m), 554 (m) cm^{-1}.

HRMS (EI): calcd. for $C_{47}H_{47}ONTi$: m/z = 689.3132 [M^+]; found: m/z = 689.3134.

Synthesis of 2c

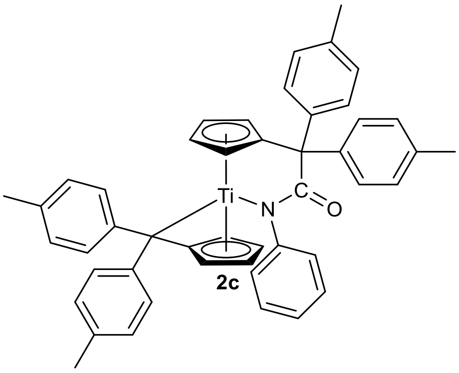

Following the general procedure **(I)** gave **2c** as a green powder (224 mg, 0.328 mmol, 82%, mp: 157 °C (dec.)).

^{1}H NMR (500 MHz, C_6D_6, 305 K): 2.00 (s, 3 H, *p*Tol–CH_3), 2.11 (s, 3 H, *p*Tol–CH_3), 2.14 (s, 3 H, *p*Tol–CH_3), 2.20 (s, 3 H, *p*Tol–CH_3), 3.51-3.53 (m, 1 H, Cp–H), 4.54-4.59 (m, 2 H, Cp–H), 4.74-4.76 (m, 1 H, Cp–H), 5.29-5.31 (m, 1 H, Cp–H), 5.41-5.44 (m, 1 H, Ph–H), 5.60-5.62 (m, 1 H, Cp–H), 6.04-6.06 (m, 1 H, Cp–H), 6.32-6.36 (m, 1 H, Ph–H), 6.64-6.67 (m, 2 H, *p*Tol–CH), 6.79-6.81 (m, 1 H, Cp–H), 6.84-6.89 (m, 1 H, Ph–CH), 6.90-6.95 (m, 2 H, Ph–CH), 7.01-7.04 (m, 2 H, *p*Tol–CH), 7.05-7.09 (m, 2 H, *p*Tol–CH), 7.10-7.13 (m, 2 H, *p*Tol–CH), 7.17-7.19 (m, 2 H, *p*Tol–CH), 7.20-7.23 (m, 2 H, *p*Tol–CH), 7.80-7.83 (m, 2 H, *p*Tol–CH), 8.20-8.23 (m, 2 H, *p*Tol–CH) ppm.

^{13}C{^{1}H} NMR (126 MHz, C_6D_6, 305 K): 20.8 (*p*Tol–CH_3), 20.9 (*p*Tol–CH_3), 21.0 (2 x *p*Tol–CH_3), 62.9 (Cp–C_{exo}), 107.7 (Cp–CH), 108.5 (Cp–CH), 109.7 (Cp–CH), 111.1 (Cp–CH), 113.5 (Cp–CH), 114.9 (Cp–CH), 119.8 (Cp–C_{exo}), 120.4 (Cp–CH), 122.5 (Cp–CH), 124.1 (Ph–CH), 126.0 (*p*Tol–CH), 126.9 (Ph–CH), 127.3 (Cp–C_{ipso}), 127.7 (Ph–CH), 128.2* (Ph–CH), 128.75 (*p*Tol–CH), 128.89 (*p*Tol–CH), 128.94 (*p*Tol–CH), 129.4 (*p*Tol–CH), 130.4 (*p*Tol–CH), 130.7 (*p*Tol–CH), 131.1 (*p*Tol–CH), 134.3 (Cp–C_{ipso}), 134.5 (*p*Tol–C_q), 135.2 (*p*Tol–C_q), 136.2 (*p*Tol–C_q), 136.6 (*p*Tol–C_q), 136.7 (*p*Tol–C_q), 141.2 (*p*Tol–C_q), 141.3 (*p*Tol–C_q), 142.7 (*p*Tol–C_q), 155.7 (Ph–C_q), 192.3 (N–C=O) ppm. * = Overlap with solvent signal.

^{1}H,^{15}N HMBC NMR (51 MHz, 500 MHz, C_6D_6, 305 K): Not detected.

IR (ATR): ṽ = 3089 (w), 3021 (w), 2954 (w), 2919 (w), 2866 (w), 1619 (s), 1588 (m), 1508 (m), 1484 (m), 1447 (m), 1259 (m), 1242 (m), 1187 (m), 1083 (m), 1070 (m), 1022 (m), 1003 (m), 807 (s), 799 (s), 778 (m), 762 (m), 751 (s), 740 (s), 698 (m), 572 (m), 568 (m), 554 (m), 503 (m) cm^{-1}.

HRMS (EI): calcd. for $C_{47}H_{41}ONTi$: m/z = 683.2662 [M^+]; found: m/z = 683.2667.

Synthesis of 3a

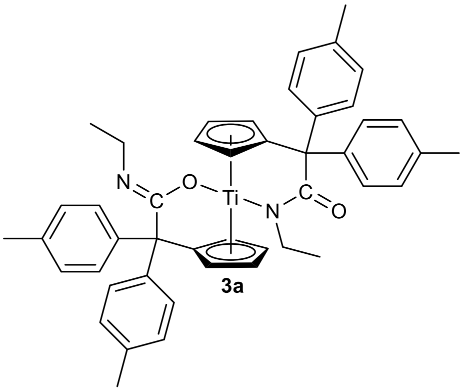

3a

Following the general procedure **(II)** gave **3a** as a yellow powder (211 mg, 0.299 mmol, 75%, mp: 186 °C (dec.)).

^{1}H NMR (500 MHz, C_6D_6, 305 K): 1.29 (t, *J* = 6.9 Hz, 3 H, N–CH$_2$–C$\underline{H}_3$), 1.35 (t, *J* = 7.3 Hz, 3 H, N–CH$_2$–C$\underline{H}_3$), 2.09 (s, 3 H, *p*Tol–CH$_3$), 2.10 (s, 3 H, *p*Tol–CH$_3$), 2.11 (s, 3 H, *p*Tol–CH$_3$), 2.13 (s, 3 H, *p*Tol–CH$_3$), 3.37 (dq, *J* = 12.8 Hz, 7.2 Hz, 1 H, N–C$\underline{H}_2$–CH$_3$), 3.51 (dq, *J* = 12.8 Hz, 7.2 Hz, 1 H, N–C$\underline{H}_2$–CH$_3$), 3.59 (dq, *J* = 12.8 Hz, 7.0 Hz, 1 H, N–C$\underline{H}_2$–CH$_3$), 3.69 (dq, *J* = 13.0 Hz, 6.8 Hz, 1 H, N–C$\underline{H}_2$–CH$_3$), 4.82-4.84 (m, 1 H, Cp–H), 4.87-4.89 (m, 1 H, Cp–H), 5.19-5.20 (m, 1 H, Cp–H), 5.30-5.32 (m, 1 H, Cp–H), 5.60-5.62 (m, 1 H, Cp–H), 5.84-5.85 (m, 1 H, Cp–H), 6.33-6.35 (m, 1 H, Cp–H), 6.37-6.39 (m, 1 H, Cp–H), 6.89-6.95 (m, 4 H, *p*Tol–CH), 7.03-7.07 (m, 4 H, *p*Tol–CH), 7.19-7.23 (m, 4 H, *p*Tol–CH), 7.80-7.84 (m, 2 H, *p*Tol–CH), 7.89-7.93 (m, 2 H, *p*Tol–CH) ppm.

^{13}C{^{1}H} NMR (126 MHz, C_6D_6, 305 K): 14.6 (N–CH$_2$–$\underline{C}$H$_3$), 16.8 (N–CH$_2$–$\underline{C}$H$_3$), 20.9 (2 x *p*Tol–CH$_3$), 21.0 (2 x *p*Tol–CH$_3$), 41.6 (N–$\underline{C}$H$_2$–CH$_3$), 48.5 (N–$\underline{C}$H$_2$–CH$_3$), 62.8 (Cp–C$_{exo}$), 63.3 (Cp–C$_{exo}$), 107.5 (Cp–CH), 108.2 (Cp–CH), 110.0 (Cp–CH), 110.7 (Cp–CH), 115.9 (Cp–CH), 116.1 (Cp–CH), 119.6 (Cp–CH), 121.4 (Cp–CH), 128.3* (*p*Tol–CH) 128.6 (*p*Tol–CH), 128.88 (*p*Tol–CH), 128.92 (*p*Tol–CH), 129.3 (*p*Tol–CH), 129.5 (*p*Tol–CH), 130.8 (*p*Tol–CH), 131.4 (*p*Tol–CH), 136.1 (*p*Tol–C$_q$), 136.26 (*p*Tol–C$_q$), 136.31 (*p*Tol–C$_q$), 136.34 (*p*Tol–C$_q$), 140.1 (Cp–C$_{ipso}$), 142.1 (*p*Tol–C$_q$), 142.4 (*p*Tol–C$_q$), 143.1 (*p*Tol–C$_q$), 143.2 (*p*Tol–C$_q$), 144.2 (Cp–C$_{ipso}$), 174.9 (N=C–O), 190.6 (N–C=O) ppm. * = Overlap with solvent signal.

^{1}H,^{15}N HMBC NMR (51 MHz, 500 MHz, C_6D_6, 305 K): 244.5 (Ti–OCN), 248.4 (Ti–NCO) ppm.

IR (ATR): ṽ = 3023 (w), 2962 (w), 2920 (w), 2866 (w), 1637 (m), 1606 (m), 1510 (m), 1191 (m), 1165 (m), 1143 (m), 1043 (m), 962 (m), 811 (s), 786 (s), 586 (m), 555 (m) cm^{-1}.

HRMS (EI): calcd. for $C_{46}H_{46}O_2N_2Ti$: m/z = 706.3033 [M^{+}]; found: m/z = 706.3032.

Synthesis of 3b

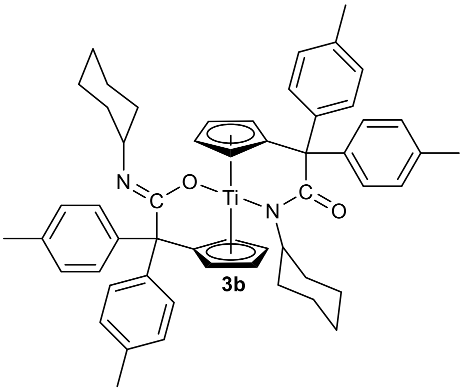

3b

Following the general procedure **(II)** gave **3b** as a yellow crystalline solid (163 mg, 0.200 mmol, 50%, mp: 160 °C (dec.)).

^{1}H NMR (500 MHz, C_6D_6, 305 K): 0.81-0.88 (m, 1 H, Cy–CH_2), 1.20-1.29 (m, 2 H, Cy–CH_2), 1.34-1.51 (m, 4 H, Cy–CH_2), 1.58-1.61 (m, 2 H, Cy–CH_2), 1.65-1.77 (m, 4 H, Cy–CH_2), 1.84-1.95 (m, 4 H, Cy–CH_2), 2.00-2.05 (m, 1 H, Cy–CH_2), 2.07 (s, 3 H, *p*Tol–CH_3), 2.10 (s, 3 H, *p*Tol–CH_3), 2.11 (s, 3 H, *p*Tol–CH_3), 2.12 (s, 3 H, *p*Tol–CH_3), 2.78-2.87 (m, 1 H, Cy–CH_2), 3.12-3.22 (m, 2 H, Cy–CH_2, Cy–CH), 3.74 (tt, J = 9.4 Hz, 3.8 Hz, 1 H, Cy–CH), 4.88-4.91 (m, 1 H, Cp–H), 4.97-5.00 (m, 1 H, Cp–H), 5.13-5.16 (m, 1 H, Cp–H), 5.33-5.36 (m, 1 H, Cp–H), 5.80-5.83 (m, 1 H, Cp–H), 6.01-6.04 (m, 1 H, Cp–H), 6.33-6.36 (m, 1 H, Cp–H), 6.52-6.55 (m, 1 H, Cp–H), 6.90-6.94 (m, 2 H, *p*Tol–CH), 6.92-6.98 (m, 2 H, *p*Tol–CH), 7.02-7.05 (m, 2 H, *p*Tol–CH), 7.05-7.09 (m, 2 H, *p*Tol–CH), 7.13-7.16 (m, 2 H, *p*Tol–CH), 7.39-7.43 (m, 2 H, *p*Tol–CH), 7.91-7.96 (m, 4 H, *p*Tol–CH) ppm.

^{13}C{^{1}H} NMR (126 MHz, C_6D_6, 305 K): 20.9 (2 x *p*Tol–CH_3), 21.0 (2 x *p*Tol–CH_3), 25.0 (Cy–CH_2), 25.4 (Cy–CH_2), 26.3 (Cy–CH_2), 26.8 (Cy–CH_2), 27.0 (Cy–CH_2), 27.4 (Cy–CH_2), 29.4 (Cy–CH_2), 30.8 (Cy–CH_2), 34.1 (Cy–CH_2), 35.4 (Cy–CH_2), 55.3 (Cy–CH), 62.9 (Cp–C_{exo}), 63.4 (Cp–C_{exo}), 67.1 (Cy–CH), 107.8 (Cp–CH), 109.1 (2 x Cp–CH), 110.1 (Cp–CH), 114.4 (Cp–CH), 116.9 (Cp–CH), 118.6 (Cp–CH), 125.3 (Cp–CH), 128.39 (2 x *p*Tol–CH), 128.43 (2 x *p*Tol–CH), 128.78 (2 x *p*Tol–CH), 128.81 (2 x *p*Tol–CH), 129.3 (2 x *p*Tol–CH), 129.5 (2 x *p*Tol–CH), 130.8 (2 x *p*Tol–CH), 131.2 (2 x *p*Tol–CH), 136.21 (*p*Tol–C_q), 136.25 (3 x *p*Tol–C_q), 138.9 (Cp–C_{ipso}), 141.9 (*p*Tol–C_q), 142.6 (*p*Tol–C_q), 143.5 (2 x *p*Tol–C_q), 144.9 (Cp–C_{ipso}), 175.0 (N=C–O), 188.5 (N–C=O) ppm.

^{1}H,^{15}N HMBC NMR (51 MHz, 500 MHz, C_6D_6, 305 K): Not detected.

IR (ATR): $\tilde{\nu}$ = 3091 (w), 3022 (w), 2964 (w), 2925 (m), 2851 (w), 1630 (m), 1602 (s), 1510 (m), 1267 (m), 1241 (m), 1231 (m), 1189 (m), 1161 (m), 1139 (m), 1120 (m), 995 (m), 984 (m), 943 (m), 840 (m), 826 (m), 811 (s), 801 (m), 788 (m), 730 (m), 523 (m), 516 (m), 507 (m) cm^{-1}.

HRMS (EI): calcd. for $C_{54}H_{58}O_2N_2Ti$: m/z = 814.3972 [M^+]; found: m/z = 814.3982.

Synthesis of 3c

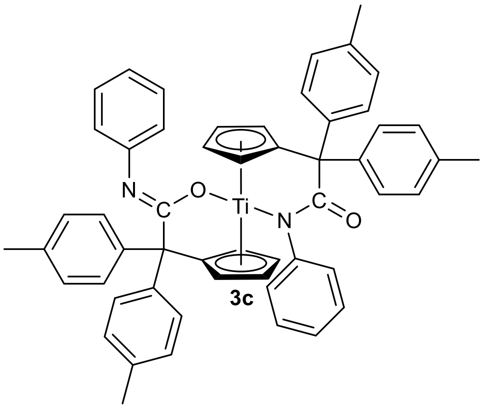

3c

Following the general procedure **(II)** gave **3c** as a green powder (247 mg, 0.310 mmol, 77%, mp: 170 °C (dec.)).

^{1}H NMR (500 MHz, C_6D_6, 305 K): 2.01 (s, 3 H, *p*Tol–CH_3), 2.10 (s, 3 H, *p*Tol–CH_3), 2.11 (s, 3 H, *p*Tol–CH_3), 2.12 (s, 3 H, *p*Tol–CH_3), 4.94-4.97 (m, 1 H, Cp–H), 5.00-5.03 (m, 1 H, Cp–H), 5.18-5.21 (m, 1 H, Cp–H), 5.42-5.45 (m, 2 H, 2 x Cp–H), 5.47-5.49 (m, 1 H, Cp–H), 5.52-5.56 (m, 1 H, Cp–H), 6.28-6.31 (m, 1 H, Cp–H), 6.85-6.91 (m, 5 H, Ar–CH), 6.94-6.97 (m, 4 H, Ar–CH), 7.00-7.03 (m, 4 H, Ar–CH), 7.23-7.30 (m, 5 H, Ar–CH), 7.45-7.49 (m, 2 H, Ar–CH), 7.52-7.55 (m, 2 H, Ar–CH), 7.58-7.61 (m, 2 H, Ar–CH), 7.71-7.74 (m, 2 H, Ar–CH) ppm.

^{13}C{^{1}H} NMR (126 MHz, C_6D_6, 305 K): 20.9 (2 x *p*Tol–CH_3), 21.0 (2 x *p*Tol–CH_3), 62.4 (Cp–C_{exo}), 63.8 (Cp–C_{exo}), 106.8 (Cp–CH), 108.5 (Cp–CH), 110.0 (Cp–CH), 113.4 (Cp–CH), 115.7 (Cp–CH), 117.0 (Cp–CH), 120.4 (Cp–CH), 121.8 (Cp–CH), 123.6 (Ar–CH), 124.4 (Ar–CH), 125.3 (Ar–CH), 128.3 (Ar–CH), 128.8 (Ar–CH), 129.0 (Ar–CH), 129.1 (Ar–CH), 129.3 (Ar–CH), 129.9 (Ar–CH), 130.2 (Ar–CH), 131.0 (Ar–CH), 136.1 (Ar–C_q), 136.3 (Ar–C_q), 136.6 (Ar–C_q), 136.7 (Ar–C_q), 141.26 (Ar–C_q), 141.33 (Cp–C_{ipso}), 141.7 (Ar–C_q), 142.1 (Ar–C_q), 143.1 (Ar–C_q), 144.8 (Cp–C_{ipso}), 148.4 (Ar–C_q), 153.3 (Ar–C_q), 175.5 (N=C–O), 191.7 (N–C=O) ppm.

IR (ATR): ṽ = 3023 (w), 2920 (w), 2864 (w), 1622 (m), 1588 (m), 1509 (m), 1484 (m), 1229 (m), 1172 (m), 1156 (m), 809 (s), 792 (m), 777 (m), 746 (s), 693 (s) cm^{-1}.

HRMS (EI or LIFDI): The target mass peak could not be detected. Instead, the mass peak of the complex **2c** was observed, indicating the loss of one molecule of PhNCO.

Synthesis of 4h

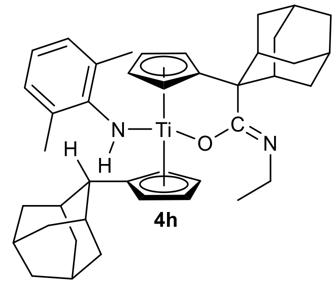

4h

In a glovebox, **1a** (206 mg, 0.400 mmol) was suspended in 5 mL of *n*-hexane and 2,6-dimethylaniline (49 mg, 0.400 mmol) was added. After stirring at ambient temperature for 16 h an orange suspension was obtained. The mother liquor was decanted, the residue washed with 3 mL of *n*-hexane and dried under high vacuum to yield **4h** as an orange powder (184 mg, 0.289 mmol, 72%, mp: 165 °C (dec.)).

^{1}H NMR (500 MHz, C_6D_6, 305 K): 1.45 (t, *J* = 7.3 Hz, 3 H, N–CH_2–C<u>H</u>$_3$), 1.49-1.54 (m, 1 H, Ad–H), 1.63-1.93 (m, 24 H, Ad–H, 2 x Ar–CH_3), 1.98-2.02 (m, 1 H, Ad–H), 2.10-2.12 (m, 1 H, Ad–H), 2.21-2.25 (m, 1 H, Ad–H), 2.32-2.36 (m, 3 H, Ad–H), 2.47-2.49 (m, 1 H, Ad–H), 2.97 (s, 1 H, Cp–C_{exo}H), 3.25-3.30 (m, 1 H, Ad–H), 3.47 (dq, *J* = 13.0 Hz, 7.2 Hz, 1 H, N–C<u>H</u>$_2$–CH_3), 3.57 (dq, *J* = 13.0 Hz, 7.2 Hz, 1 H, N–C<u>H</u>$_2$–CH_3), 4.63-4.68 (m, 1 H, Ad–H), 5.33-5.35 (m, 2 H, 2 x Cp–H), 5.36-5.38 (m, 2 H, 2 x Cp–H), 5.38-5.39 (m, 1 H, Cp–H), 5.54-5.56 (m, 1 H, Cp–H), 5.73-5.75 (m, 1 H, Cp–H), 5.88-5.89 (m, 1 H, Cp–H), 6.95-6.98 (m, 1 H, Ph–H), 7.02-7.13 (m, 2 H, Ph–H), 7.81 (s, 1 H, N–H) ppm.

^{13}C{^{1}H} NMR (126 MHz, C_6D_6, 305 K): 17.8 (N–CH_2–<u>C</u>H_3), 19.8 (Ph–CH_3), 20.5 (Ph–CH_3), 28.1 (Ad–CH), 28.4 (Ad–CH), 28.4 (Ad–CH), 28.8 (Ad–CH), 32.1 (Ad–CH), 32.5 (Ad–CH_2), 32.8 (Ad–CH_2), 32.8 (Ad–CH), 33.0 (2 x Ad–CH_2), 33.7 (Ad–CH), 35.5 (Ad–CH_2), 36.2 (Ad–CH), 37.3 (Ad–CH_2), 38.3 (Ad–CH_2), 39.0 (2 x Ad–CH_2), 39.9 (Ad–CH_2), 41.6 (N–<u>C</u>H_2–CH_3), 43.4 (Cp–C_{exo}H), 52.9 (Cp–C_{exo}), 104.6 (Cp–CH), 105.1 (Cp–CH), 107.1 (Cp–CH), 107.9 (Cp–CH), 111.8 (Cp–CH), 113.8 (Cp–CH), 115.2 (Cp–CH), 115.7 (Cp–CH), 123.5 (Ph–CH), 129.2 (Ph–CH), 134.0 (Ph–C_q), 139.0 (Cp–C_{ipso}), 147.5 (Cp–C_{ipso}), 159.1 (Ph–C_q), 177.1 (N=C–O) ppm.

^{1}H,^{15}N HMBC NMR (51 MHz, 500 MHz, C_6D_6, 305 K): 235.9 (N=C–O), 239.0 (d, *J* = 66 Hz, N–H) ppm.

IR (ATR): ṽ = 3280 (w), 2897 (m), 2849 (m), 1614 (s), 1468 (m), 1450 (m), 1251 (m), 1209 (s), 1184 (s), 1098 (m), 1069 (m), 1043 (m), 979 (m), 860 (m), 818 (s), 790 (m), 764 (s), 743 (m), 703 (m), 685 (m), 517 (m), 505 (m) cm^{-1}.

HRMS (EI): calcd. for $C_{41}H_{52}ON_2Ti$: m/z = 636.3554 [M^+]; found: m/z = 636.3559.

Synthesis of 4i

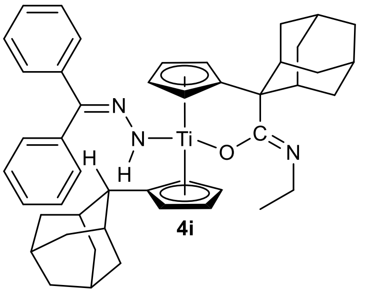

4i

In a glovebox, **1a** (155 mg, 0.300 mmol) and benzophenone hydrazone (59 mg, 0.300 mmol) were suspended in 5 mL of *n*-hexane. After stirring at ambient temperature for 16 h an orange suspension was obtained. The mother liquor was decanted, the residue washed with 3 mL of *n*-hexane and dried under high vacuum to yield **4i** as an orange powder (184 mg, 0.258 mmol, 86%, mp: 197 °C).

^{1}H NMR (500 MHz, C_6D_6, 305 K): 1.45 (t, *J* = 7.2 Hz, 3 H, N–CH_2–C<u>H</u>$_3$), 1.45-1.54 (m, 2 H, Ad–H), 1.67-1.92 (m, 19 H, Ad–H), 1.96-1.98 (m, 1 H, Ad–H), 2.00-2.05 (m, 2 H, Ad–H), 2.35-2.40 (m, 1 H, Ad–H), 2.58-2.62 (m, 1 H, Ad–H), 2.66-2.68 (m, 1 H, Ad–H), 3.10 (s, 1 H, Cp–C_{exo}H), 3.21 (dq, *J* = 12.4 Hz, 7.3 Hz, 1 H, N–C<u>H</u>$_2$–CH_3), 3.35 (dq, *J* = 12.3 Hz, 7.3 Hz, 1 H, N–C<u>H</u>$_2$–CH_3), 4.46-4.50 (m, 1 H, Ad–H), 5.38-5.41 (m, 1 H, Cp–H), 5.45-5.48 (m, 1 H, Cp–H), 5.49-5.53 (m, 1 H, Cp–H), 5.54-5.56 (m, 1 H, Cp–H), 5.68-5.72 (m, 1 H, Cp–H), 5.80-5.83 (m, 1 H, Cp–H), 6.28-6.31 (m, 1 H, Cp–H), 6.52-6.55 (m, 1 H, Cp–H), 7.04-7.15 (m, 5 H, Ph–H), 7.17-7.19 (m, 1 H, Ph–H), 7.20-7.25 (m, 2 H, Ph–H), 7.79-7.83 (m, 2 H, Ph–H), 9.92 (s, 1 H, N–H) ppm.

^{13}C{^{1}H} NMR (126 MHz, C_6D_6, 305 K): 17.3 (N–CH_2–<u>C</u>H$_3$), 28.3 (Ad–CH), 28.4 (Ad–CH), 28.5 (Ad–CH), 28.8 (Ad–CH), 32.4 (Ad–CH), 32.5 (Ad–CH_2), 32.8 (Ad–CH_2), 32.9 (Ad–CH), 33.0 (Ad–CH), 33.6 (Ad–CH), 34.0 (Ad–CH), 34.7 (Ad–CH_2), 35.9 (Ad–CH_2), 37.0 (Ad–CH), 38.3 (Ad–CH_2), 39.0 (Ad–CH_2), 39.3 (Ad–CH_2), 39.4 (Ad–CH_2), 40.6 (N–<u>C</u>H$_2$–CH_3), 43.5 (Cp–C_{exo}H), 53.3 (Cp–C_{exo}), 104.0 (Cp–CH), 107.1 (Cp–CH), 108.8 (Cp–CH), 110.1 (Cp–CH), 111.1 (Cp–CH), 112.8 (Cp–CH), 115.6 (Cp–CH), 116.2 (Cp–CH), 127.0 (Ph–CH), 127.4 (Ph–CH), 128.6 (Ph–CH), 128.7 (Ph–CH), 129.5 (Ph–CH), 130.0 (Ph–CH), 135.2 (Ph–C_q), 138.0 (Cp–C_{ipso}), 139.9 (Ph–C_q), 140.1 (Ph–C_q), 145.0 (Cp–C_{ipso}), 173.9 (N=C–O) ppm.

^{1}H,^{15}N HMBC NMR (51 MHz, 500 MHz, C_6D_6, 305 K): 223.2 (N=C–O), 277.3 (d, *J* = 63 Hz, N–H), 362.7 (<u>N</u>=NH) ppm.

IR (ATR): ṽ = 3226 (w), 3052 (w), 2958 (w), 2897 (m), 2848 (m), 1620 (s), 1488 (m), 1439 (m), 1209 (m), 1182 (m), 1096 (m), 1081 (s), 1065 (s), 1045 (m), 1026 (m), 848 (m), 828 (m), 818 (m), 808m (m), 762 (m), 701 (m), 692 (s), 678 (m), 627 (m), 597 (m), 527 (m) cm^{-1}.

HRMS (LIFDI): calcd. for $C_{46}H_{53}ON_3Ti$: m/z = 711.3663 [M^+]; found: m/z = 711.3651.

Synthesis of 4j/4j’

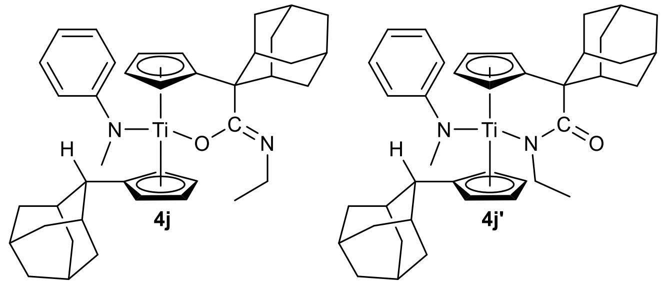

In a glovebox, **1a** (206 mg, 0.400 mmol) was suspended in 5 mL of *n*-hexane and 2,6-dimethylaniline (49 mg, 0.400 mmol) was added. After stirring at ambient temperature for 40 h a red suspension was obtained. The mother liquor was decanted, the residue washed with 3 mL of *n*-hexane and dried under high vacuum to yield **4j/4j’** as a red powder (167 mg, 0.268 mmol, 67%, mp: 163 °C). NMR data are given for the main product **4j**.

^{1}H NMR (500 MHz, C_6D_6, 305 K): 1.42 (t, *J* = 7.2 Hz, 3 H, N–CH$_2$–C$\underline{H}_3$), 1.46-1.54 (m, 2 H, Ad–H), 1.57-1.91 (m, 19 H, Ad–H), 1.94-1.97 (m, 1 H, Ad–H), 2.06-2.09 (m, 1 H, Ad–H), 2.19-2.22 (m, 2 H, Ad–H), 2.42-2.45 (m, 1 H, Ad–H), 2.85 (s, 1 H, Cp–C$_{exo}$H), 3.10-3.15 (m, 1 H, Ad–H), 3.18 (s, 3 H, N–CH$_3$), 3.39 (dq, *J* = 12.6 Hz, 7.3 Hz, 1 H, N–C$\underline{H}_2$–CH$_3$), 3.58 (dq, *J* = 12.7 Hz, 7.2 Hz, 1 H, N–C$\underline{H}_2$–CH$_3$), 4.48-4.53 (m, 1 H, Ad–H), 5.24-5.27 (m, 1 H, Cp–H), 5.47-5.50 (m, 1 H, Cp–H), 5.55-5.58 (m, 1 H, Cp–H), 5.60-5.63 (m, 2 H, 2 x Cp–H), 5.95-5.98 (m, 1 H, Cp–H), 6.08-6.11 (m, 1 H, Cp–H), 6.24-6.27 (m, 1 H, Cp–H), 6.48-6.52 (m, 2 H, Ph–H), 6.87-6.92 (m, 1 H, Ph–H), 7.18-7.22 (m, 2 H, Ph–H) ppm.

^{13}C{^{1}H} NMR (126 MHz, C_6D_6, 305 K): 17.5 (N–CH$_2$–$\underline{C}$H$_3$), 28.1 (Ad–CH), 28.4 (2 x Ad–CH), 28.6 (Ad–CH), 32.1 (Ad–CH), 32.4 (Ad–CH), 32.6 (Ad–CH$_2$), 32.8 (Ad–CH$_2$), 32.8 (Ad–CH$_2$), 32.9 (Ad–CH$_2$), 33.6 (Ad–CH), 35.0 (Ad–CH$_2$), 35.5 (Ad–CH), 37.2 (Ad–CH$_2$), 38.2 (Ad–CH$_2$), 39.0 (Ad–CH$_2$), 39.1 (Ad–CH$_2$), 39.7 (Ad–CH$_2$), 42.0 (N–$\underline{C}$H$_2$–CH$_3$), 43.7 (Cp–C$_{exo}$H), 51.0 (N–CH$_3$), 52.7 (Cp–C$_{exo}$), 104.2 (Cp–CH), 106.4 (Cp–CH), 110.1 (Cp–CH), 111.5 (Cp–CH), 112.4 (Cp–CH), 113.9 (Cp–CH), 116.3 (Cp–CH), 116.7 (Cp–CH), 120.5 (Ph–CH), 122.0 (2 x Ph–CH), 128.5 (2 x Ph–CH), 137.5 (Cp–C$_{ipso}$), 146.2 (Cp–C$_{ipso}$), 164.3 (Ph–C$_q$), 176.3 (N=C–O) ppm.

^{1}H,^{15}N HMBC NMR (51 MHz, 500 MHz, C_6D_6, 305 K): 200.0 (N–CH$_3$), 235.1 (N=C–O) ppm.

IR (ATR): ṽ = 2951 (w), 2896 (m), 2848 (m), 2771 (w), 1604 (s), 1585 (m), 1480 (m), 1448 (w), 1228 (m), 1206 (s), 1100 (m), 1067 (m), 1012 (m), 992 (m), 836 (m), 826 (s), 806 (m), 798 (s), 780 (m), 772 (m), 705 (s), 692 (m), 538 (w), 515 (m), 505 (s) cm^{-1}.

HRMS (LIFDI): calcd. for $C_{40}H_{50}ON_2Ti$: m/z = 622.3397 [M$^+$]; found: m/z = 622.3399.

Synthesis of 4k(R)/4k(S)

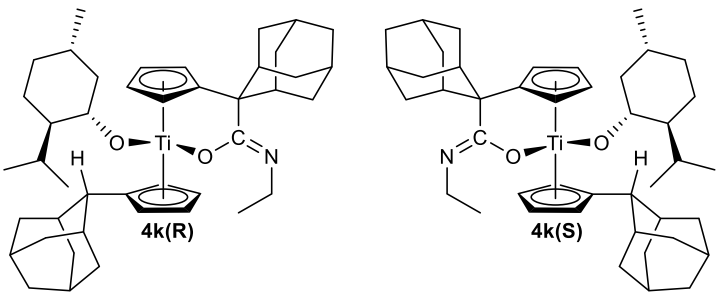

In a glovebox, **1a** (206 mg, 0.400 mmol) and analytically pure (-)-menthol (63 mg, 0.400 mmol) were suspended in 5 mL of *n*-hexane. After stirring at ambient temperature for 16 h a pale-yellow suspension was obtained. The mother liquor was decanted, the residue washed with 3 mL of *n*-hexane and dried under high vacuum to yield **4k(R)/4k(S)** as a pale-yellow powder (170 mg, 0.253 mmol, 63%, mp: 192 °C). Both isomers are present in a ratio of approximately 1:1. The signals cannot be clearly assigned and are therefore listed together below.

^{1}H NMR (500 MHz, C_6D_6, 305 K): 0.81 (d, *J* = 6.8 Hz, 3 H, ^{i}Pr–CH_3), 0.83 (d, *J* = 6.9 Hz, 3 H, ^{i}Pr–CH_3), 0.94 (d, *J* = 6.6 Hz, 3 H, CH–C$\underline{H}_3$), 0.96 (d, *J* = 6.6 Hz, 3 H, CH–C$\underline{H}_3$), 0.99 (d, *J* = 7.1 Hz, 3 H, ^{i}Pr–CH_3), 1.00 (d, *J* = 7.1 Hz, 3 H, ^{i}Pr–CH_3), 1.42 (t, *J* = 7.3 Hz, 3 H, N–CH_2–C$\underline{H}_3$), 1.43 (t, *J* = 7.3 Hz, 3 H, N–CH_2–C$\underline{H}_3$), 0.73-2.53 (m, 70 H, Ad–H, Menth–H), 2.99-3.04 (m, 1 H, Ad–H), 3.26-3.30 (m, 1 H, Ad–H), 3.32-3.40 (m, 4 H, Cp–C_{exo}H, N–C$\underline{H}_2$–CH_3), 3.55 (dq, *J* = 21.7 Hz, 7.3 Hz, 1 H, N–C$\underline{H}_2$–CH_3), 3.57 (dq, *J* = 21.5 Hz, 7.2 Hz, 1 H, N–C$\underline{H}_2$–CH_3), 3.81-3.89 (m, 2 H, ^{i}Pr–CH), 4.52-4.57 (m, 1 H, Ad–H), 4.59-4.63 (m, 1 H, Ad–H), 5.25-5.28 (m, 2 H, 2 x Cp–H), 5.57-5.59 (m, 1 H, Cp–H), 5.62-5.64 (m, 1 H, Cp–H), 5.66-5.68 (m, 2 H, 2 x Cp–H), 5.72-5.76 (m, 2 H, 2 x Cp–H), 5.78-5.80 (m, 1 H, Cp–H), 5.80-5.82 (m, 1 H, Cp–H), 5.83-5.85 (m, 1 H, Cp–H), 5.87-5.89 (m, 1 H, Cp–H), 5.90-5.92 (m, 1 H, Cp–H), 5.98-6.00 (m, 1 H, Cp–H), 6.01-6.03 (m, 2 H, 2 x Cp–H) ppm.

^{13}C{^{1}H} NMR (126 MHz, C_6D_6, 305 K): 16.3 (^{i}Pr–CH_3), 16.6 (^{i}Pr–CH_3), 17.5 (N–CH_2–$\underline{C}H_3$), 17.6 (N–CH_2–$\underline{C}H_3$), 22.0 (2 x ^{i}Pr–CH_3), 22.7 (2 x Menth–CH_3), 23.2 (CH_2), 23.3 (CH_2), 25.1 (CH), 25.6 (CH), 28.1 (CH), 28.2 (CH), 28.5 (2/3 x CH), 28.7 (CH), 28.8 (CH), 32.3 (CH), 32.3 (CH), 32.3 (CH), 32.4 (CH), 32.5 (CH), 32.6 (CH_2), 32.8 (CH_2), 32.9 (CH_2), 32.9 (CH_2), 32.9 (CH_2), 33.0 (CH_2), 33.0 (CH_2), 33.2 (CH_2), 33.3 (CH), 33.5 (CH), 35.1 (CH_2), 35.2 (CH_2), 35.3 (CH_2), 35.4 (CH_2), 35.8 (CH), 35.9 (CH), 37.3 (CH_2), 37.3 (CH_2), 37.3 (CH_2), 38.4 (2 x CH_2), 39.0 (CH_2), 39.1 (CH_2), 39.2 (CH_2), 39.2 (CH_2), 39.7 (CH_2), 39.8 (CH_2), 41.3 (N–$\underline{C}H_2$–CH_3), 41.5 (N–$\underline{C}H_2$–CH_3), 43.7 (Cp–C_{exo}H), 43.8 (Cp–C_{exo}H), 46.4 (CH_2), 47.9 (CH_2), 52.5 (CH), 52.7 (Cp–C_{exo}), 52.8 (Cp–C_{exo}), 53.3 (CH), 88.4 (^{i}Pr–CH), 88.5 (^{i}Pr–CH), 106.5 (Cp–CH), 106.7 (Cp–CH), 107.2 (Cp–CH), 107.5 (Cp–CH), 109.7 (Cp–CH), 109.8 (Cp–CH), 110.0 (Cp–CH), 110.6 (Cp–CH), 110.8 (Cp–CH), 112.3 (Cp–CH), 112.9 (Cp–CH), 117.0 (Cp–CH), 117.1 (Cp–CH),

117.4 (Ph–CH), 118.5 (Cp–CH), 136.6 (Cp–C_{ipso}), 136.6 (Cp–C_{ipso}), 147.5 (Cp–C_{ipso}), 148.1 (Cp–C_{ipso}), 176.6 (N=C–O), 177.2 (N=C–O) ppm.

^{1}H,^{15}N HMBC NMR (51 MHz, 500 MHz, C_6D_6, 305 K): 232.2 (N=C–O), 237.1 (N=C–O) ppm.

IR (ATR): $\tilde{\nu}$ = 2956 (w), 2897 (s), 2847 (m), 2808 (w), 1625 (s), 1467 (w), 1449 (m), 1365 (w), 1353 (w), 1317 (w), 1201 (m), 1184 (m), 1143 (w), 1079 (s), 1080 (s), 1063 (s), 1050 (s), 990 (w), 976 (w), 850 (w), 809 (s), 797 (s), 779 (m), 688 (m), 671 (s) cm^{-1}.

HRMS (LIFDI): calcd. for $C_{43}H_{61}O_2NTi$: m/z = 671.4176 [M^+]; found: m/z = 671.4183.

Synthesis of 4l/4l'

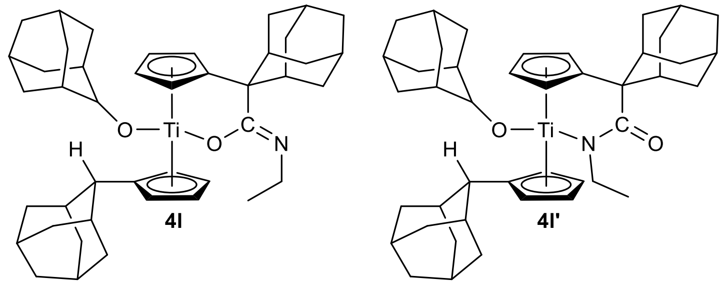

In a glovebox, **1a** (155 mg, 0.300 mmol) and 2,6-diphenylphenol (46 mg, 0.400 mmol) were suspended in 5 mL of *n*-hexane. After stirring at ambient temperature for 16 h, a pale-yellow suspension was obtained. The mother liquor was decanted, the residue washed with 3 mL of *n*-hexane and dried under high vacuum to yield **4l/4l'** as a pale-yellow powder (164 mg, 0.246 mmol, 82%, mp: 177 °C.). Both conformers are present in a ratio of approximately 1:1. The signals cannot be clearly assigned and are therefore listed together below.

^{1}H NMR (500 MHz, C_6D_6, 305 K): 0.94-0.97 (m, 1 H, Ad–H), 1.28 (t, *J* = 6.7 Hz, 3 H, CH_3), 1.40 (t, *J* = 7.3 Hz, 3 H, CH_3), 1.43-2.21 (m, 75 H, Ad–H), 2.22-2.26 (m, 1 H, Ad–H), 2.32-2.37 (m, 2 H, Ad–H), 2.53-2.58 (m, 1 H, Ad–H), 2.79-2.85 (m, 2 H, Ad–H, Cp–C_{exo}H), 3.22-3.31 (m, 2 H, Cp–C_{exo}H, N–C$\underline{H}_2$–CH_3), 3.46-3.56 (m, 2 H, N–C$\underline{H}_2$–CH_3), 3.72-3.80 (m, 2 H, Ad–H), 3.85 (dq, *J* = 13.1 Hz, 6.6 Hz, 1 H, N–C$\underline{H}_2$–CH_3),4.24-4.29 (m, 2 H, Ad–H), 4.57-4.63 (m, 1 H, Ad–H), 5.21-5.24 (m, 1 H, Cp–H), 5.24-5.27 (m, 1 H, Cp–H), 5.52-5.55 (m, 1 H, Cp–H), 5.57-5.60 (m, 1 H, Cp–H) 5.63-5.66 (m, 1 H, Cp–H), 5.73-5.77 (m, 2 H, 2 x Cp–H), 5.78-5.85 (m, 4 H, 4 x Cp–H), 5.88-5.91 (m, 1 H, Cp–H), 5.94-5.97 (m, 1 H, Cp–H), 6.00-6.03 (m, 1 H, Cp–H), 6.04-6.07 (m, 1 H, Cp–H), 6.12-6.15 (m, 1 H, Cp–H) ppm.

^{13}C{^{1}H} NMR (126 MHz, C_6D_6, 305 K): 15.3 (N–CH_2–$\underline{C}H_3$), 17.5 (N–CH_2–$\underline{C}H_3$), 27.8 (2 x Ad–CH), 27.9 (Ad–CH), 28.2 (Ad–CH), 28.3 (Ad–CH), 28.4 (3 x Ad–CH), 28.4 (Ad–CH), 28.5 (Ad–CH), 28.7 (Ad–CH), 28.8 (Ad–CH), 31.9 (Ad–CH), 32.2 (Ad–CH_2), 32.3 (Ad–CH_2), 32.4 (2 x Ad–CH_2), 32.6 (2 x Ad–CH_2), 32.7 (Ad–CH_2), 32.8 (Ad–CH_2), 32.9 (Ad–CH_2), 33.0 (2 x Ad–CH_2), 33.1 (2 x Ad–CH_2), 33.2 (Ad–CH_2), 33.3 (Ad–CH_2), 33.5 (Ad–CH_2), 33.9 (Ad–CH_2), 34.9 (Ad–CH_2), 35.2 (Ad–CH_2), 35.9 (Ad–CH), 36.2 (2 x Ad–CH), 36.2 (2 x Ad–CH), 36.5 (Ad–CH), 36.6 (Ad–CH), 36.7 (Ad–CH), 37.0 (2 x Ad–CH), 37.1 (3 x Ad–CH), 37.2 (Ad–CH), 38.0 (Ad–CH_2), 38.1 (Ad–CH_2), 38.2 (Ad–CH_2), 38.4 (Ad–CH_2), 38.7 (Ad–CH_2), 38.8 (Ad–CH_2), 39.1 (2 x Ad–CH_2), 39.6 (Ad–CH_2), 39.8 (Ad–CH_2), 40.9 (Ad–CH_2), 43.2 (Cp–C_{exo}H), 43.6 (Cp–C_{exo}H), 49.5 (N–$\underline{C}H_2$–CH_3), 52.3 (Cp–C_{exo}), 52.9 (Cp–C_{exo}), 92.1 (Ad–CH), 92.7 (Ad–CH), 106.1 (Cp–CH), 108.1 (Cp–CH), 108.5 (Cp–CH), 109.0 (Cp–CH), 109.6 (Cp–CH), 110.0 (Cp–CH), 110.7 (Cp–CH), 110.8 (Cp–CH), 111.7 (Cp–CH), 111.9 (Cp–CH), 112.3 (Cp–CH), 112.5 (Cp–CH),

112.7 (Cp–CH), 114.8 (Cp–CH), 116.0 (Cp–CH), 117.0 (Cp–CH), 133.5 (Cp–C_{ipso}), 138.1 (Cp–C_{ipso}), 138.5 (Cp–C_{ipso}), 146.8 (Cp–C_{ipso}), 175.6 (N=C–O), 188.0 (N–C=O) ppm.

^{1}H,^{15}N HMBC NMR (51 MHz, 500 MHz, C_6D_6, 305 K): 227.3 (N–C=O), 231.2 (N=C–O) ppm.

IR (ATR): $\tilde{\nu}$ = 2898 (s), 2847 (m), 1629 (m), 1587 (m), 1447 (m), 1182 (m), 1099 (s), 1089 (s), 1062 (s), 1029 (m), 836 (m), 814 (m), 807 (m), 796 (m), 718 (s), 645 (m), 620 (m) cm^{-1}.

HRMS (LIFDI): calcd. for $C_{43}H_{57}O_2NTi$: m/z = 667.3863 [M^+]; found: m/z = 667.3840.

Synthesis of 4m/4m'

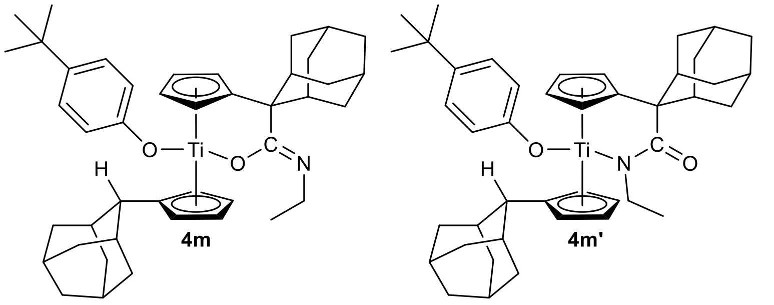

In a glovebox, **1a** (206 mg, 0.400 mmol) and 4-*tert*-butylphenol (60 mg, 0.400 mmol) were suspended in 5 mL of *n*-hexane. After stirring at ambient temperature for 16 h a yellow suspension was obtained. The mother liquor was decanted, the residue washed with 3 mL of *n*-hexane and dried under high vacuum to yield **4m/4m'** as a yellow powder (225 mg, 0.338 mmol, 84%, mp: 211 °C). NMR data are given for the assignable signals of the main product **4m'**.

^{1}H NMR (500 MHz, C_6D_6, 305 K): 1.26 (s, 9 H, $C(CH_3)_3$), 1.43 (t, *J* = 7.3 Hz, 3 H, N–CH_2–C<u>H</u>$_3$), 1.56-1.85 (m, 19 H, Ad–H), 1.94-1.96 (m, 2 H, Ad–H), 2.08-2.13 (m, 4 H, Ad–H), 2.28-2.31 (m, 1 H, Ad–H), 3.02 (s, 1 H, Cp–C_{exo}H), 3.39-3.43 (m, 1 H, Ad–H), 3.91 (dq, *J* = 13.2 Hz, 6.7 Hz, 1 H, N–<u>C</u>H_2–CH_3), 3.98 (dq, *J* = 13.6 Hz, 6.7 Hz, 1 H, N–C<u>H</u>$_2$–CH_3), 4.05-4.09 (m, 1 H, Ad–H), 5.26-5.28 (m, 1 H, Cp–H), 5.44-5.46 (m, 1 H, Cp–H), 5.53-5.54 (m, 1 H, Cp–H), 5.73-5.74 (m, 1 H, Cp–H), 5.79-5.80 (m, 1 H, Cp–H), 5.85-5.87 (m, 1 H, Cp–H), 5.99-5.99 (m, 1 H, Cp–H), 6.14-6.15 (m, 1 H, Cp–H), 6.48-6.51 (m, 2 H, Ph–CH), 7.19-7.22 (m, 2 H, Ph–CH) ppm.

^{13}C{^{1}H} NMR (126 MHz, C_6D_6, 305 K): 15.5 (N–CH_2–<u>C</u>H_3), 27.7 (Ad–CH), 28.2 (Ad–CH), 28.4 (Ad–CH), 28.6 (Ad–CH), 31.9 (C(<u>C</u>H_3)$_3$), 32.2 (Ad–CH), 32.6 (Ad–CH), 32.7 (Ad–CH_2), 33.0 (Ad–CH_2), 33.1 (Ad–CH_2), 33.2 (Ad–CH_2), 33.6 (Ad–CH), 34.2 (<u>C</u>(CH_3)$_3$), 35.5 (Ad–CH_2), 35.9 (Ad–CH_2), 36.4 (Ad–CH), 38.2 (Ad–CH_2), 38.9 (Ad–CH_2), 39.3 (Ad–CH_2), 39.7 (Ad–CH_2), 44.1 (Cp–C_{exo}H), 49.5 (N–<u>C</u>H_2–CH_3), 52.7 (Cp–C_{exo}), 105.6 (Cp–CH), 107.9 (Cp–CH), 109.9 (Cp–CH), 110.2 (Cp–CH), 111. (Cp–CH), 113.2 (Cp–CH), 114.9 (Cp–CH), 116.9 (Cp–CH), 117.4 (2 x Ph–CH), 126.3 (2 x Ph–CH), 140.3 (Cp–C_{ipso}), 142.4 (Ph–$C_q{}^t$Bu), 143.4 (Cp–C_{ipso}), 168.0 (Ph–C_q), 190.7 (O=C–N) ppm.

^{1}H,^{15}N HMBC NMR (51 MHz, 500 MHz, C_6D_6, 305 K): 238.0 (N–C=O) ppm.

IR (ATR): ṽ = 2894 (m), 2849 (m), 1596 (s), 1503 (s), 1487 (m), 1447 (m), 1267 (s), 1260 (s), 1245 (s), 1223 (s), 1174 (m), 1100 (m), 877 (m), 855 (m), 837 (s), 827 (s), 805 (m), 701 (m), 552 (m) cm^{-1}.

HRMS (LIFDI): calcd. for $C_{43}H_{55}O_2NTi$: m/z = 665.3707 [M^+]; found: m/z = 665.3707.

Synthesis of 4n/4n’

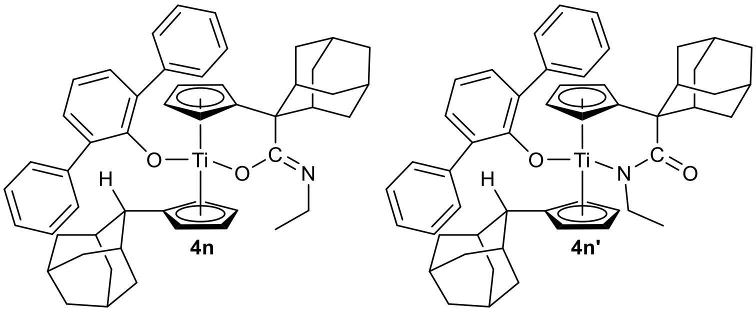

In a glovebox, **1a** (155 mg, 0.300 mmol) and 2,6-Diphenylphenol (74 mg, 0.400 mmol) were suspended in 5 mL of *n*-hexane. After stirring at ambient temperature for 16 h an orange suspension was obtained. The mother liquor was decanted, the residue washed with 3 mL of *n*-hexane and dried under high vacuum to yield **4n/4n’** as a yellow powder (161 mg, 0.211 mmol, 70%, mp: 216 °C). NMR data are given for the assignable signals of the main product **4n’**.

^{1}H NMR (500 MHz, C_6D_6, 305 K): 1.01 (t, *J* = 6.6 Hz, 3 H, N–CH$_2$–C$\underline{\text{H}}_3$), 1.36-1.40 (m, 1 H, Ad–H), 1.43-1.48 (m, 2 H, Ad–H), 1.50-1.83 (m, 18 H, Ad–H), 1.89-1.92 (m, 1 H, Ad–H), 2.01-2.07 (m, 2 H, Ad–H), 2.12-2.16 (m, 1 H, Ad–H), 2.24-2.28 (m, 1 H, Ad–H), 2.40 (dq, *J* = 13.5 Hz, 6.7 Hz, 1 H, N–C$\underline{\text{H}}_2$–CH$_3$), 2.61 (s, 1 H, Cp–C$_{exo}$H), 2.83 (dq, *J* = 13.2 Hz, 6.5 Hz, 1 H, N–C$\underline{\text{H}}_2$–CH$_3$), 3.09-3.14 (m, 1 H, Ad–H), 4.06-4.09 (m, 1 H, Ad–H), 4.26-4.29 (m, 1 H, Cp–H), 5.07-5.11 (m, 1 H, Cp–H), 5.54-5.57 (m, 1 H, Cp–H), 5.57-5.59 (m, 2 H, 2 x Cp–H) 5.85-5.87 (m, 1 H, Cp–H), 5.94-5.95 (m, 1 H, Cp–H), 6.05-6.06 (m, 1 H, Cp–H), 6.83 (t, *J* = 7.4 Hz, 1 H, Ph–H), 7.17-7.19 (m, 2 H, Ph–H), 7.26-7.27 (m, 2 H, Ph–H), 7.31-7.35 (m, 4 H, Ph–H), 7.43-7.46 (m, 4 H, Ph–H) ppm.

^{13}C{^{1}H} NMR (126 MHz, C_6D_6, 305 K): 14.6 (N–CH$_2$–$\underline{\text{C}}$H$_3$), 27.5 (Ad–CH), 28.0 (Ad–CH), 28.6 (Ad–CH), 31.3 (Ad–CH), 32.4 (Ad–CH$_2$), 32.6 (Ad–CH$_2$), 32.9 (Ad–CH$_2$), 33.6 (Ad–CH), 33.7 (Ad–CH), 35.5 (Ad–CH$_2$), 36.1 (Ad–CH$_2$), 36.3 (Ad–CH), 37.9 (Ad–CH$_2$), 38.3 (Ad–CH$_2$), 38.6 (Ad–CH$_2$), 39.7 (Ad–CH$_2$), 43.0 (Cp–C$_{exo}$H), 48.6 (N–$\underline{\text{C}}$H$_2$–CH$_3$), 52.1 (Cp–C$_{exo}$), 104.2 (Cp–CH), 107.0 (Cp–CH), 110.2 (Cp–CH), 113.5 (Cp–CH), 114.2 (Cp–CH), 115.9 (Cp–CH), 119.5 (Ph–CH), 120.8 (Cp–CH), 121.8 (Cp–CH), 127.0 (Ph–CH), 128.6 (Ph–CH), 130.5 (Ph–CH), 131.5 (Ph–CH), 132.5 (Ph–C$_q$), 133.7 (Cp–C$_{ipso}$), 141.1 (Cp–C$_{ipso}$), 142.3 (2 x Ph–C$_q$), 166.8 (Ph–C$_q$), 191.6 (N–C=O) ppm.

^{1}H,^{15}N HMBC NMR (51 MHz, 500 MHz, C_6D_6, 305 K): 254.6 (N–C=O) ppm.

IR (ATR): ṽ = 2902 (m), 2849 (w), 1618 (m), 1449 (m), 1404 (m), 1235 (s), 1220 (m), 1100 (m), 855 (m), 843 (m), 825 (m), 803 (m), 758 (s), 704 (s), 611 (m) cm^{-1}.

HRMS (EI): calcd. for $C_{43}H_{55}NO_2Ti$: m/z = 761.3707 [M$^+$]; found: m/z = 761.3690.

Synthesis of 5o/5o'

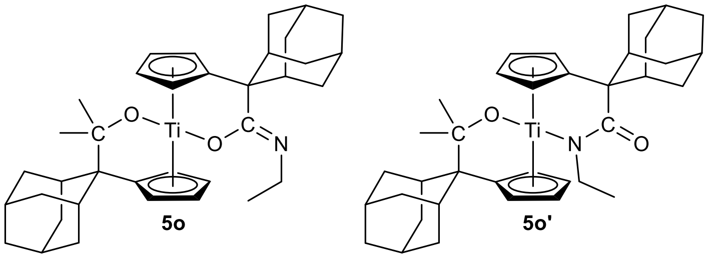

In a glovebox, **1a** (155 mg, 0.300 mmol) was suspended in 5 mL of *n*-hexane and acetone (23 mg, 0.400 mmol) was added. After stirring at ambient temperature for 16 h a yellow suspension was obtained. The mother liquor was decanted, the residue washed with 3 mL of *n*-hexane and dried under high vacuum to yield **5o/5o'** as a yellow powder (110 mg, 0.192 mmol, 64%, mp: 187 °C). NMR data are given for the assignable signals of the main product **5o**.

^{1}H NMR (500 MHz, C_6D_6, 305 K): 1.33 (s, 3 H, CH_3), 1.43 (t, *J* = 7.3 Hz, 3 H, N–CH_2–C$\underline{H}_3$), 1.44 (s, 3 H, CH_3), 1.47-1.51 (m, 1 H, Ad–H), 1.53-1.98 (m, 18 H, Ad–H), 2.09-2.13 (m, 2 H, Ad–H), 2.21-2.27 (m, 1 H, Ad–H), 2.34-2.46 (m, 3 H, Ad–H), 2.59-2.62 (m, 1 H, Ad–H), 3.06-3.11 (m, 1 H, Ad–H), 3.48 (dq, *J* = 12.7 Hz, 7.3 Hz, 1 H, N–C$\underline{H}_2$–CH_3), 3.59 (dq, *J* = 12.6 Hz, *J* = 7.3 Hz, 1 H, N–C$\underline{H}_2$–CH_3), 4.49-4.56 (m, 1 H, Ad–H), 5.14-5.16 (m, 1 H, Cp–H), 5.19-5.22 (m, 1 H, Cp–H), 5.22-5.24 (m, 1 H, Cp–H), 5.58-5.60 (m, 1 H, Cp–H), 5.60-5.62 (m, 1 H, Cp–H), 5.74-5.76 (m, 1 H, Cp–H), 6.00-6.02 (m, 1 H, Cp–H), 6.20-6.23 (m, 1 H, Cp–H) ppm.

^{13}C{^{1}H} NMR (126 MHz, C_6D_6, 305 K): 17.5 (N–CH_2–$\underline{C}H_3$), 27.7 (Ad–CH), 28.1 (Ad–CH), 28.3 (Ad–CH), 28.8 (Ad–CH), 30.5 (Ad–CH), 32.7 (Ad–CH), 33.2 (Ad–CH_2), 33.3 (Ad–CH_2), 33.6 (Ad–CH_2), 33.7 (Ad–CH_2), 33.8 (CH_3), 34.4 (Ad–CH), 34.5 (Ad–CH_2), 35.3 (Ad–CH_2), 35.9 (Ad–CH), 37.1 (Ad–CH_2), 37.2 (Ad–CH_2), 37.6 (Ad–CH_2), 39.6 (Ad–CH_2), 39.7 (Ad–CH_2), 41.0 (N–$\underline{C}H_2$–CH_3), 53.2 (C–C_{exo}), 54.3 (C–C_{exo}), 101.4 (Cp–CH), 102.6 (Cp–CH), 108.7 ($\underline{C}(CH_3)_2$), 109.2 (Cp–CH), 109.7 (Cp–CH), 111.0 (Cp–CH), 114.9 (Cp–CH), 116.0 (Cp–CH), 116.7 (Cp–CH), 146.7 (C–C_{ipso}), 158.2 (C–C_{ipso}), 176.2 (N=C–O) ppm.

^{1}H,^{15}N HMBC NMR (51 MHz, 500 MHz, C_6D_6, 305 K): 232.0 (N=C–O) ppm.

IR (ATR): ṽ = 2954 (w), 2916 (m), 2894 (s), 2849 (m), 1624 (s), 1453 (m), 1206 (m), 1178 (m), 1131 (m), 1121 (m), 1101 (m), 1064 (m), 1040 (m), 990 (m), 974 (m), 952 (m), 888 (m), 879 (m), 863 (m), 849 (m), 823 (s), 807 (w), 784 (w), 693 (w), 685 (w), 653 (w), 630 (w), 539 (m), 521 (m), 505 (s) cm^{-1}.

HRMS (LIFDI): calcd. for $C_{36}H_{47}O_2NTi$: m/z = 574.3159 [M^+]; found: m/z = 574.3155.

Synthesis of 5p/5p'

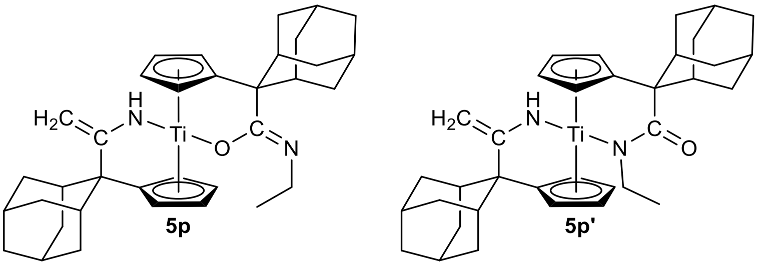

In the glovebox, **1a** (155 mg, 0.300 mmol) was suspended in 5 mL of *n*-hexane and acetonitrile (16 mg, 0.400 mmol) was added. After stirring at ambient temperature for 40 h an orange suspension was obtained. The mother liquor was then decanted, the residue washed with 3 mL of *n*-hexane and dried under vacuum to yield **5p/5p** as an orange powder (99 mg, 0.178 mmol, 59%, mp: 172 °C). NMR data are given for the assignable signals of the main product **5p**.

^{1}H NMR (500 MHz, C_6D_6, 305 K): 1.17 (t, *J* = 6.7 Hz, 3 H, N–CH_2–C<u>H</u>$_3$), 1.54-2.82 (m, 26 H, Ad–H), 3.42 (dq, *J* = 12.5 Hz, 6.7 Hz, 1 H, N–C<u>H</u>$_2$–CH_3), 3.50-3.55 (m, 1 H, Ad–H), 3.59 (dq, *J* = 12.7 Hz, *J* = 6.7 Hz, 1 H, N–C<u>H</u>$_2$–CH_3), 3.84 (s, 1 H, C=CH_2), 4.14-4.19 (m, 1 H, Ad–H), 4.29 (s, 1 H, C=CH_2), 5.12-5.17 (m, 2 H, 2 x Cp–H), 5.19-5.22 (m, 2 H, 2 x Cp–H), 5.29-5.32 (m, 1 H, Cp–H), 5.32-5.34 (m, 1 H, Cp–H), 5.35-5.38 (m, 1 H, Cp–H), 6.23-6.27 (m, 1 H, Cp–H), 8.05 (br.s, 1 H, N–H) ppm.

^{13}C{^{1}H} NMR (126 MHz, C_6D_6, 305 K): 15.4 (N–CH_2–<u>C</u>H_3), 27.6 (Ad–CH), 27.8 (Ad–CH), 28.5 (Ad–CH), 28.8 (Ad–CH), 32.9 (Ad–CH_2), 33.2 (Ad–CH_2), 33.4 (Ad–CH_2), 33.6 (Ad–CH), 33.7 (Ad–CH_2), 34.0 (Ad–CH), 34.2 (Ad–CH_2), 35.2 (Ad–CH_2), 35.6 (Ad–CH), 35.8 (Ad–CH_2), 36.0 (Ad–CH_2), 36.8 (Ad–CH), 37.0 (Ad–CH_2), 39.8 (Ad–CH_2), 46.5 (N–<u>C</u>H_2–CH_3), 51.1 (C–C_{exo}), 52.5 (C–C_{exo}), 86.7 (C=<u>C</u>H_2), 103.4 (Cp–CH), 104.2 (Cp–CH), 107.0 (Cp–CH), 107.5 (Cp–CH), 108.0 (Cp–CH), 109.8 (Cp–CH), 113.9 (Cp–CH), 114.0 (Cp–CH), 139.3 (C–C_{ipso}), 148.3 (C–C_{ipso}), 178.8 (<u>C</u>=CH_2), 190.6 (N–C=O) ppm.

^{1}H,^{15}N HMBC NMR (51 MHz, 500 MHz, C_6D_6, 305 K): 220.3 (N–C=O), 271.8 (N–H) ppm.

IR (ATR): ṽ = 3326 (w), 3225 (w), 2898 (m), 2851 (m), 1620 (s), 1585 (m), 1467 (m), 1447 (m), 1262 (m), 1244 (s), 1228 (m), 1211 (m), 1182 (m), 1096 (m), 1063 (m), 1047 (m), 823 (s), 796 (s), 684 (m), 505 (s) cm^{-1}.

HRMS (LIFDI): calcd. for $C_{35}H_{44}ON_2Ti$: m/z = 556.2928 [M^+]; found: m/z = 556.2934.

Synthesis of 5q/5q'

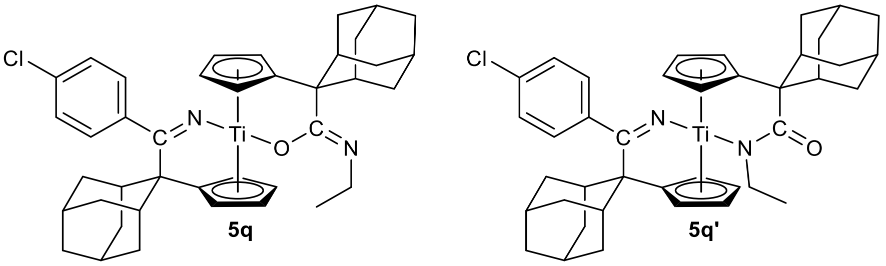

In a glovebox, **1a** (206 mg, 0.400 mmol) and *para*-chlorbenzonitrile (55 mg, 0.400 mmol) were suspended in 5 mL of *n*-hexane. After stirring at ambient temperature for 16 h a pink suspension was obtained. The mother liquor was decanted, the residue washed with 3 mL of *n*-hexane and dried under high vacuum to yield **5q/5q'** as a pink powder (197 mg, 0.302 mmol, 75%, mp: 194 °C). Both conformers are present in a ratio of approximately 1:1. The signals cannot be clearly assigned and are therefore listed together below.

^{1}H NMR (500 MHz, C_6D_6, 305 K): 1.01 (t, *J* = 7.1 Hz, 3 H, N–CH$_2$–C$\underline{H}_3$), 1.04 (t, *J* = 7.2 Hz, 3 H, N–CH$_2$–C$\underline{H}_3$), 1.13-1.18 (m, 2 H, Ad–H), 1.42-1.90 (m, 34 H, Ad–H), 1.95-1.98 (m, 2 H, Ad–H), 1.99-2.04 (m, 3 H, Ad–H), 2.10-2.22 (m, 5 H, Ad–H), 2.34-2.38 (m, 1 H, Ad–H), 2.39-2.42 (m, 1 H, Ad–H), 2.44-2.52 (m, 3 H, Ad–H), 2.59-2.62 (m, 1 H, Ad–H), 2.63-2.67 (m, 1 H, Ad–H), 2.85 (dq, *J* = 12.9 Hz, 7.3 Hz, 1 H, N–C$\underline{H}_2$–CH$_3$), 3.13 (dq, *J* = 12.8 Hz, 7.4 Hz, 1 H, N–C$\underline{H}_2$–CH$_3$), 3.36 (dq, *J* = 13.4 Hz, 6.9 Hz, 1 H, N–C$\underline{H}_2$–CH$_3$), 3.58-3.61 (m, 1 H, Ad–H), 3.92-3.98 (m, 1 H, Ad–H), 4.01 (dq, *J* = 12.7 Hz, 6.8 Hz, 1 H, 1 H, N–C$\underline{H}_2$–CH$_3$), 4.28-4.30 (m, 1 H, Ad–H), 5.01-5.02 (m, 2 H, 2 x Cp–H), 5.21-5.23 (m, 1 H, Cp–H), 5.39-5.40 (m, 1 H, Cp–H), 5.48-5.49 (m, 1 H, Cp–H), 5.56-5.57 (m, 1 H, Cp–H), 5.58- 5.60 (m, 1 H, Cp–H), 5.62-5.63 (m, 1 H, Cp–H), 5.67-5.68 (m, 1 H, Cp–H), 5.76-5.78 (m, 1 H, Cp–H), 5.81-5.83 (m, 1 H, Cp–H), 5.83-5.84 (m, 1 H, Cp–H), 5.98-6.00 (m, 1 H, Cp–H), 6.19-6.20 (m, 1 H, Cp–H), 6.29-6.30 (m, 1 H, Cp–H), 6.40-6.41 (m, 1 H, Cp–H), 6.68-6.99 (m, 4 H, Ph–H) 7.05-7.08 (m, 4 H, Ph–H) ppm.

^{13}C{^{1}H} NMR (126 MHz, C_6D_6, 305 K): 15.0 (N–CH$_2$–$\underline{C}$H$_3$), 16.8 (N–CH$_2$–$\underline{C}$H$_3$), 27.7 (Ad–CH), 27.7 (Ad–CH), 27.7 (Ad–CH), 27.9 (Ad–CH), 27.9 (Ad–CH), 28.4 (Ad–CH), 28.7 (Ad–CH), 28.8 (Ad–CH), 32.7 (Ad–CH$_2$), 32.8 (Ad–CH), 33.0 (Ad–CH$_2$), 33.1 (Ad–CH$_2$), 33.1 (Ad–CH$_2$), 33.7 (Ad–CH$_2$), 34.2 (Ad–CH$_2$), 34.2 (Ad–CH$_2$), 34.2 (Ad–CH), 34.2 (Ad–CH$_2$), 34.3 (Ad–CH), 34.4 (Ad–CH), 34.8 (Ad–CH$_2$), 34.9 (Ad–CH$_2$), 35.2 (Ad–CH$_2$), 35.3 (Ad–CH$_2$), 35.4 (Ad–CH$_2$), 35.6 (Ad–CH), 35.6 (Ad–CH), 35.6 (Ad–CH), 35.9 (Ad–CH$_2$), 36.2 (Ad–CH$_2$), 36.3 (Ad–CH), 36.8 (Ad–CH), 38.8 (Ad–CH$_2$), 39.3 (N–$\underline{C}$H$_2$–CH$_3$), 39.9 (Ad–CH$_2$), 40.2 (N–$\underline{C}$H$_2$–CH$_3$), 47.9 (N–$\underline{C}$H$_2$–CH$_3$), 52.7 (Cp–C$_{exo}$), 53.5 (Cp–C$_{exo}$), 61.0 (2 x Cp–C$_{exo}$), 103.7 (Cp–CH), 103.9 (Cp–CH), 104.4 (Cp–CH), 104.9 (Cp–CH), 106.7 (Cp–CH), 107.7 (Cp–CH), 108.7 (Cp–CH), 110.2 (Cp–CH), 110.5 (Cp–CH), 111.1 (Cp–CH), 111.1 (Cp–CH), 112.1 (Cp–CH), 114.1 (Cp–CH),

116.7 (Cp–CH), 116.8 (Cp–CH), 119.3 (Cp–CH), 132.9 (Ph–C_q), 133.0 (Ph–C_q), 138.9 (Cp–C_{ipso}), 142.5 (Cp–C_{ipso}), 143.4 (Ph–C_q), 143.6 (Ph–C_q), 150.4 (Cp–C_{ipso}), 150.6 (Cp–C_{ipso}), 172.3 (N=C–O), 191.3 (N–C=O), 199.9 (C=N), 200.0 (C=N) ppm.

^{1}H,^{15}N HMBC NMR (51 MHz, 500 MHz, C_6D_6, 305 K): 228.9, 236.7 ppm.

IR (ATR): ṽ = 2902 (m), 2849 (m), 1625 (m), 1588 (s), 1471 (m), 1447 (m), 1099 (m), 1086 (m), 1066 (m), 829 (s), 816 (s), 804 (m), 789 (m), 736 (m), 678 (m), 623 (m), 611 (m), 534 (m), 511 (s) cm^{-1}.

HRMS (LIFDI): calcd. for $C_{35}H_{44}ON_2Ti$: m/z = 556.2928 [M^+]; found: m/z = 556.2934.

NMR Spectra

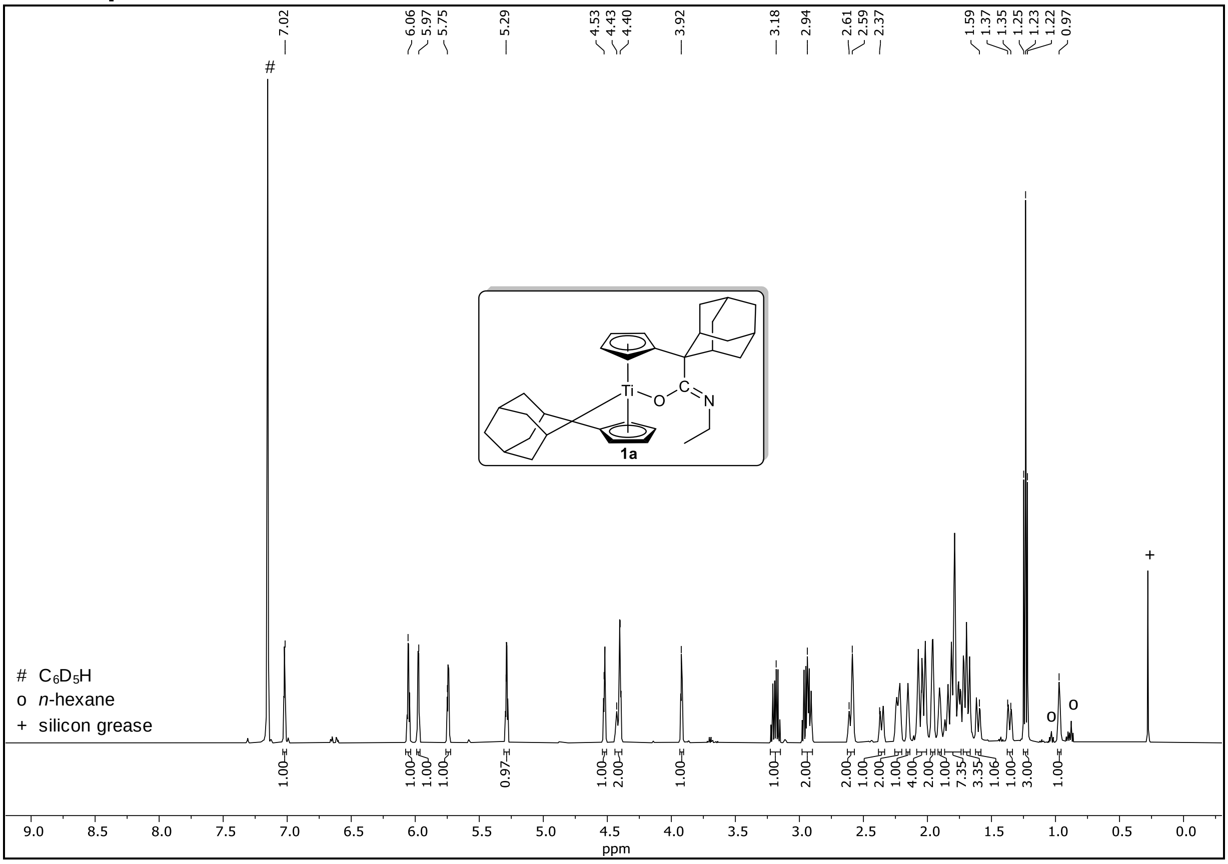

Figure S1: ^{1}H NMR spectrum of **1a** (500 MHz, C_6D_6, 305 K).

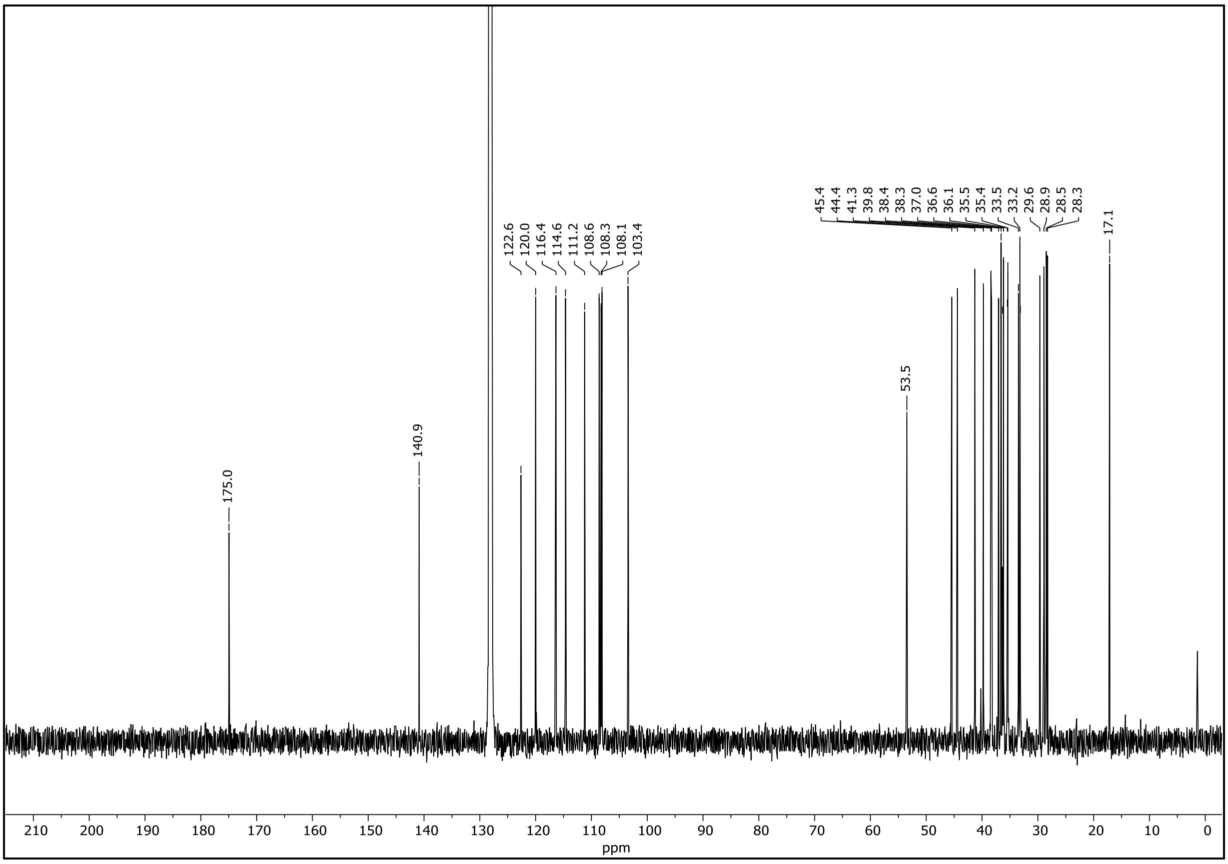

Figure S2: ^{13}C{^{1}H} NMR spectrum of **1a** (126 MHz, C_6D_6, 305 K).

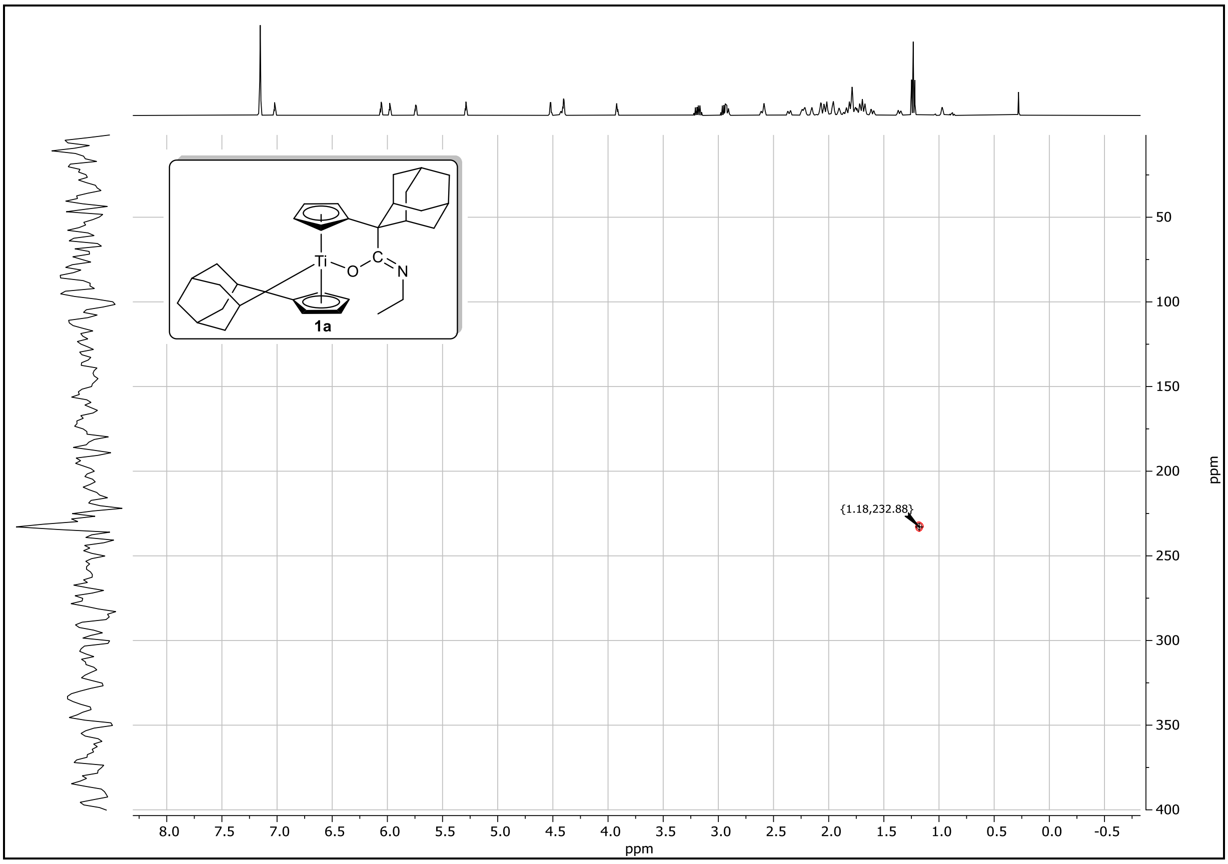

Figure S3: $^{1}H,^{15}N$ HMBC NMR spectrum of **1a** (500 MHz, 51 MHz, C_6D_6, 305 K).

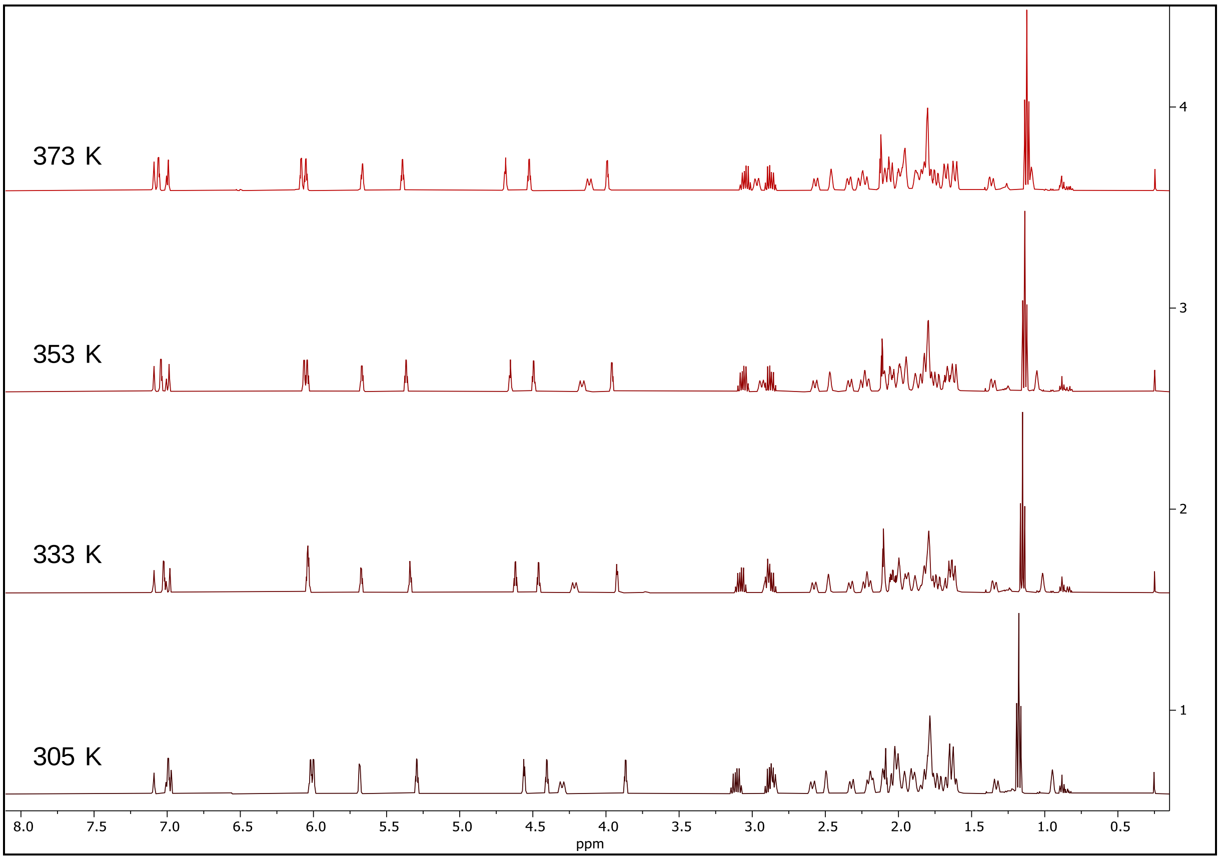

Figure S4: Variable temperature ^{1}H NMR spectrum of **1a** (500 MHz, toluene-d_8, 305 K - 373 K).

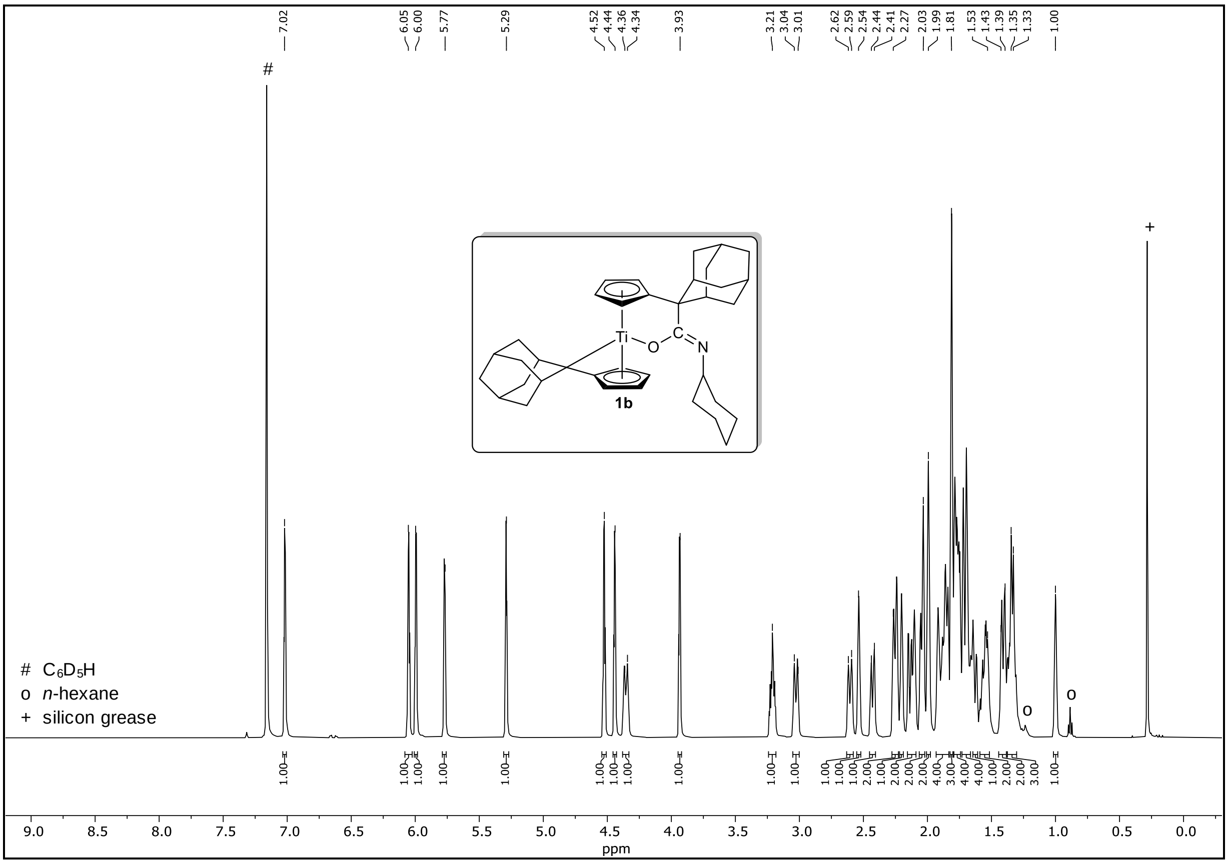

Figure S5: ^{1}H NMR spectrum of **1b** (500 MHz, C_6D_6, 305 K).

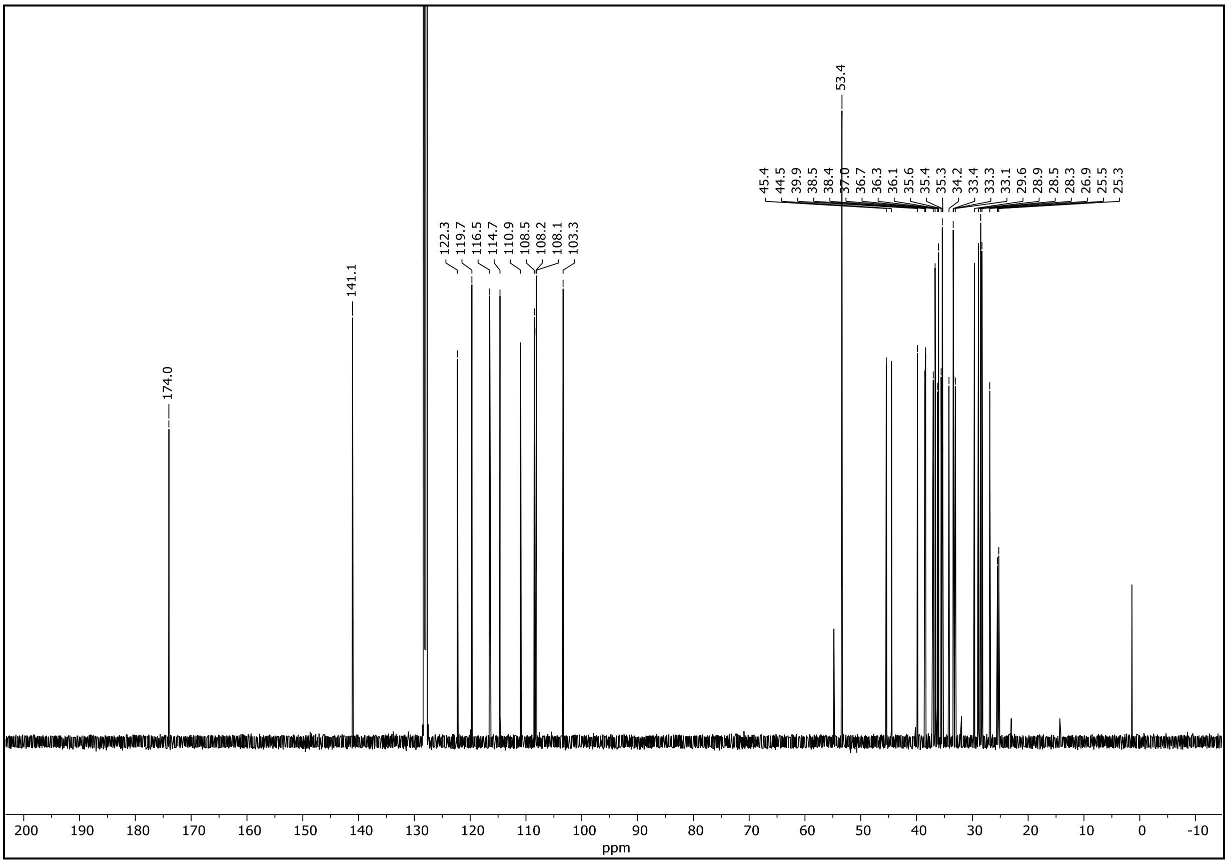

Figure S6: ^{13}C{^{1}H} NMR spectrum of **1b** (126 MHz, C_6D_6, 305 K).

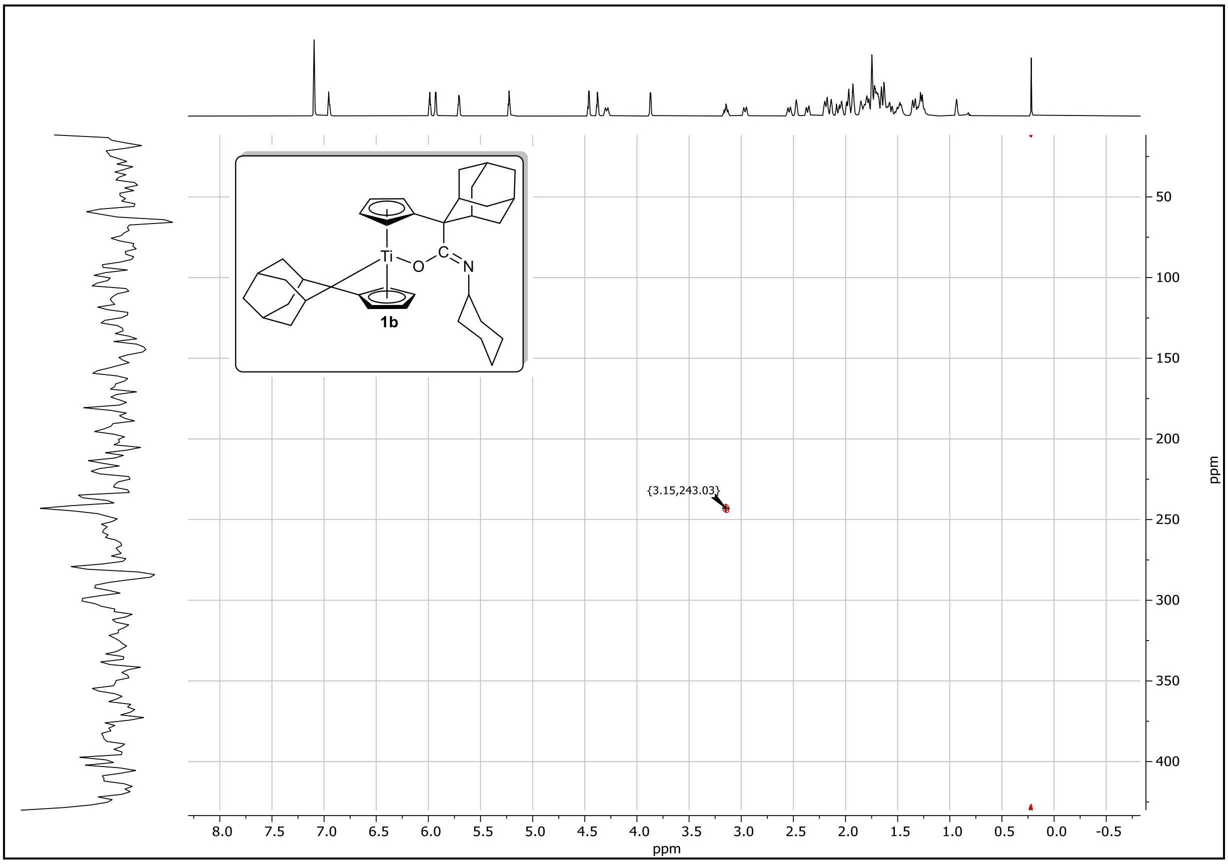

Figure S7: ^{1}H,^{15}N HMBC NMR spectrum of **1b** (500 MHz, 51 MHz, C_6D_6, 305 K).

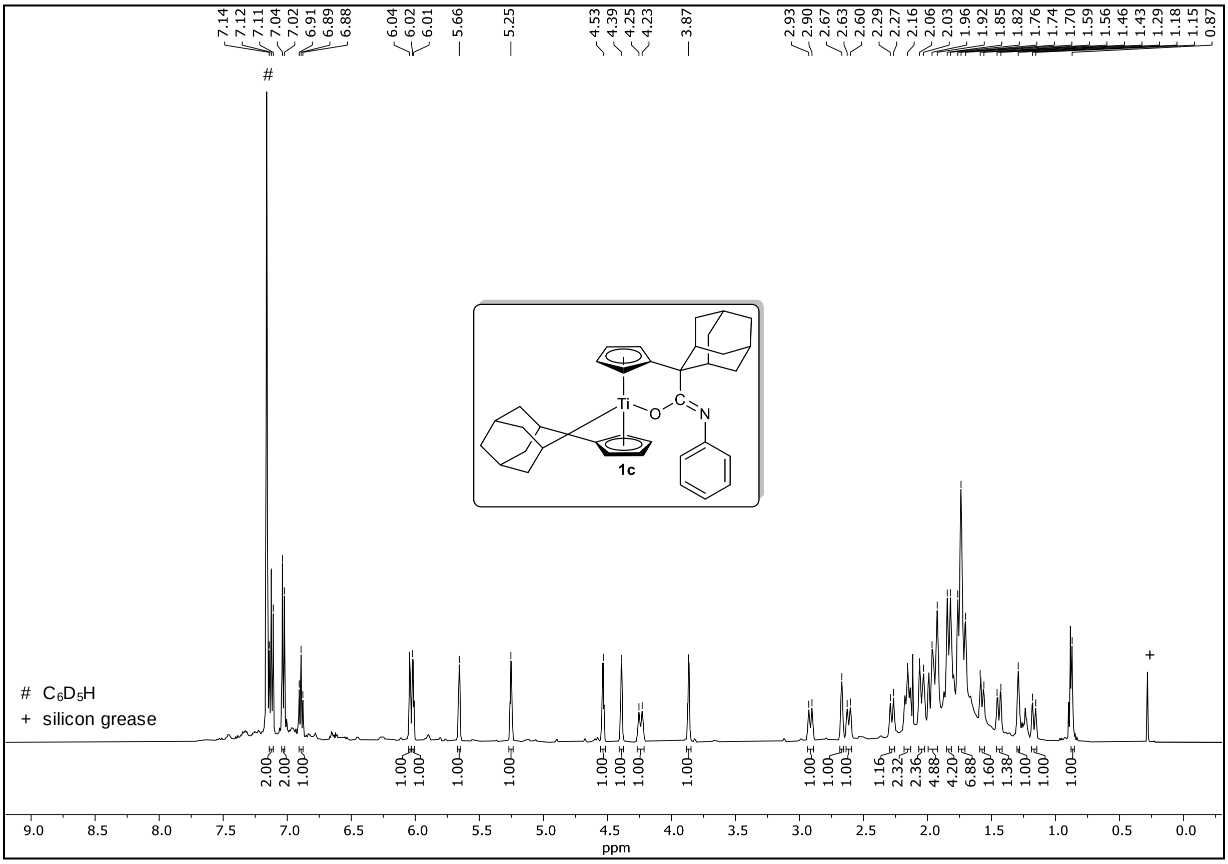

Figure S8: ^{1}H NMR spectrum of **1c** (500 MHz, C_6D_6, 305 K).

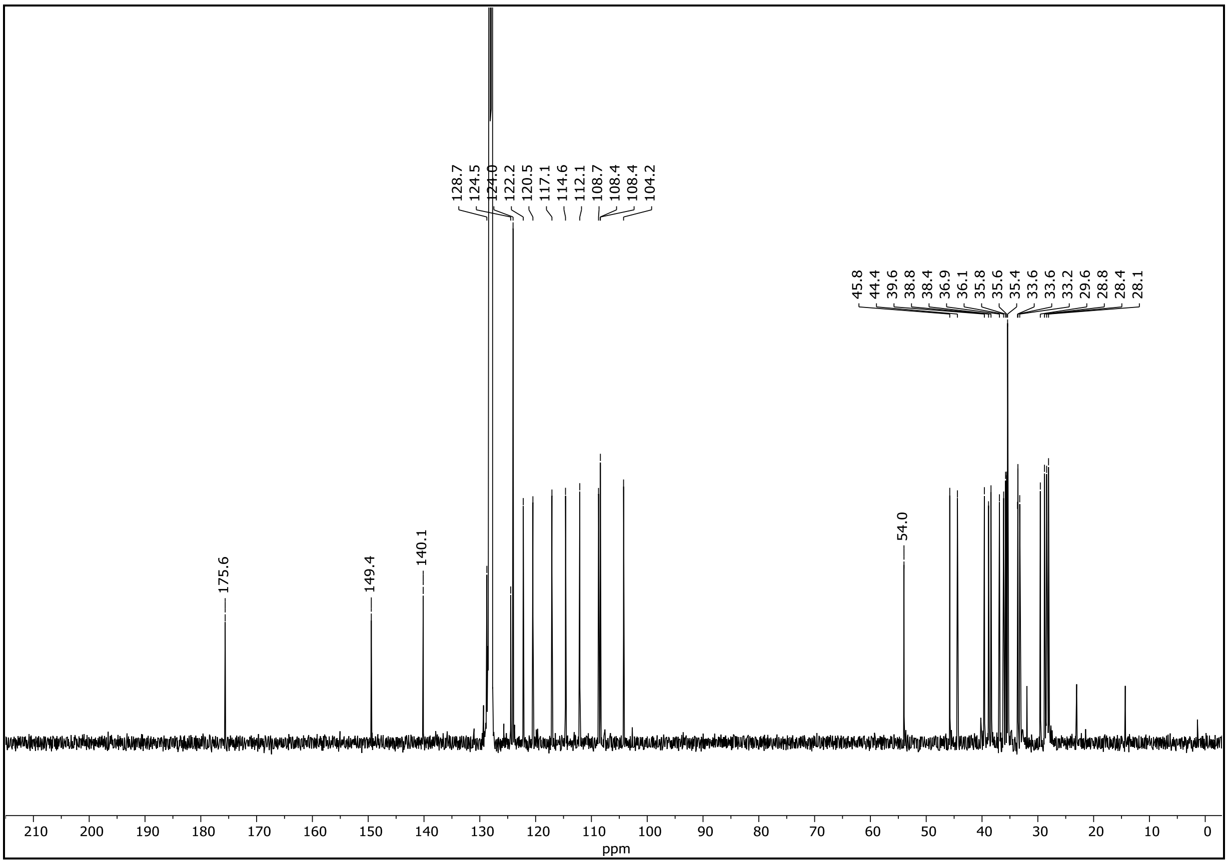

Figure S9: $^{13}C\{^{1}H\}$ NMR spectrum of **1c** (126 MHz, C_6D_6, 305 K).

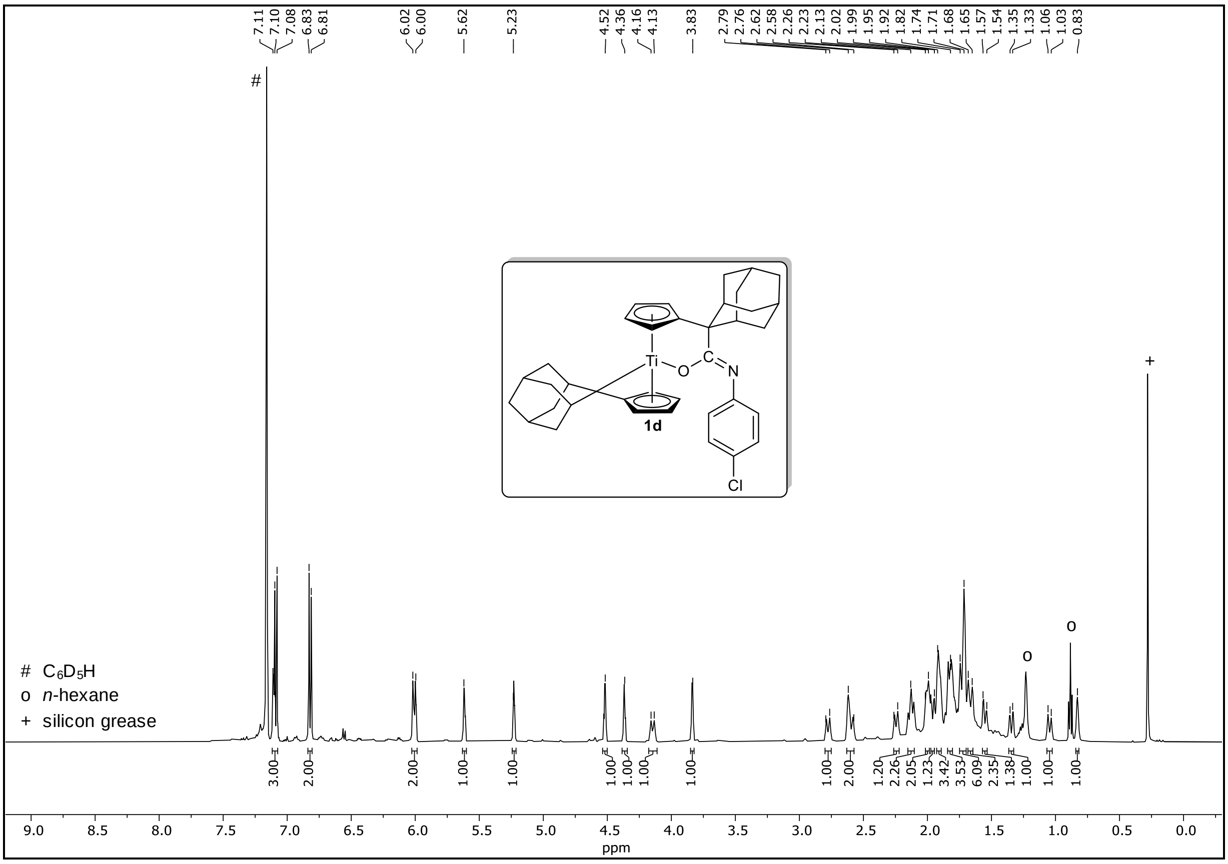

Figure S10: ^{1}H NMR spectrum of **1d** (500 MHz, C_6D_6, 305 K).

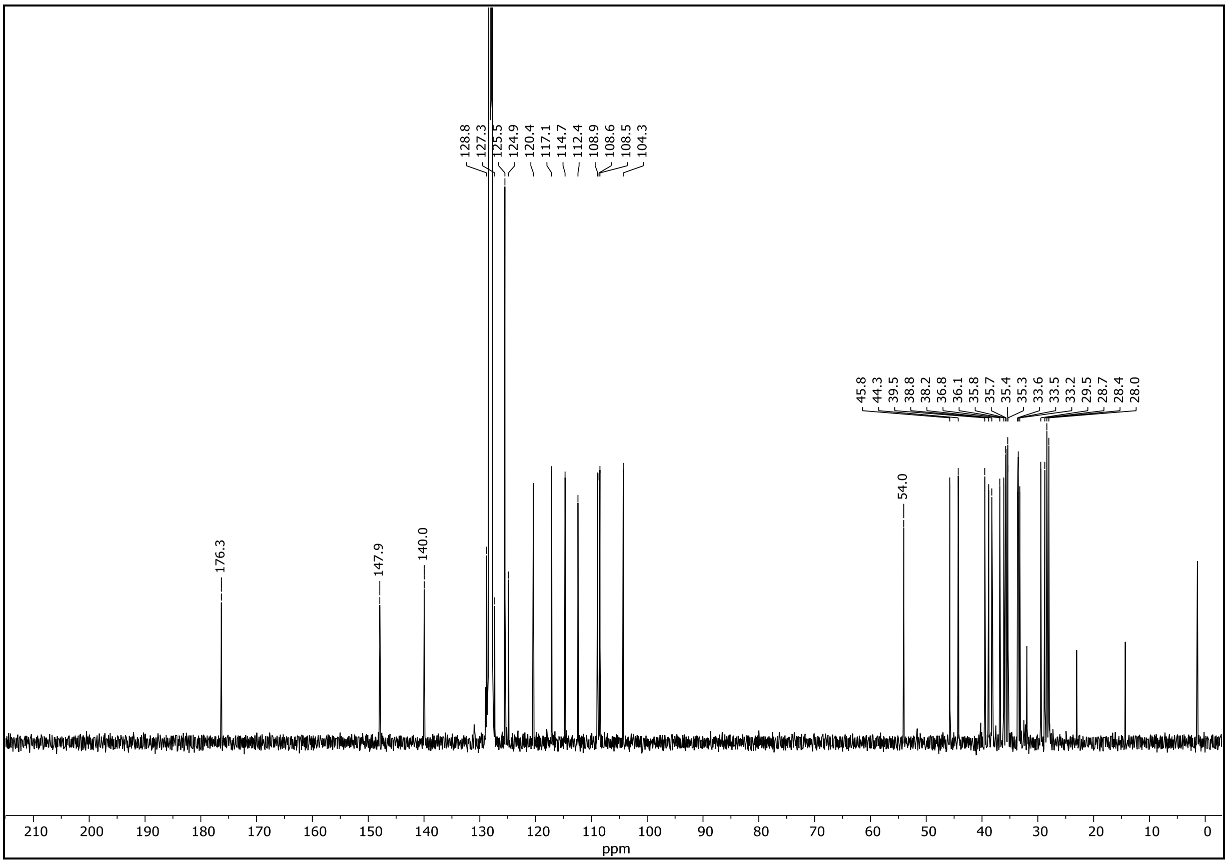

Figure S11: $^{13}C\{^{1}H\}$ NMR spectrum of **1d** (126 MHz, C_6D_6, 305 K).

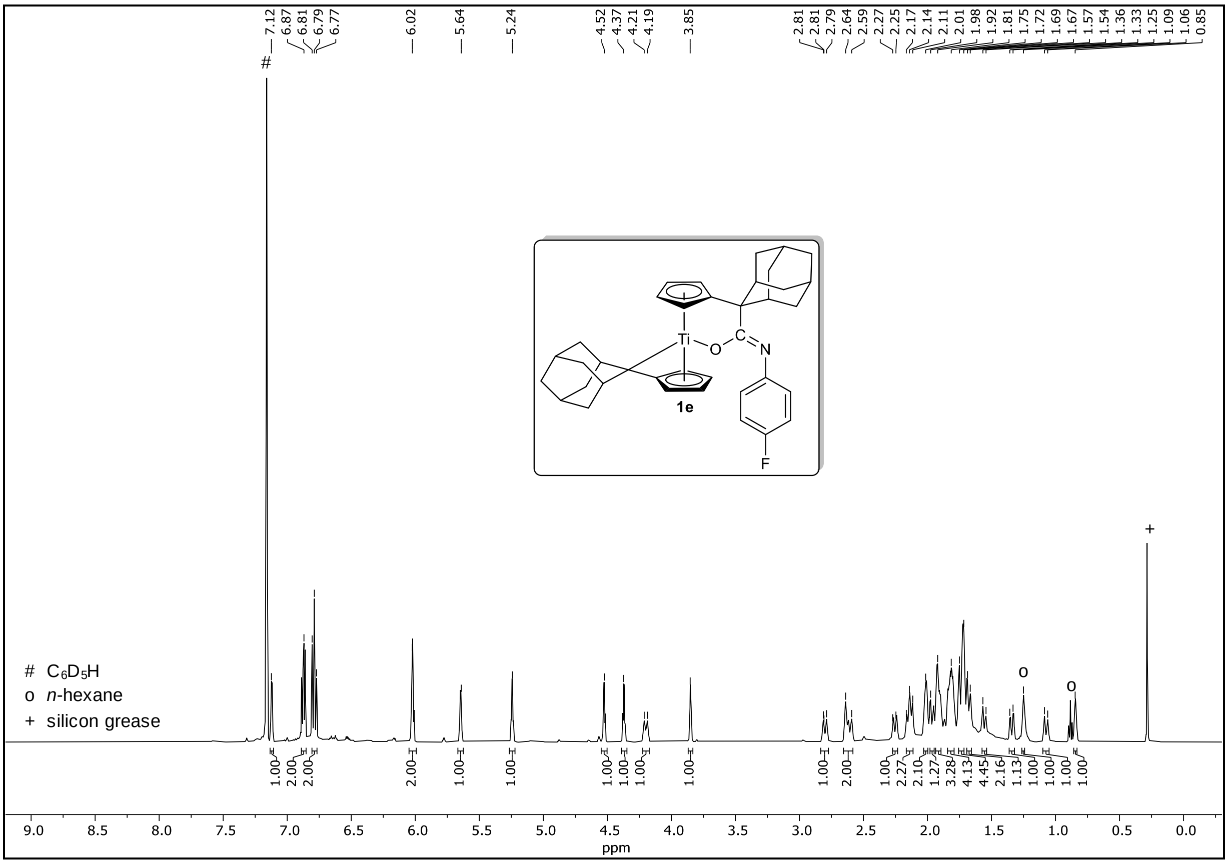

Figure S12: ^{1}H NMR spectrum of **1e** (500 MHz, C_6D_6, 305 K).

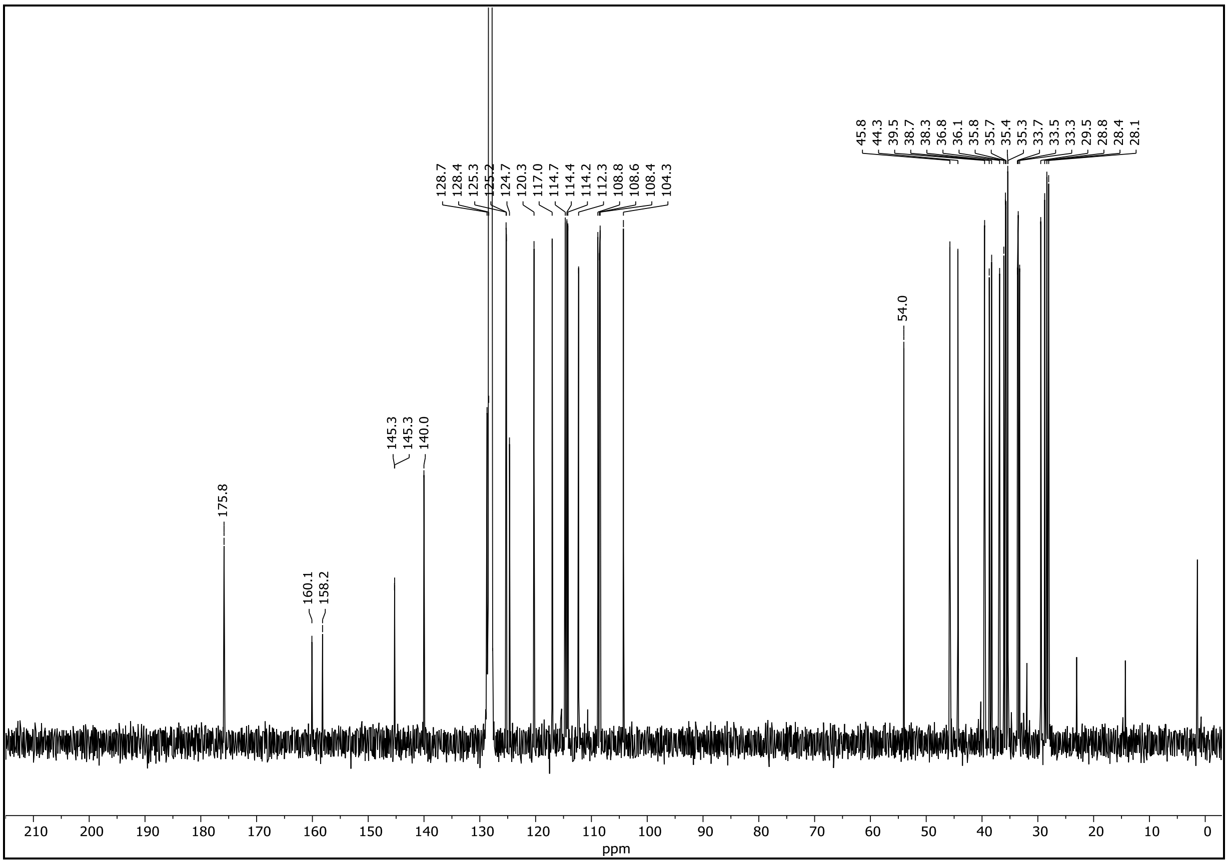

Figure S13: $^{13}C\{^1H\}$ NMR spectrum of **1e** (126 MHz, C_6D_6, 305 K).

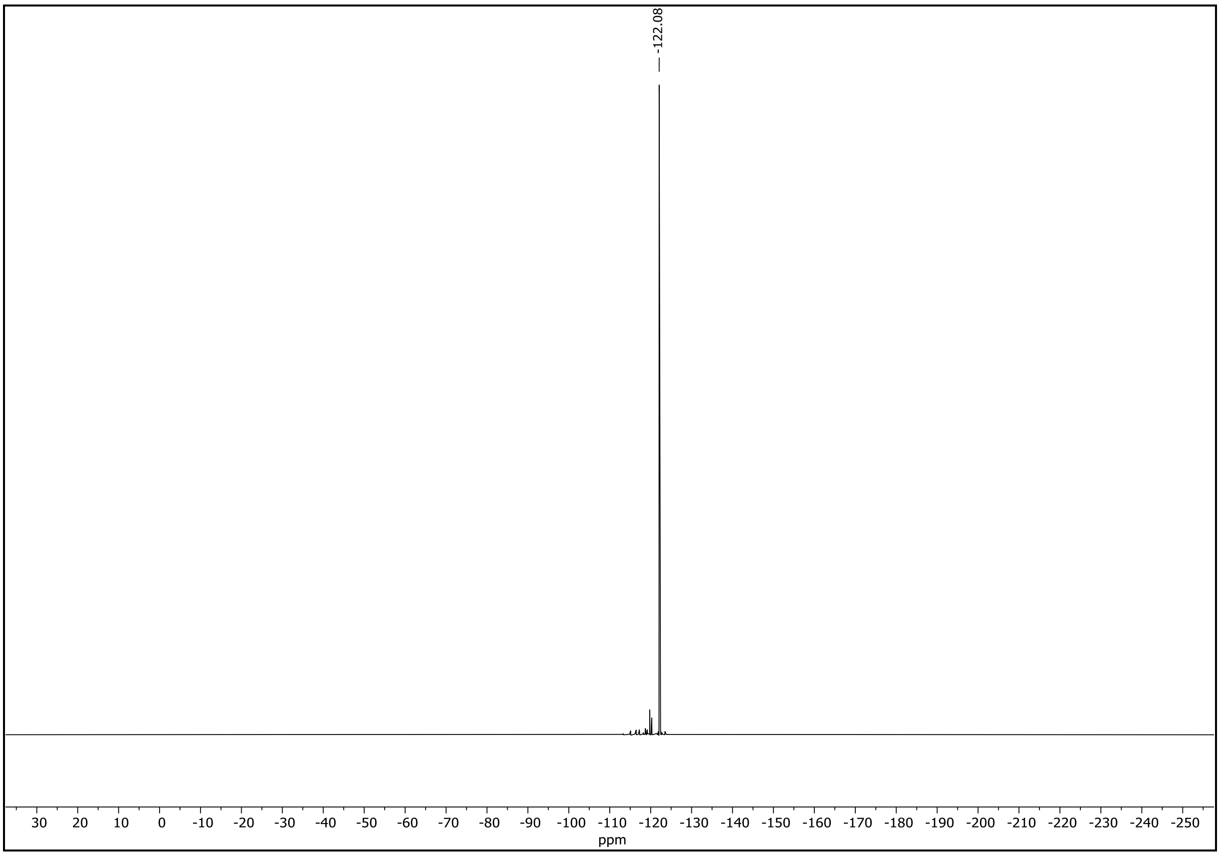

Figure S14: $^{19}F\{^1H\}$ NMR spectrum of **1e** (470 MHz, C_6D_6, 305 K).

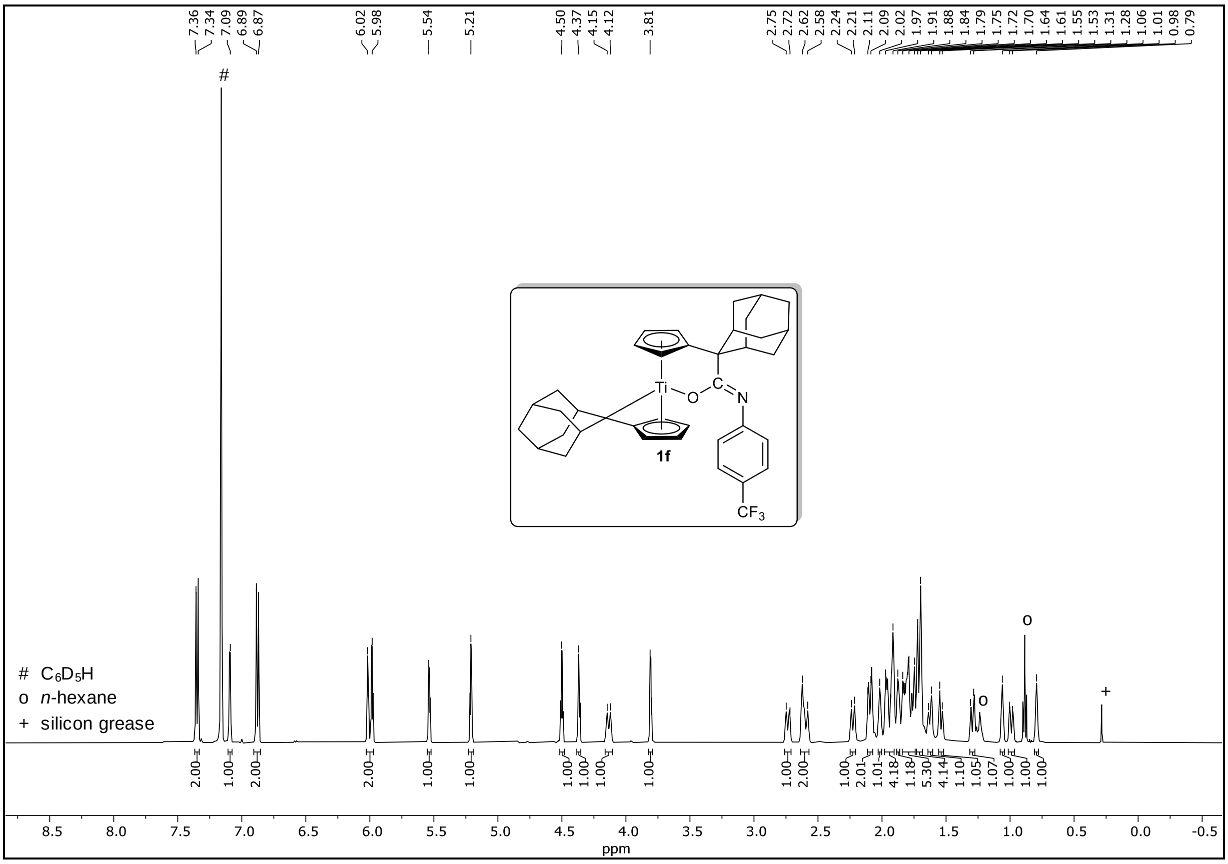

Figure S15: ^{1}H NMR spectrum of **1f** (500 MHz, C_6D_6, 305 K).

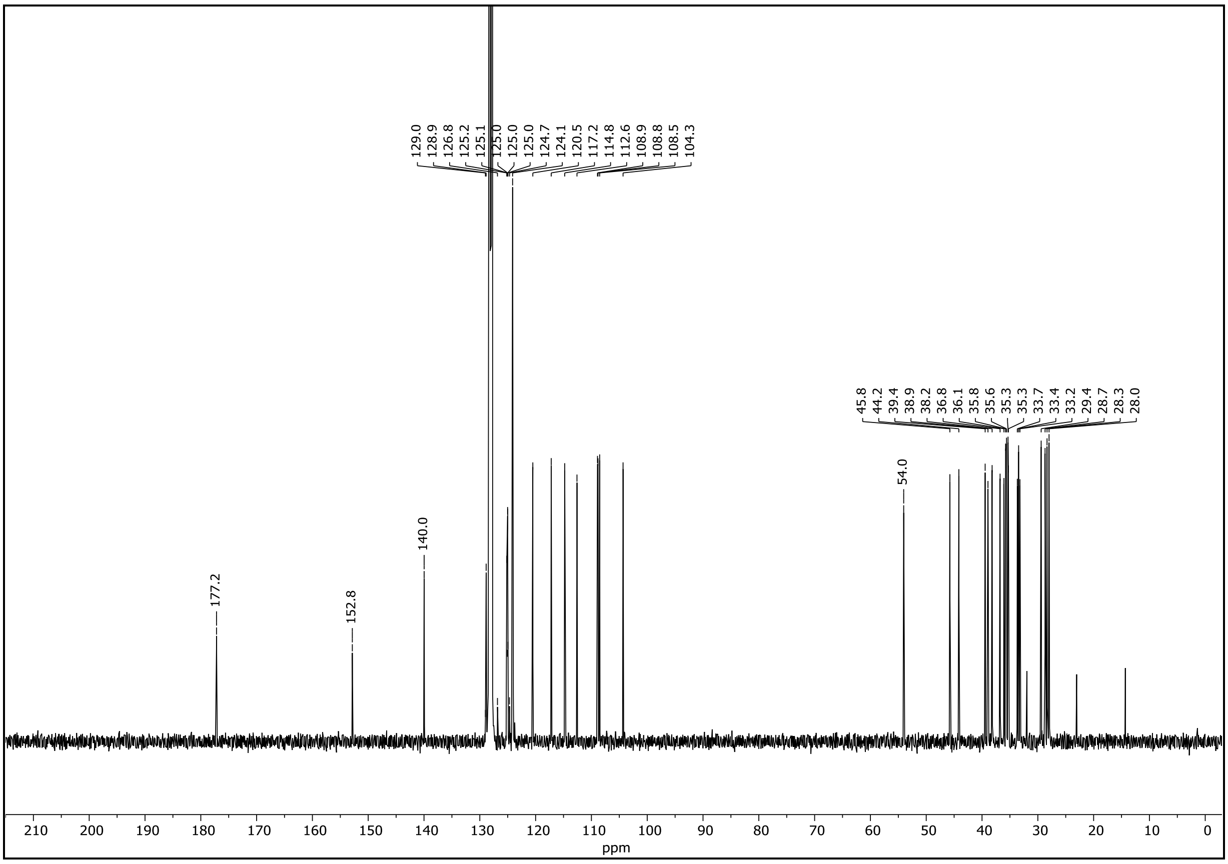

Figure S16: $^{13}C\{^{1}H\}$ NMR spectrum of **1f** (126 MHz, C_6D_6, 305 K).

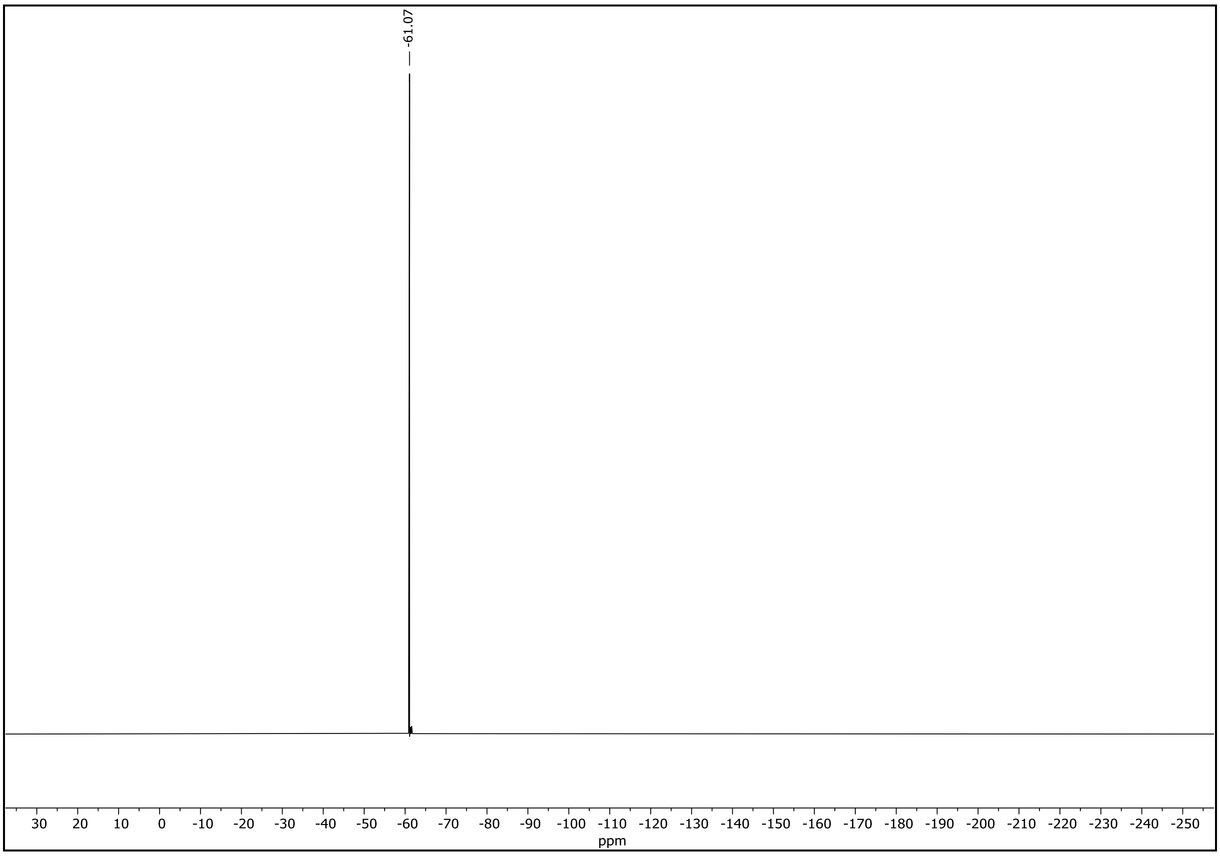

Figure S17: $^{19}F\{^1H\}$ NMR spectrum of **1f** (470 MHz, C_6D_6, 305 K).

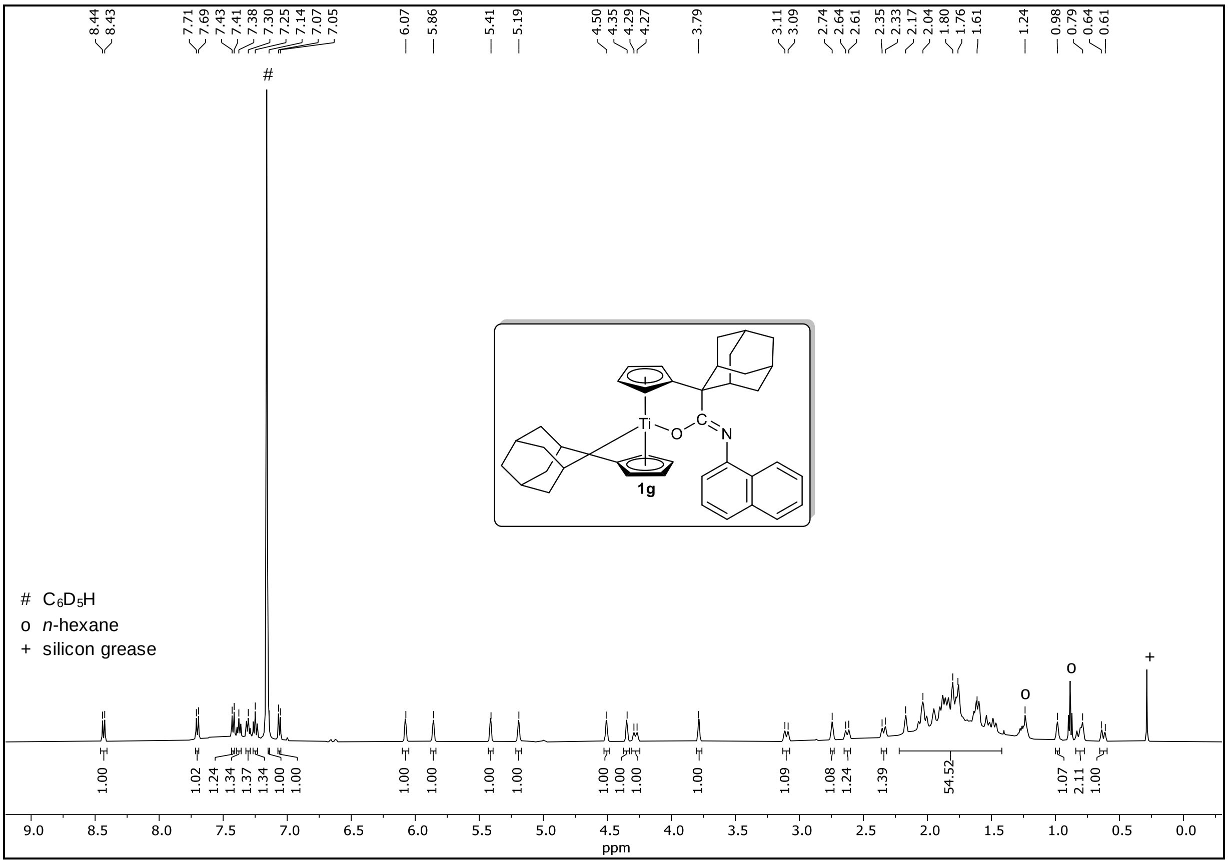

Figure S18: ^1H NMR spectrum of **1g** (500 MHz, C_6D_6, 305 K).

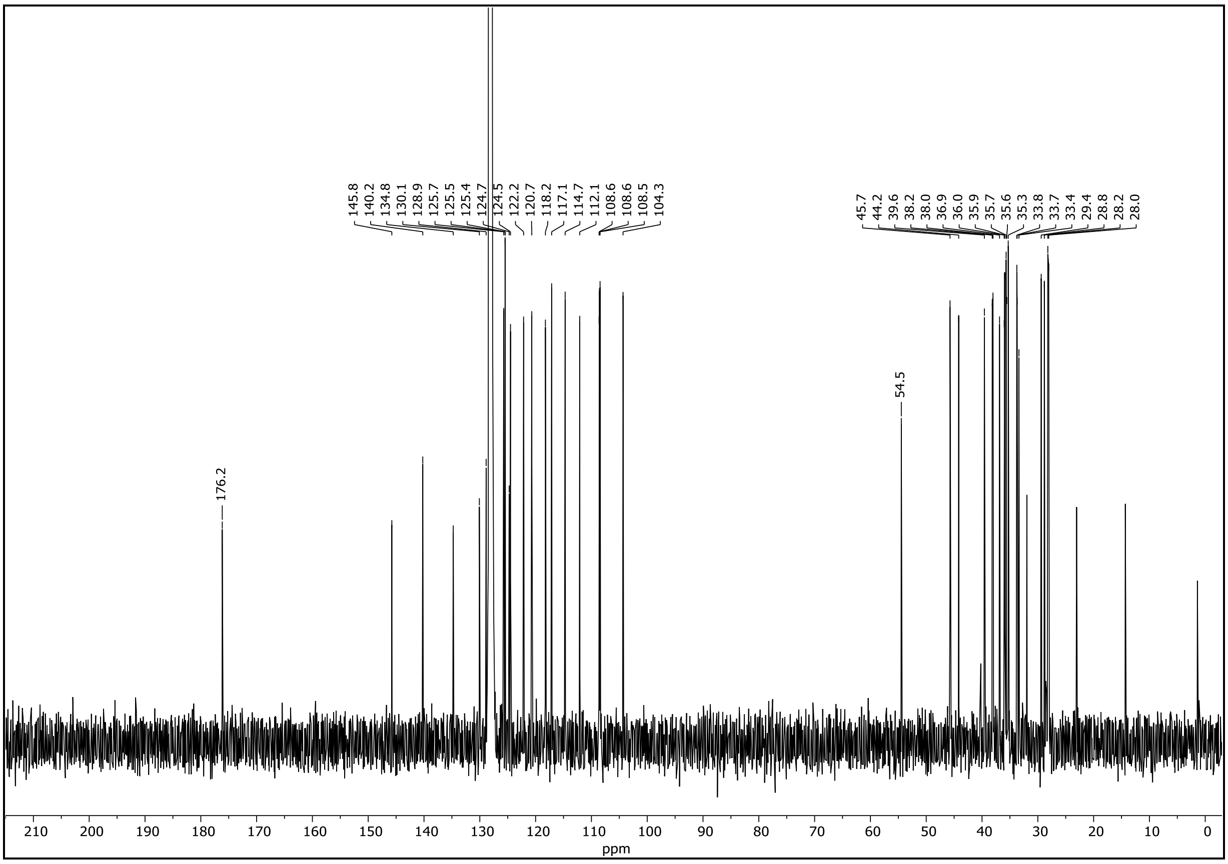

Figure S19: $^{13}C\{^{1}H\}$ NMR spectrum of **1g** (126 MHz, C_6D_6, 305 K).

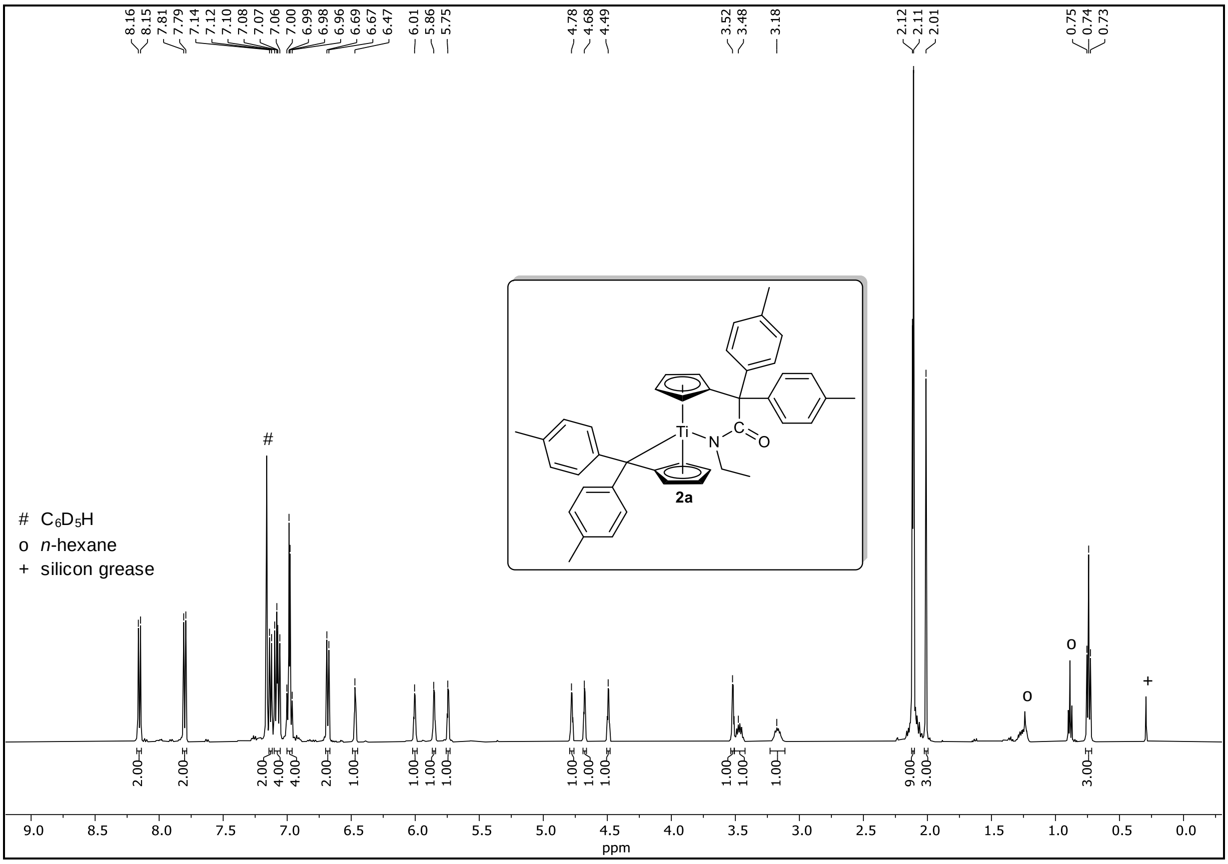

Figure S20: ^{1}H NMR spectrum of **2a** (500 MHz, C_6D_6, 305 K).

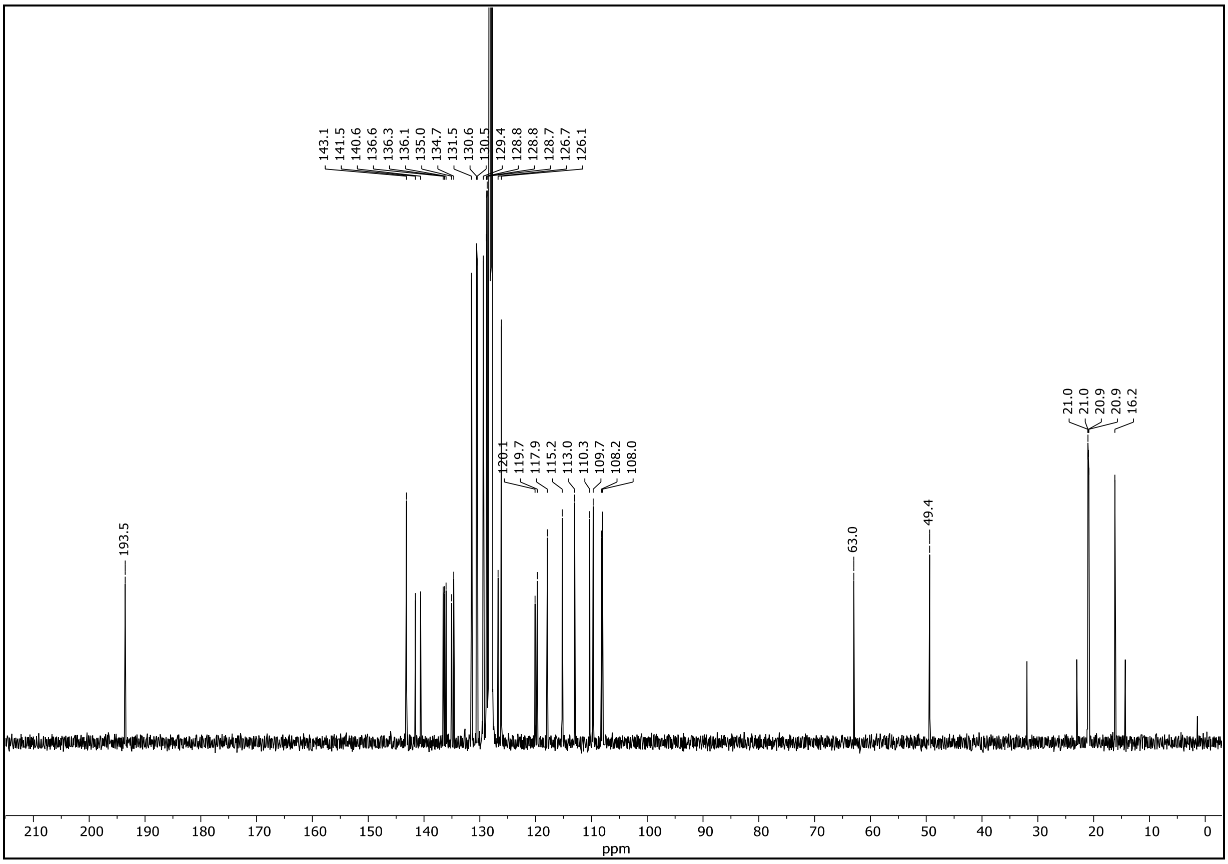

Figure S21: $^{13}C\{^{1}H\}$ NMR spectrum of **2a** (126 MHz, C_6D_6, 305 K).

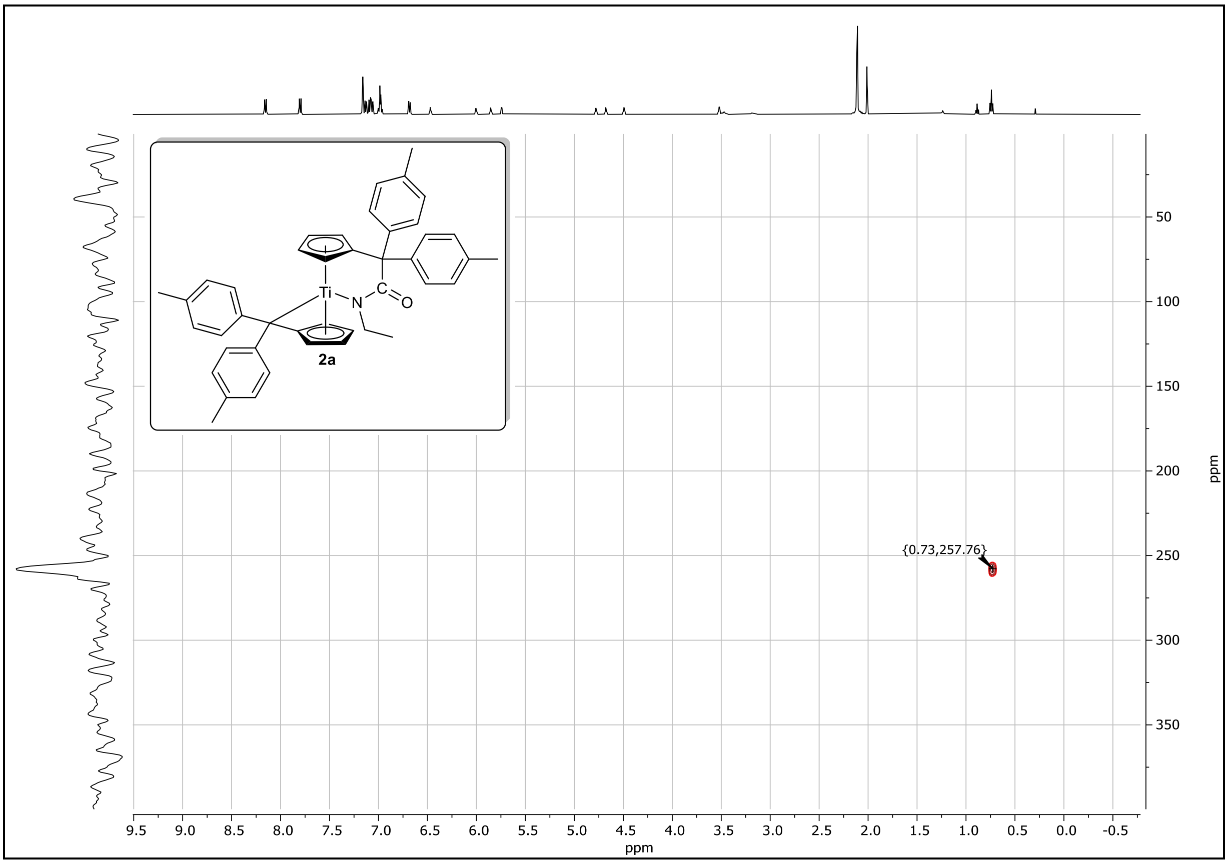

Figure S22: $^{1}H,^{15}N$ HMBC NMR spectrum of **2a** (500 MHz, 51 MHz, C_6D_6, 305 K).

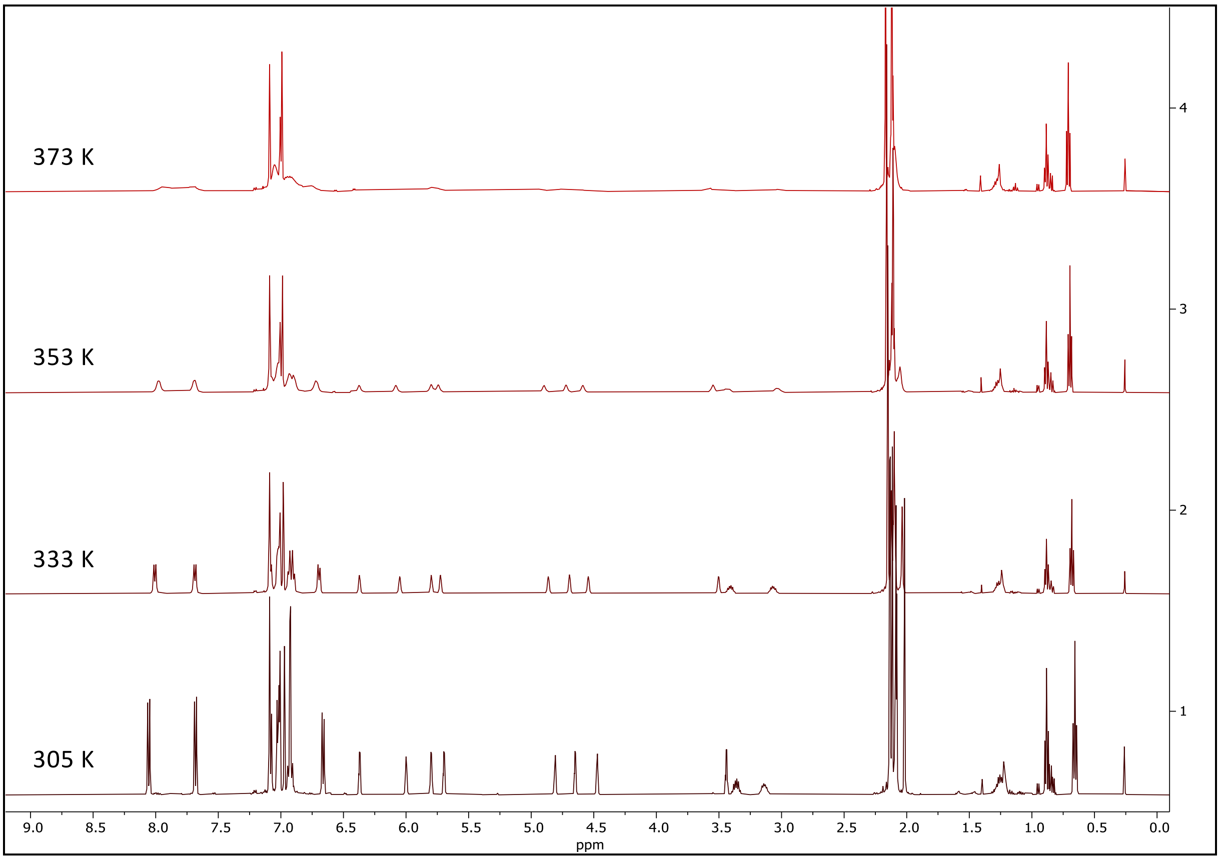

Figure S23: Variable temperature ^{1}H NMR spectrum of **2a** (500 MHz, toluene-d_8, 305 K - 373 K).

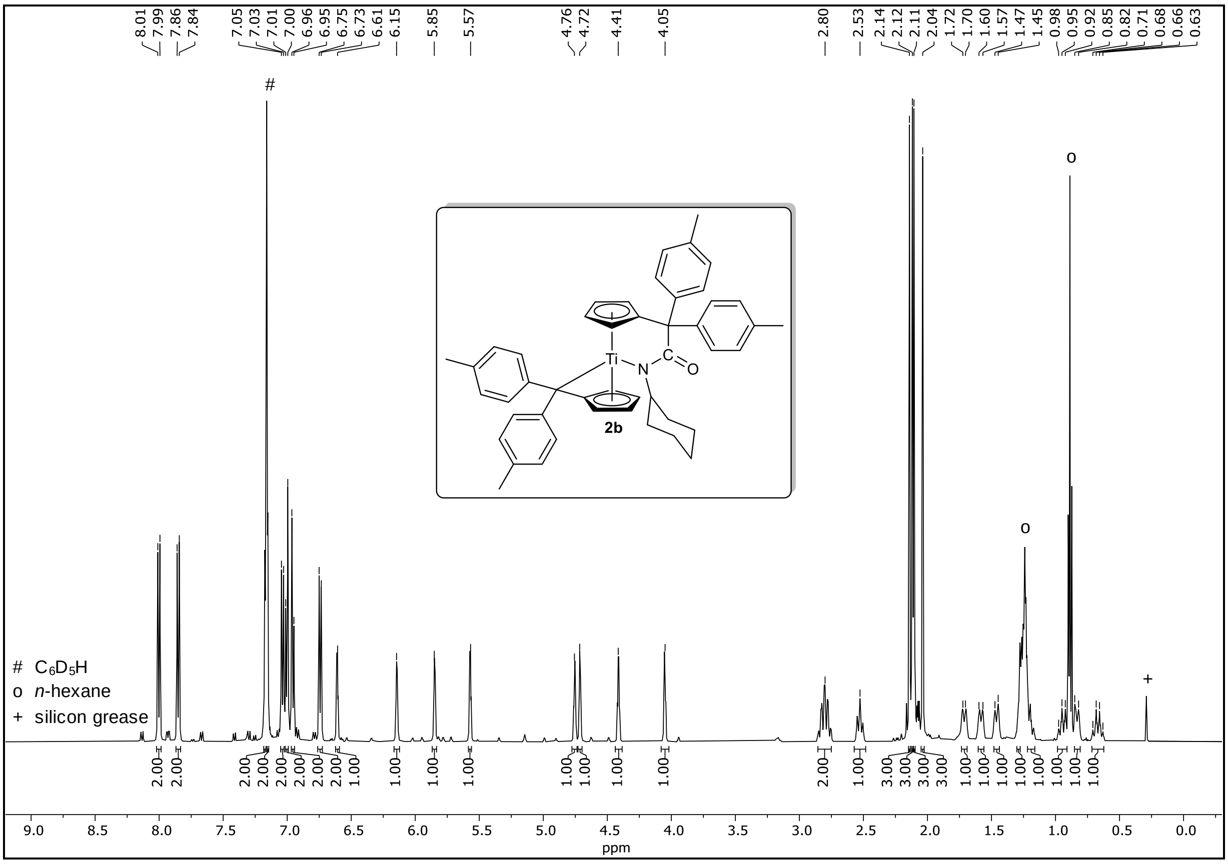

Figure S24: ^{1}H NMR spectrum of **2b** (500 MHz, C_6D_6, 305 K).

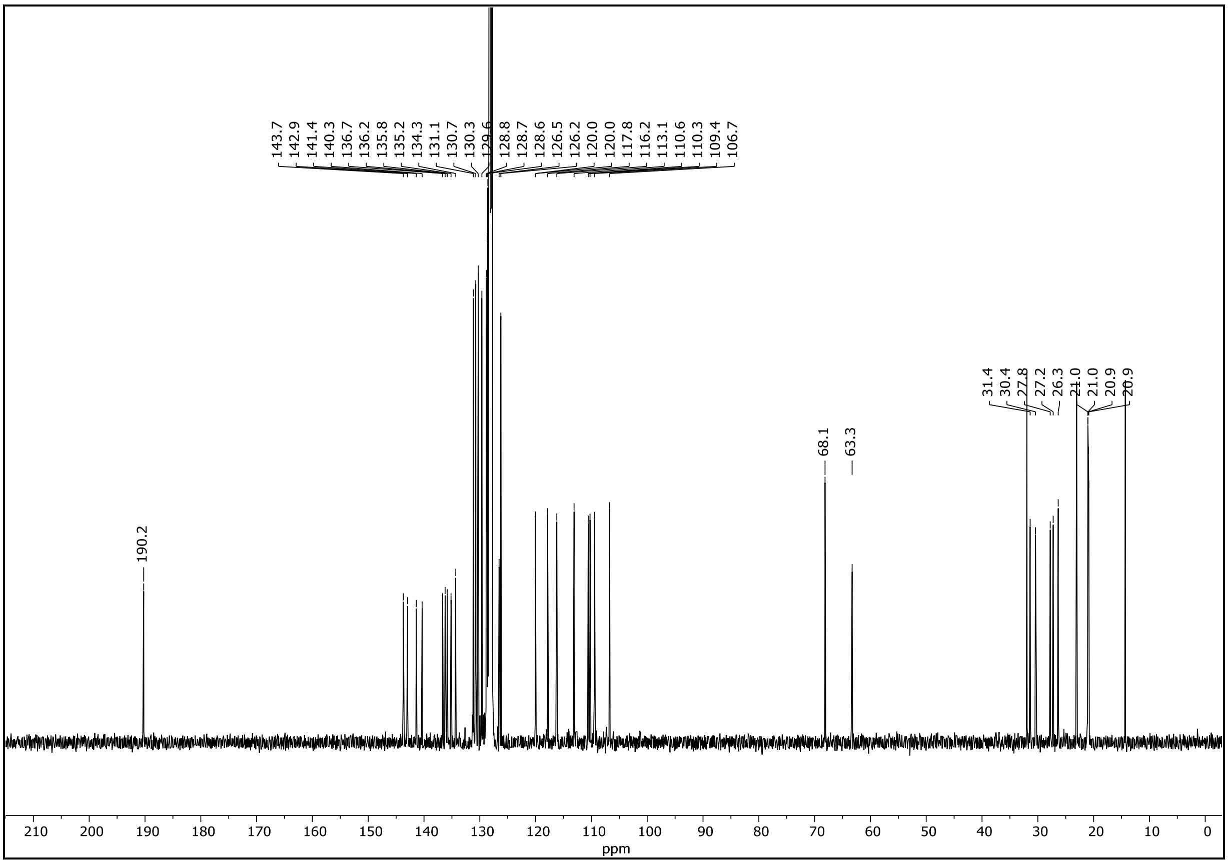

Figure S25: $^{13}C\{^{1}H\}$ NMR spectrum of **2b** (126 MHz, C_6D_6, 305 K).

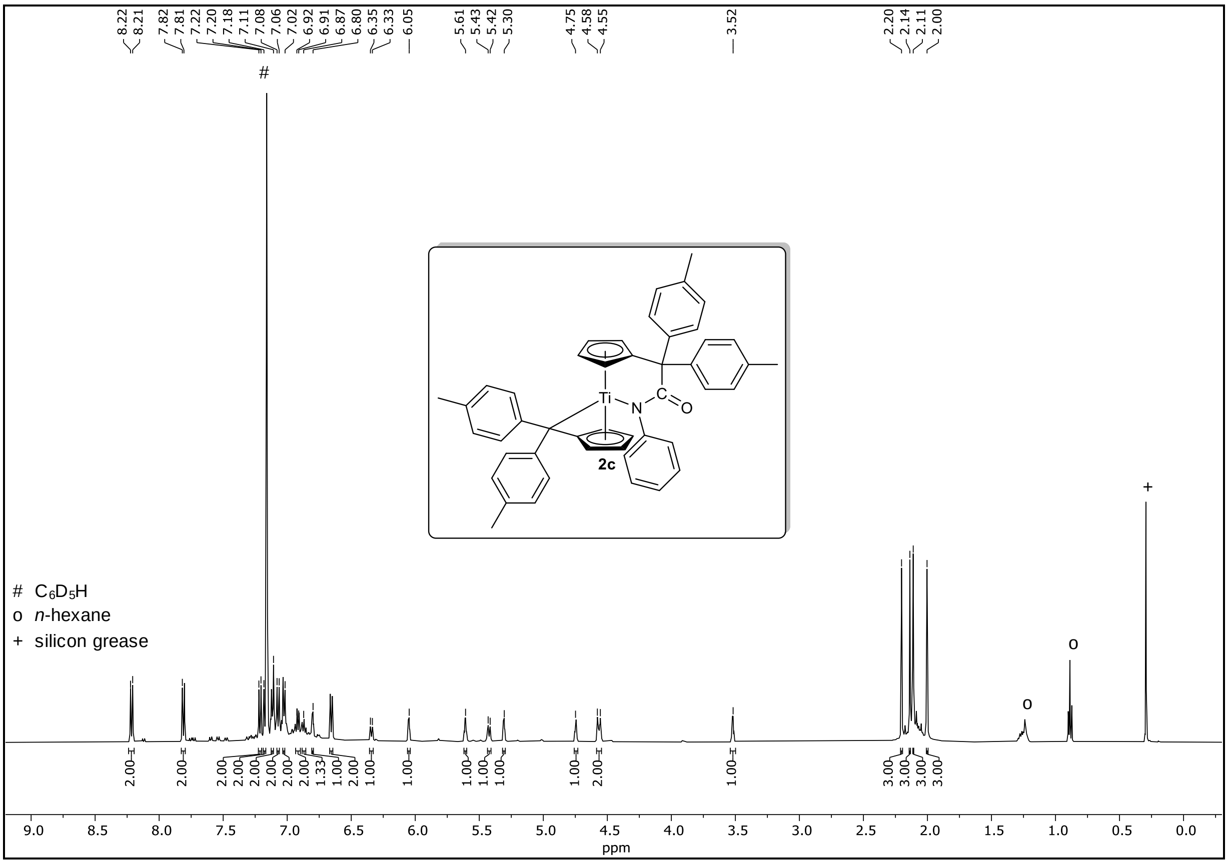

Figure S26: ^{1}H NMR spectrum of **2c** (500 MHz, C_6D_6, 305 K).

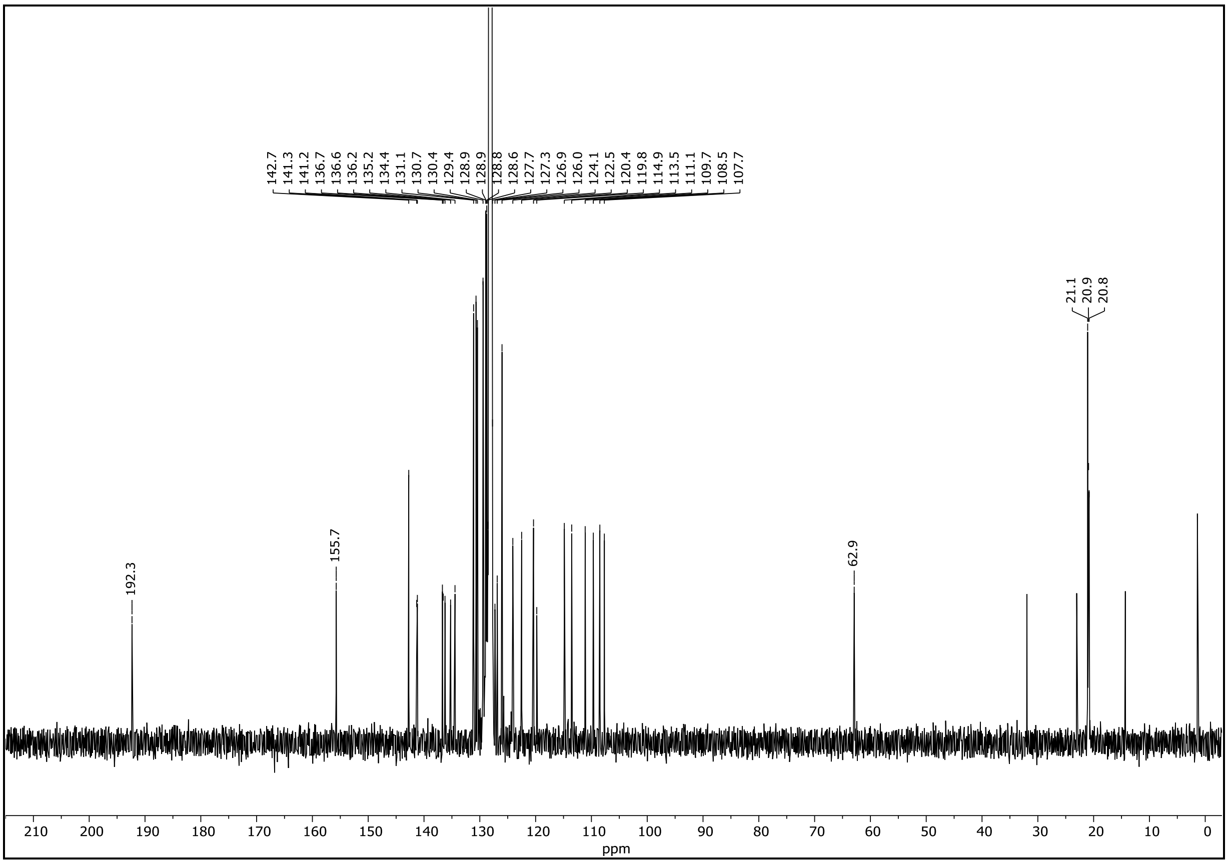

Figure S27: $^{13}C\{^{1}H\}$ NMR spectrum of **2c** (126 MHz, C_6D_6, 305 K).

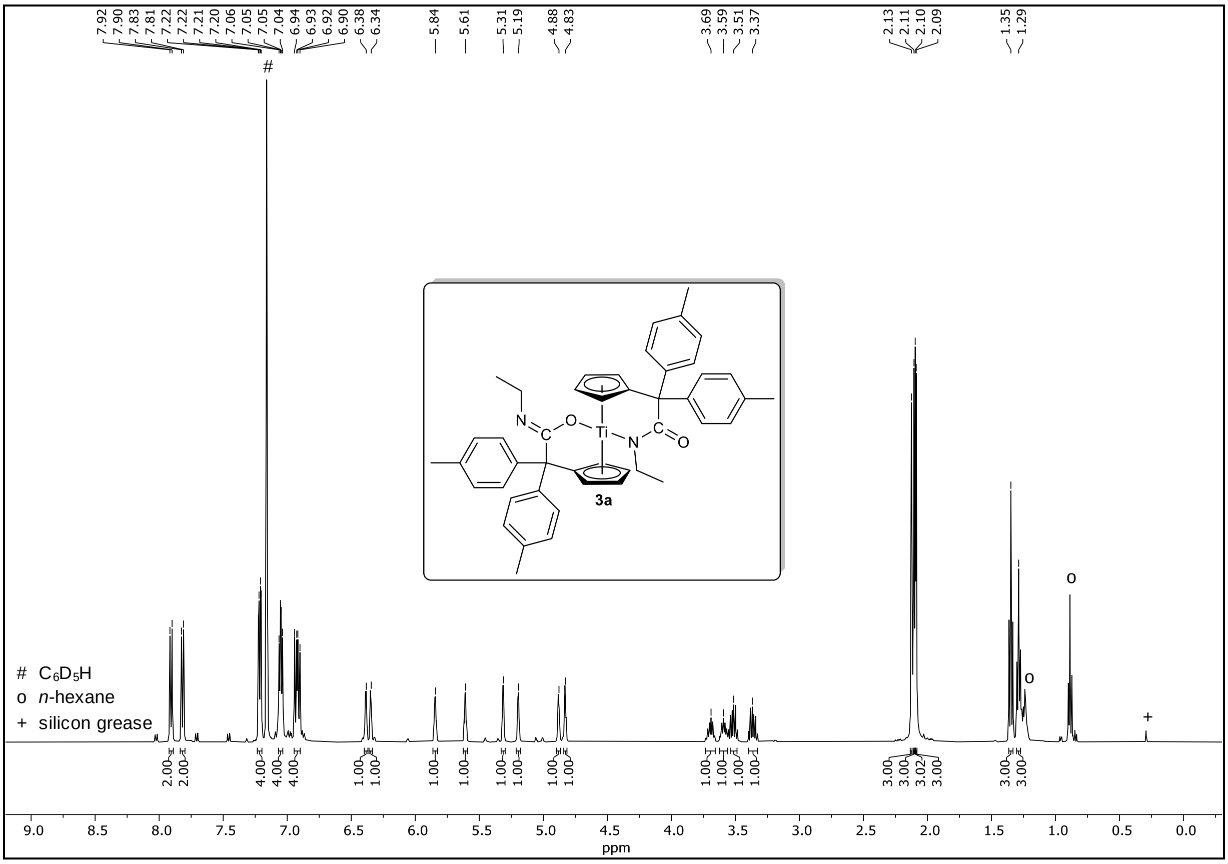

Figure S28: ^{1}H NMR spectrum of **3a** (500 MHz, C_6D_6, 305 K).

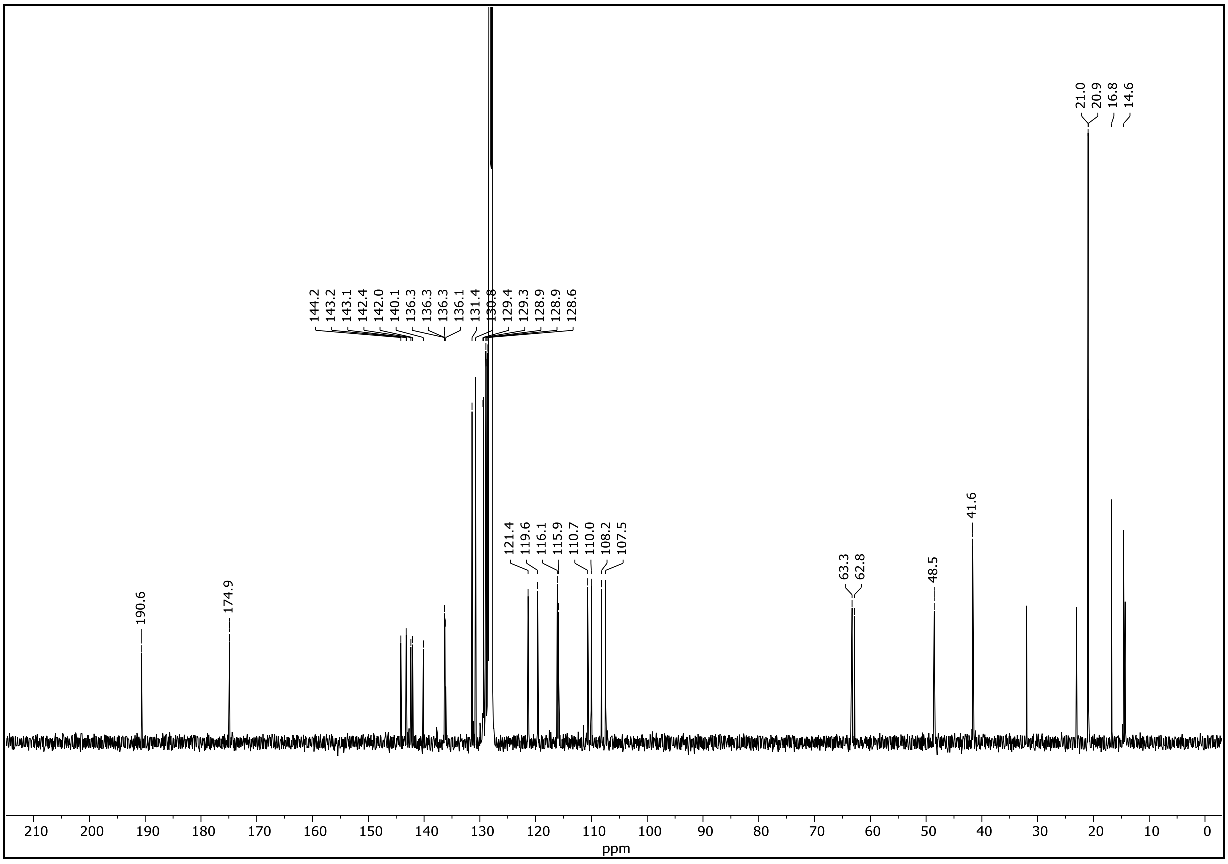

Figure S29: $^{13}C\{^{1}H\}$ NMR spectrum of **3a** (126 MHz, C_6D_6, 305 K).

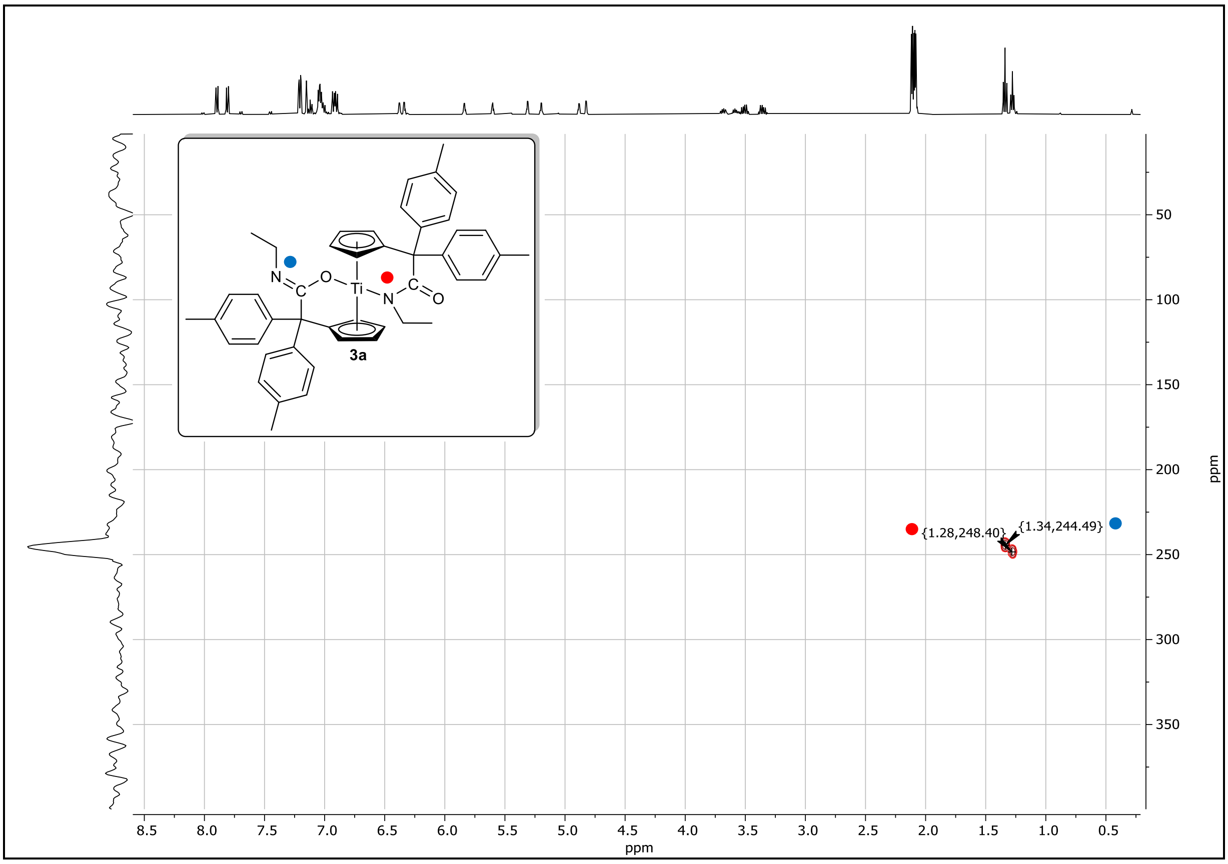

Figure S30: $^{1}H,^{15}N$ HMBC NMR spectrum of **3a** (500 MHz, 51 MHz, C_6D_6, 305 K).

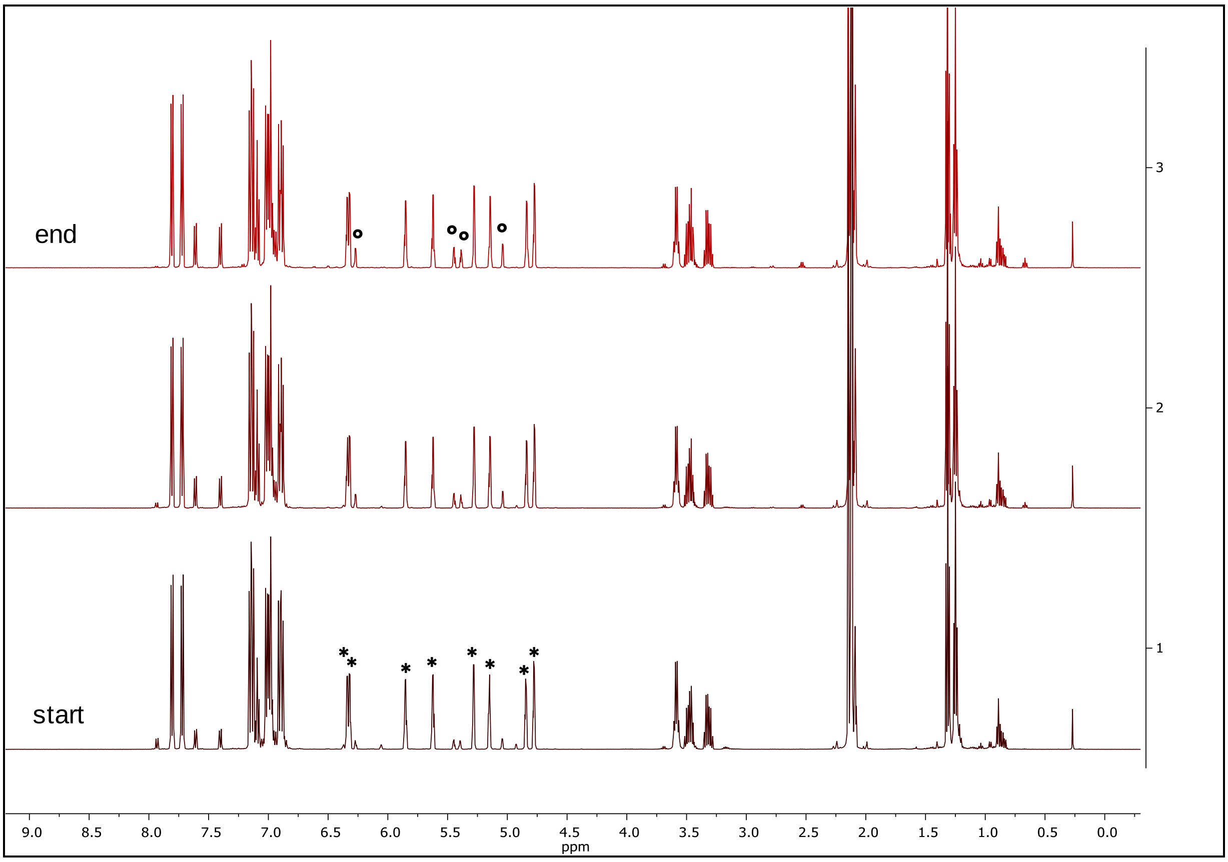

Figure S31: Stacked ^{1}H NMR spectra of **3a/3a'** (500 MHz, toluene-d_8, 305 K, **3a** = *, **3a'** = °) after heating the NMR tube at 120 °C for 4 weeks.

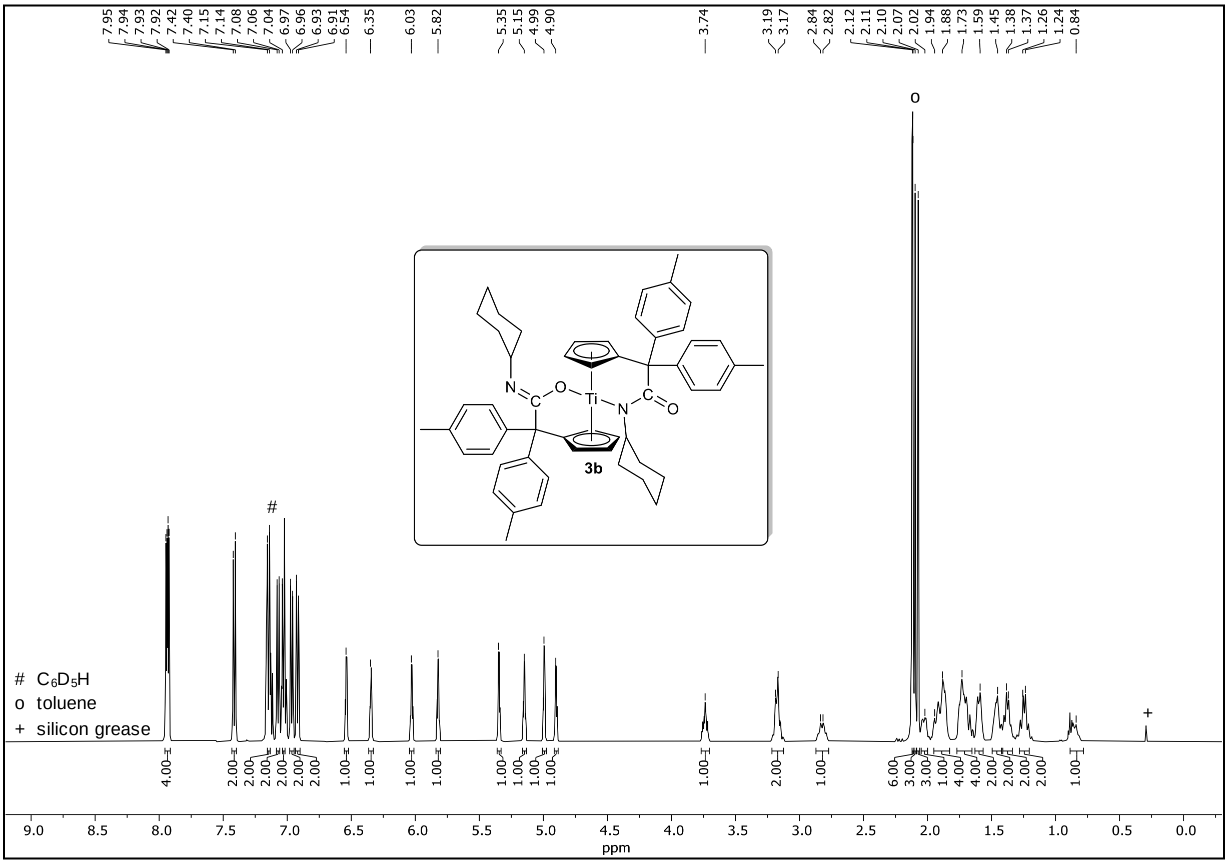

Figure S32: ^{1}H NMR spectrum of **3b** (500 MHz, C_6D_6, 305 K).

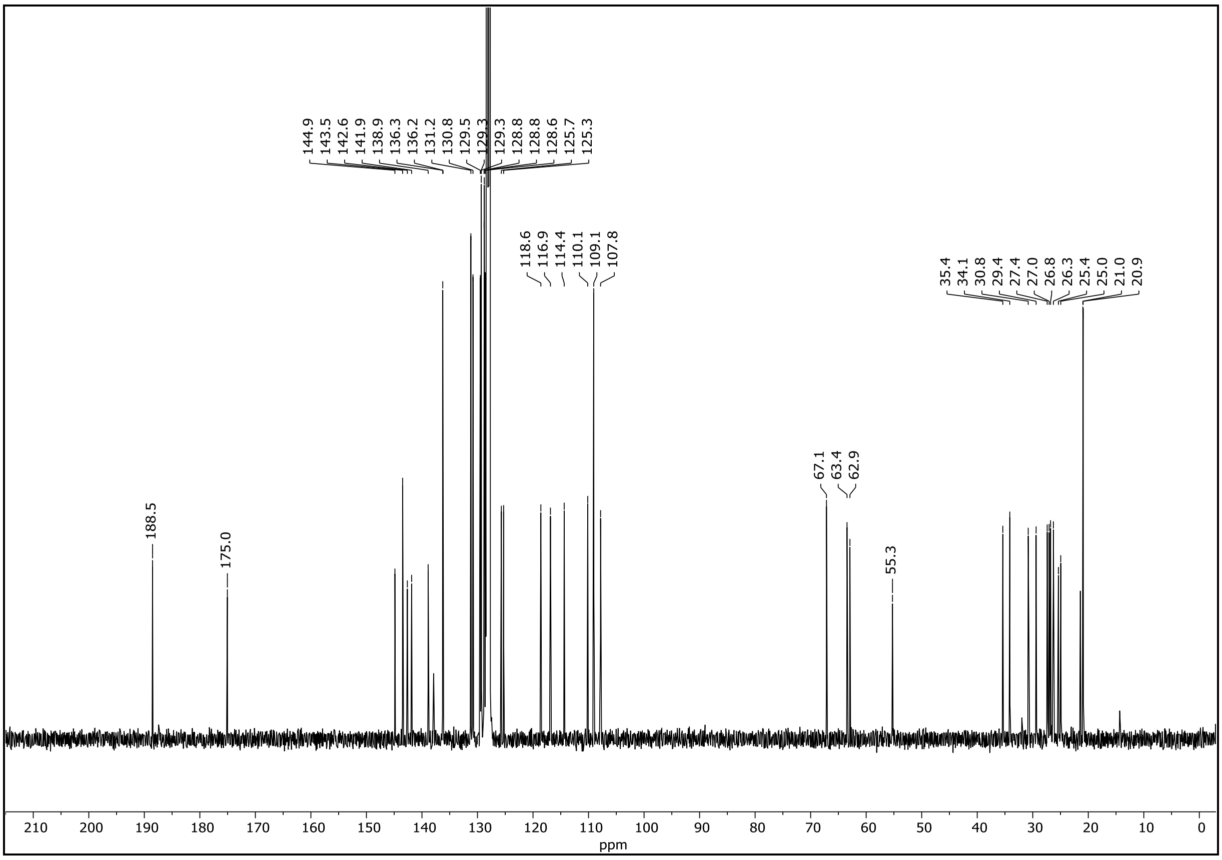

Figure S33: $^{13}C\{^{1}H\}$ NMR spectrum of **3b** (126 MHz, C_6D_6, 305 K).

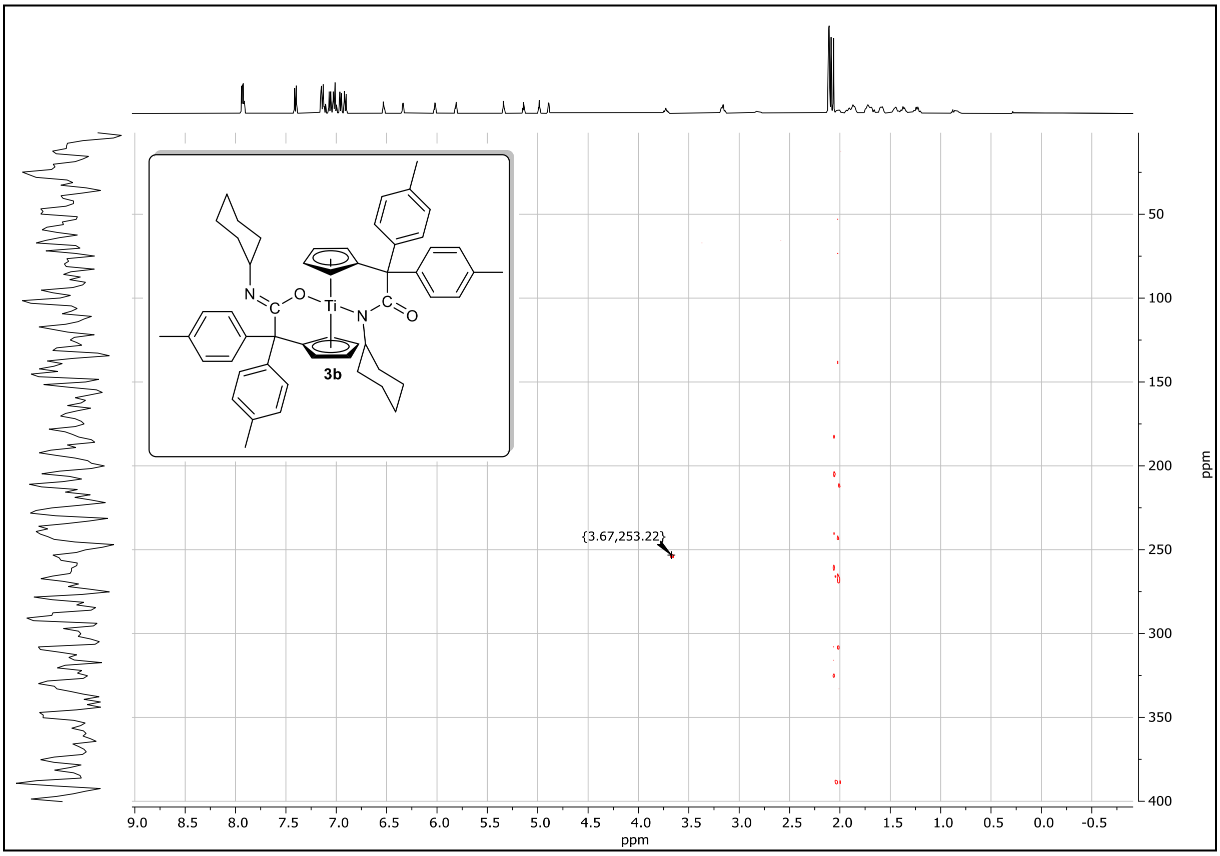

Figure S34: $^{1}H,^{15}N$ HMBC NMR spectrum of **3b** (500 MHz, 51 MHz, C_6D_6, 305 K).

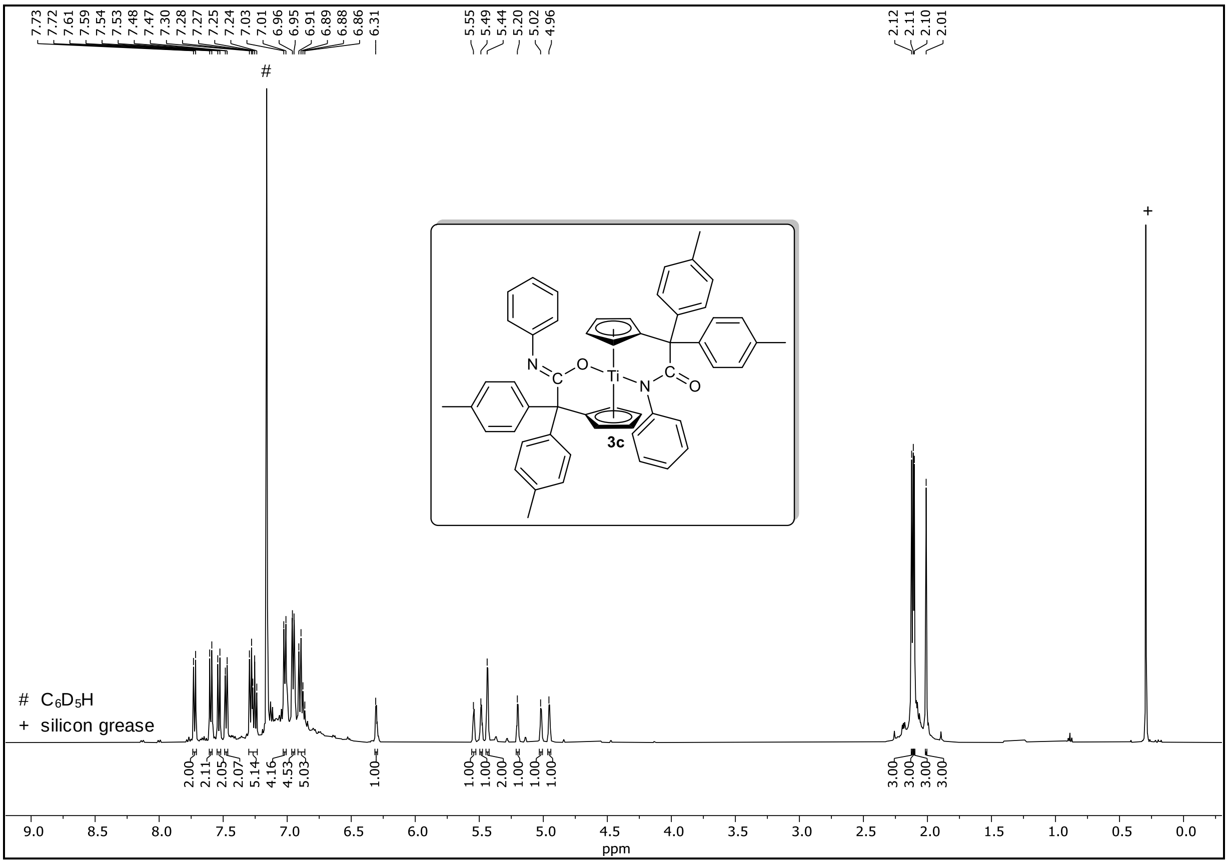

Figure S35: ^{1}H NMR spectrum of **3c** (500 MHz, C_6D_6, 305 K).

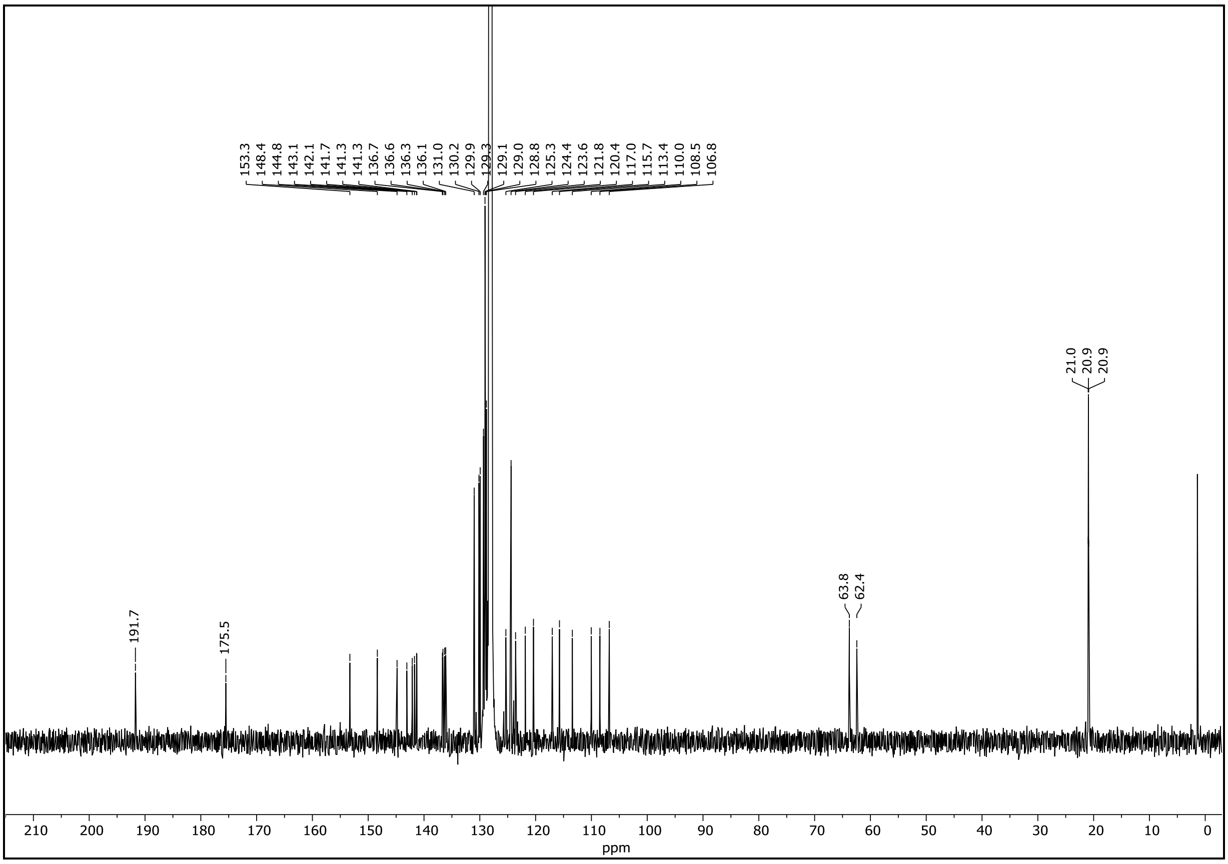

Figure S36: ^{13}C{^{1}H} NMR spectrum of **3c** (126 MHz, C_6D_6, 305 K).

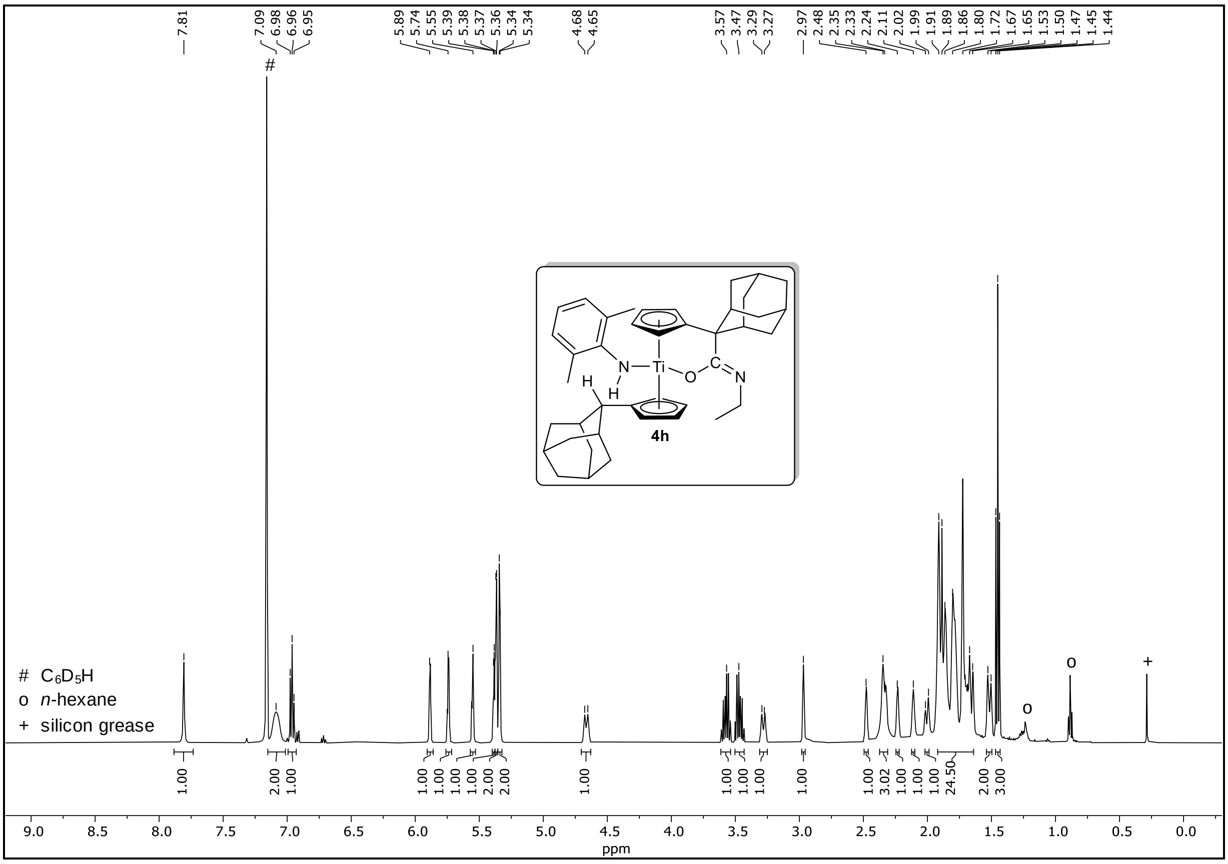

Figure S37: ^{1}H NMR spectrum of **4h** (500 MHz, C_6D_6, 305 K).

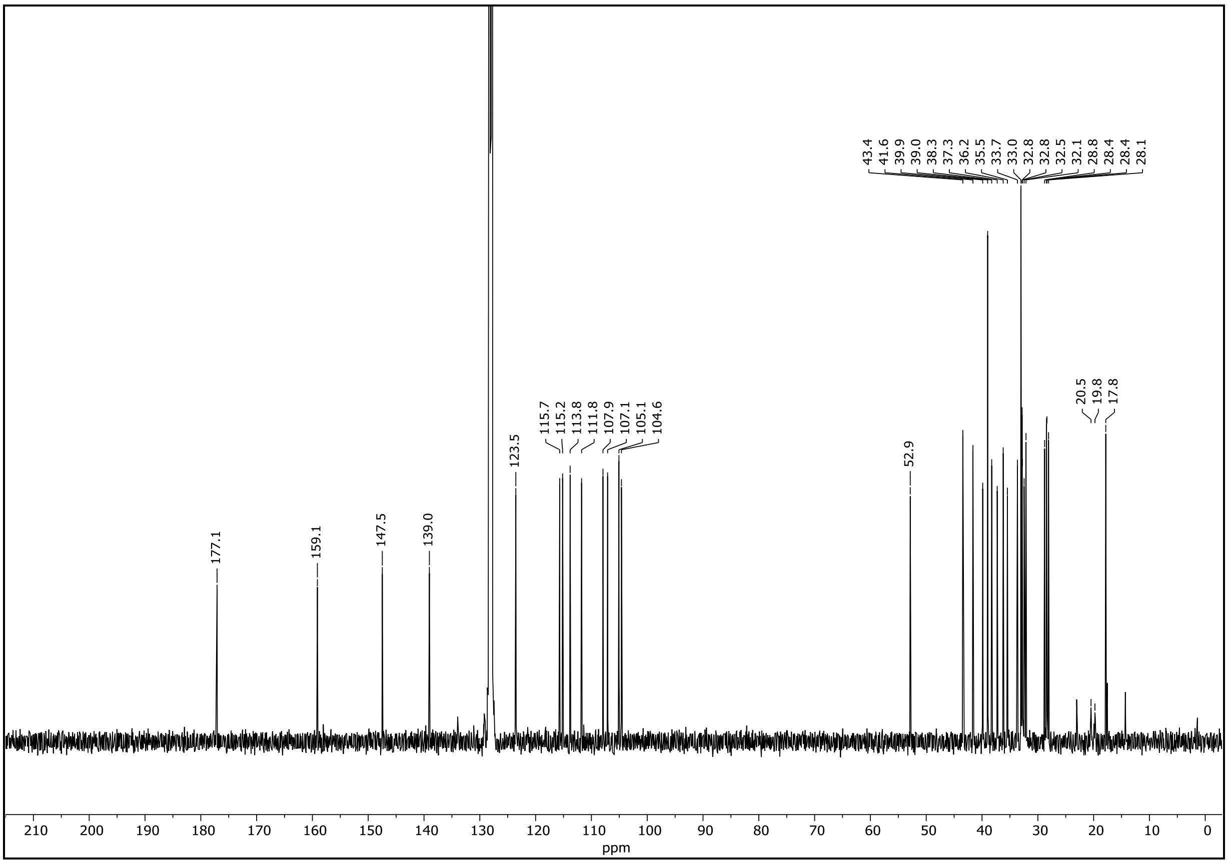

Figure S38: $^{13}C\{^1H\}$ NMR spectrum of **4h** (126 MHz, C_6D_6, 305 K).

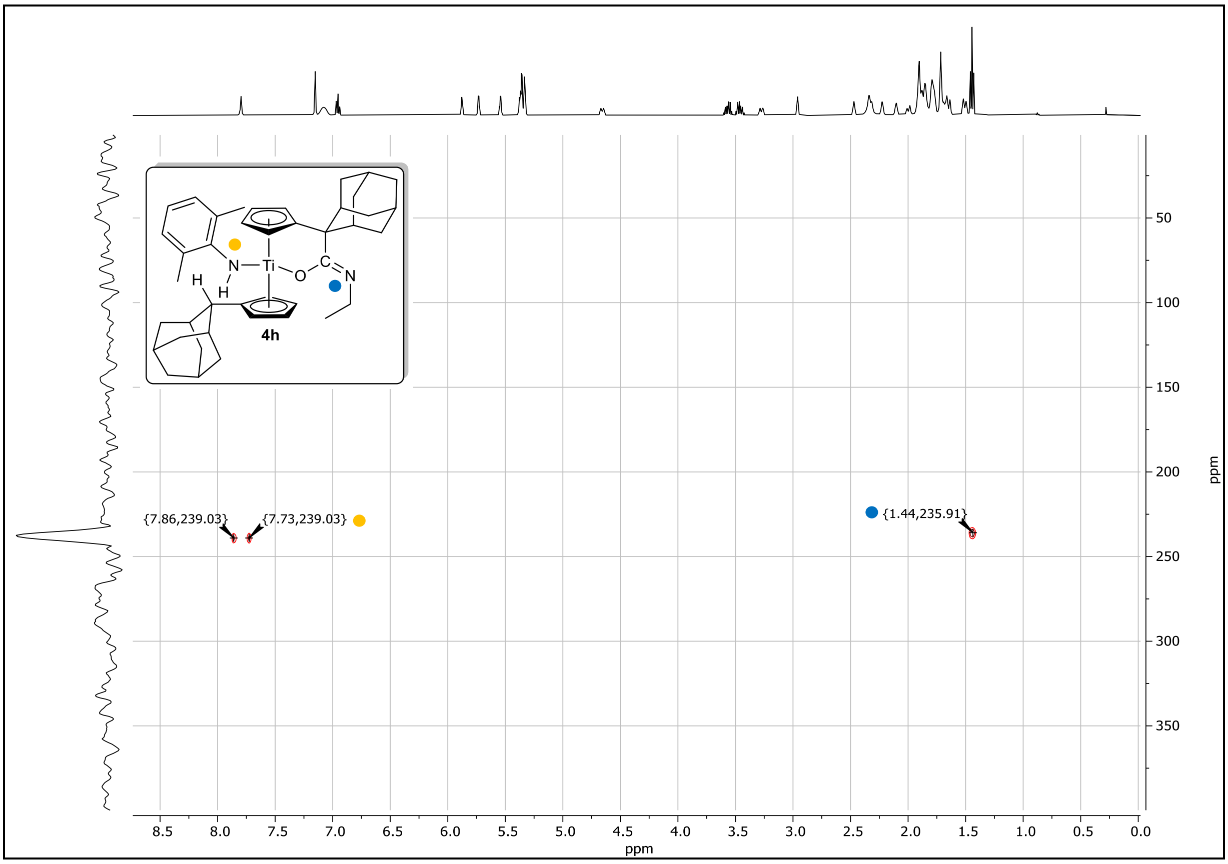

Figure S39: $^{1}H,^{15}N$ HMBC NMR spectrum of **4h** (126 MHz, C_6D_6, 305 K).

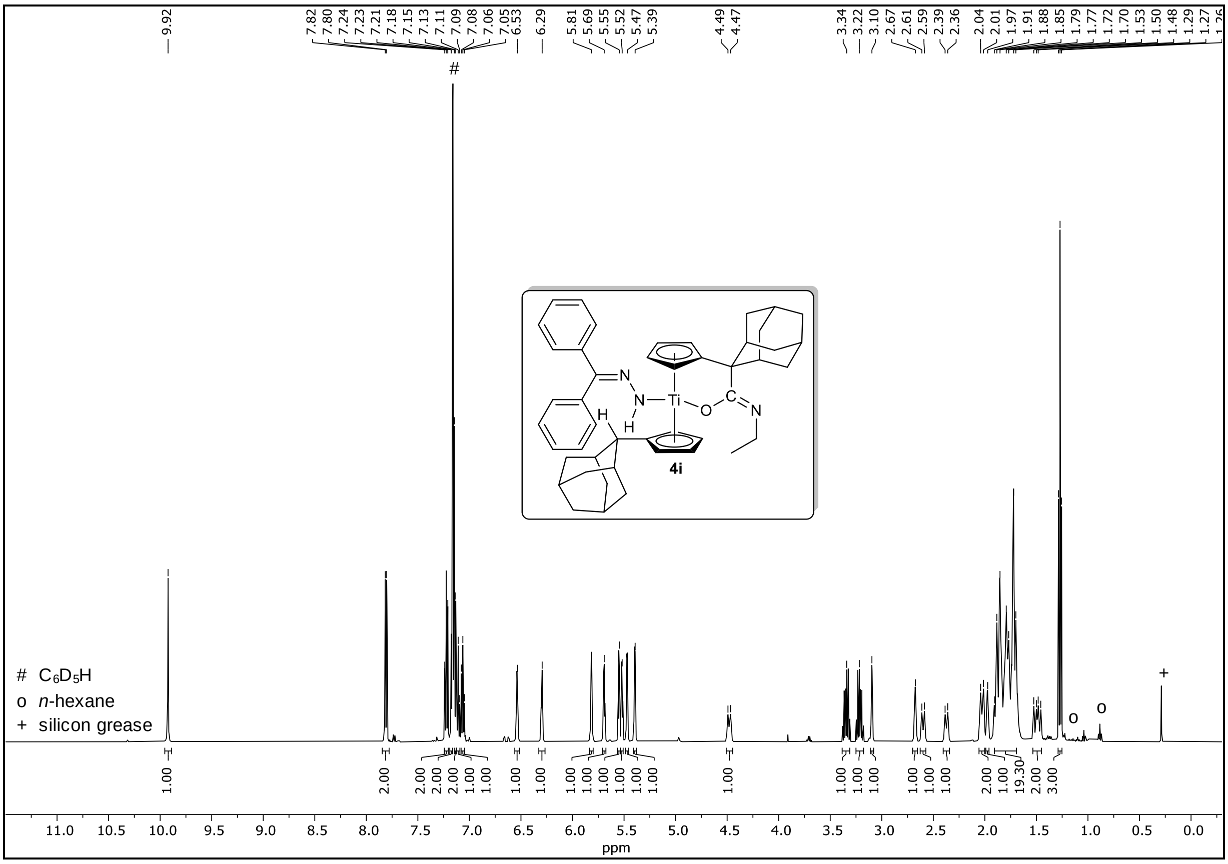

Figure S40: ^{1}H NMR spectrum of **4i** (500 MHz, C_6D_6, 305 K).

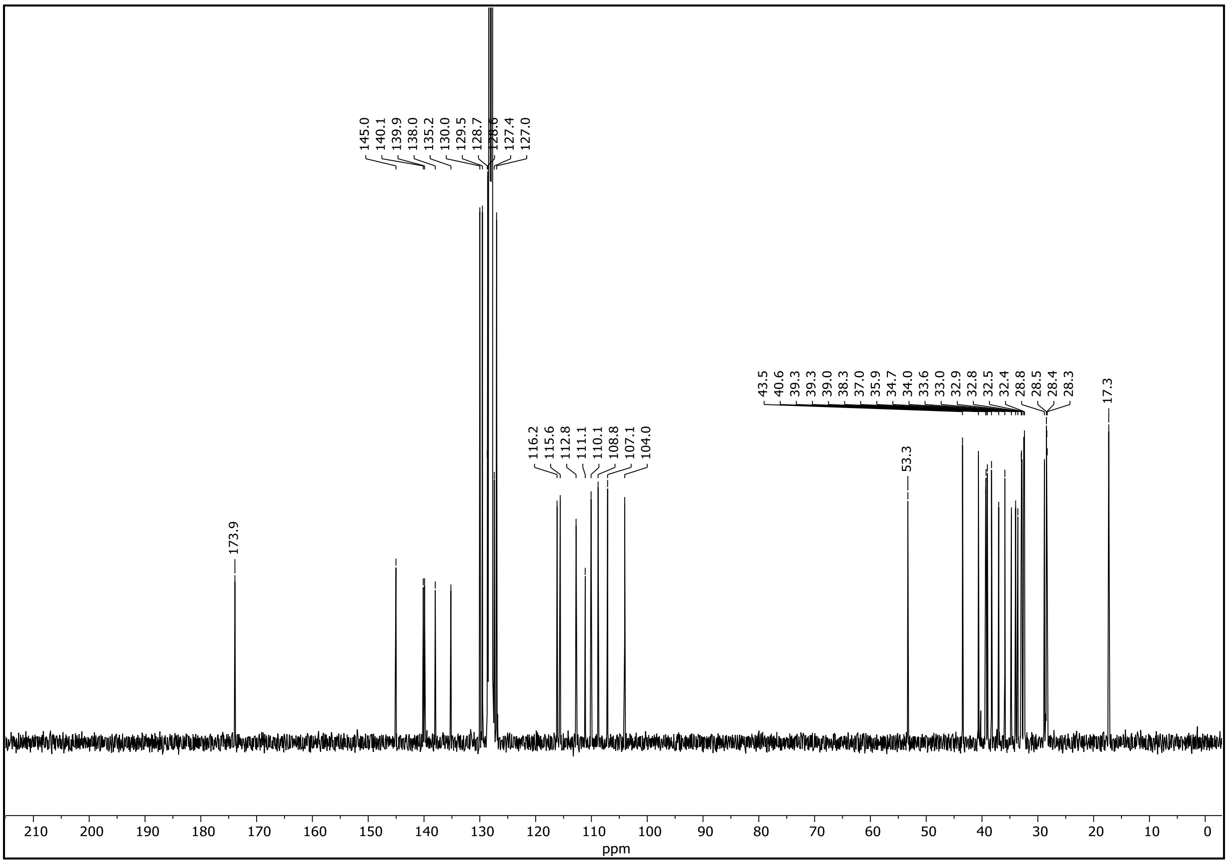

Figure S41: $^{13}C\{^{1}H\}$ NMR spectrum of **4i** (126 MHz, C_6D_6, 305 K).

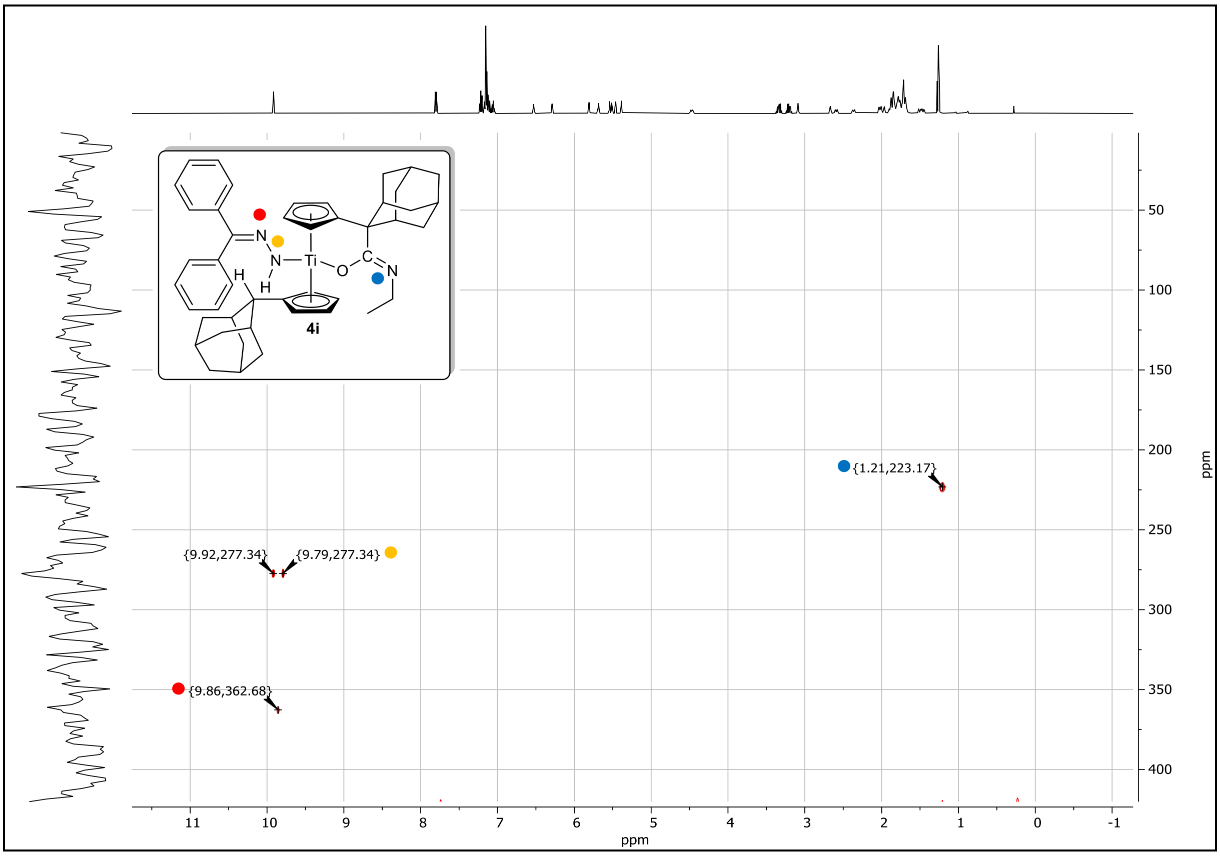

Figure S42: $^{1}H,^{15}N$ HMBC NMR spectrum of **4i** (500 MHz, 51 MHz, C_6D_6, 305 K).

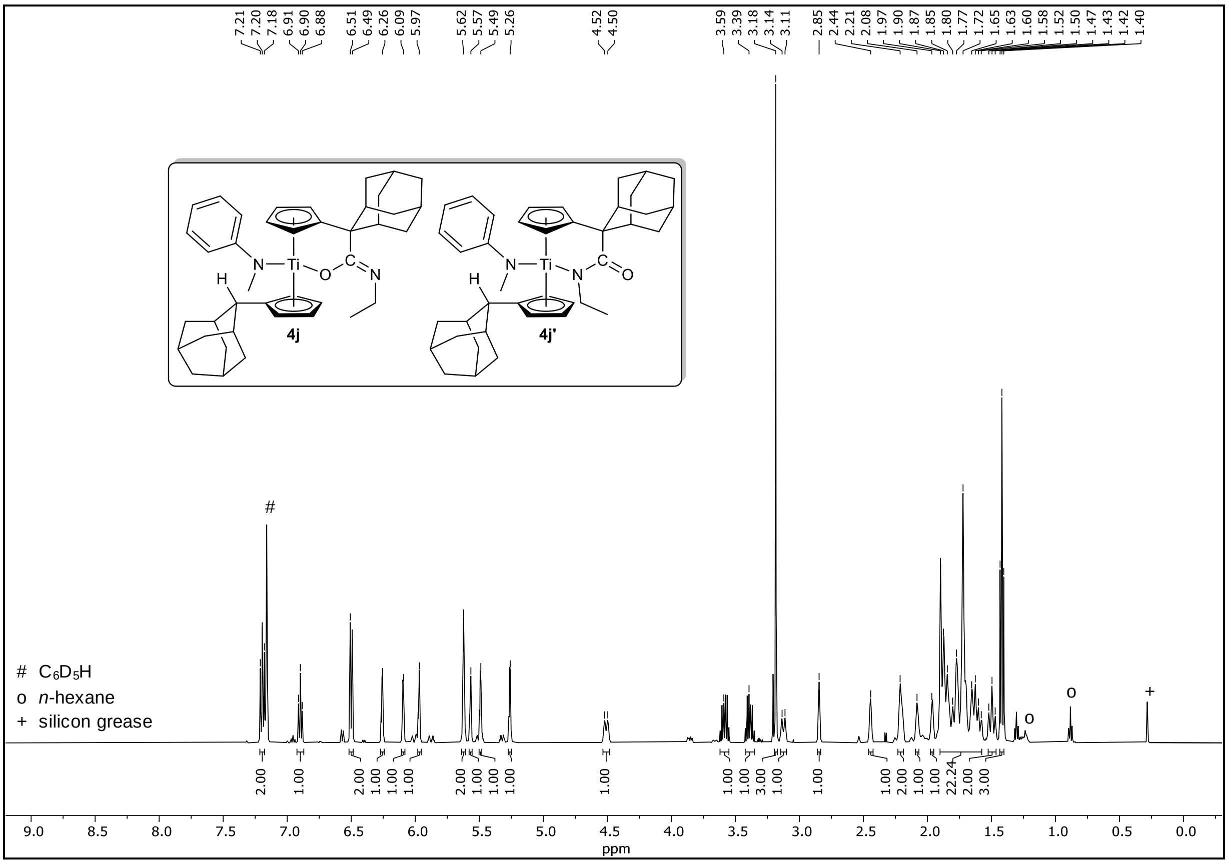

Figure S43: ^{1}H NMR spectrum of **4j/4j’** (500 MHz, C_6D_6, 305 K). Signals are marked for **4j**.

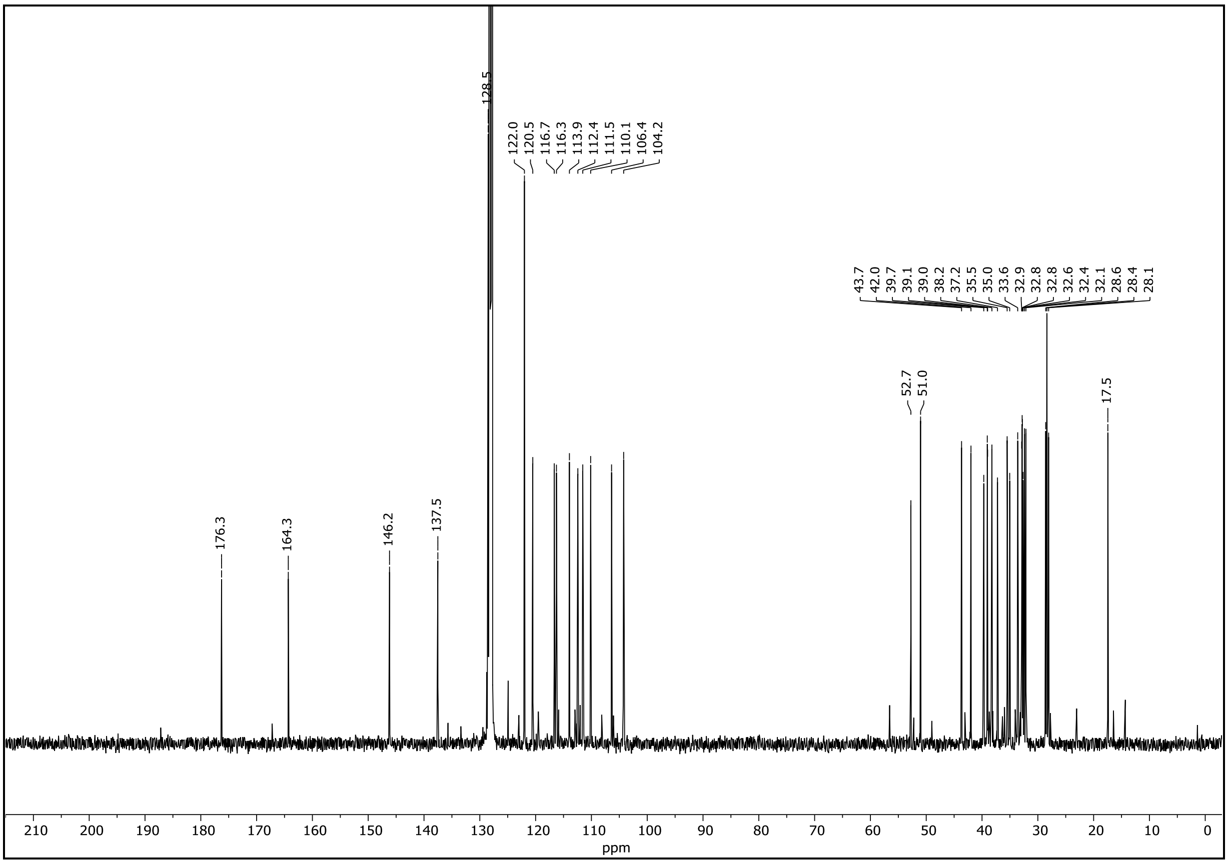

Figure S44: ^{13}C{^{1}H} NMR spectrum of **4j/4j’** (126 MHz, C_6D_6, 305 K). Signals are marked for **4j**.

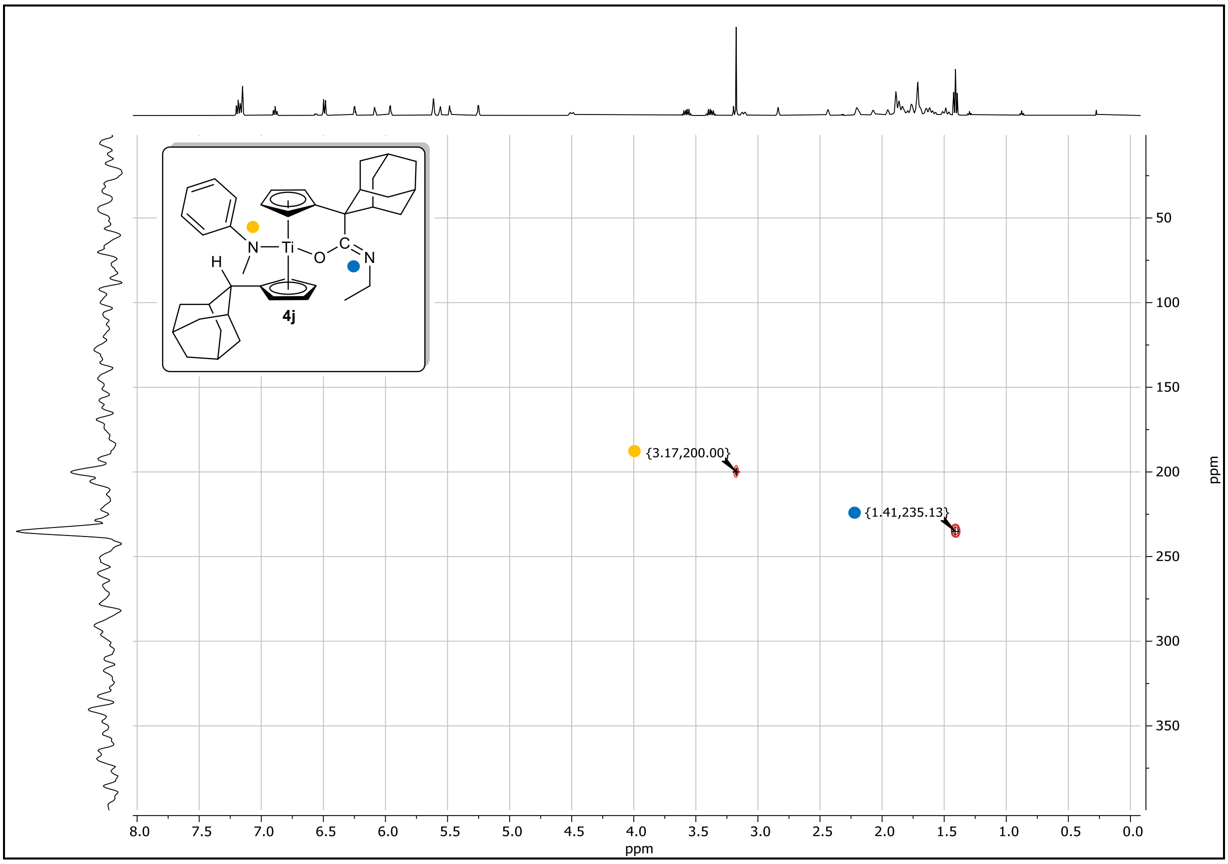

Figure S45: $^{1}H,^{15}N$ HMBC NMR spectrum of **4j/4j'** (500 MHz, 51 MHz, C_6D_6, 305 K). Signals are marked for **4j**.

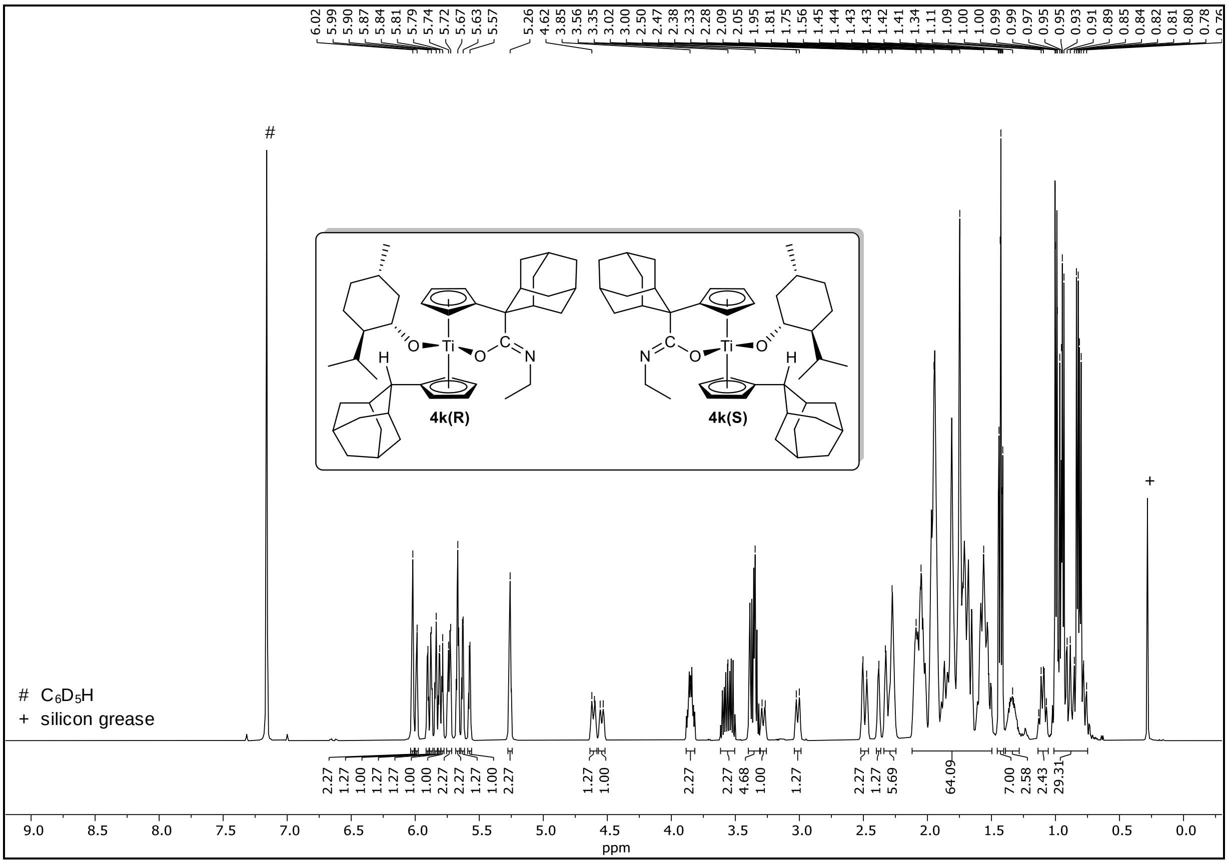

Figure S46: ^{1}H NMR spectrum of **4k(R)/4k(S)** (500 MHz, C_6D_6, 305 K). Signals are marked for **4k(R)** and **4k(S)**.

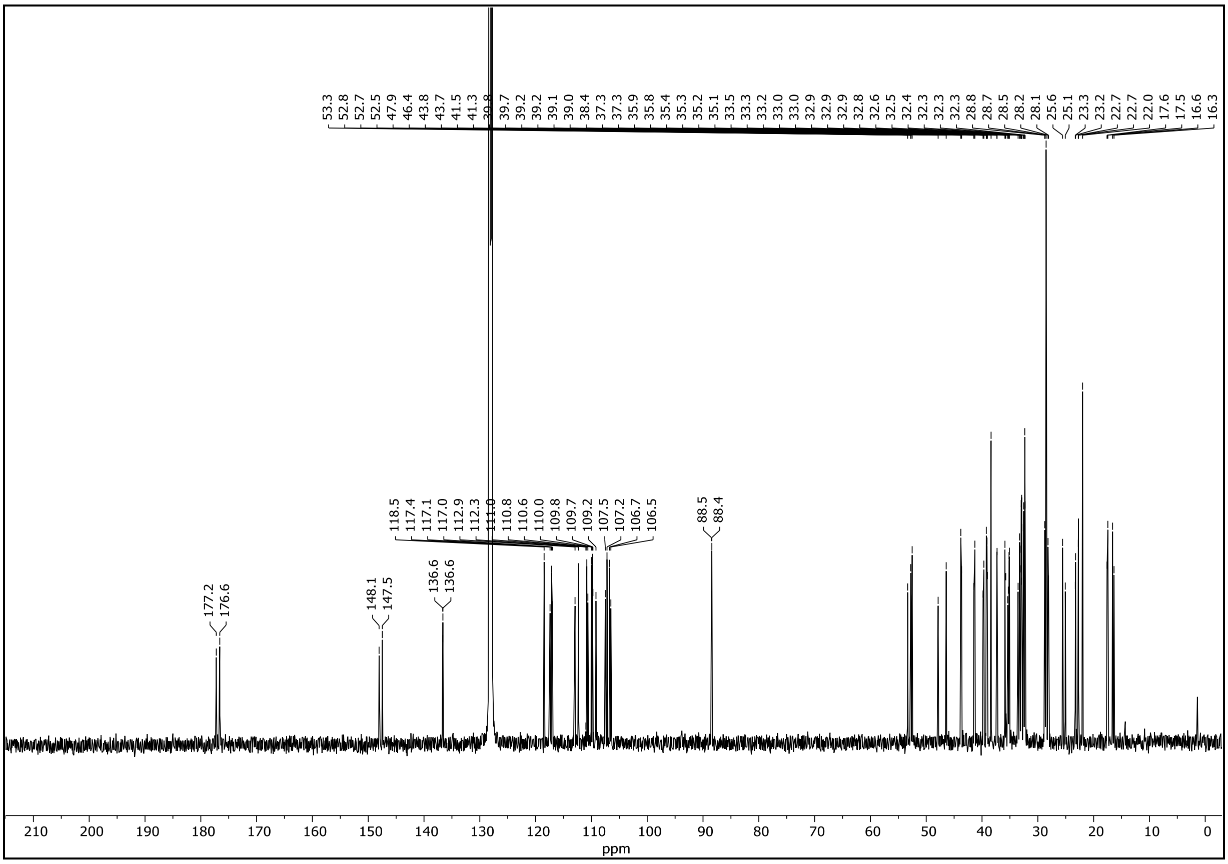

Figure S47: $^{13}C\{^{1}H\}$ NMR spectrum of **4k(R)/4k(S)** (126 MHz, C_6D_6, 305 K). Signals are marked for **4k(R)** and **4k(S)**.

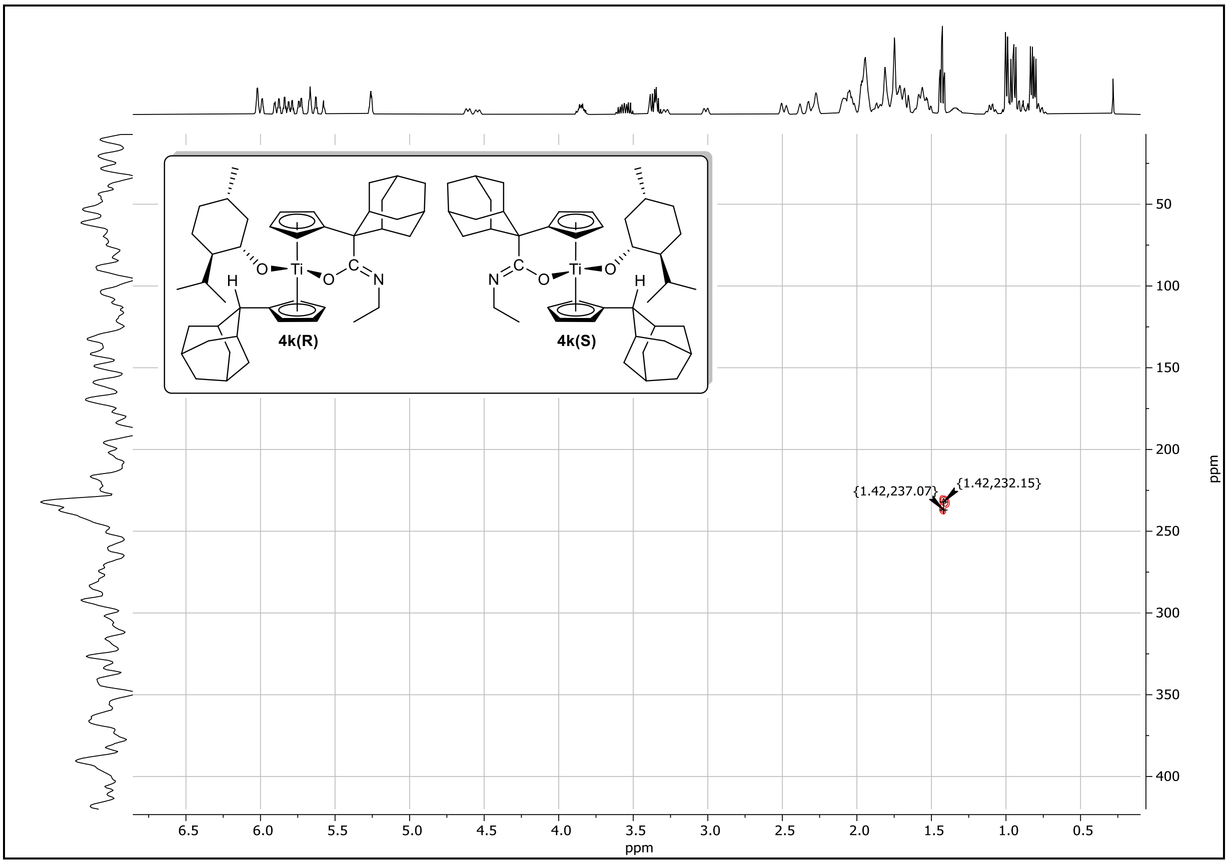

Figure S48: $^{1}H,^{15}N$ HMBC NMR spectrum of **4k(R)/4k(S)** (500 MHz, 51 MHz, C_6D_6, 305 K). Signals are marked for **4k(R)** and **4k(S)**.

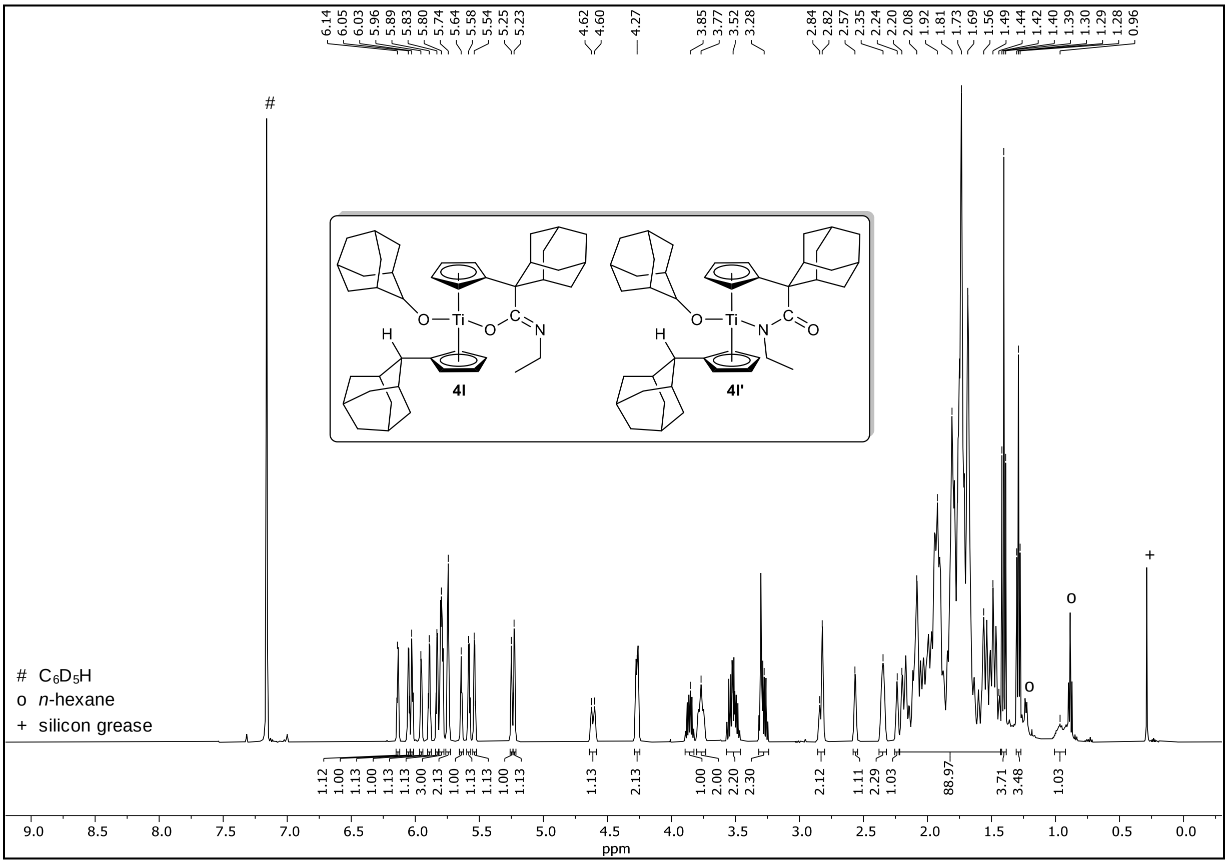

Figure S49: ^{1}H NMR spectrum of **4l/4l’** (500 MHz, C_6D_6, 305 K). Signals are marked for **4l** and **4l’**.

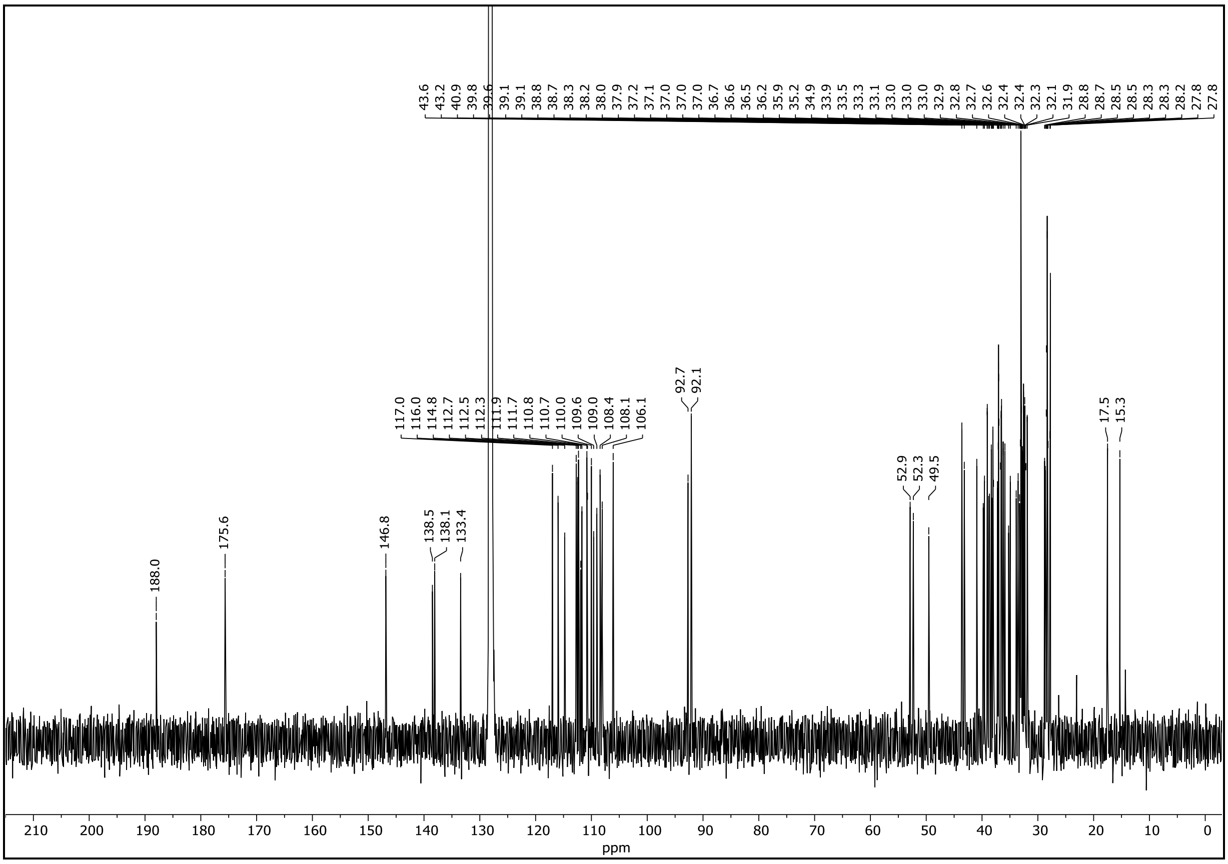

Figure S50: $^{13}C\{^{1}H\}$ NMR spectrum of **4l/4l’** (126 MHz, C_6D_6, 305 K). Signals are marked for **4l** and **4l’**.

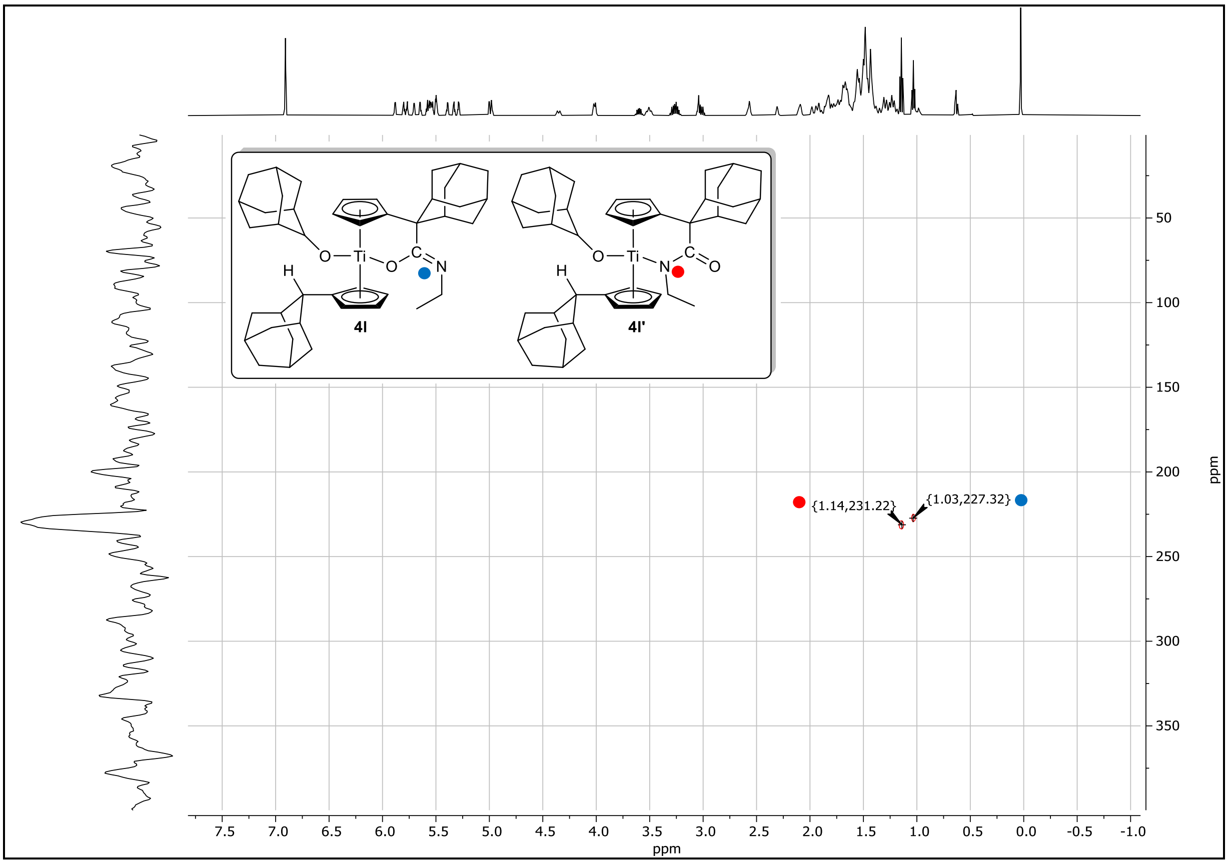

Figure S51: $^{1}H,^{15}N$ HMBC NMR spectrum of **4l/4l'** (500 MHz, 51 MHz, C_6D_6, 305 K). Signals are marked for **4l** and **4l'**.

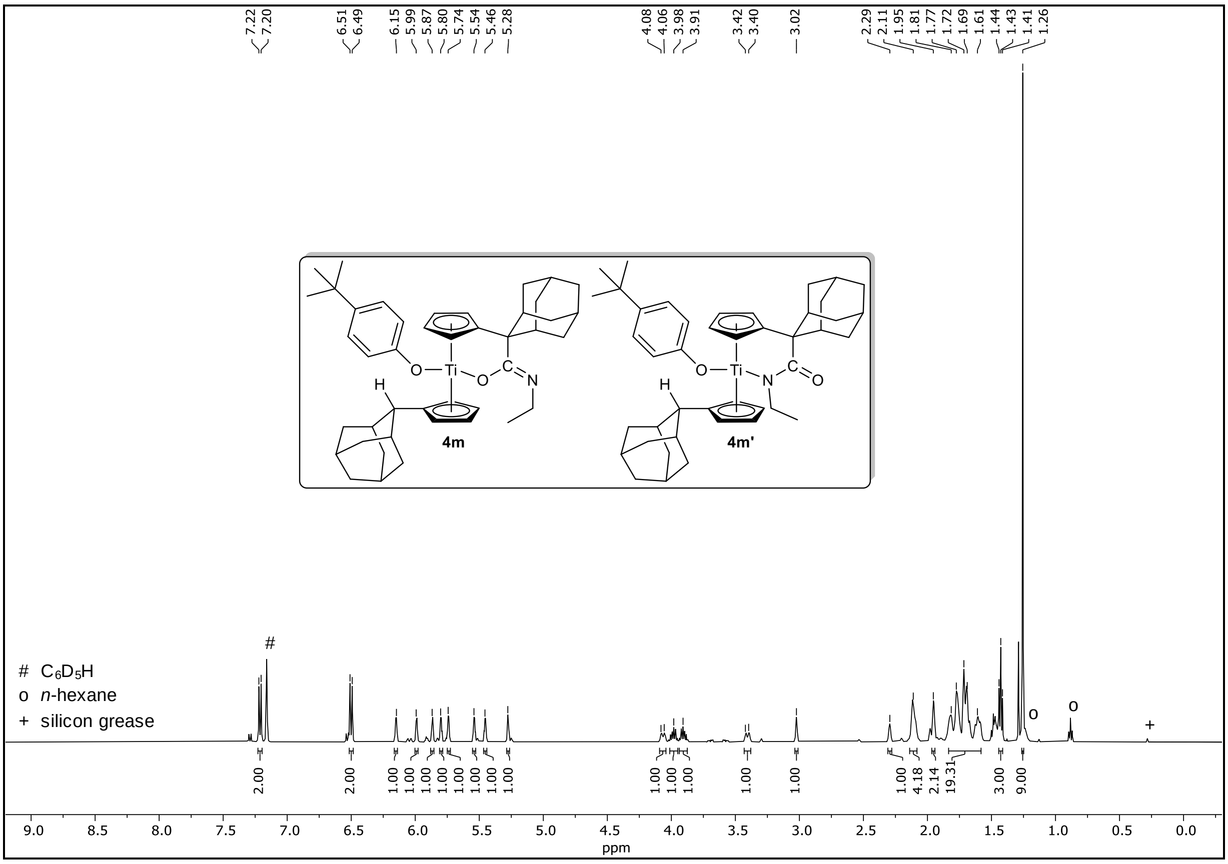

Figure S52: ^{1}H NMR spectrum of **4m/4m'** (500 MHz, C_6D_6, 305 K). Signals are marked for **4m'**.

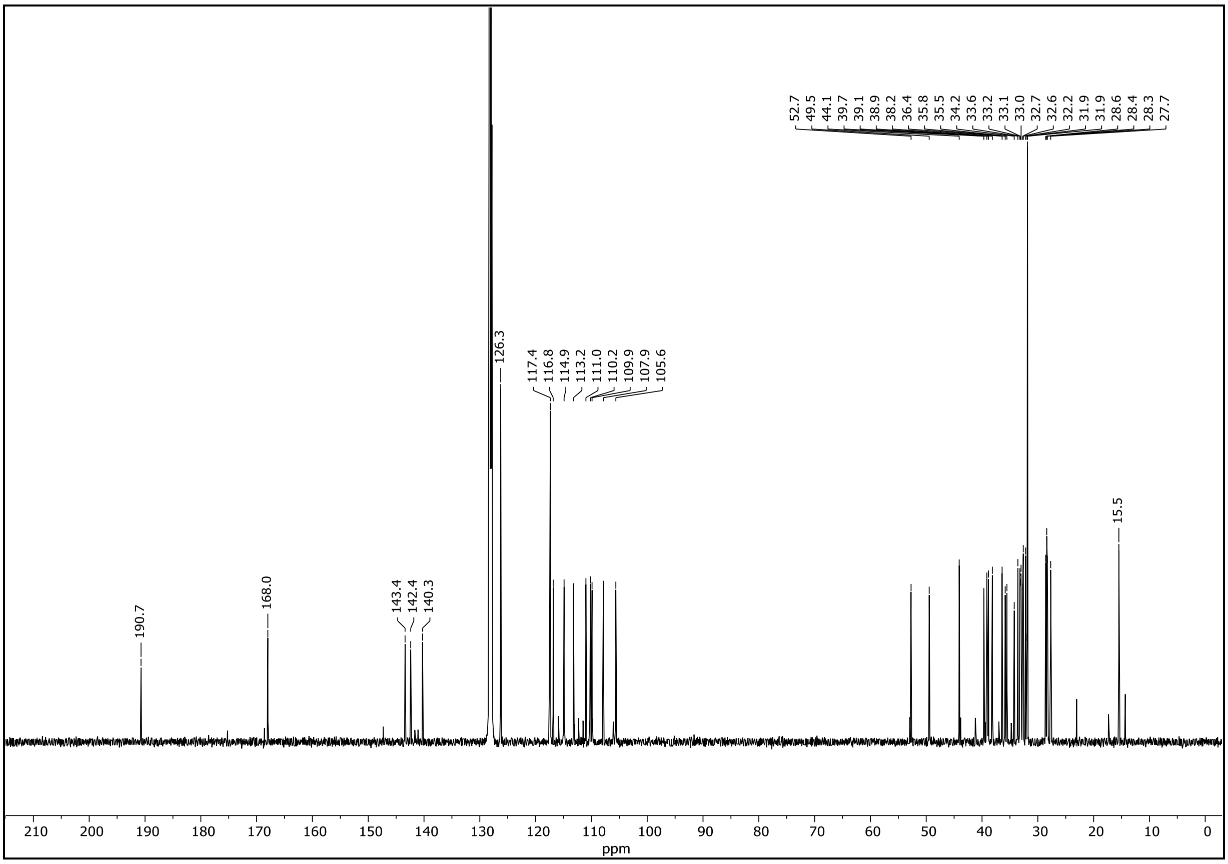

Figure S53: $^{13}C\{^{1}H\}$ NMR spectrum of **4m/4m’** (126 MHz, C_6D_6, 305 K). Signals are marked for **4m’**.

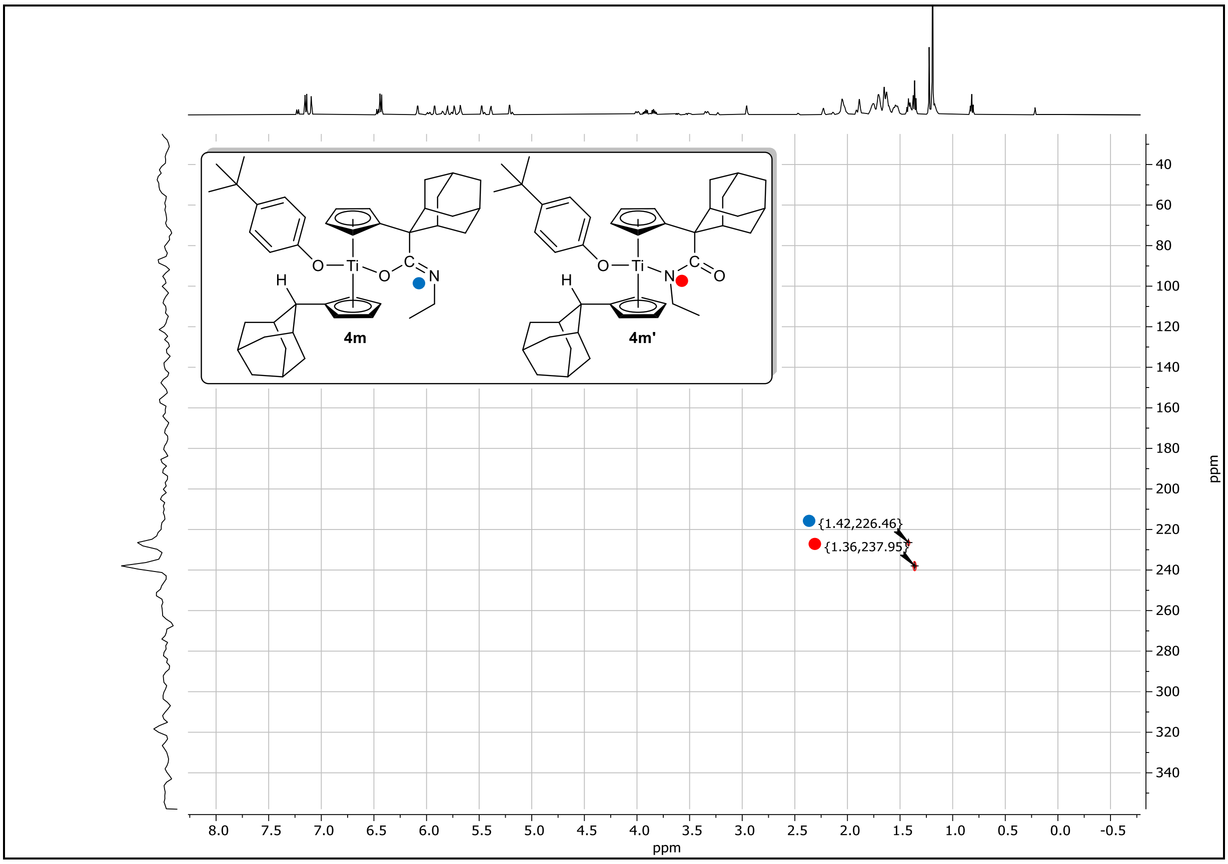

Figure S54: $^{1}H,^{15}N$ HMBC NMR spectrum of **4m/4m’** (500 MHz, 51 MHz, C_6D_6, 305 K). Signals are marked for **4m** and **4m’**.

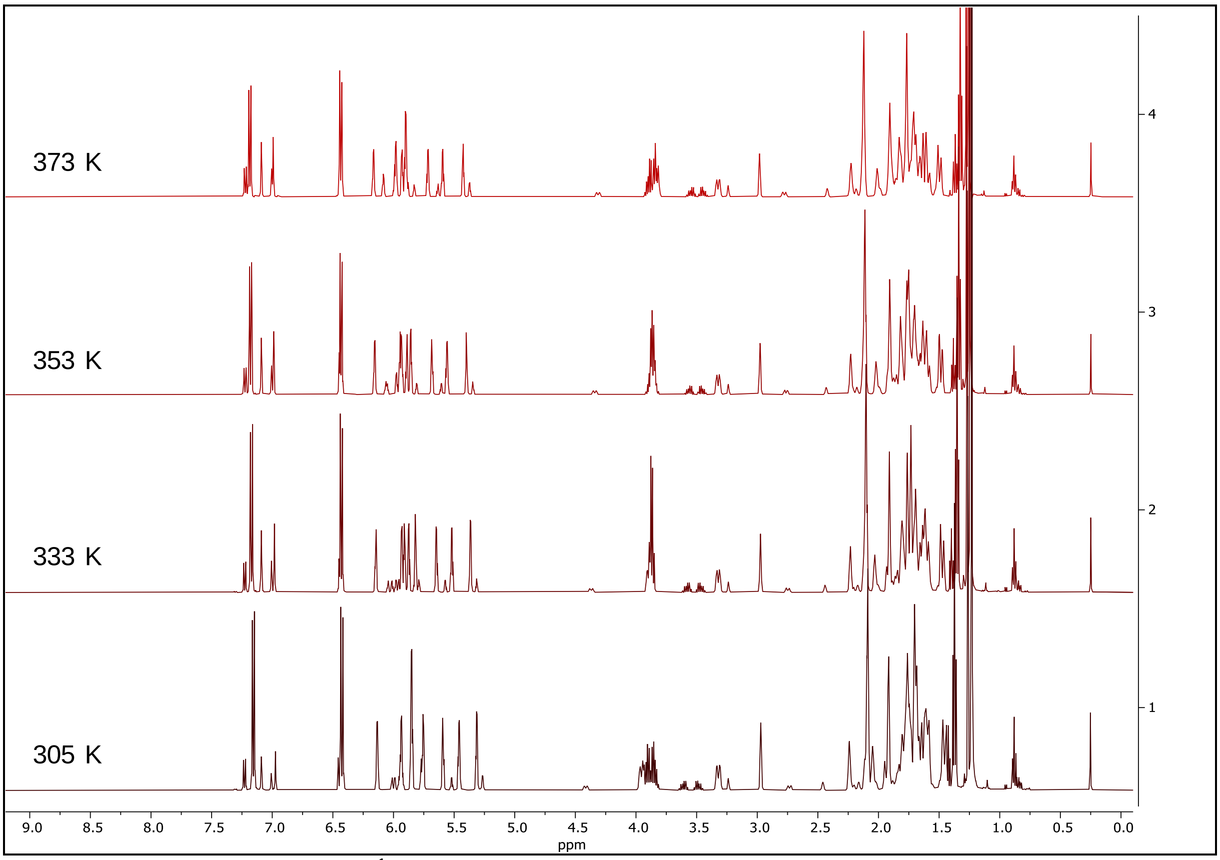

Figure S55: Variable temperature ^{1}H NMR spectrum of **4m/4m'** (500 MHz, toluene-d_8, 305 K - 373 K).

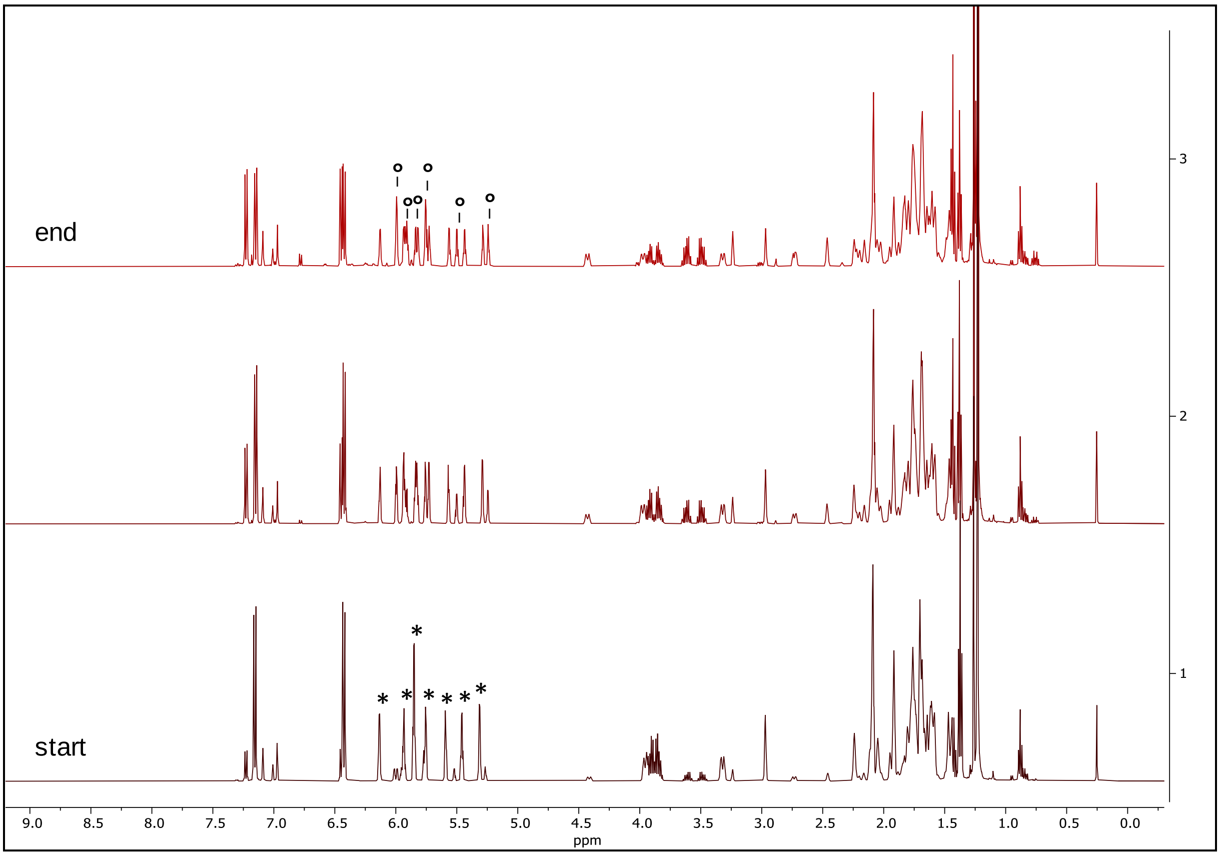

Figure S56: Stacked ^{1}H NMR spectra of **4m/4m'** (500 MHz, toluene-d_8, 305 K, **3a** = *, **3a'** = °) after heating the NMR tube at 120 °C for 4 weeks.

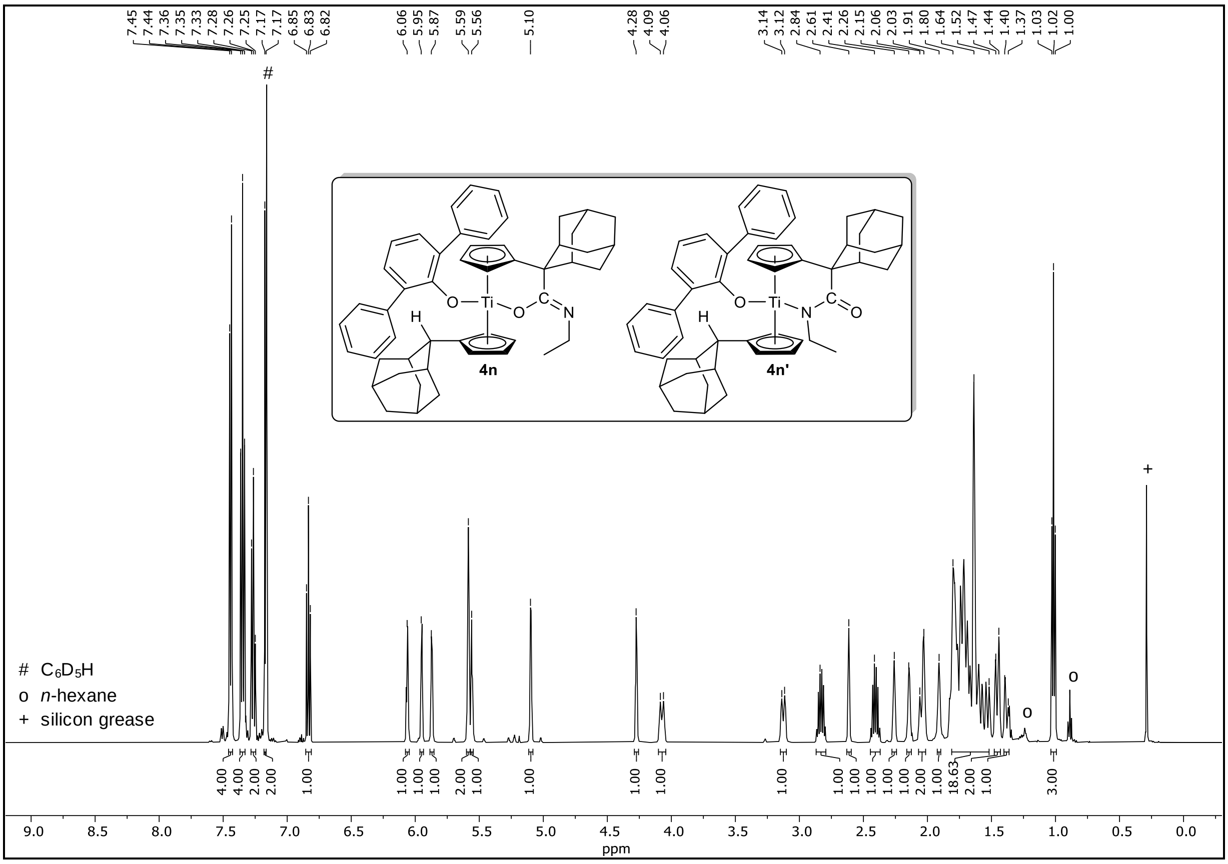

Figure S57: ^{1}H NMR spectrum of **4n/4n'** (500 MHz, C_6D_6, 305 K). Signals are marked for **4n'**.

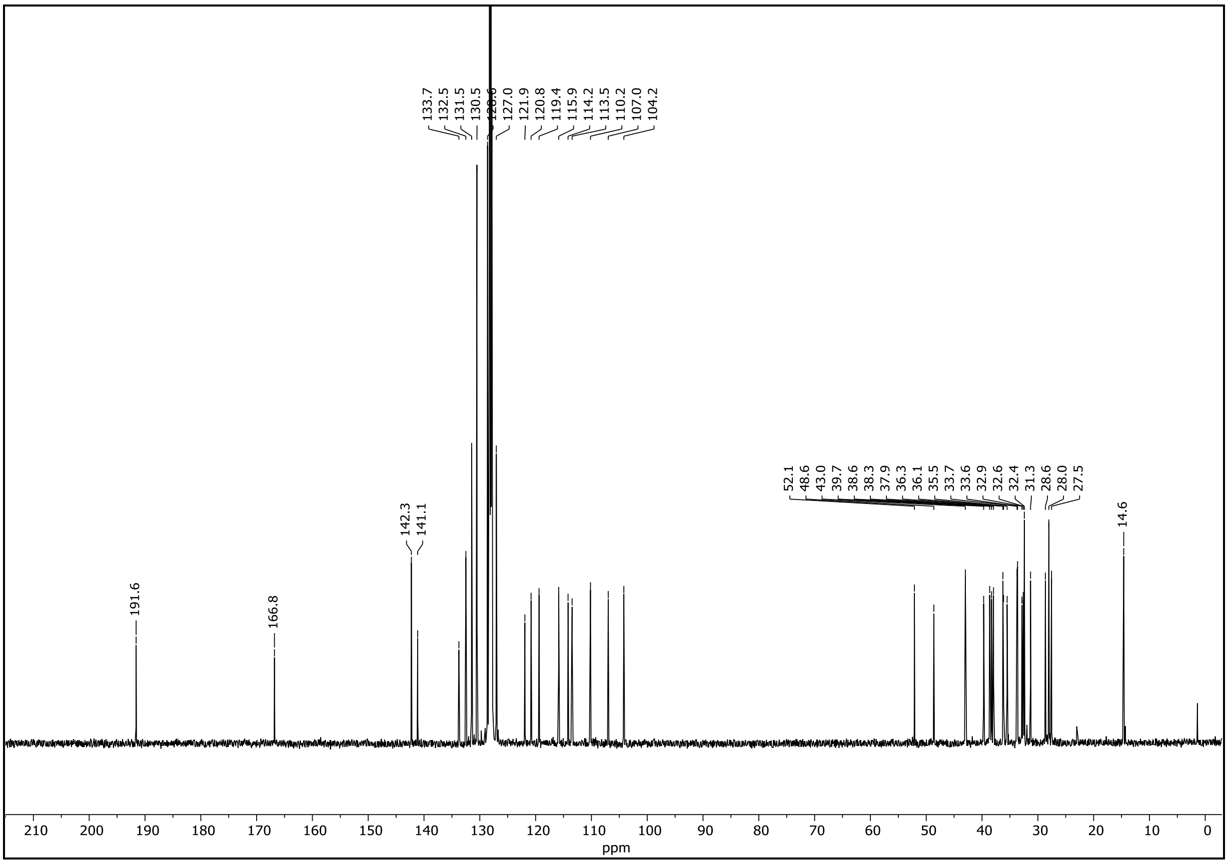

Figure S58: $^{13}C\{^1H\}$ NMR spectrum of **4n/4n'** (126 MHz, C_6D_6, 305 K). Signals are marked for **4n'**.

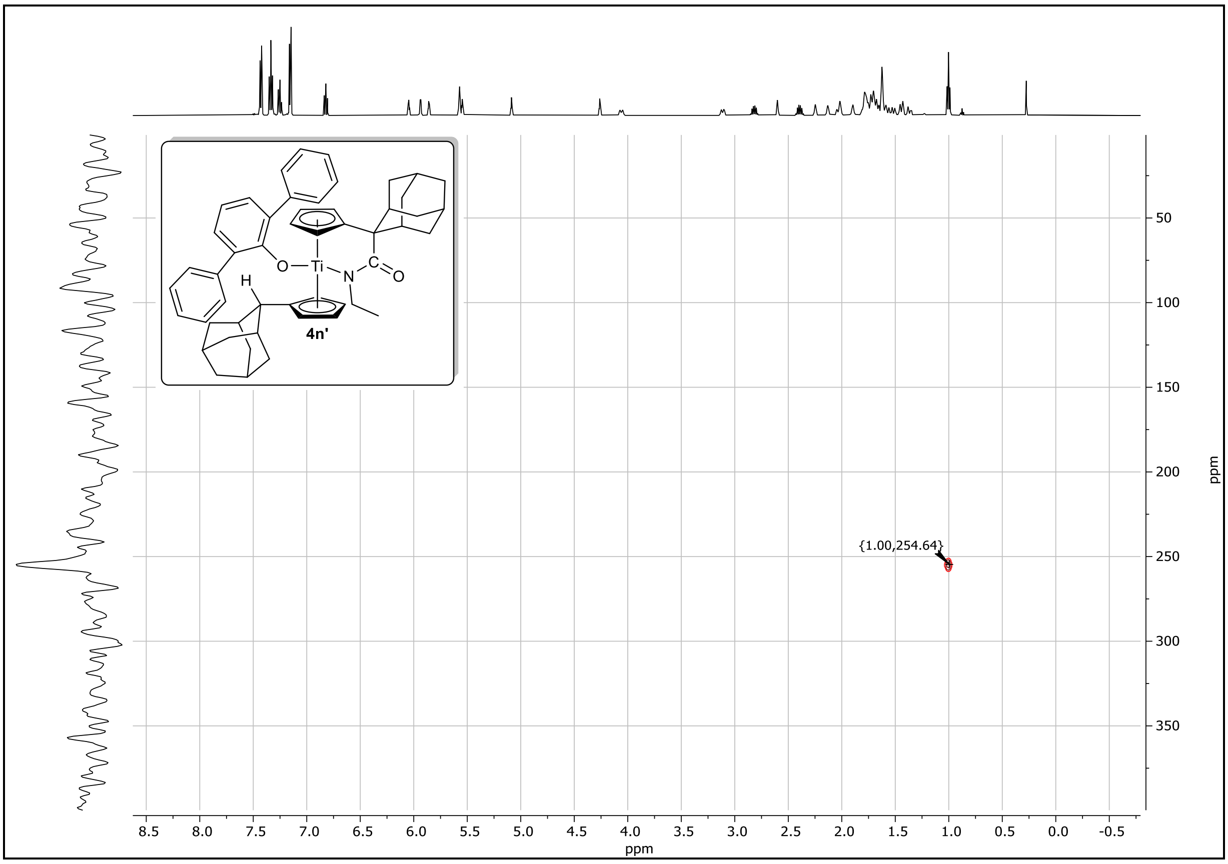

Figure S59: ^{1}H,^{15}N HMBC NMR spectrum of **4n/4n’** (500 MHz, 51 MHz, C_6D_6, 305 K). Signals are marked for **4n’**.

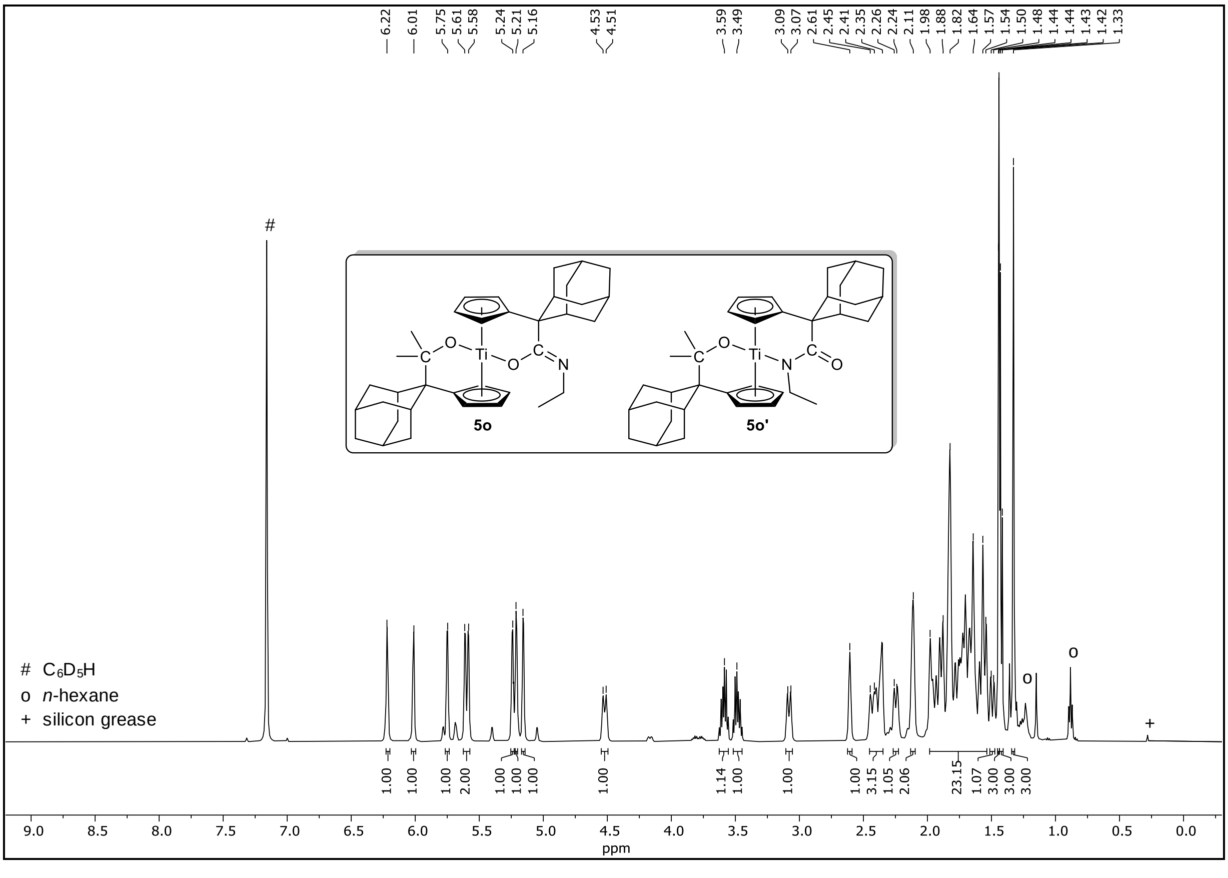

Figure S60: ^{1}H NMR spectrum of **5o/5o’** (500 MHz, C_6D_6, 305 K). Signals are marked for **5o**.

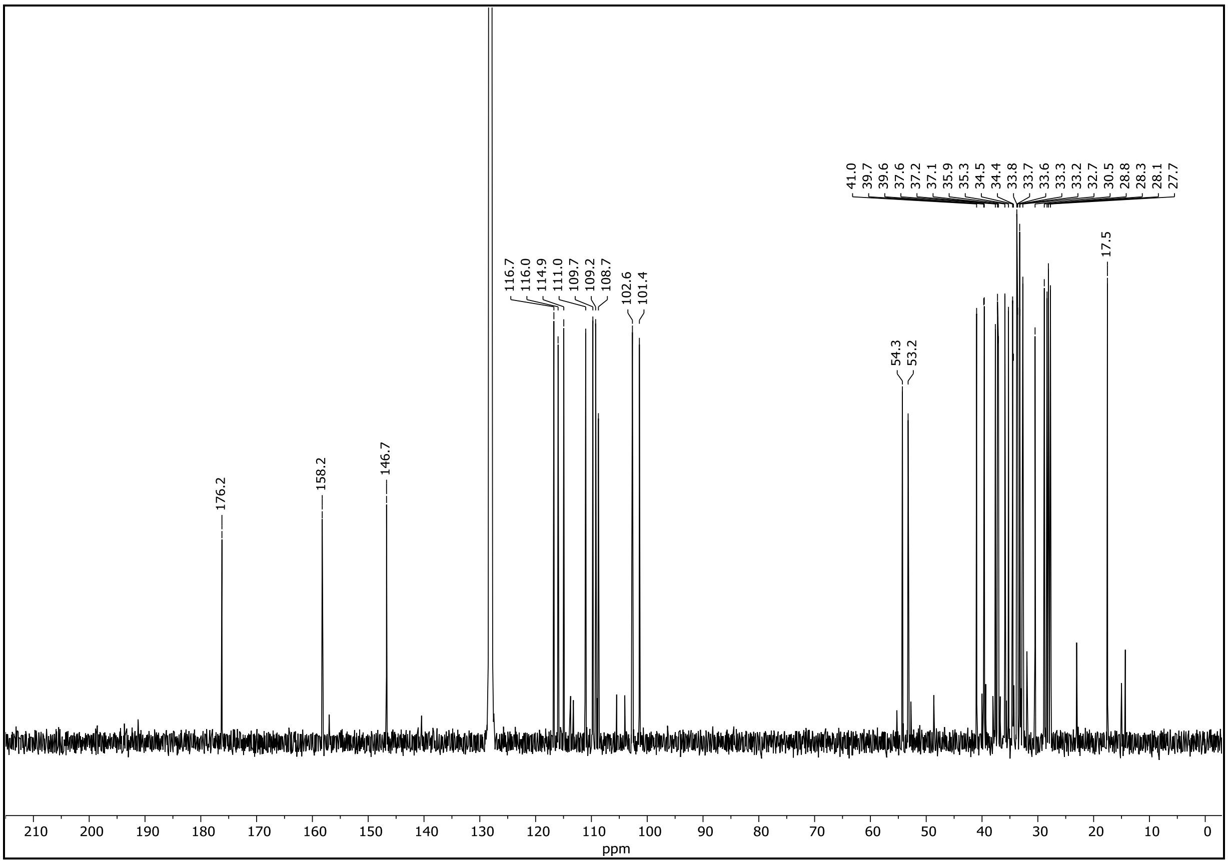

Figure S61: $^{13}C\{^1H\}$ NMR spectrum of **5o/5o'** (126 MHz, C_6D_6, 305 K). Signals are marked for **5o**.

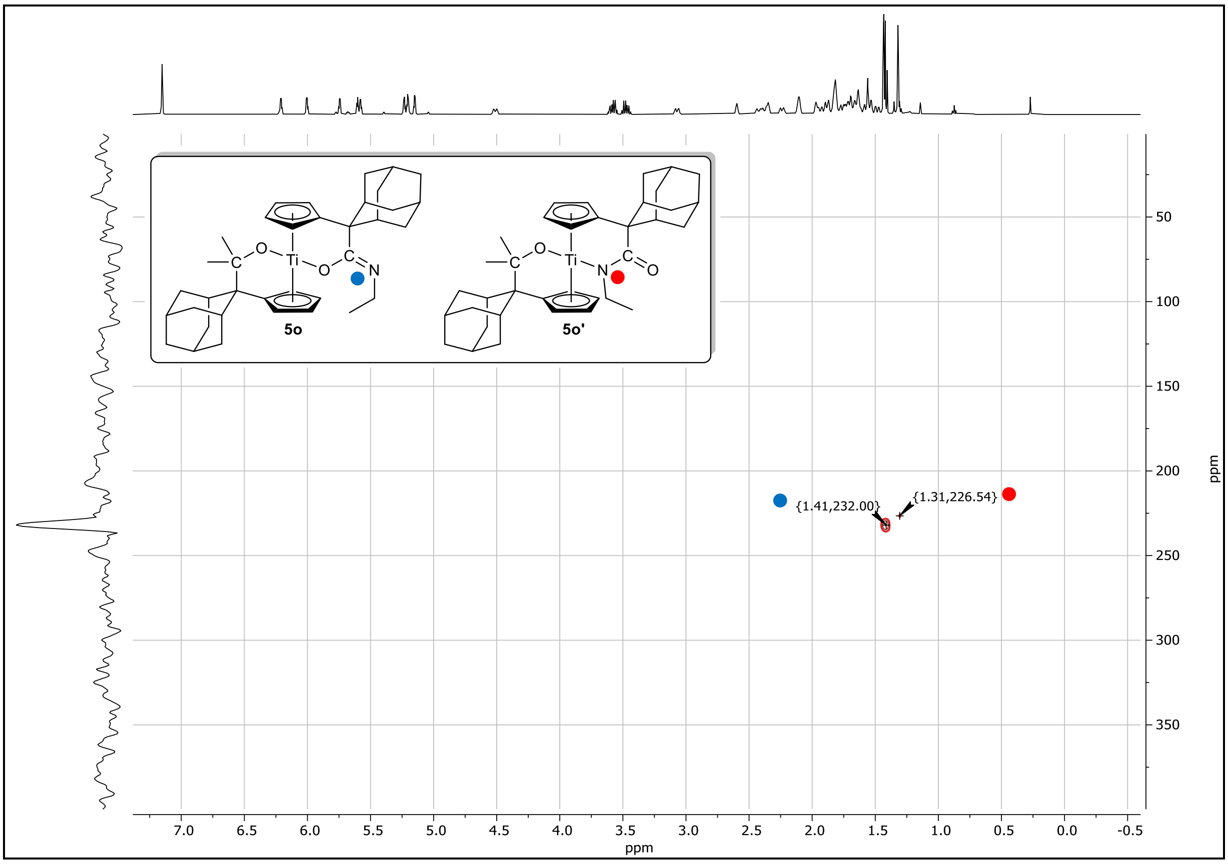

Figure S62: $^1H,^{15}N$ HMBC NMR spectrum of **5o/5o'** (500 MHz, 51 MHz, C_6D_6, 305 K). Signals are marked for **5o** and **5o'**.

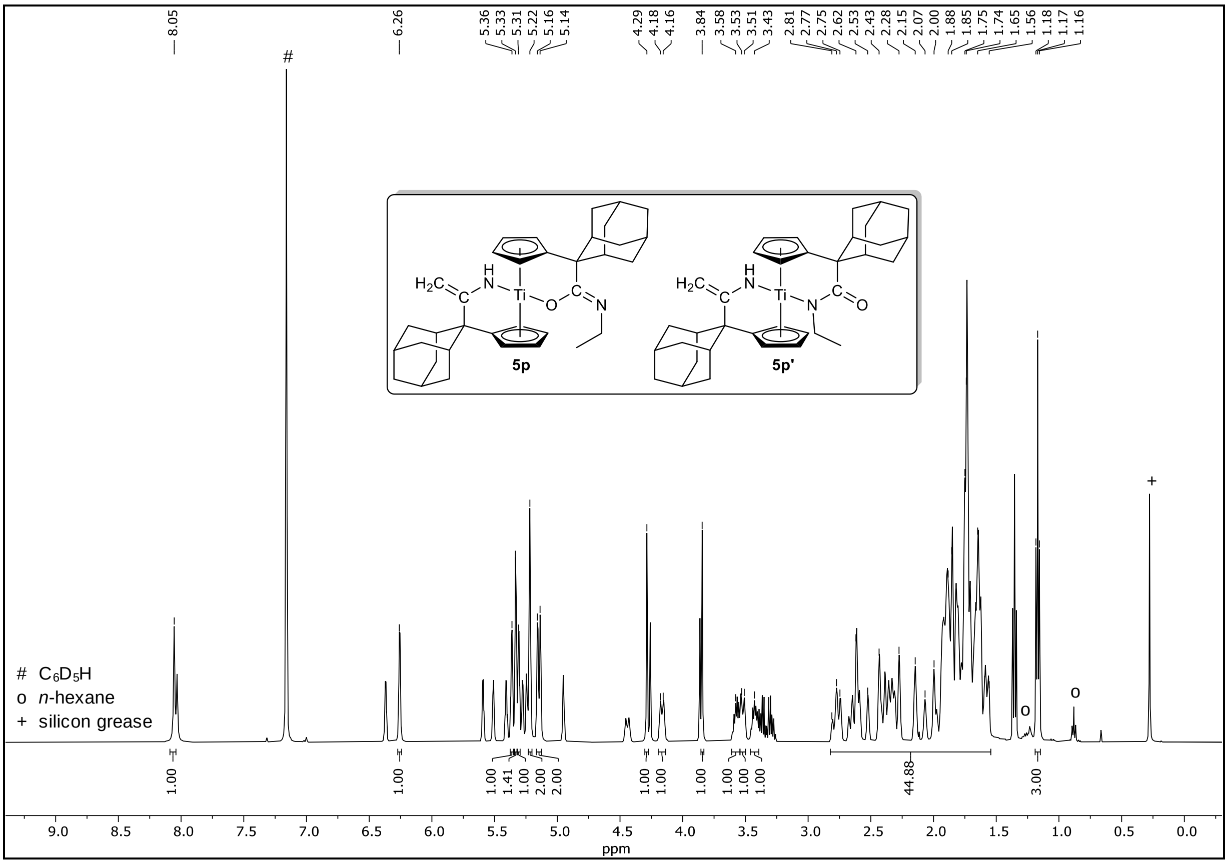

Figure S63: ^{1}H NMR spectrum of **5p/5p'** (500 MHz, C_6D_6, 305 K). Signals are marked for **5p**.

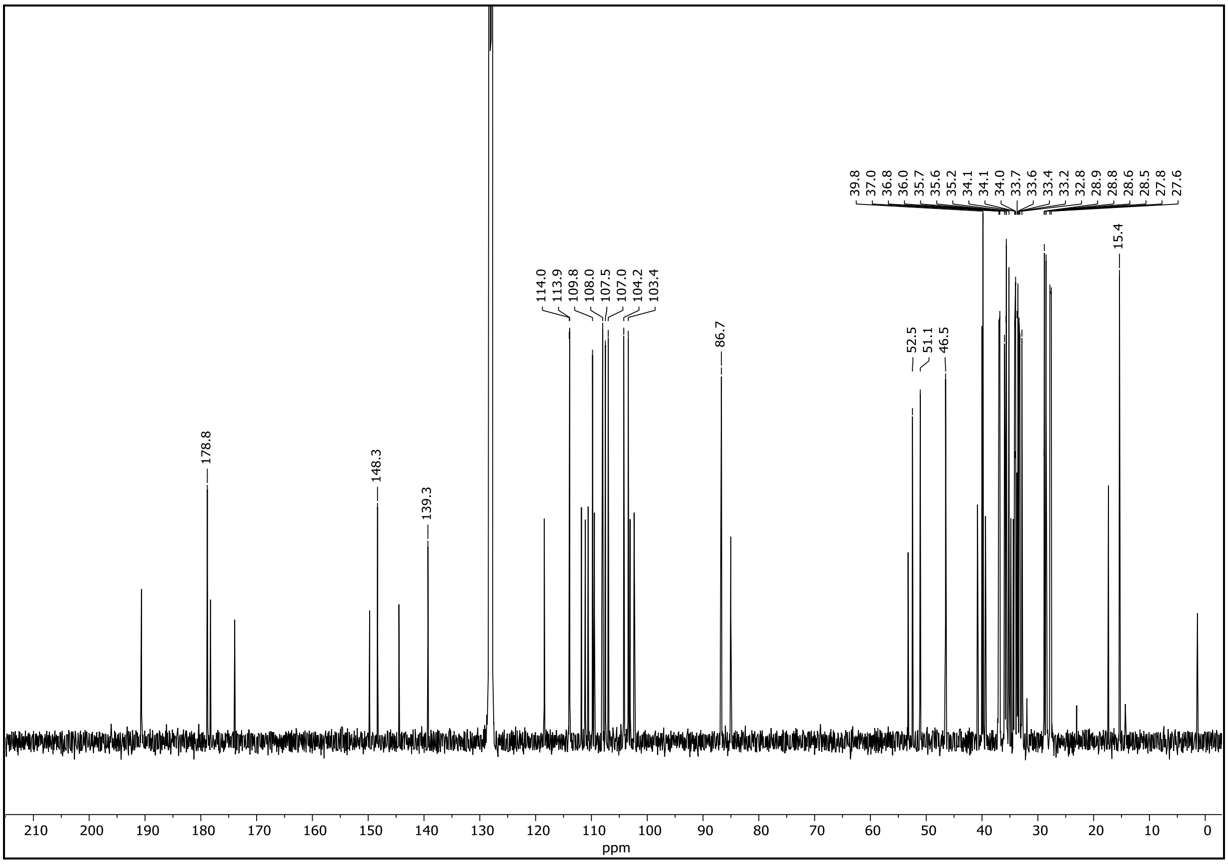

Figure S64: ^{13}C{^{1}H} NMR spectrum of **5p/5p'** (126 MHz, C_6D_6, 305 K). Signals are marked for **5p**.

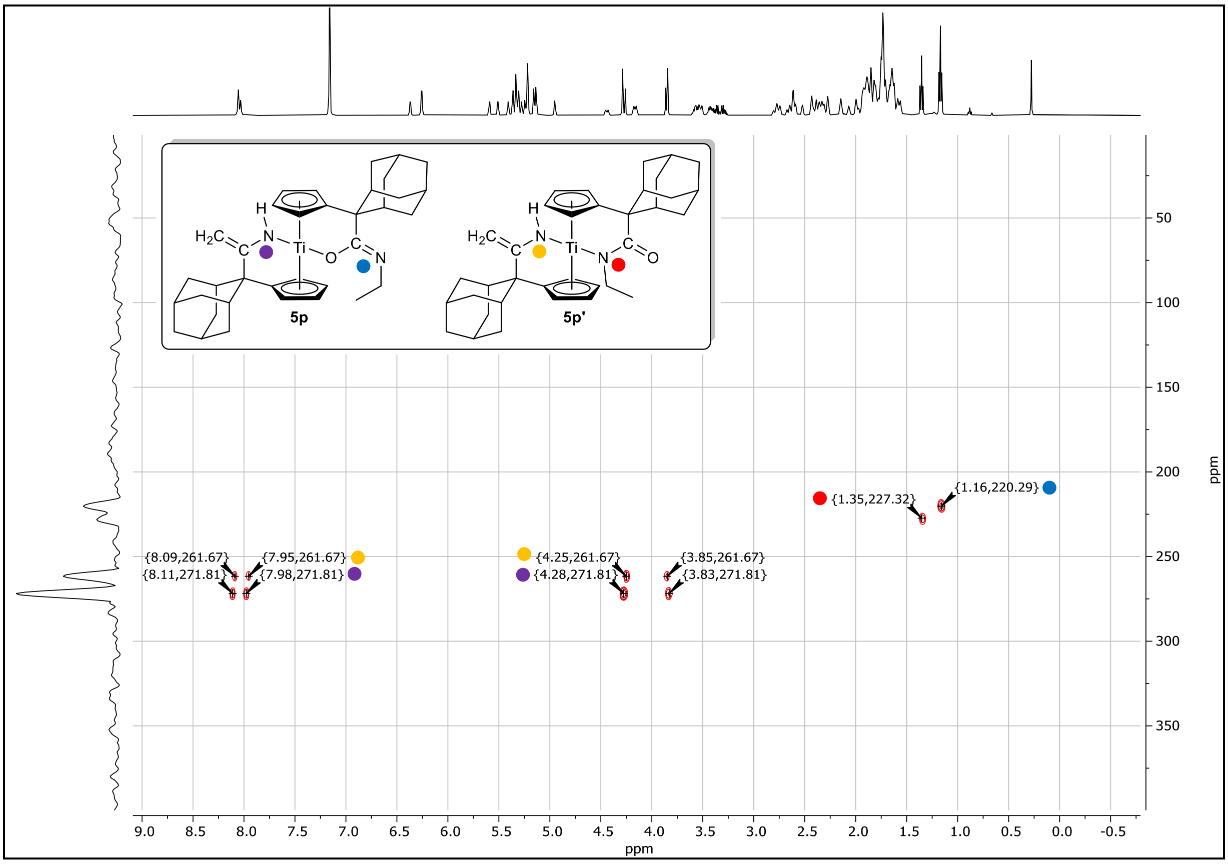

Figure S65: $^{1}H,^{15}N$ HMBC NMR spectrum of **5p/5p'** (500 MHz, 51 MHz, C_6D_6, 305 K). Signals are marked for **5o** and **5p'**.

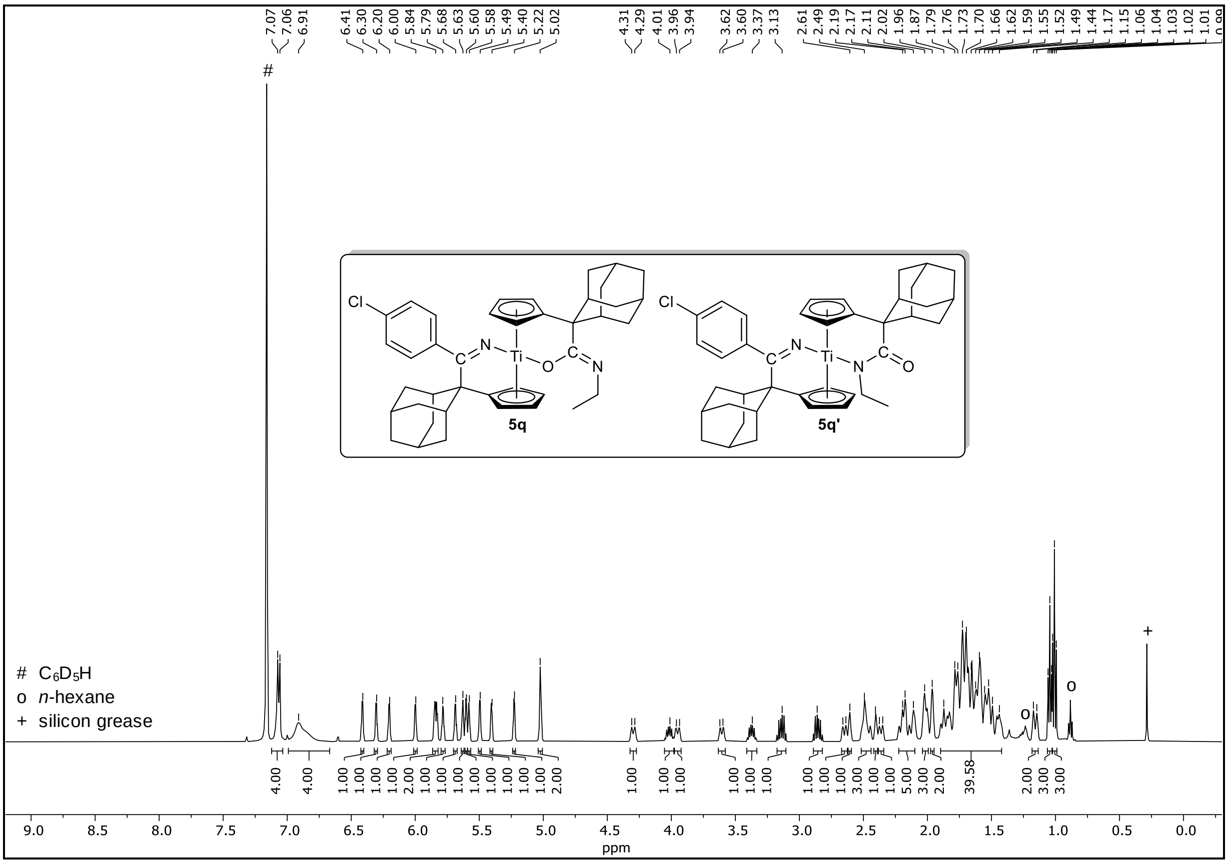

Figure S66: ^{1}H NMR spectrum of **5q/5q'** (500 MHz, C_6D_6, 305 K). Signals are marked for **5q** and **5q'**.

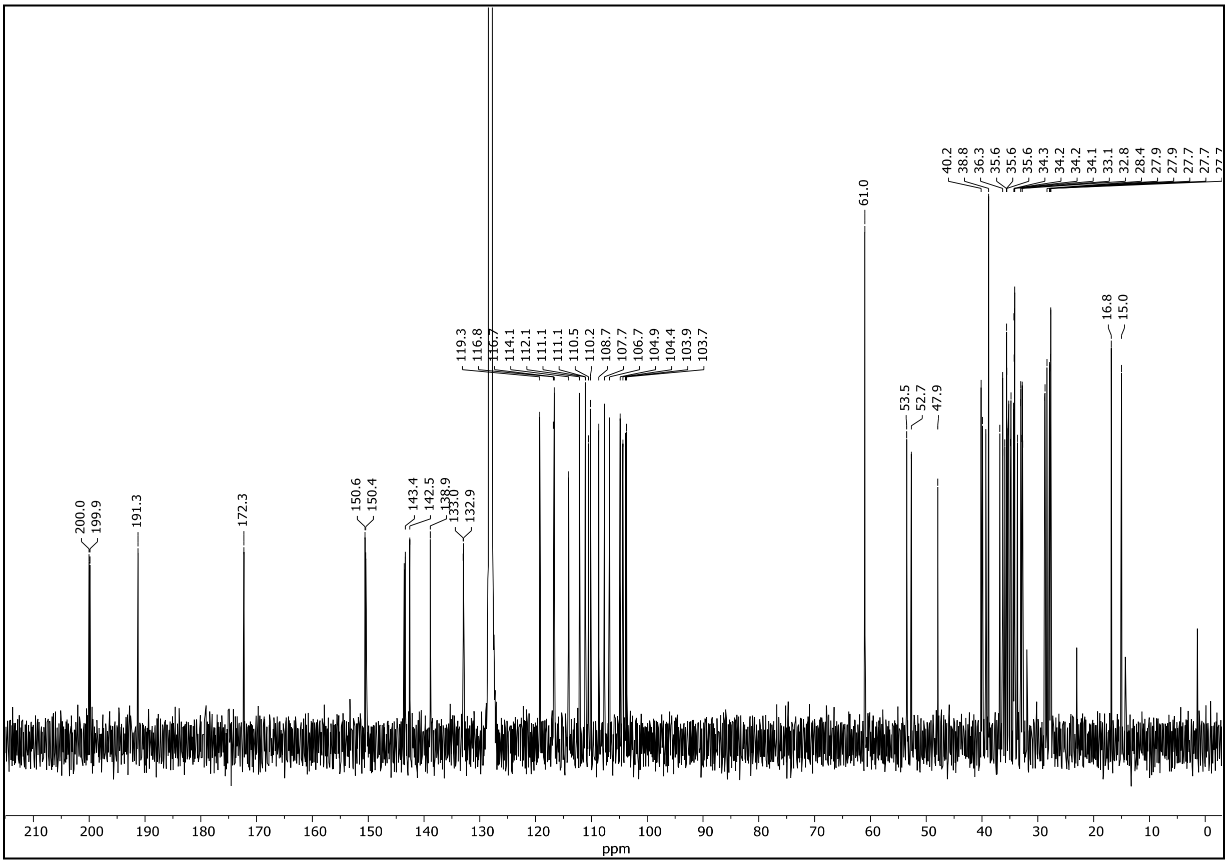

Figure S67: $^{13}C\{^1H\}$ NMR spectrum of **5q/5q'** (126 MHz, C_6D_6, 305 K). Signals are marked for **5q** and **5q'**.

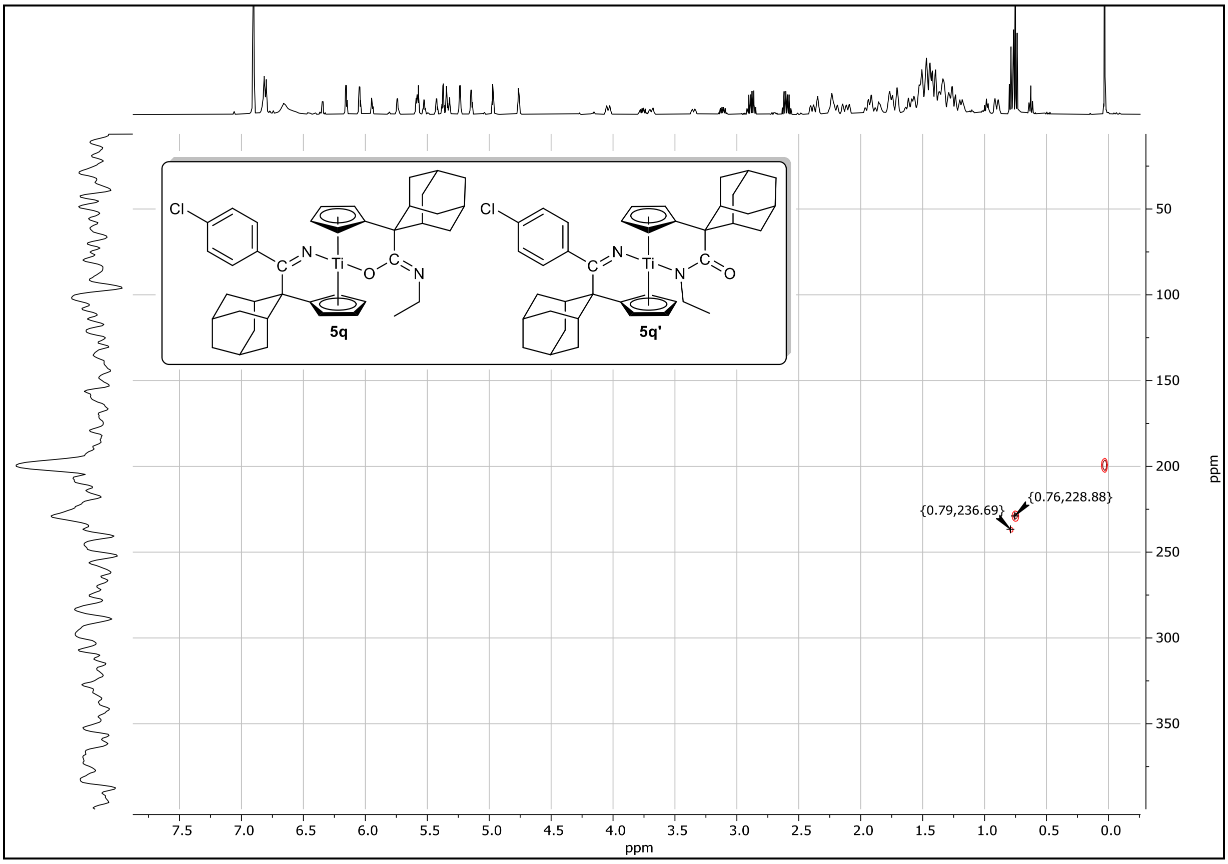

Figure S68: $^1H,^{15}N$ HMBC NMR spectrum of **5q/5q'** (500 MHz, 51 MHz, C_6D_6, 305 K). Signals are marked for **5q** and **5q'**.

IR Spectra

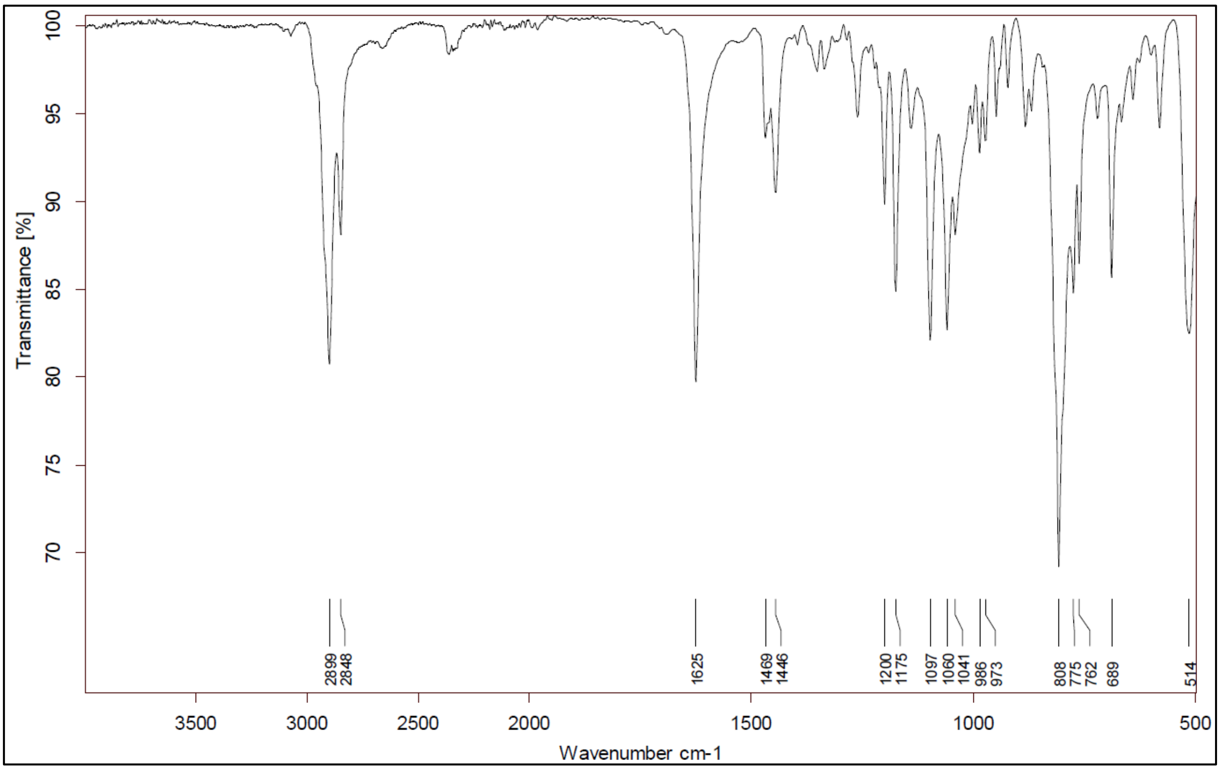

Figure S69: IR (ATR) spectrum of **1a**.

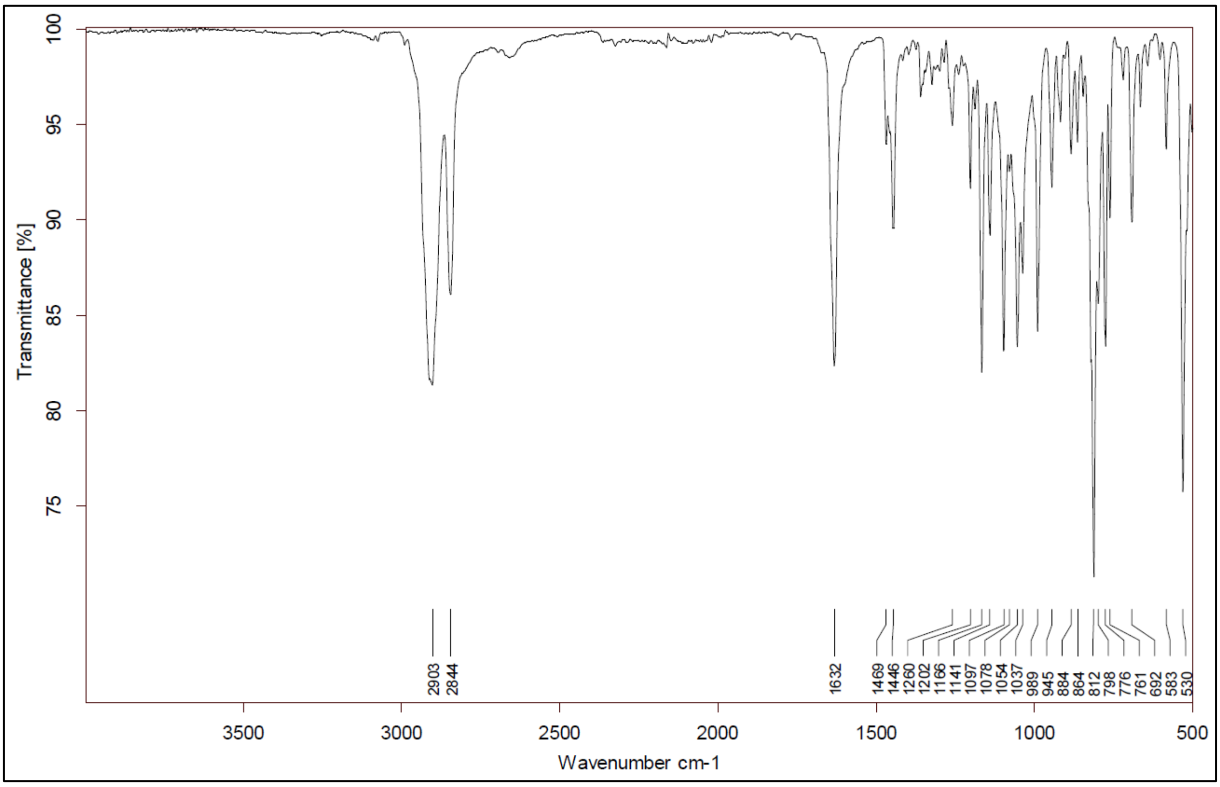

Figure S70: IR (ATR) spectrum of **1b**.

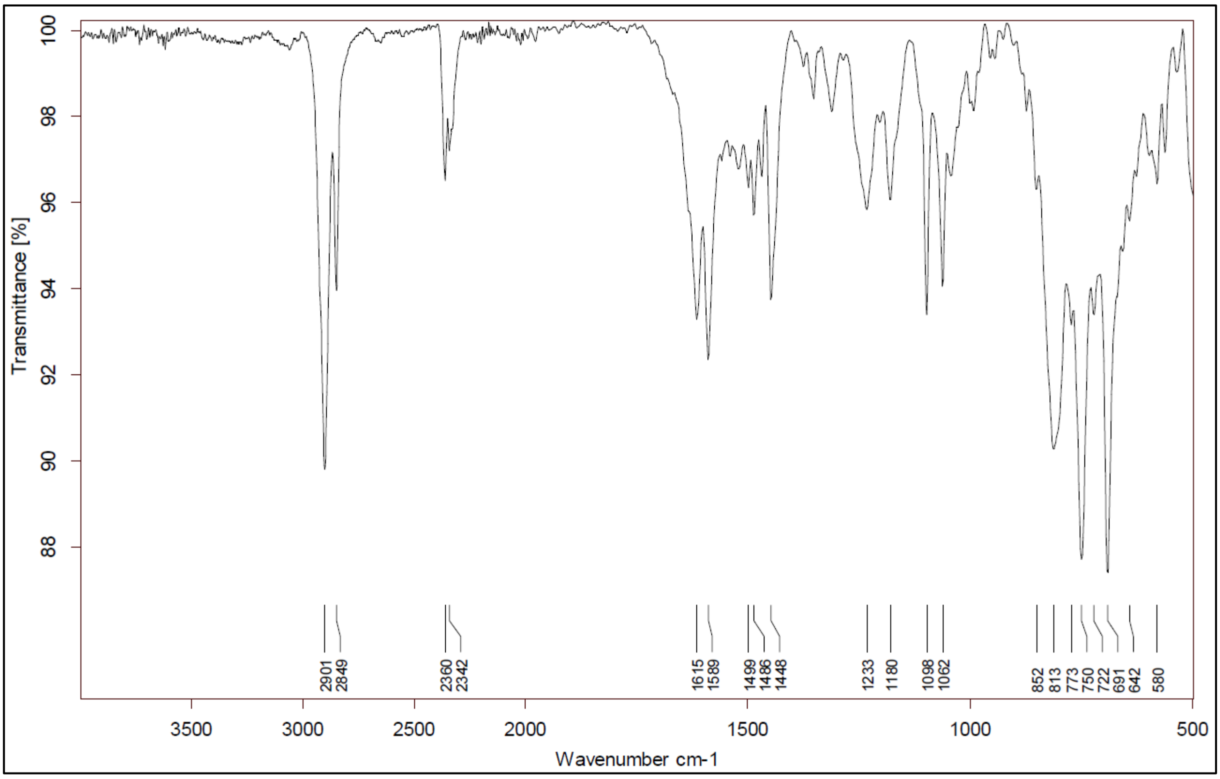

Figure S71: IR (ATR) spectrum of **1c**.

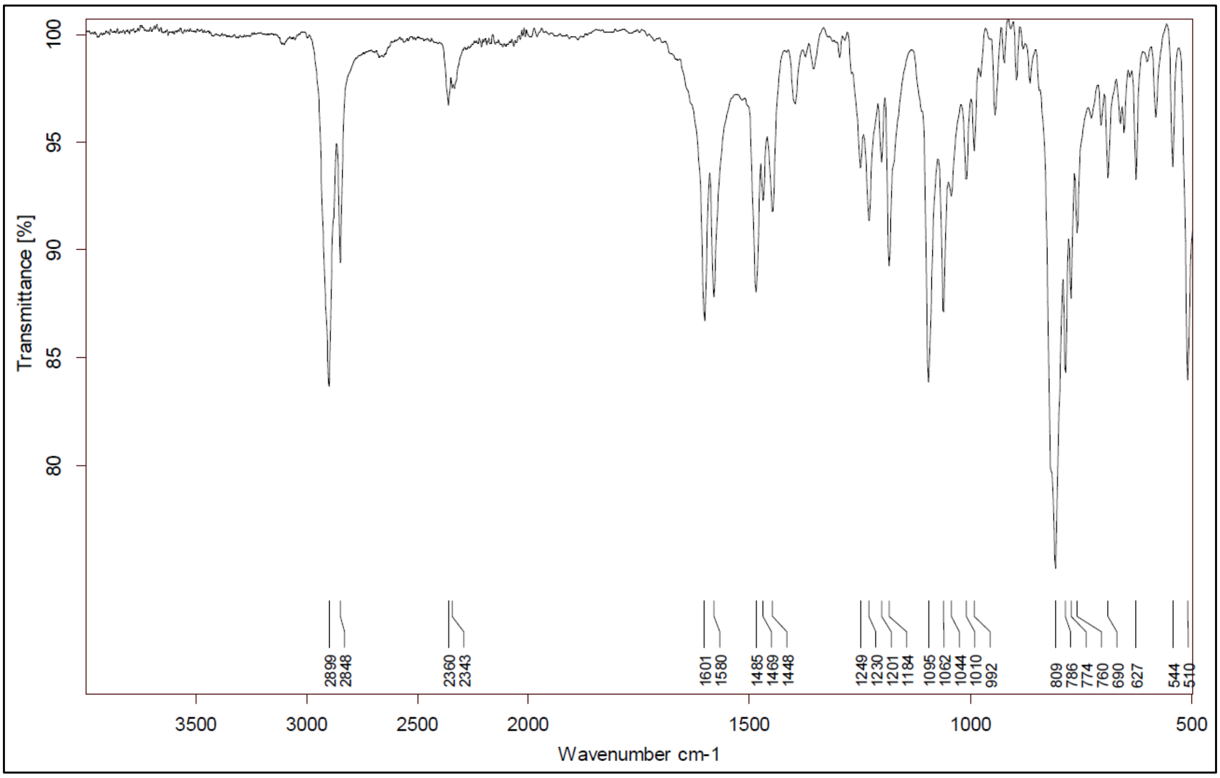

Figure S72: IR (ATR) spectrum of **1d**.

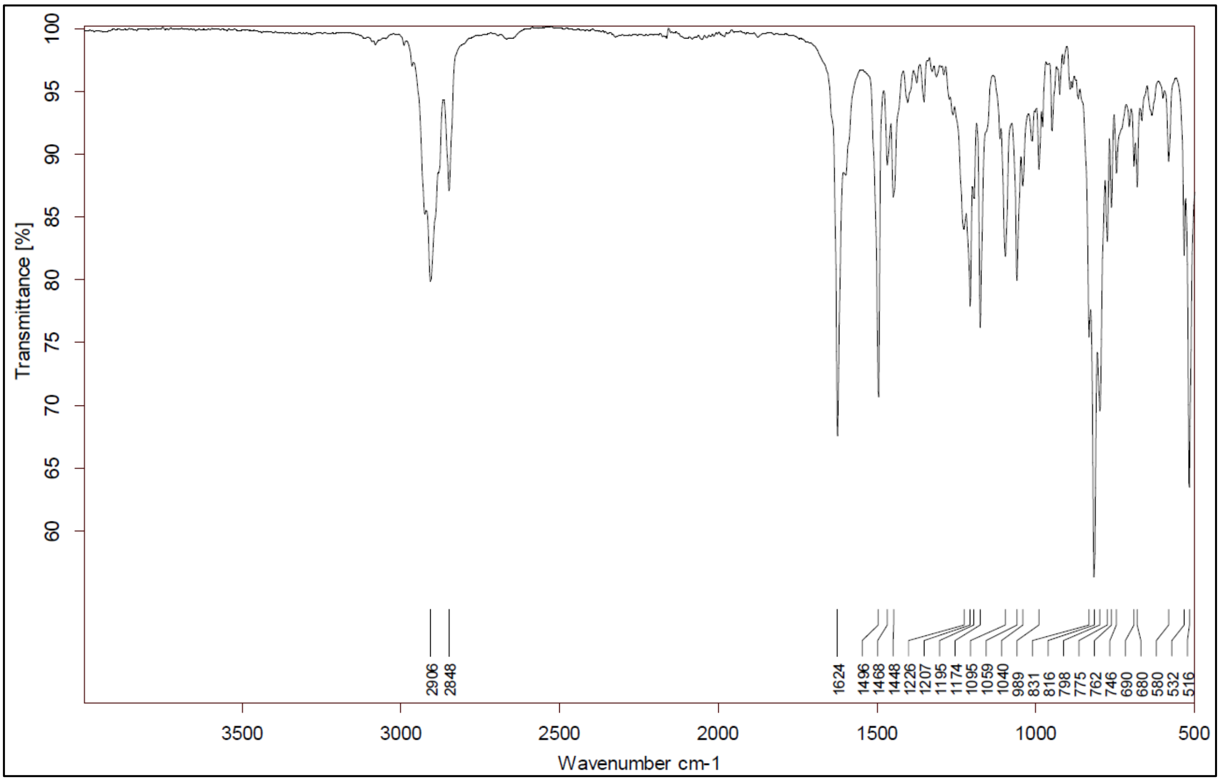

Figure S73: IR (ATR) spectrum of **1e**.

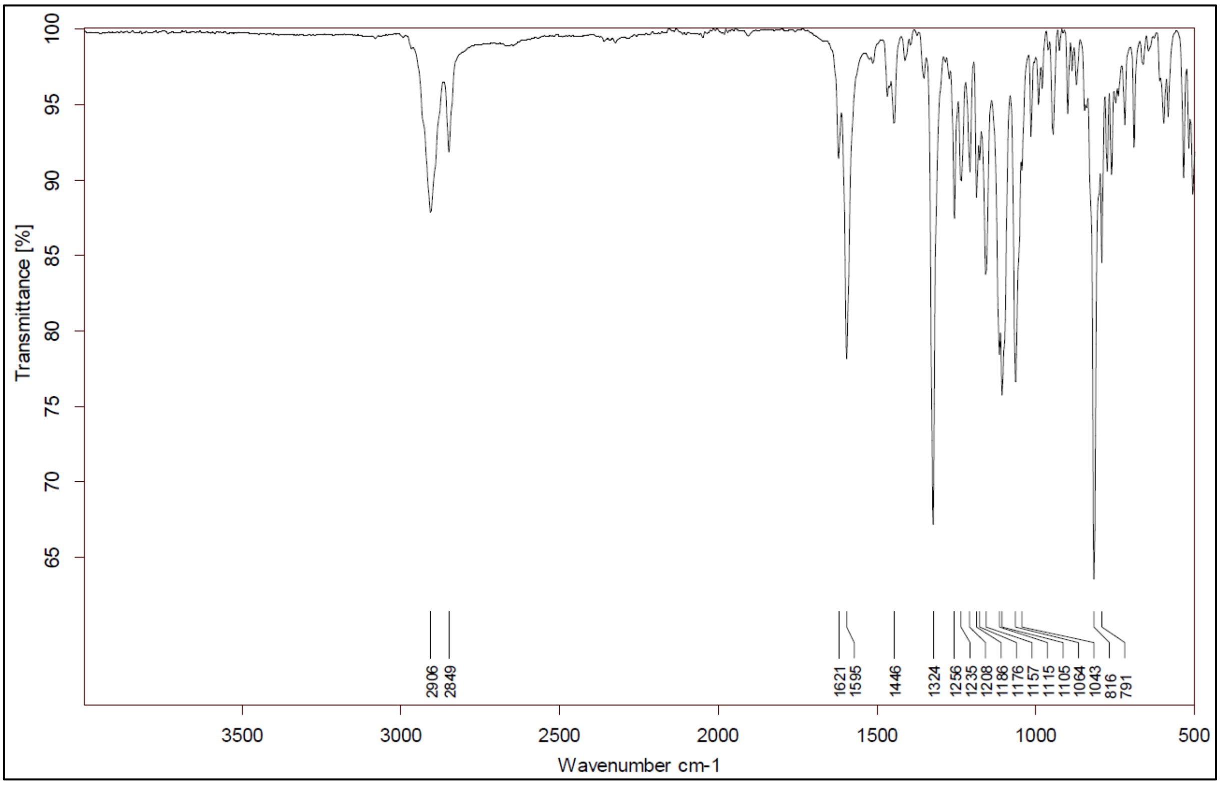

Figure S74: IR (ATR) spectrum of **1f**.

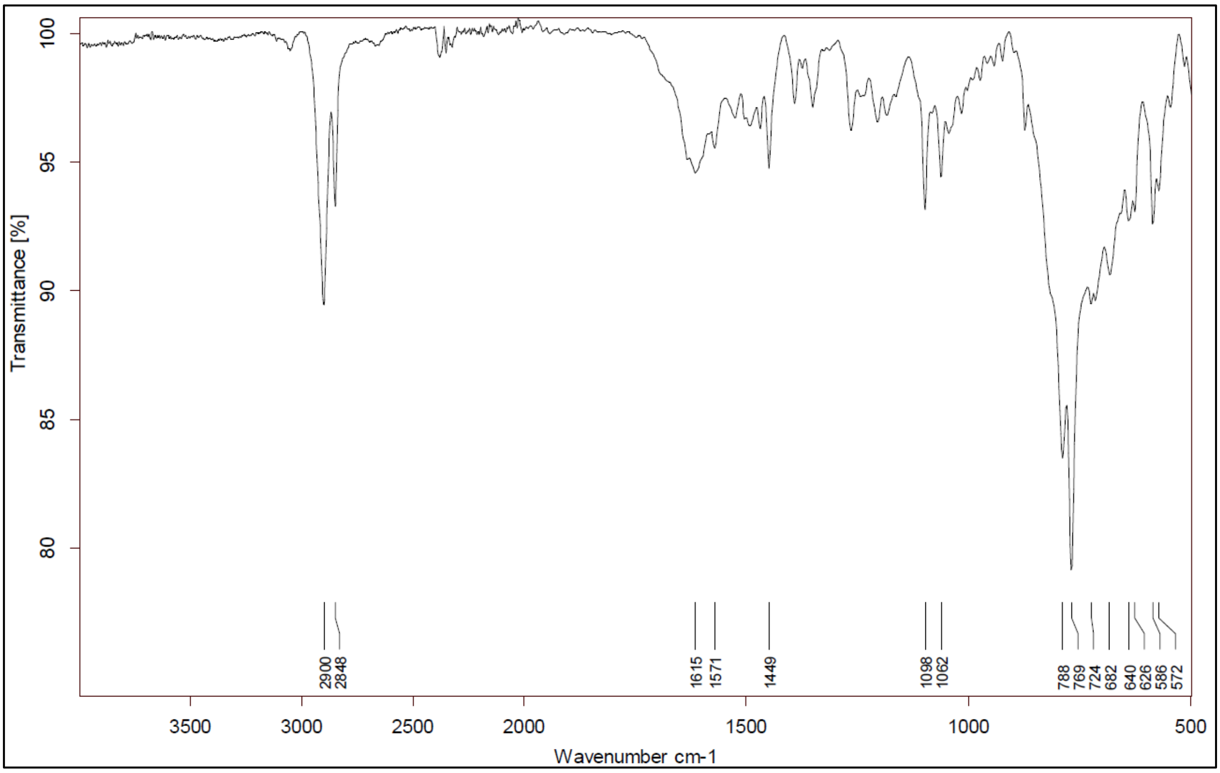

Figure S75: IR (ATR) spectrum of **1g**.

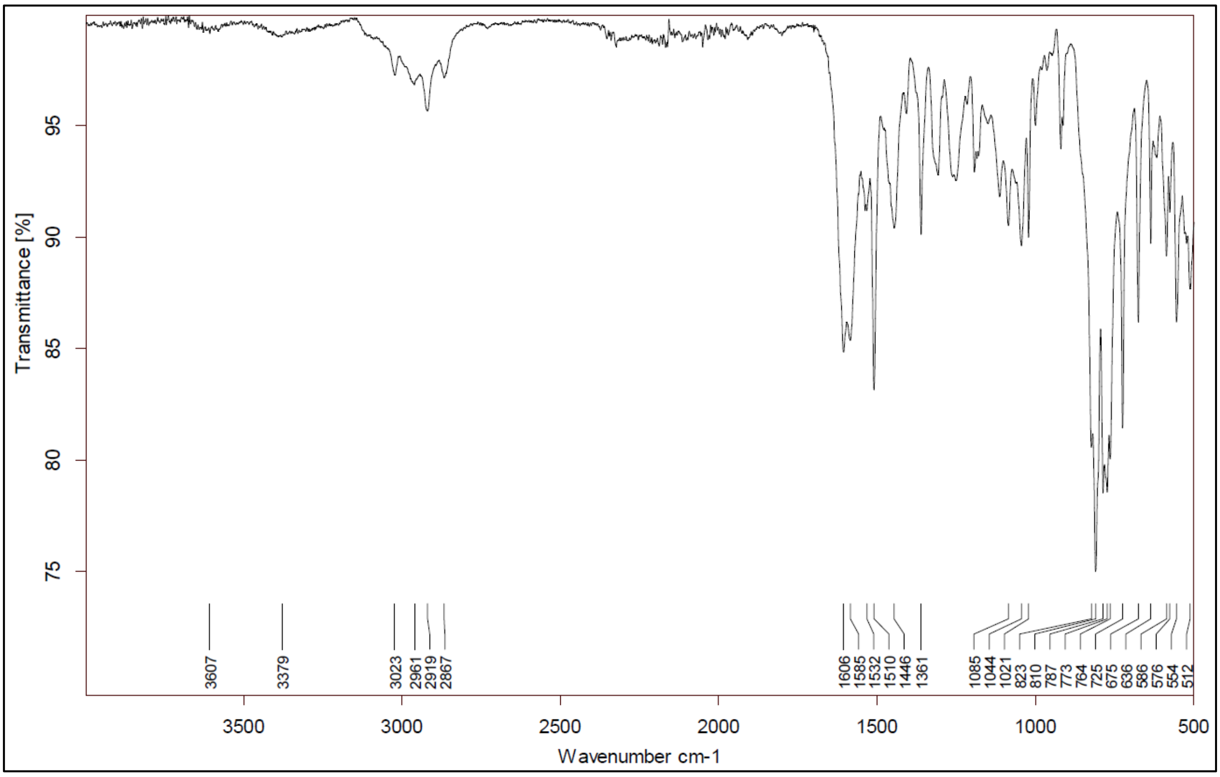

Figure S76: IR (ATR) spectrum of **2a**.

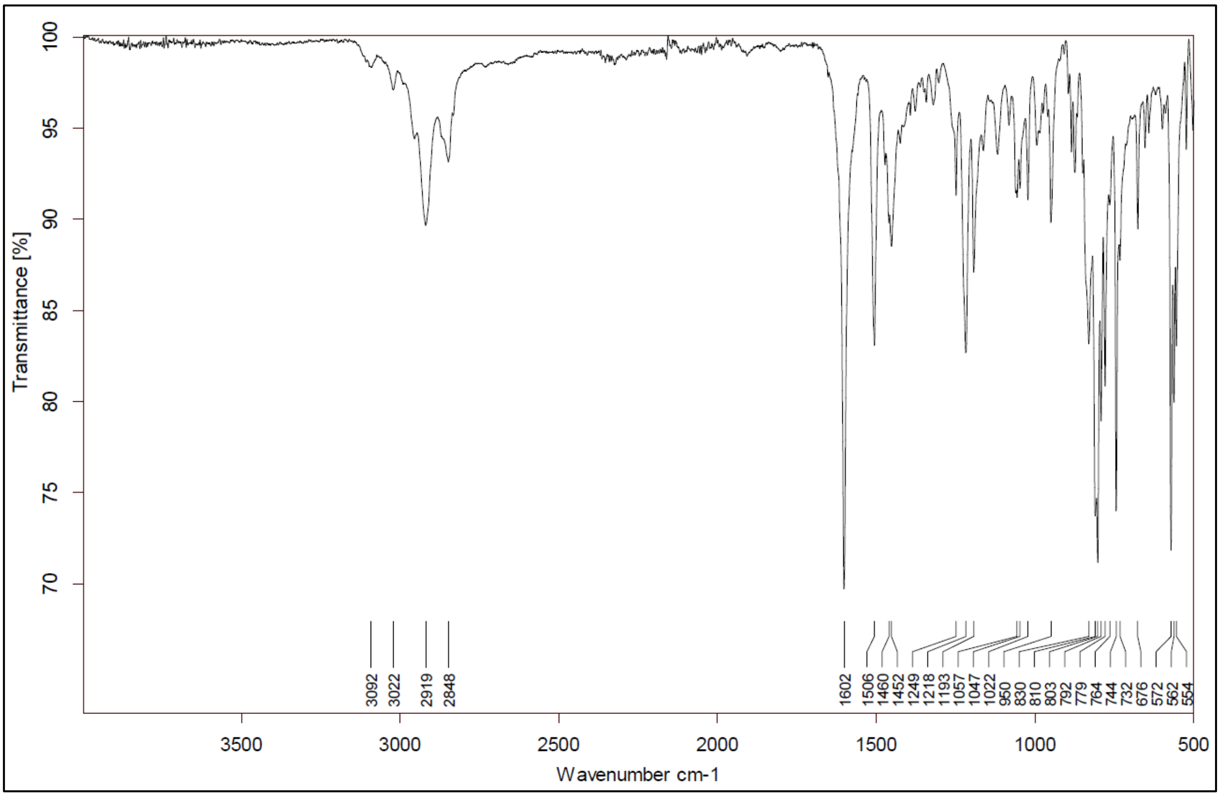

Figure S77: IR (ATR) spectrum of **2b**.

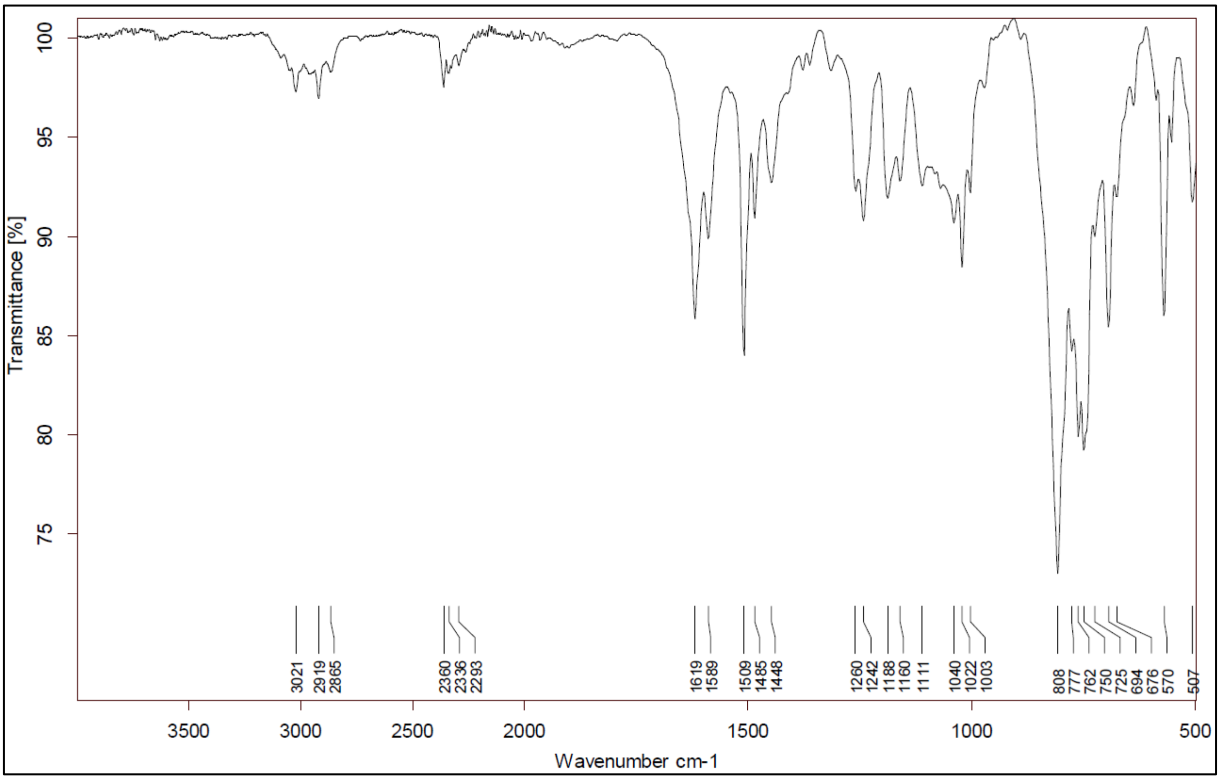

Figure S78: IR (ATR) spectrum of **2c**.

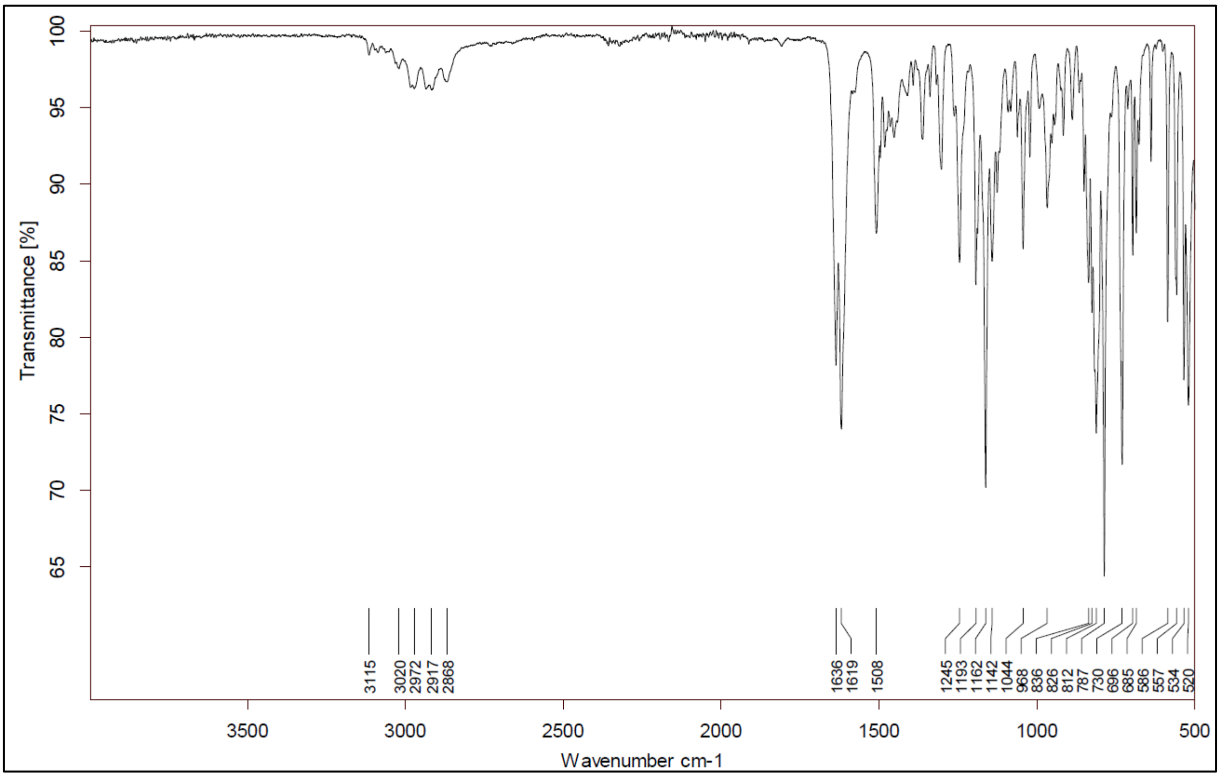

Figure S79: IR (ATR) spectrum of **3a**.

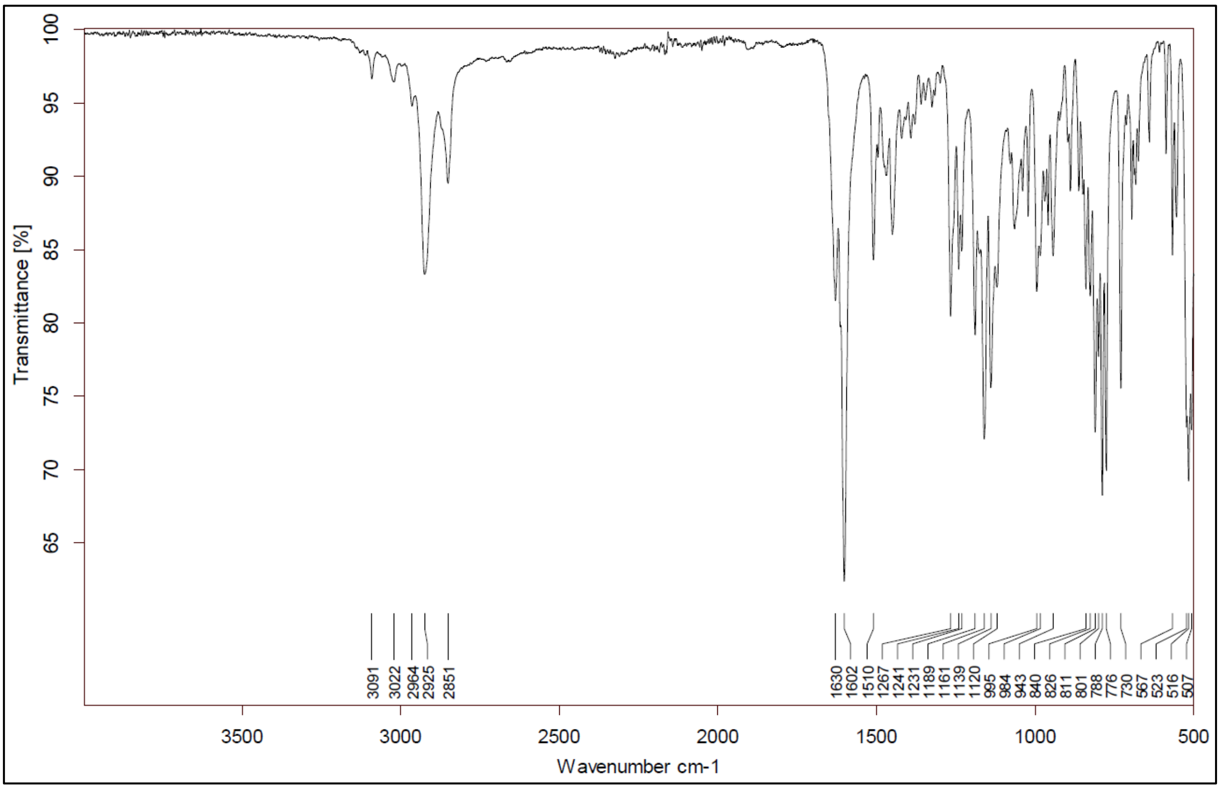

Figure S80: IR (ATR) spectrum of **3b**.

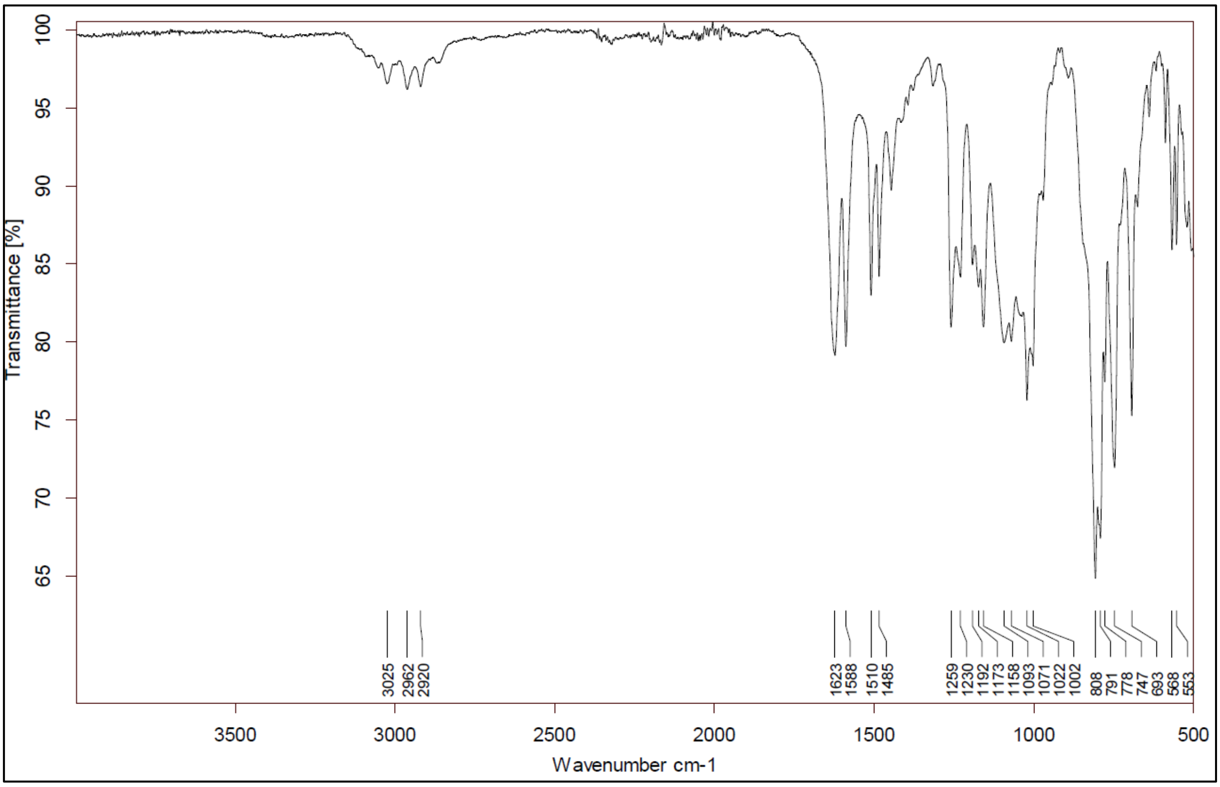

Figure S81: IR (ATR) spectrum of **3c**.

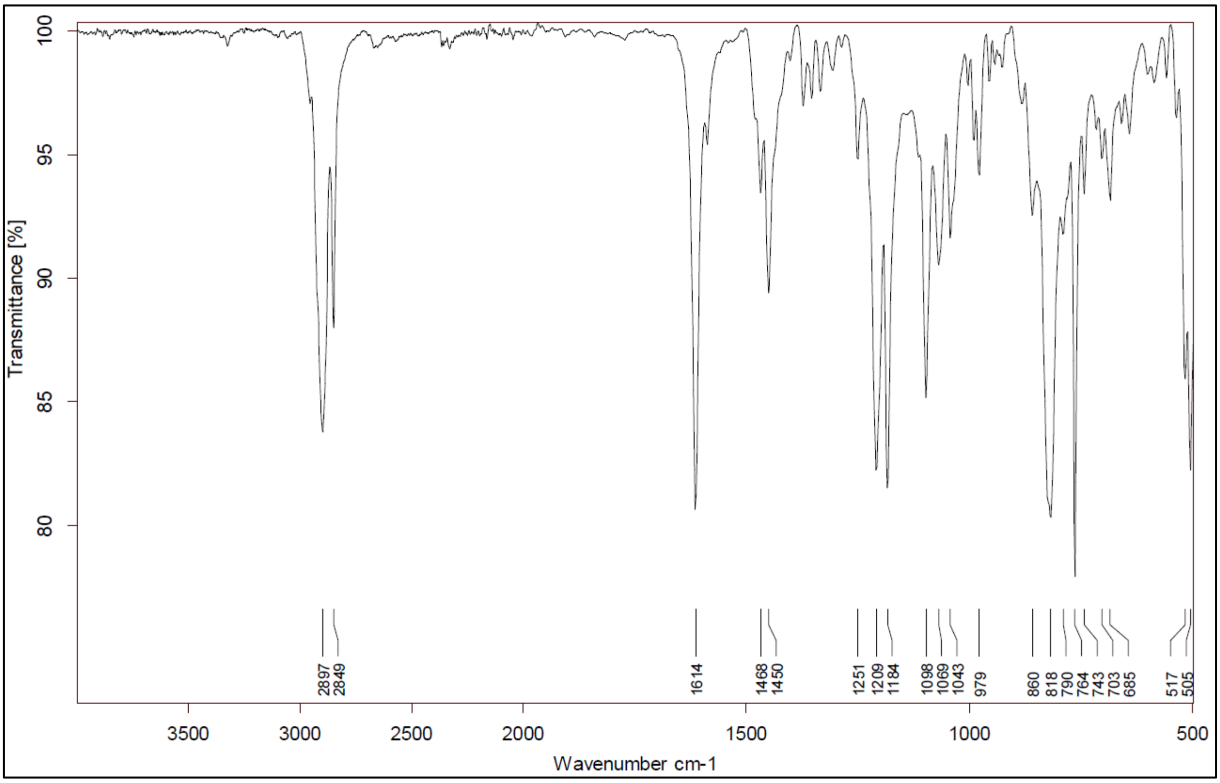

Figure S82: IR (ATR) spectrum of **4h**.

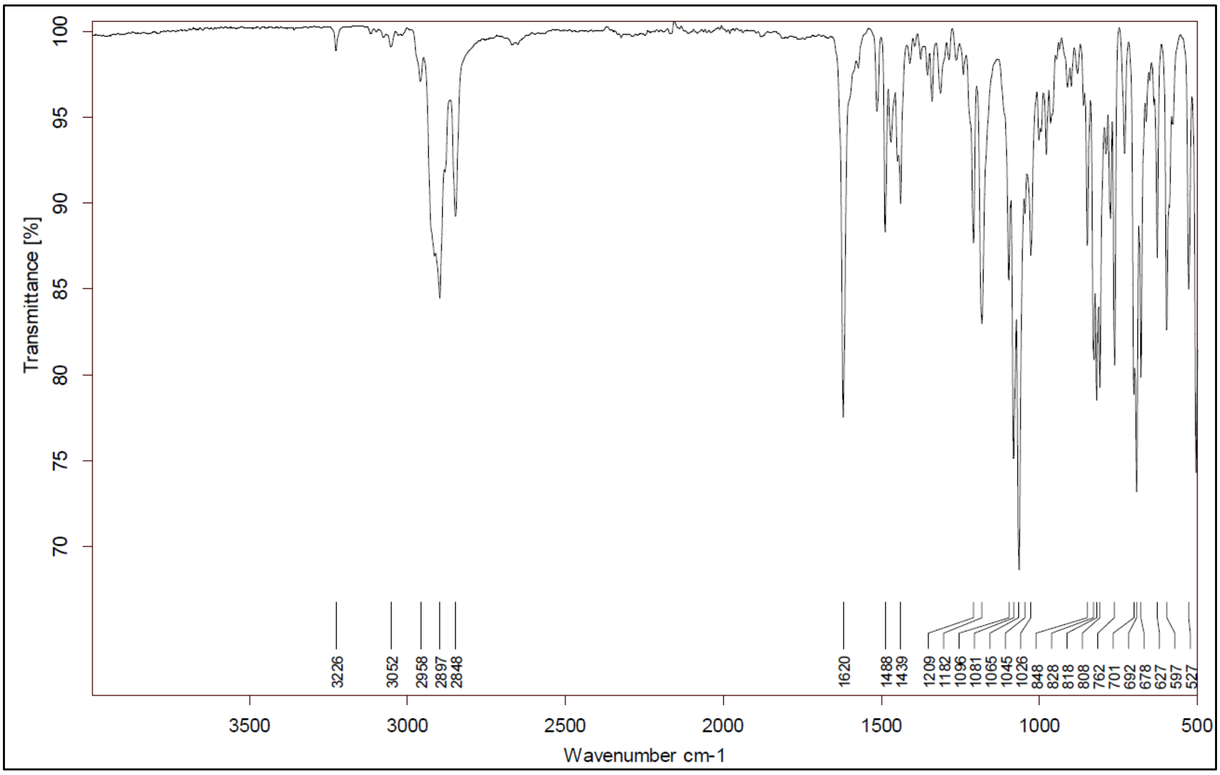

Figure S83: IR (ATR) spectrum of **4i**.

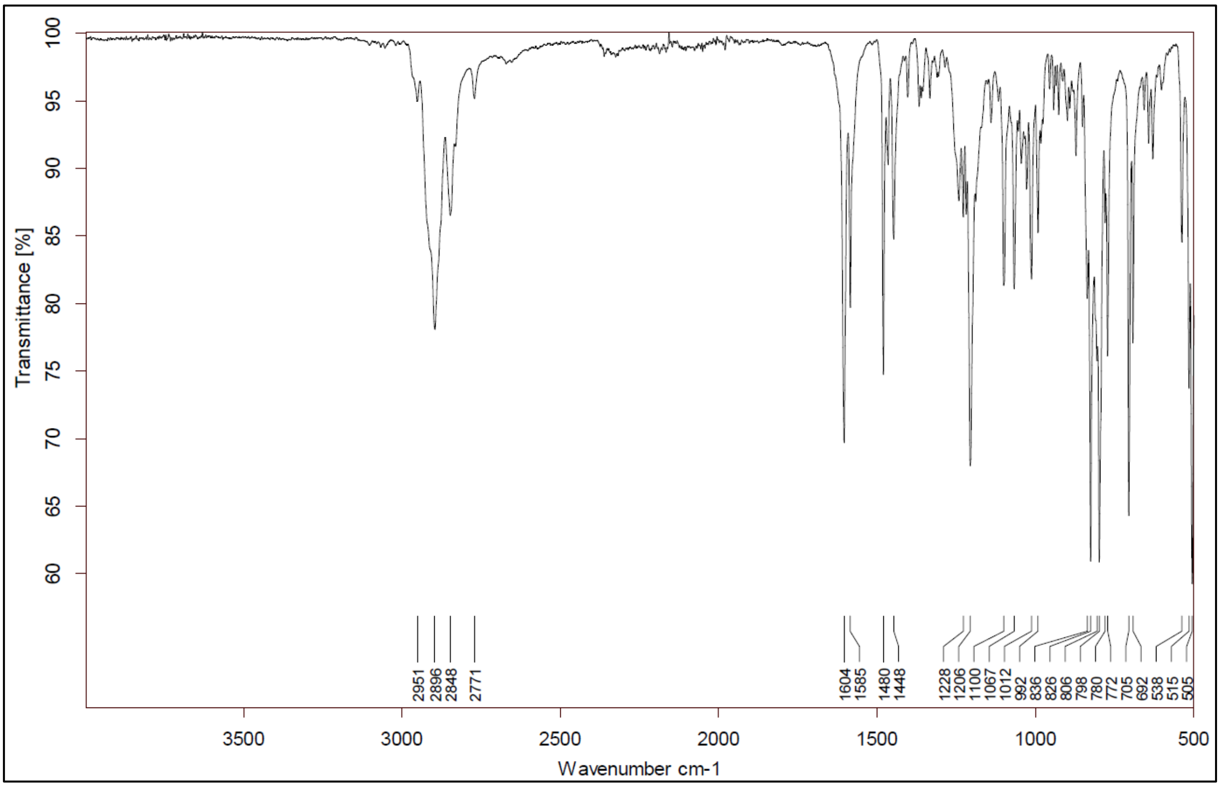

Figure S84: IR (ATR) spectrum of **4j/4j'**.

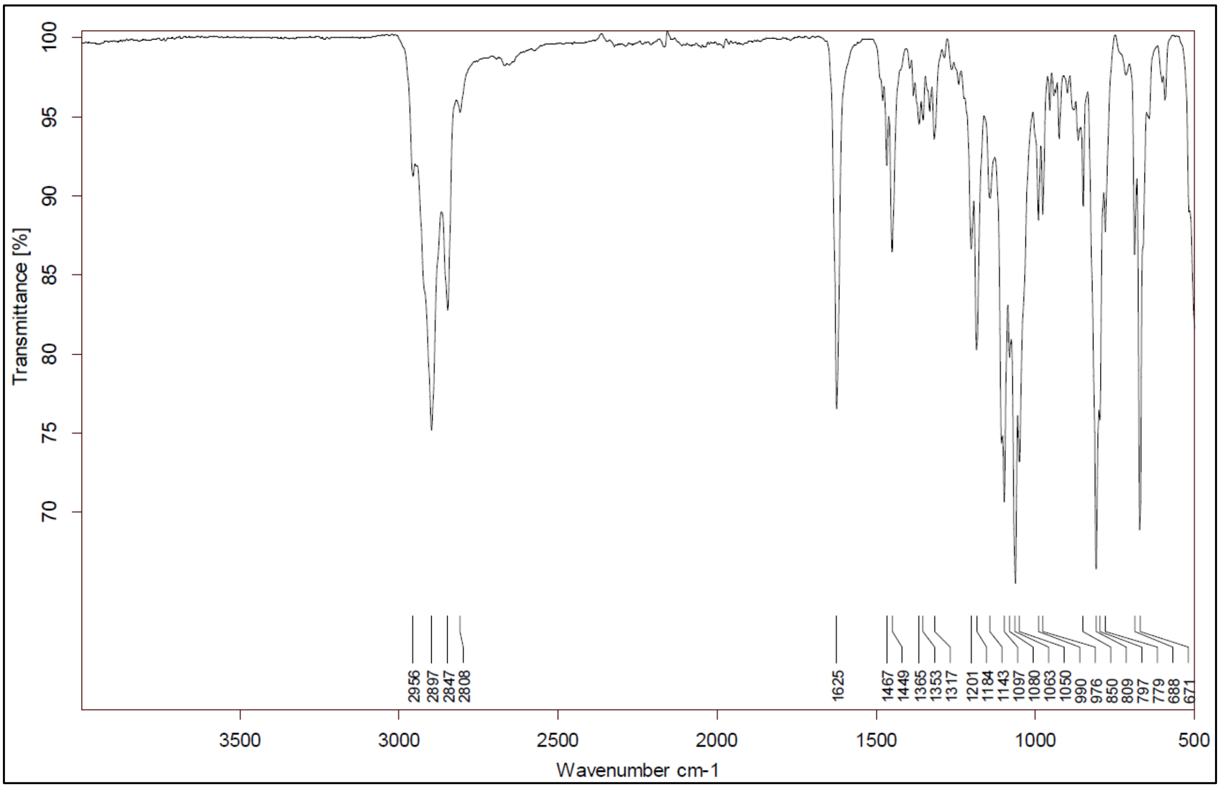

Figure S85: IR (ATR) spectrum of **4k(R)/4k(S)**.

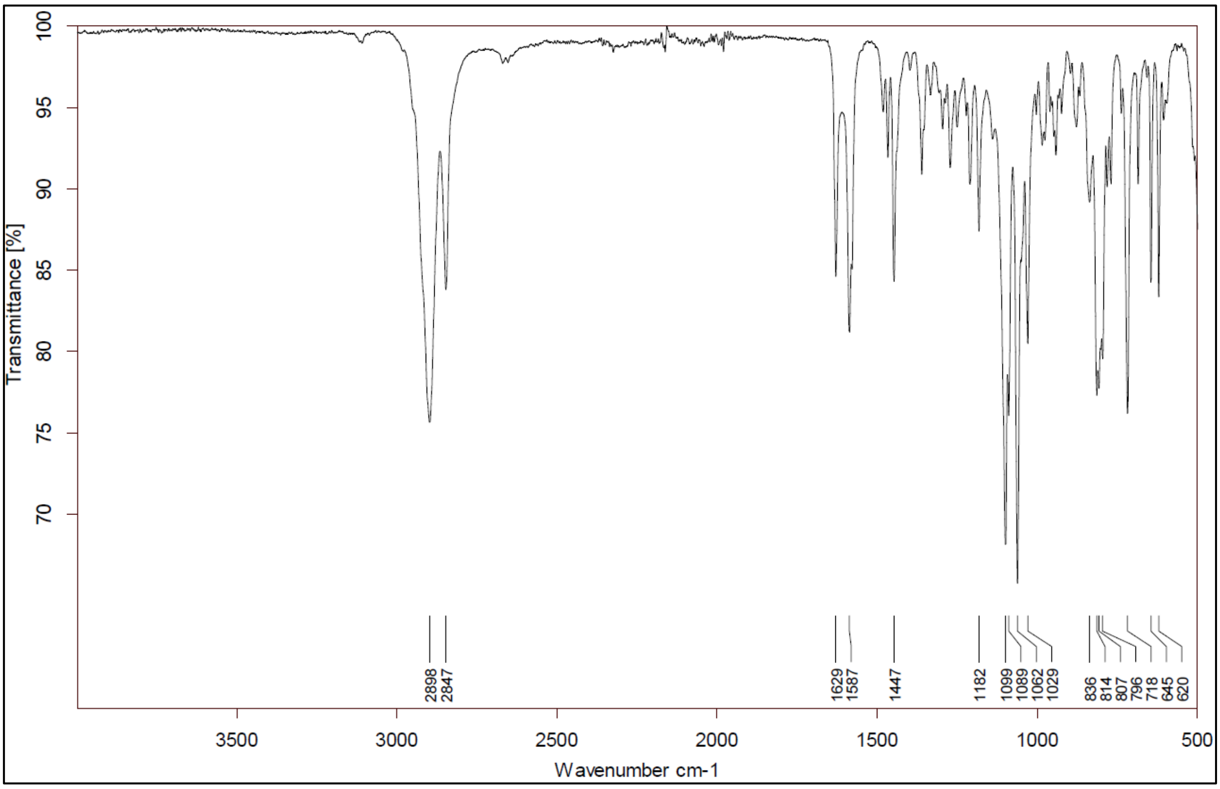

Figure S86: IR (ATR) spectrum of **4l/4l'**.

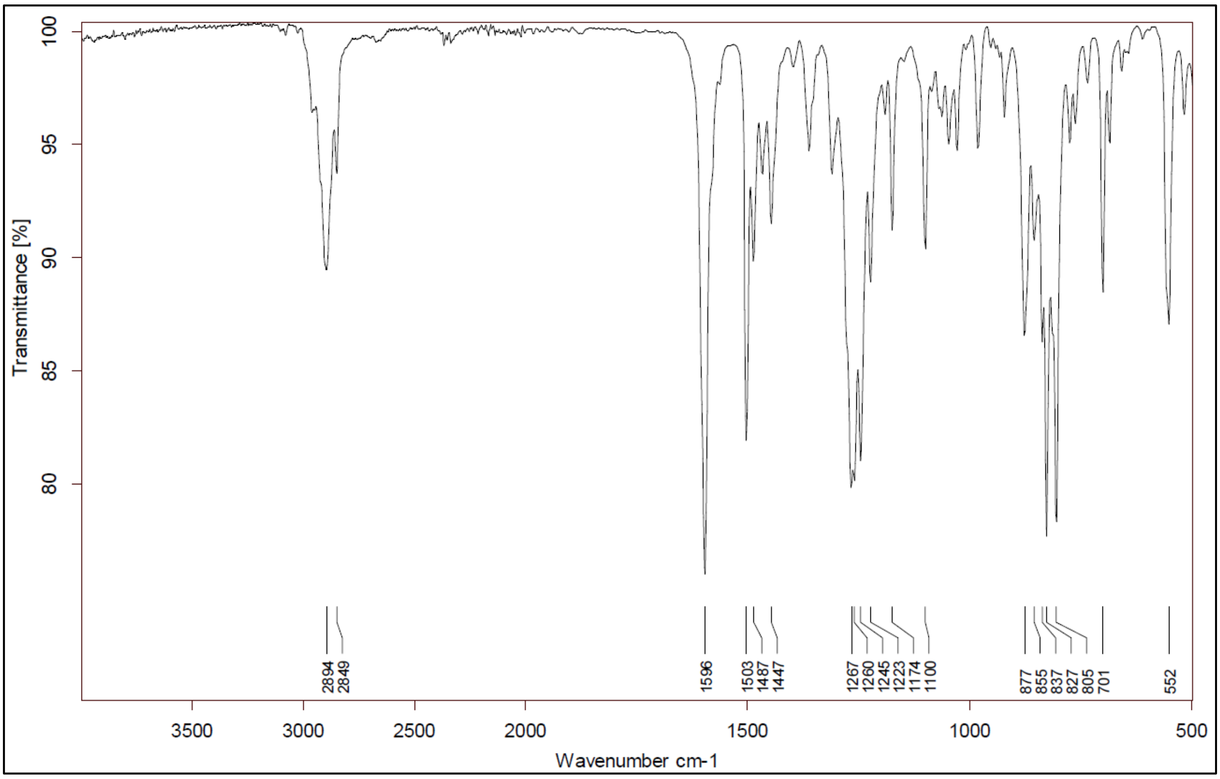

Figure S87: IR (ATR) spectrum of **4m/4m’**.

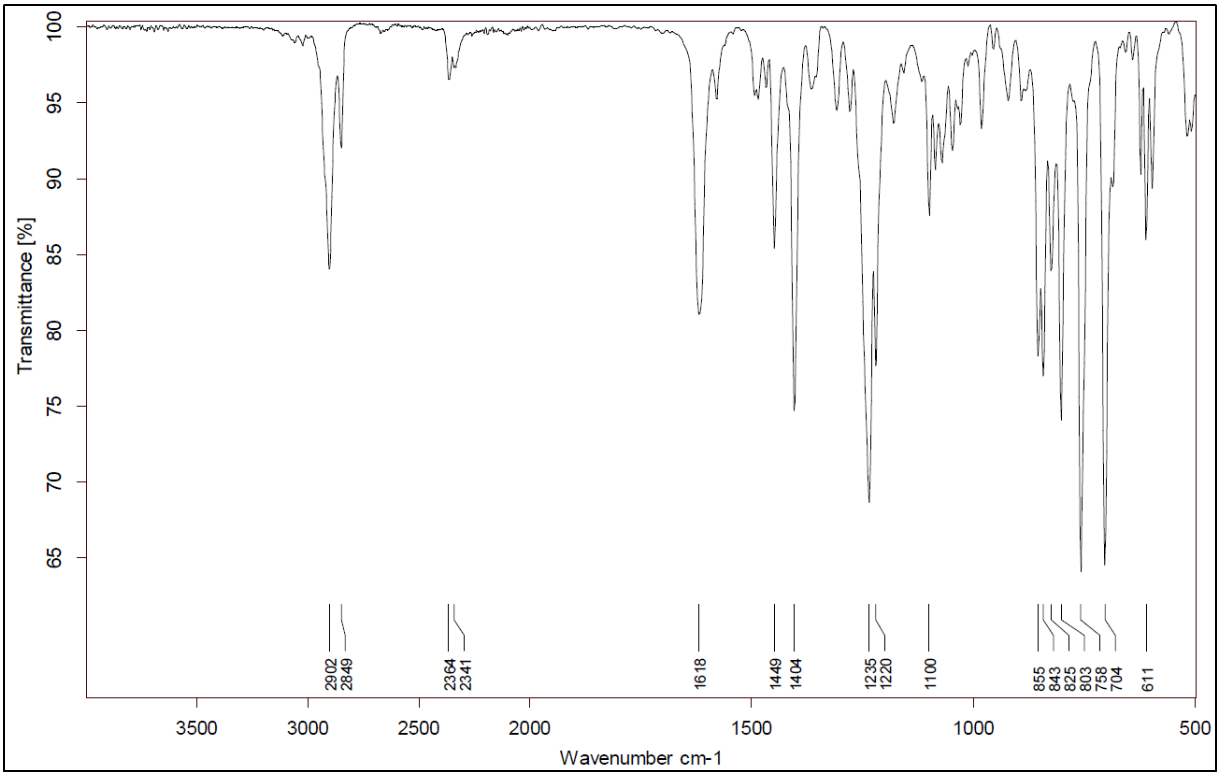

Figure S88: IR (ATR) spectrum of **4n/4n’**.

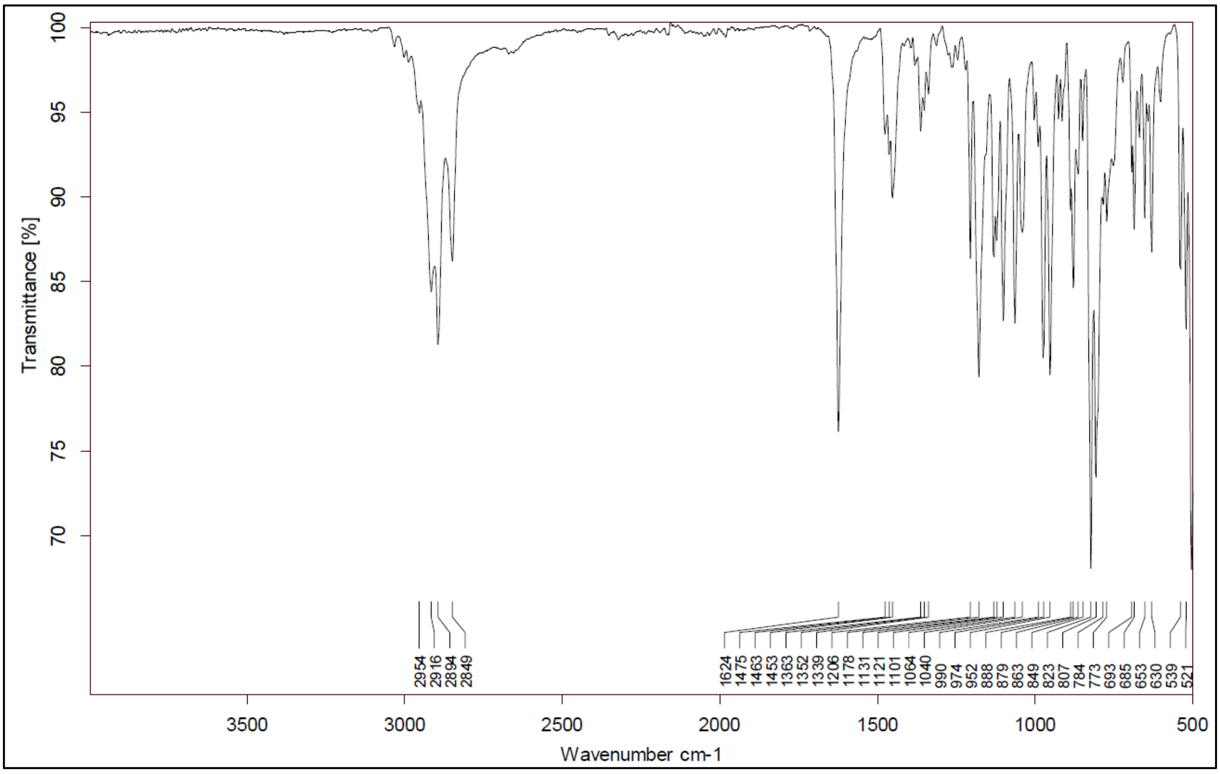

Figure S89: IR (ATR) spectrum of **5o/5o'**.

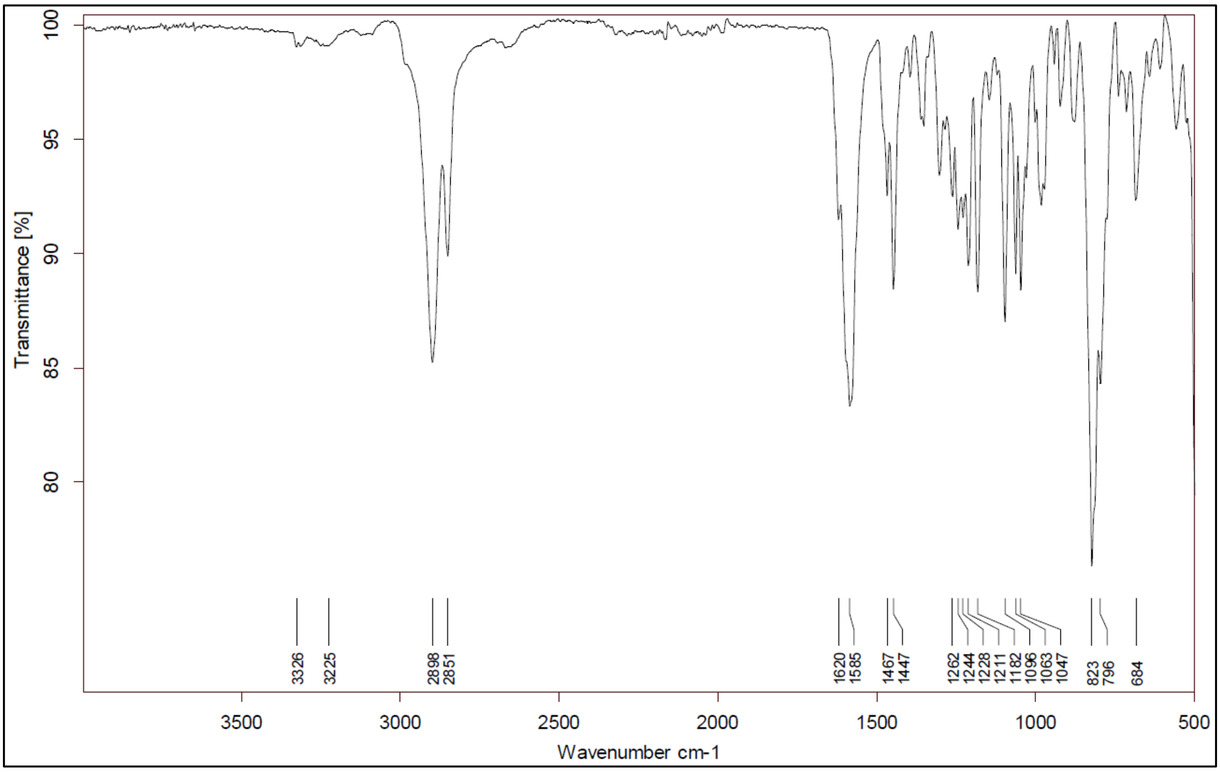

Figure S90: IR (ATR) spectrum of **5p/5p'**.

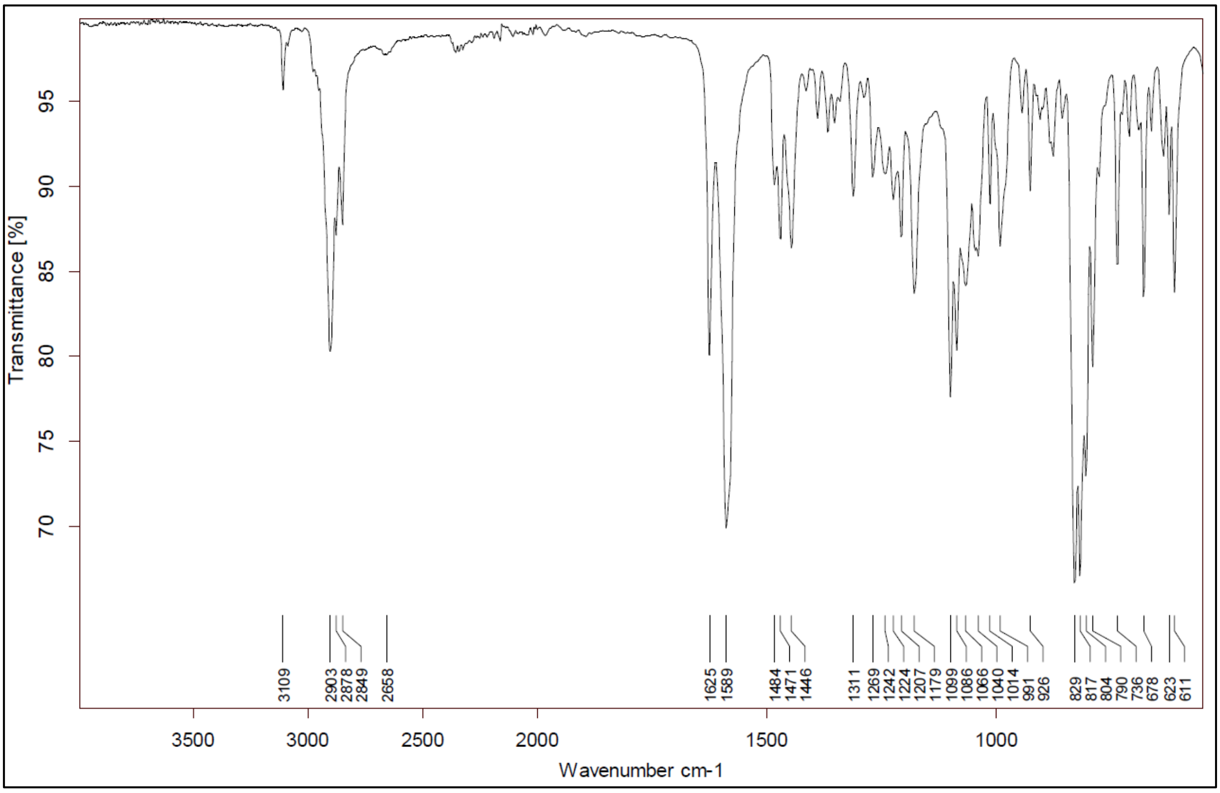

Figure S91: IR (ATR) spectrum of **5q/5q'**.

UV/vis Spectra

UV/vis spectra were recorded with a visible amount of sample dissolved in *n*-hexane at room temperature. An exact concentration was not determined.

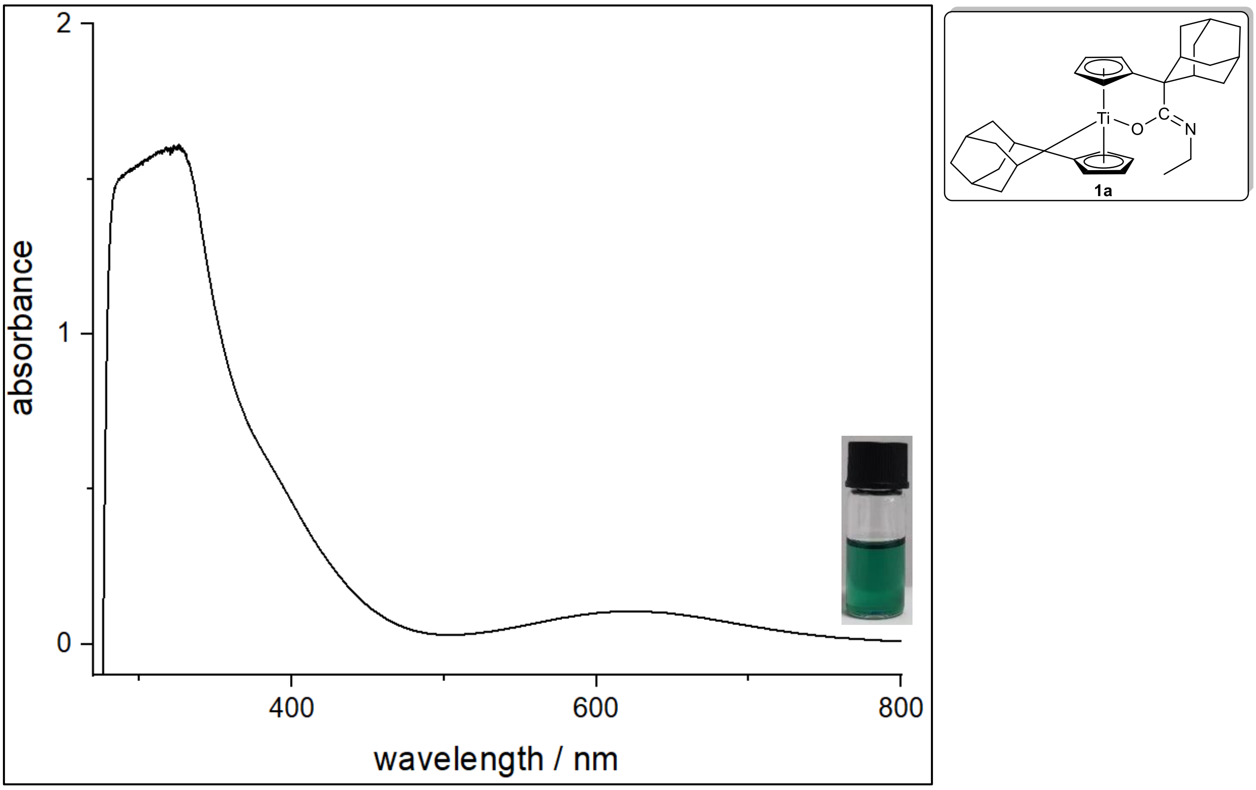

Figure S92: UV/vis spectrum of **1a**.

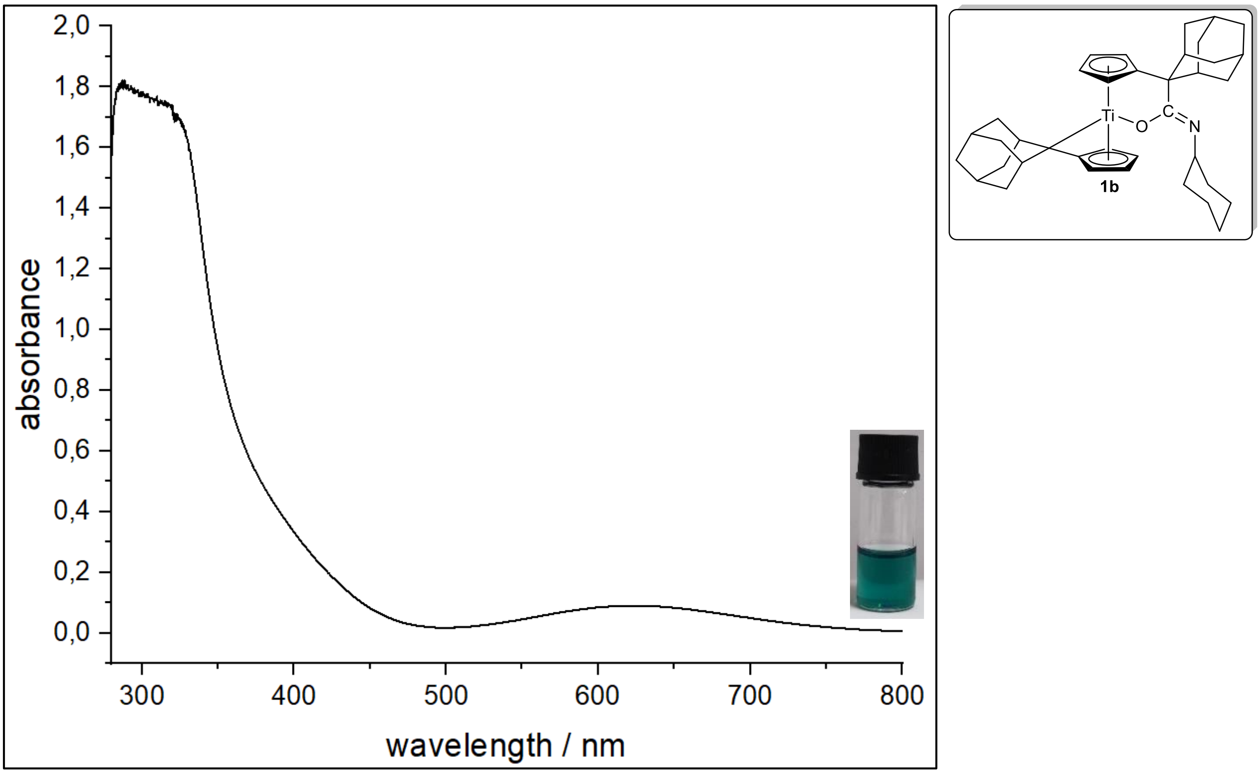

Figure S93: UV/vis spectrum of **1b**.

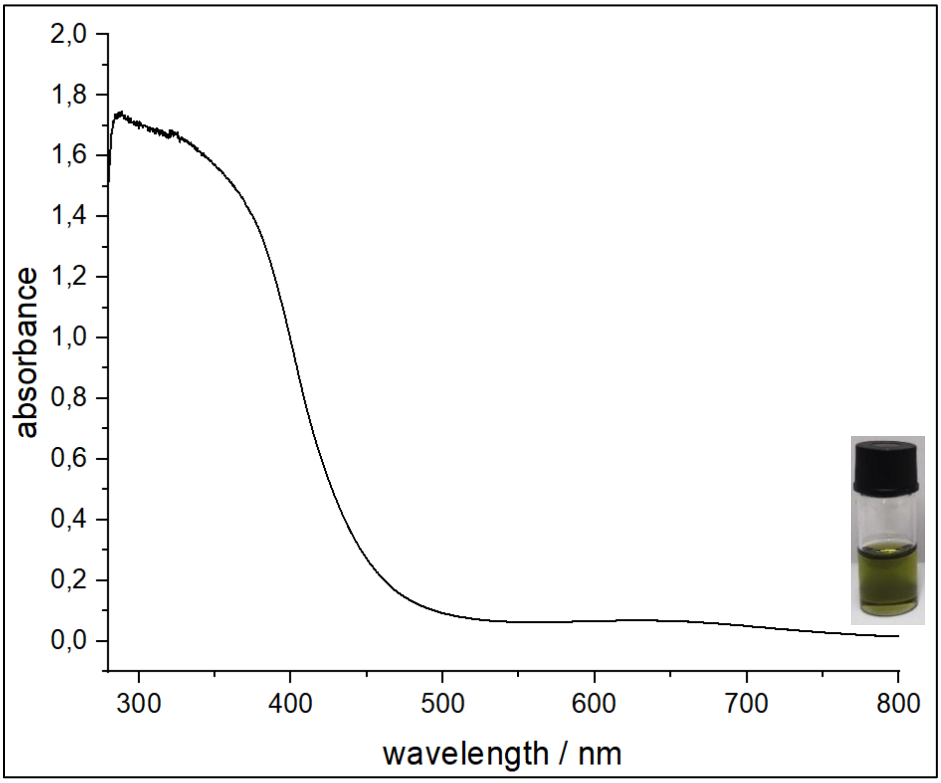

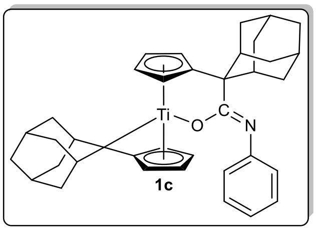

Figure S94: UV/vis spectrum of **1c**.

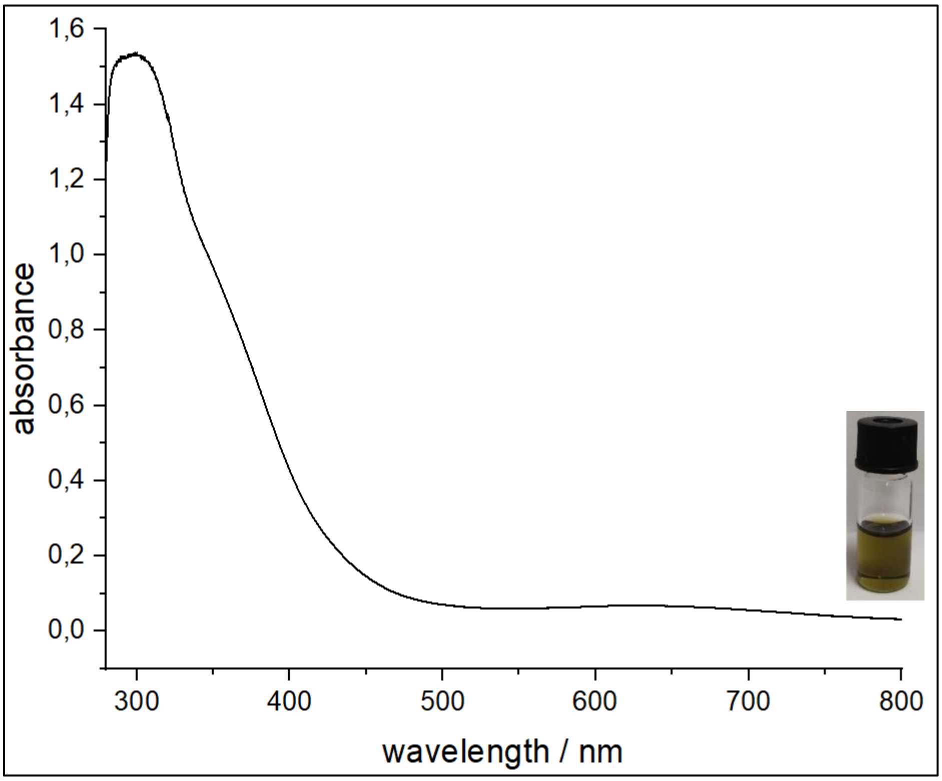

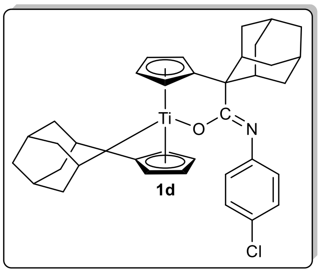

Figure S95: UV/vis spectrum of **1d**.

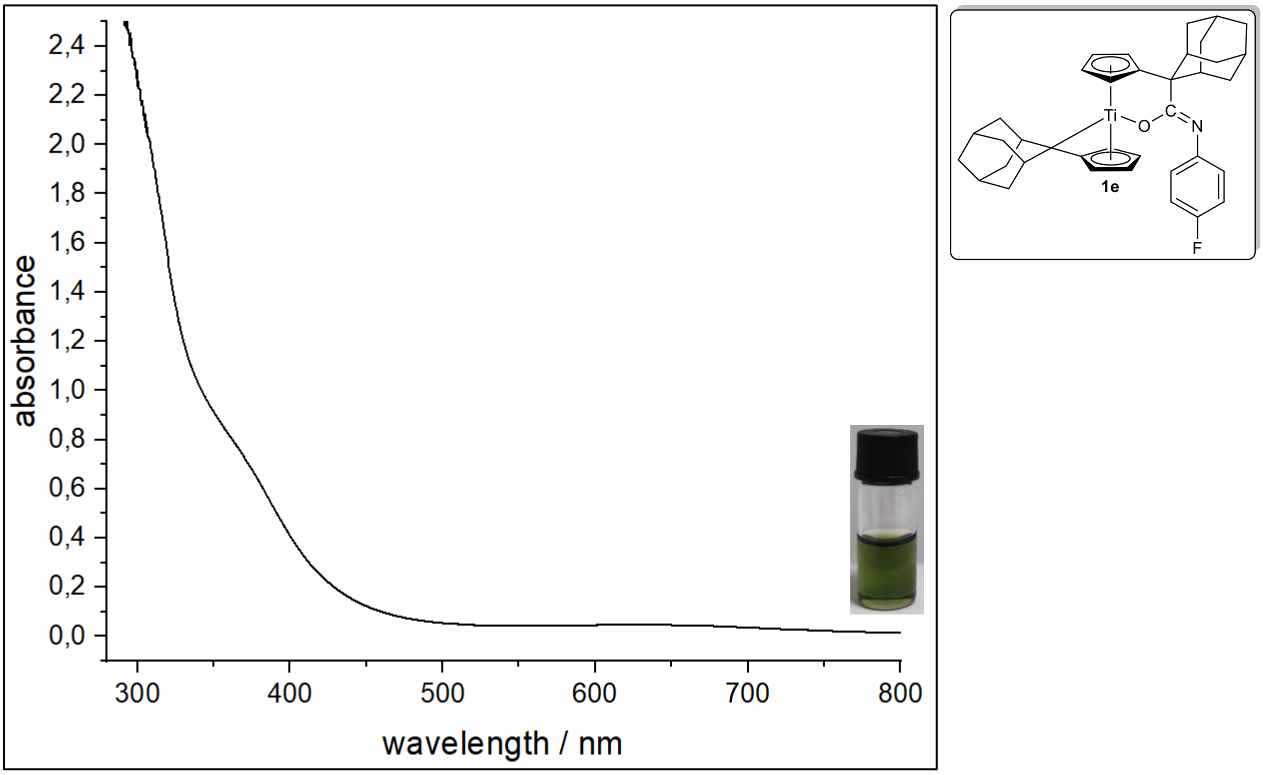

Figure S96: UV/vis spectrum of **1e**.

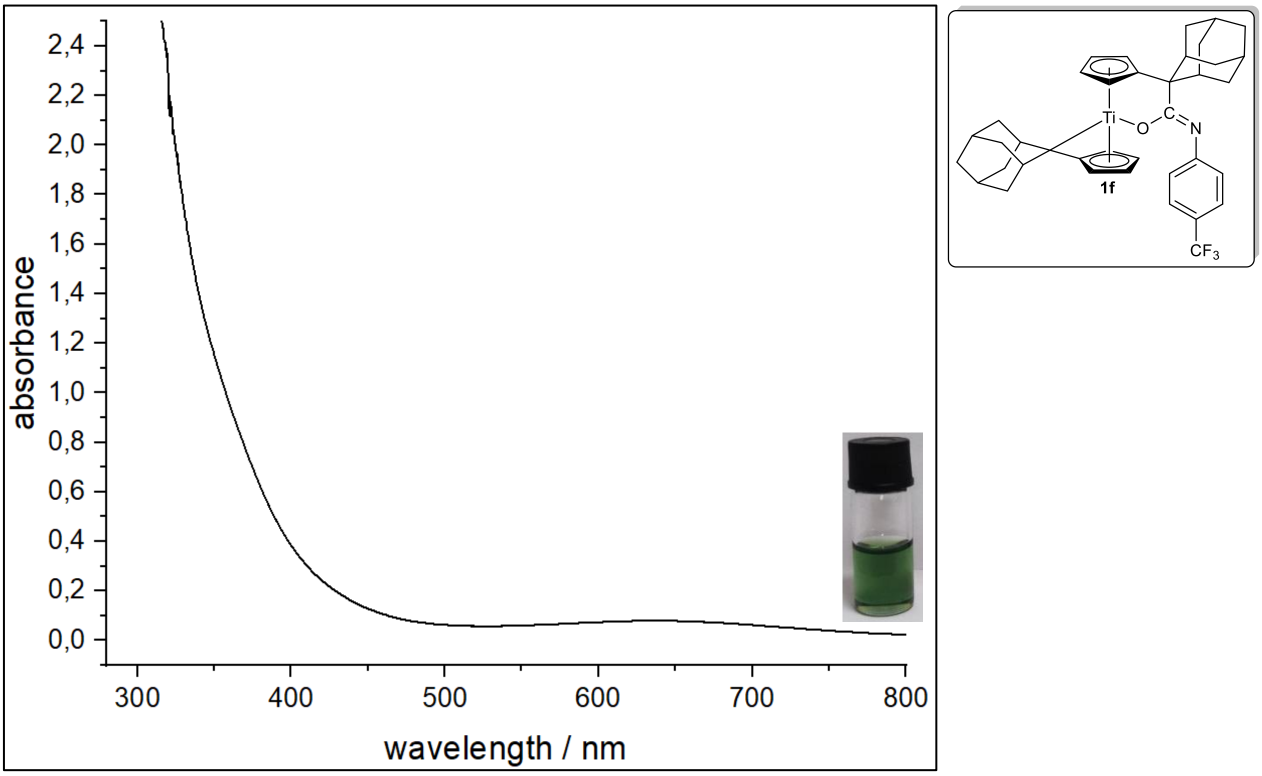

Figure S97: UV/vis spectrum of **1f**.

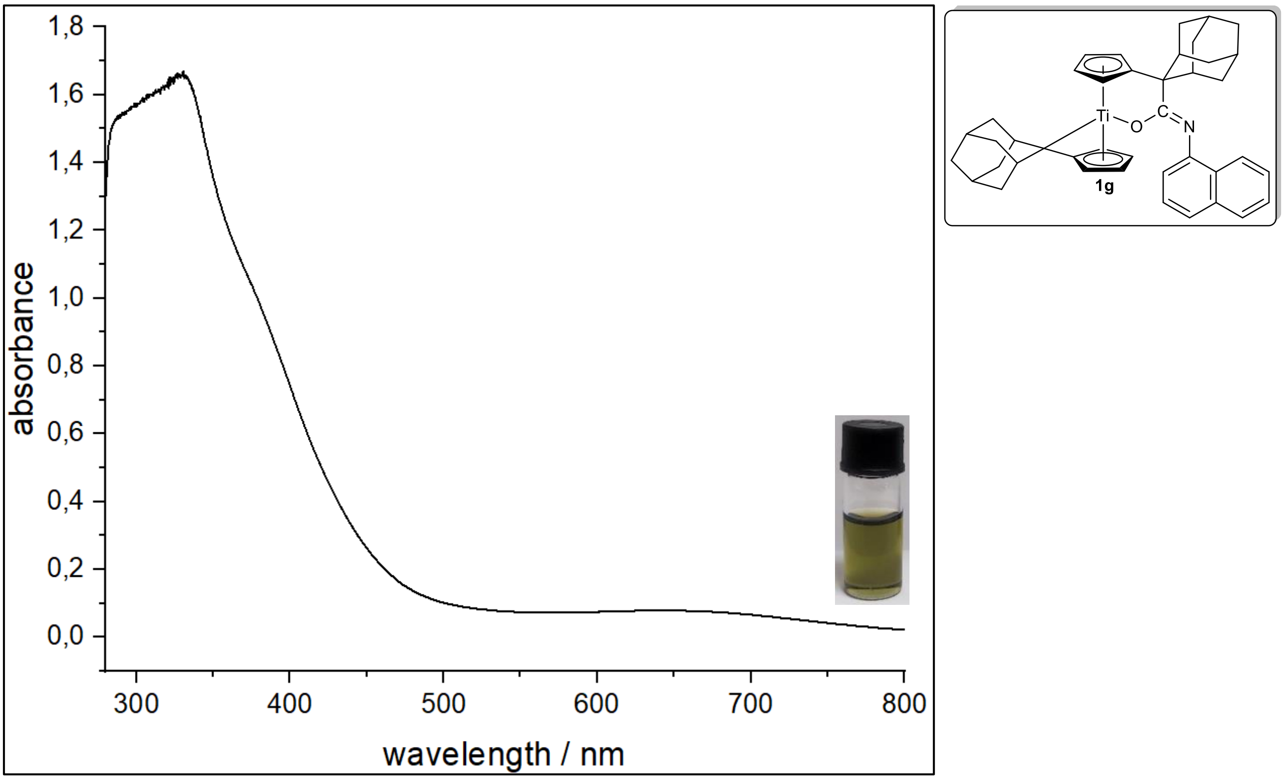

Figure S98: UV/vis spectrum of **1g**.

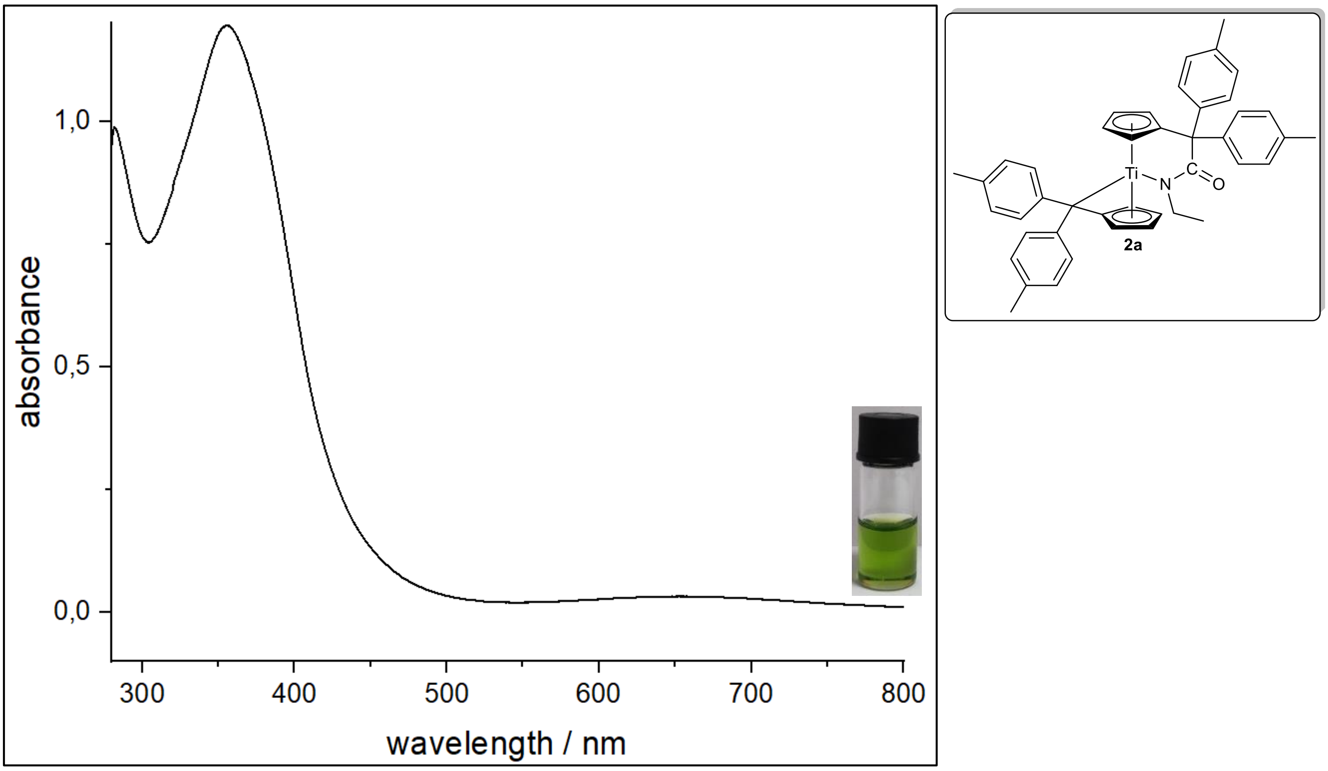

Figure S99: UV/vis spectrum of **2a**.

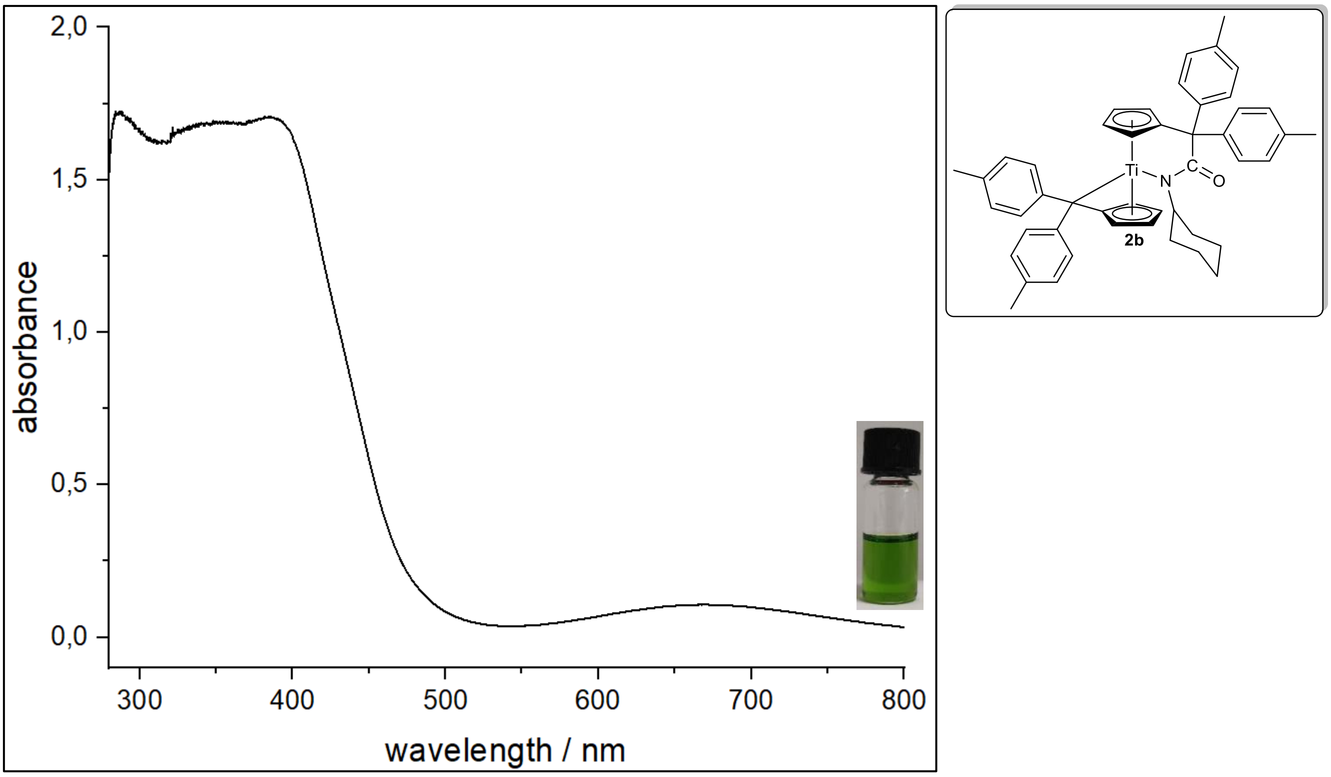

Figure S100: UV/vis spectrum of **2b**.

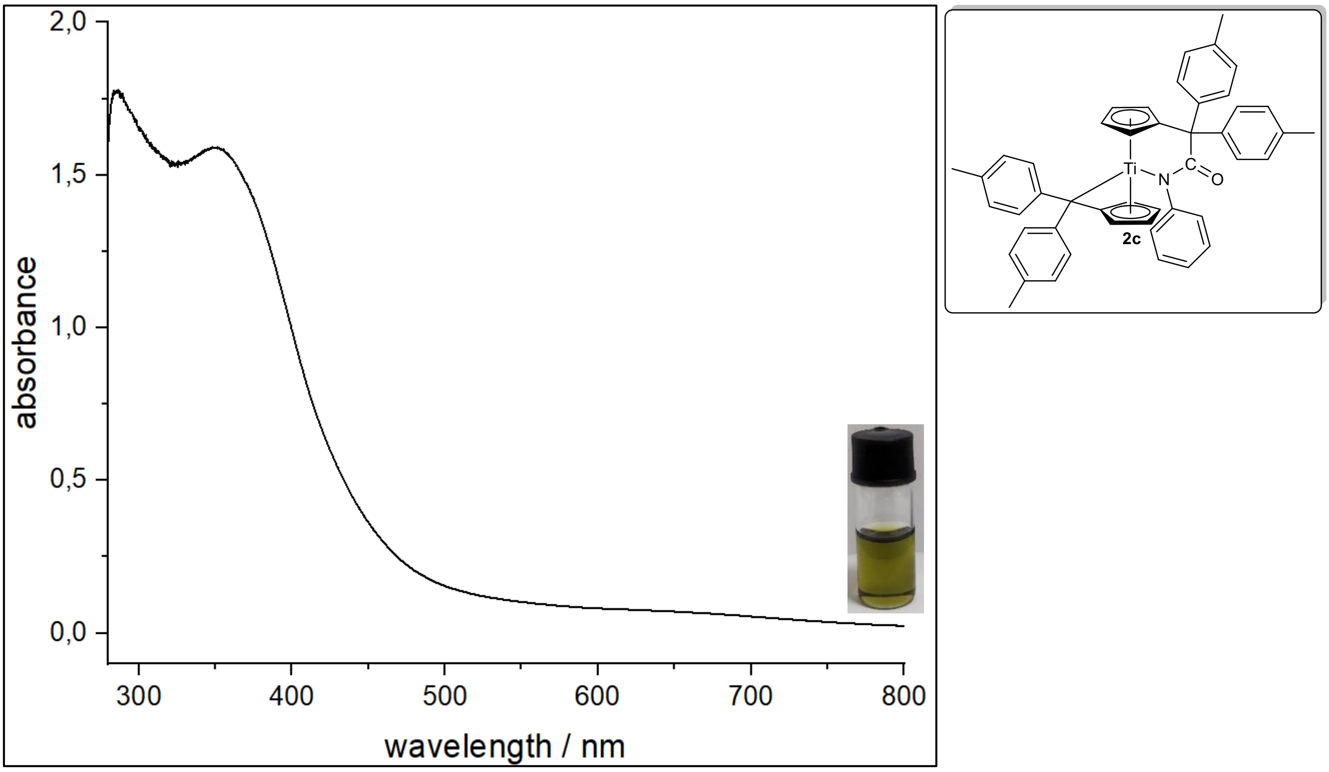

Figure S101: UV/vis spectrum of **2c**.

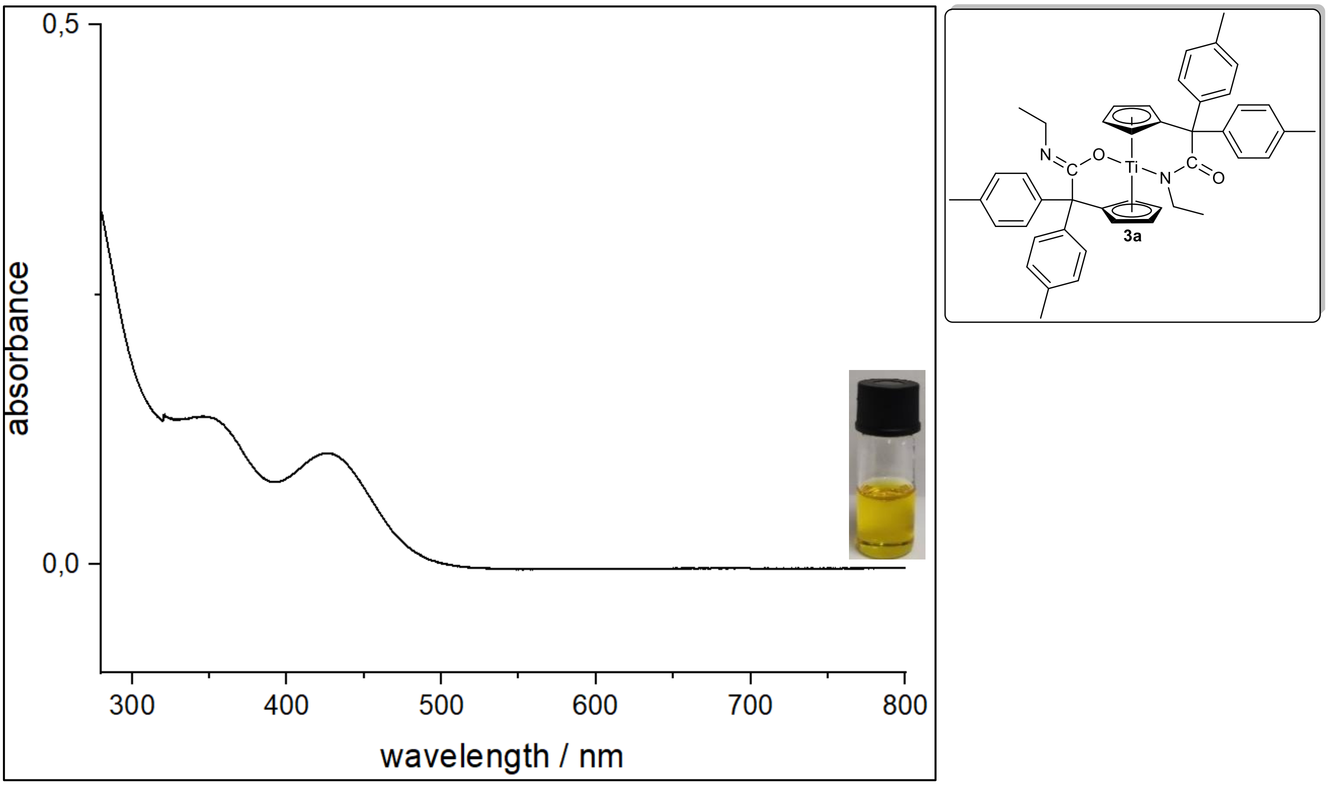

Figure S102: UV/vis spectrum of **3a**.

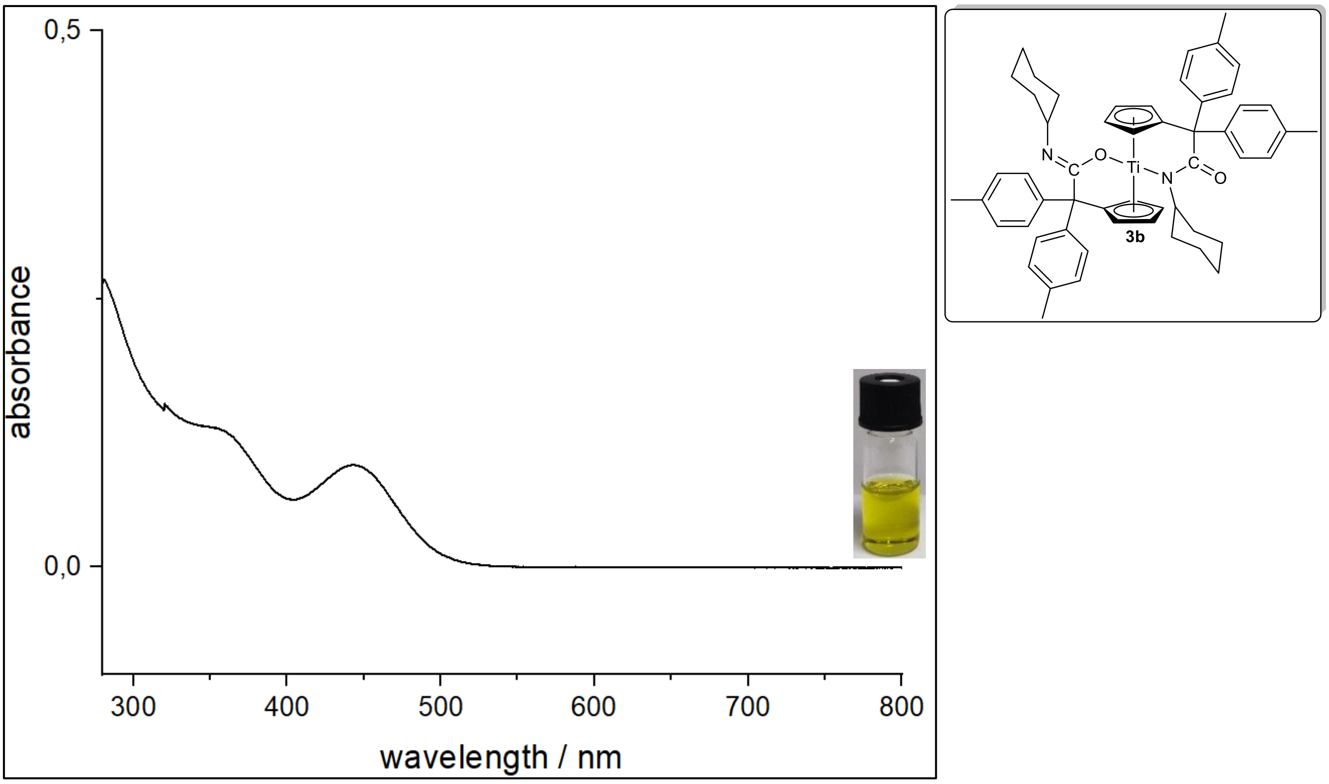

Figure S103: UV/vis spectrum of **3b**.

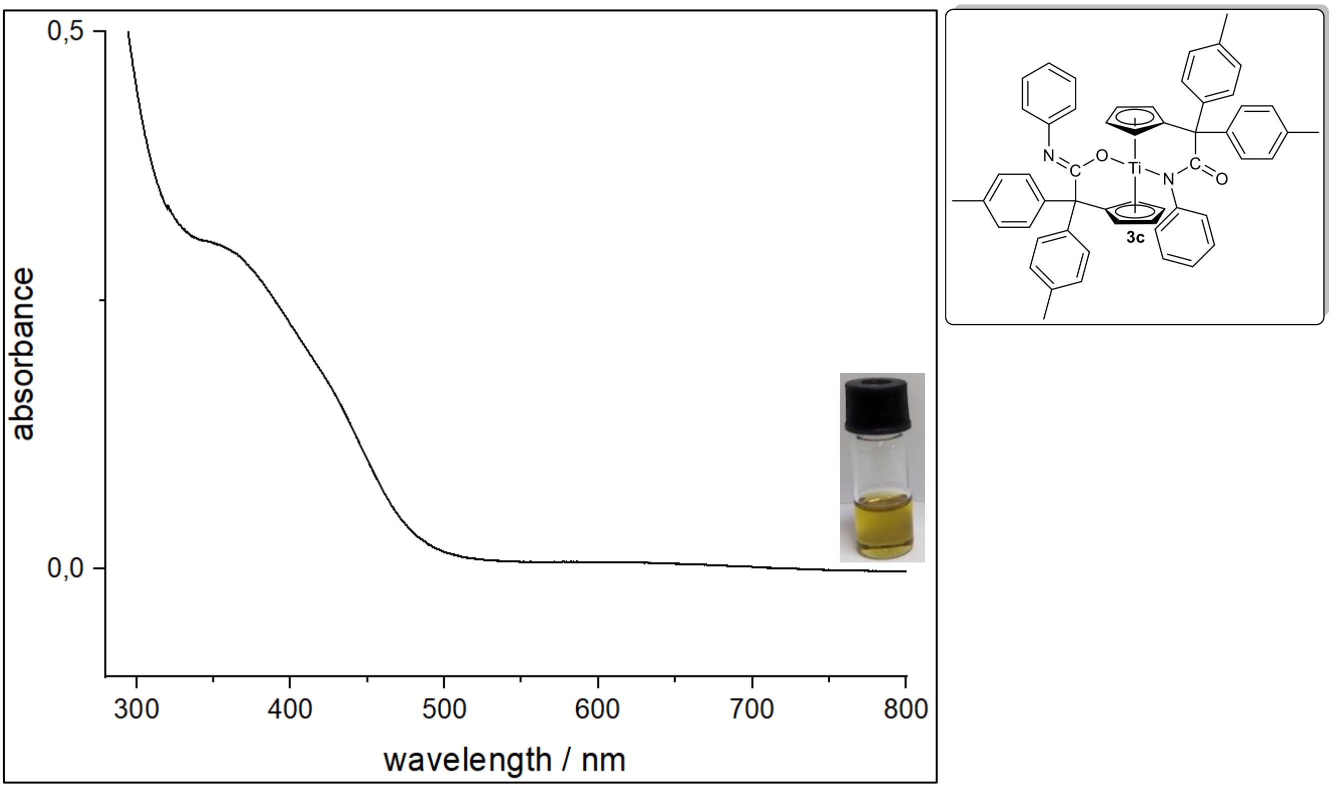

Figure S104: UV/vis spectrum of **3c**.

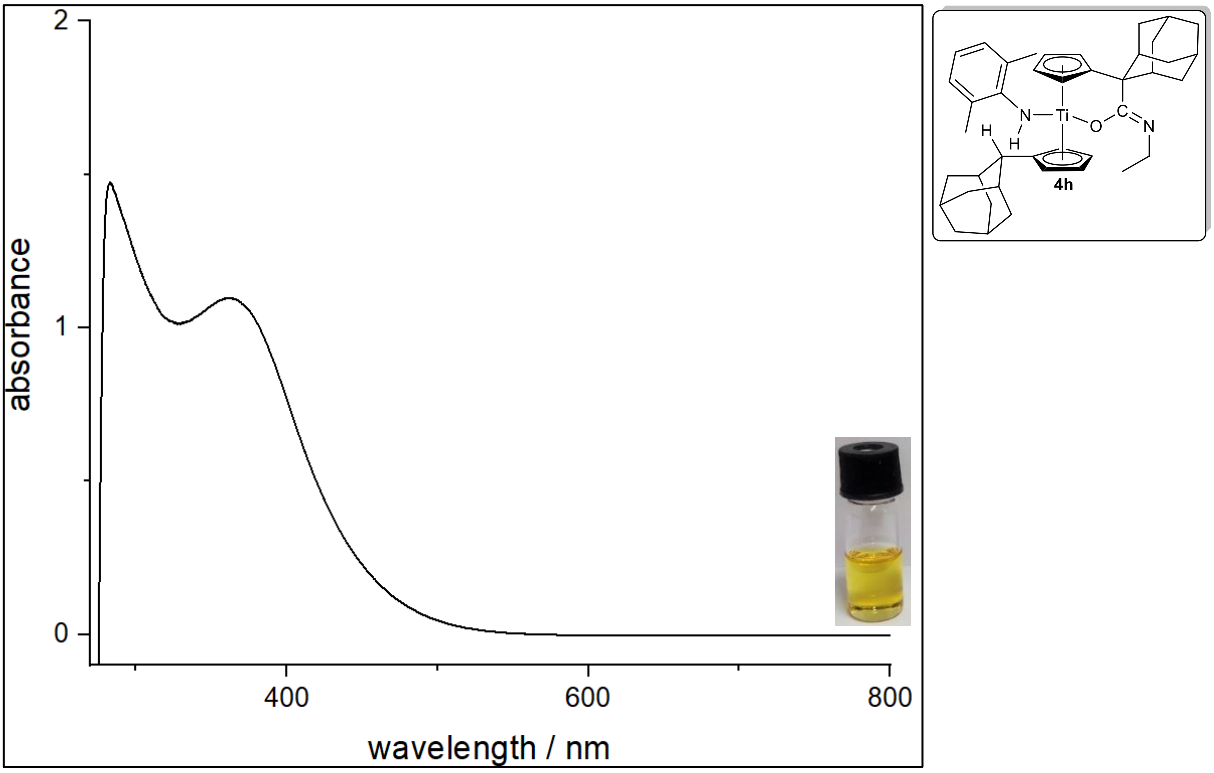

Figure S105: UV/vis spectrum of **4h**.

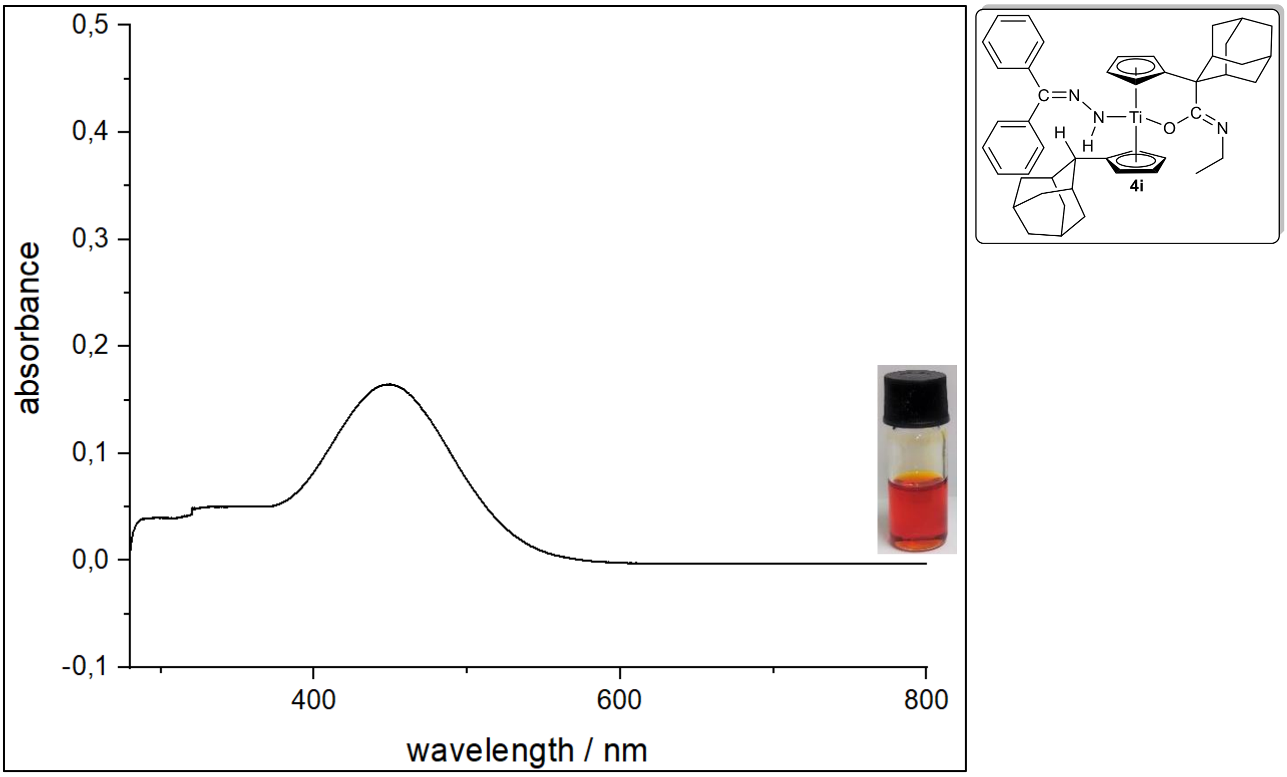

Figure S106: UV/vis spectrum of **4i**.

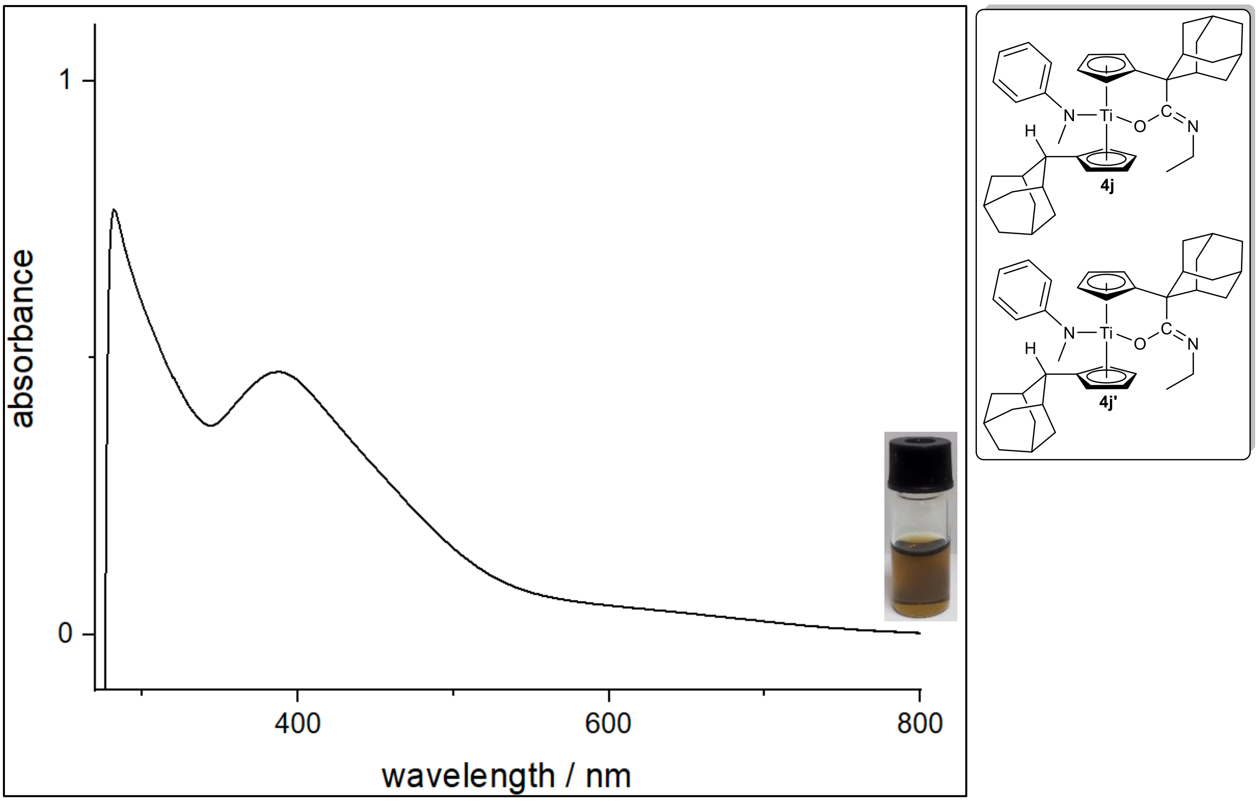

Figure S107: UV/vis spectrum of **4j/4j'**.

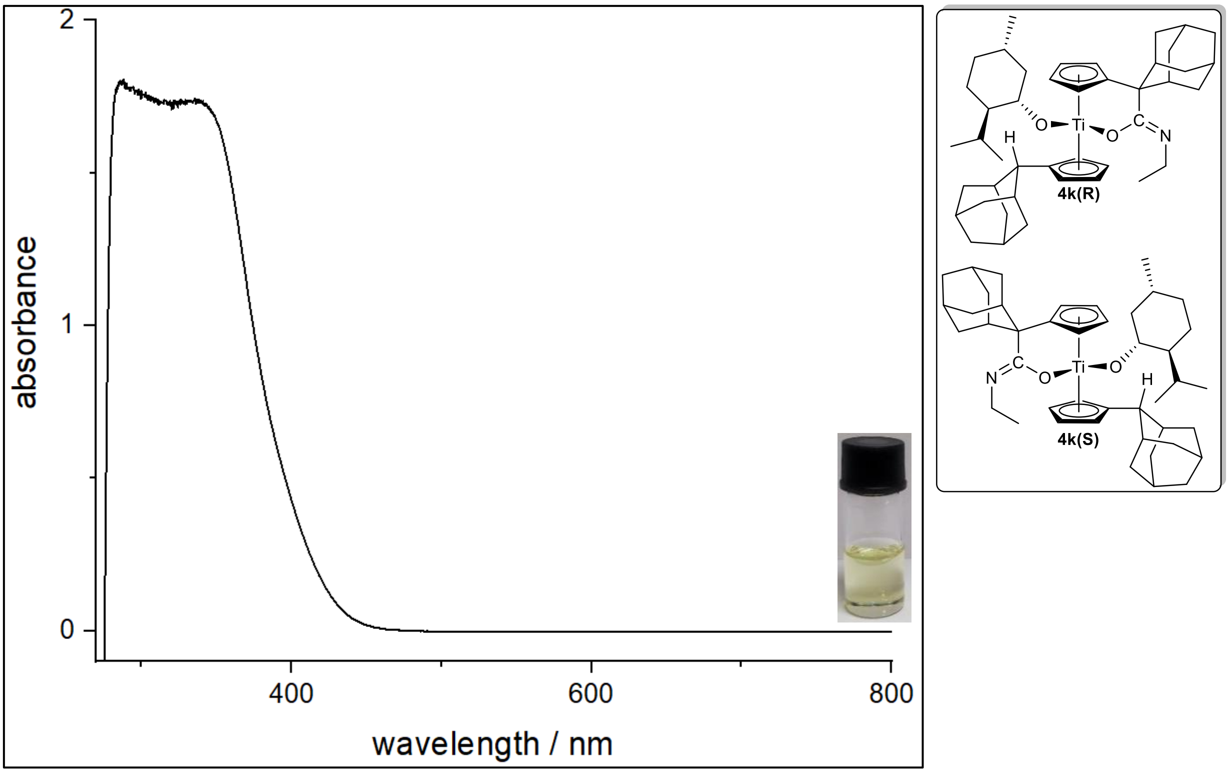

Figure S108: UV/vis spectrum of **4k(R)/4k (S)**.

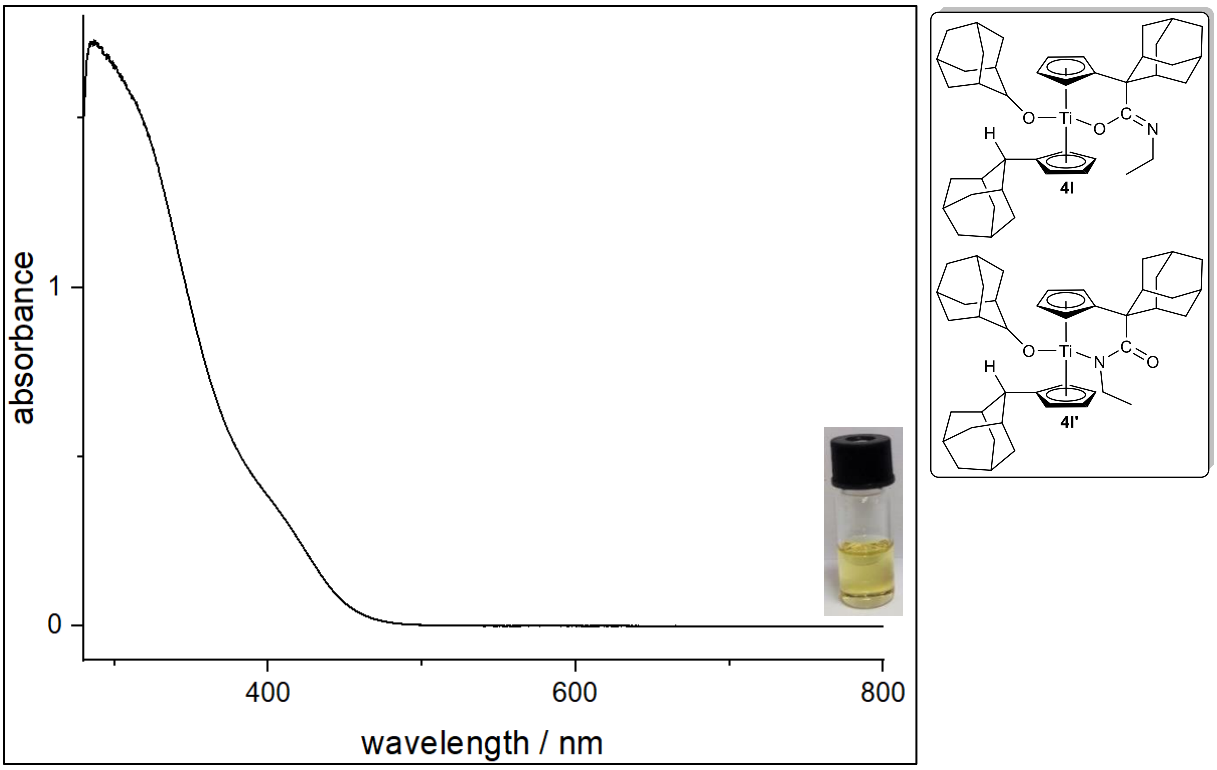

Figure S109: UV/vis spectrum of **4l/4l'**.

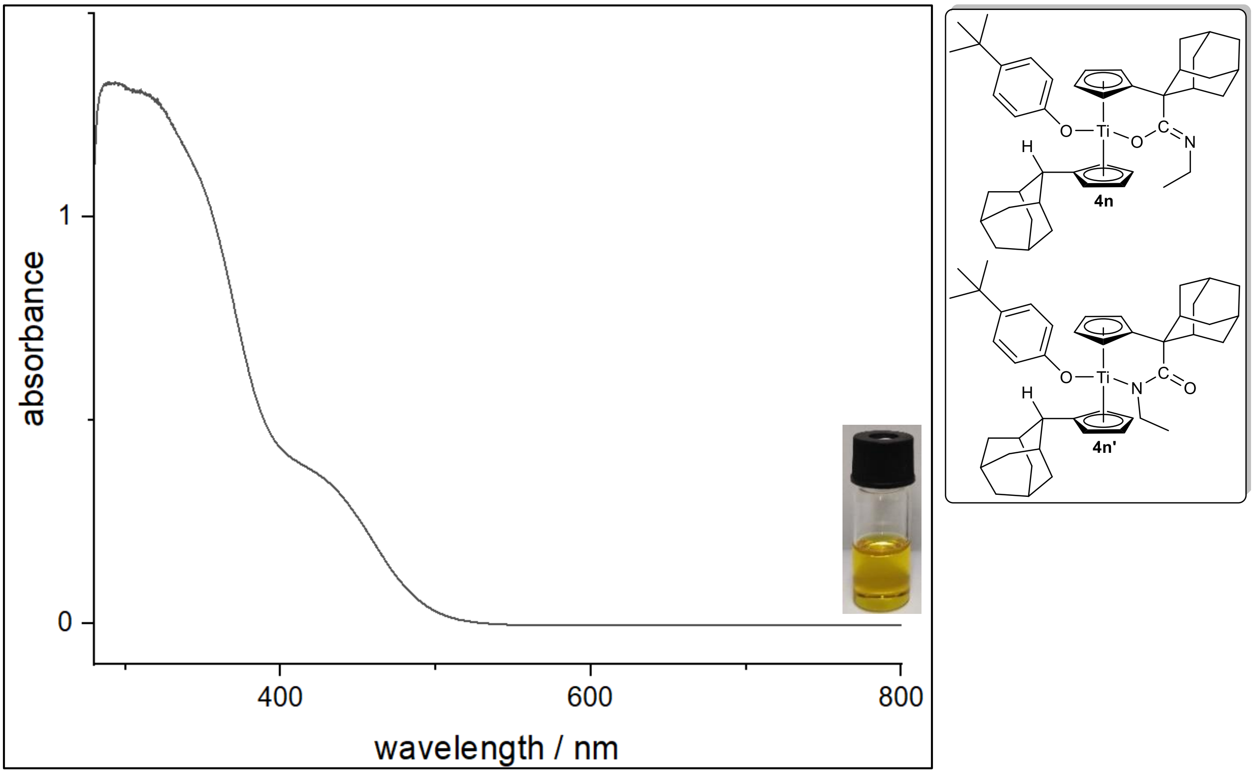

Figure S110: UV/vis spectrum of **4m/4m'**.

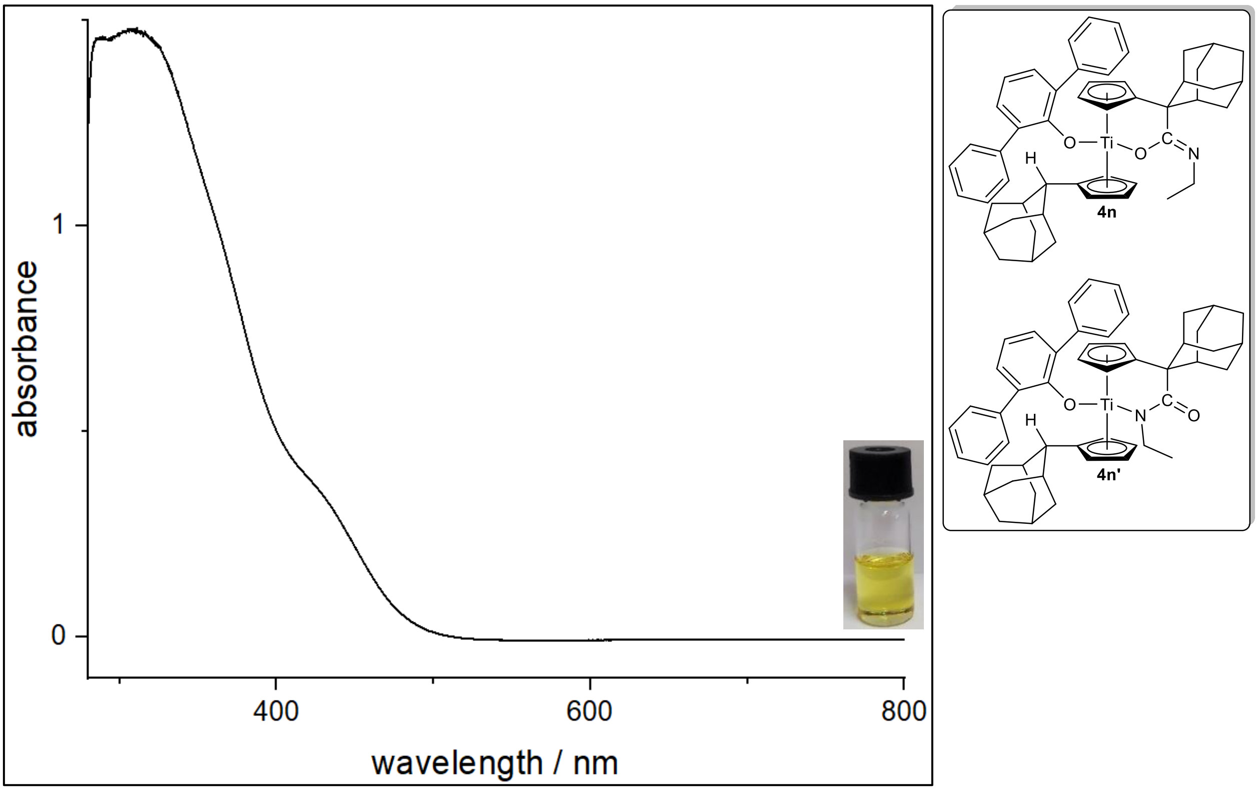

Figure S111: UV/vis spectrum of **4n/4n'**.

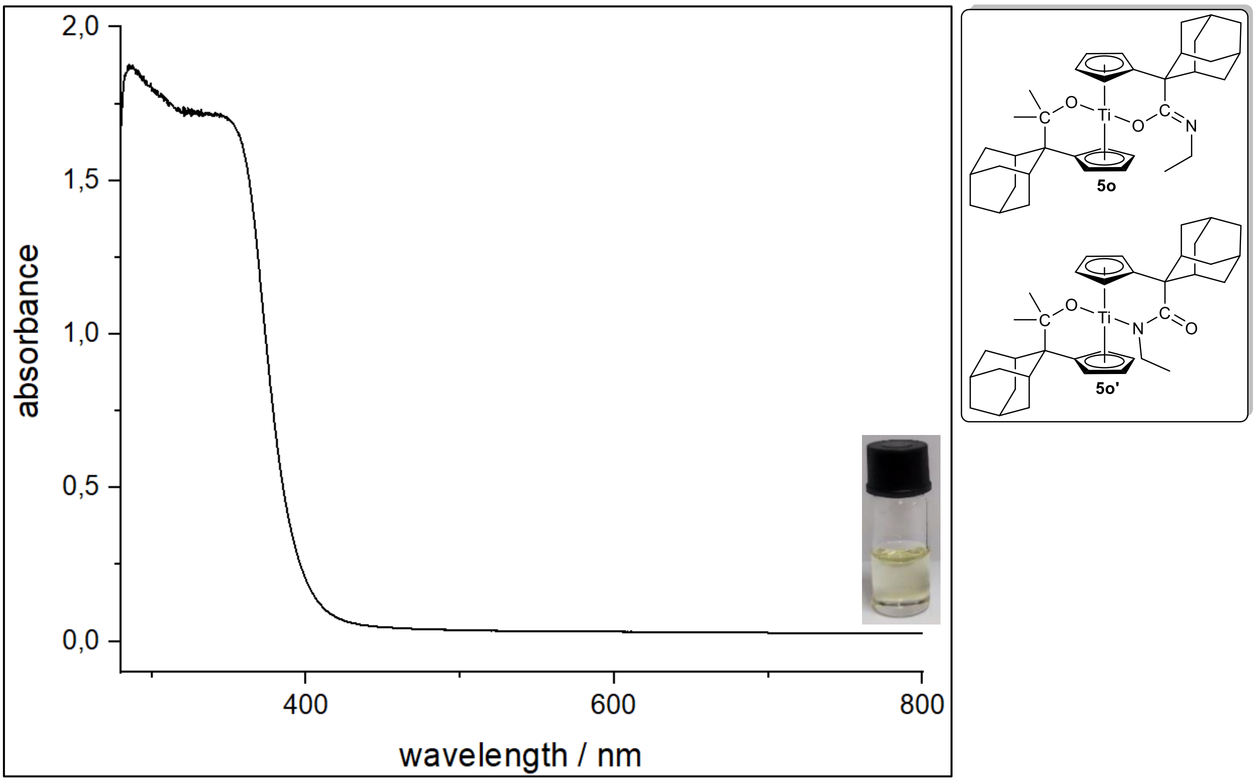

Figure S112: UV/vis spectrum of **5o/5o'**.

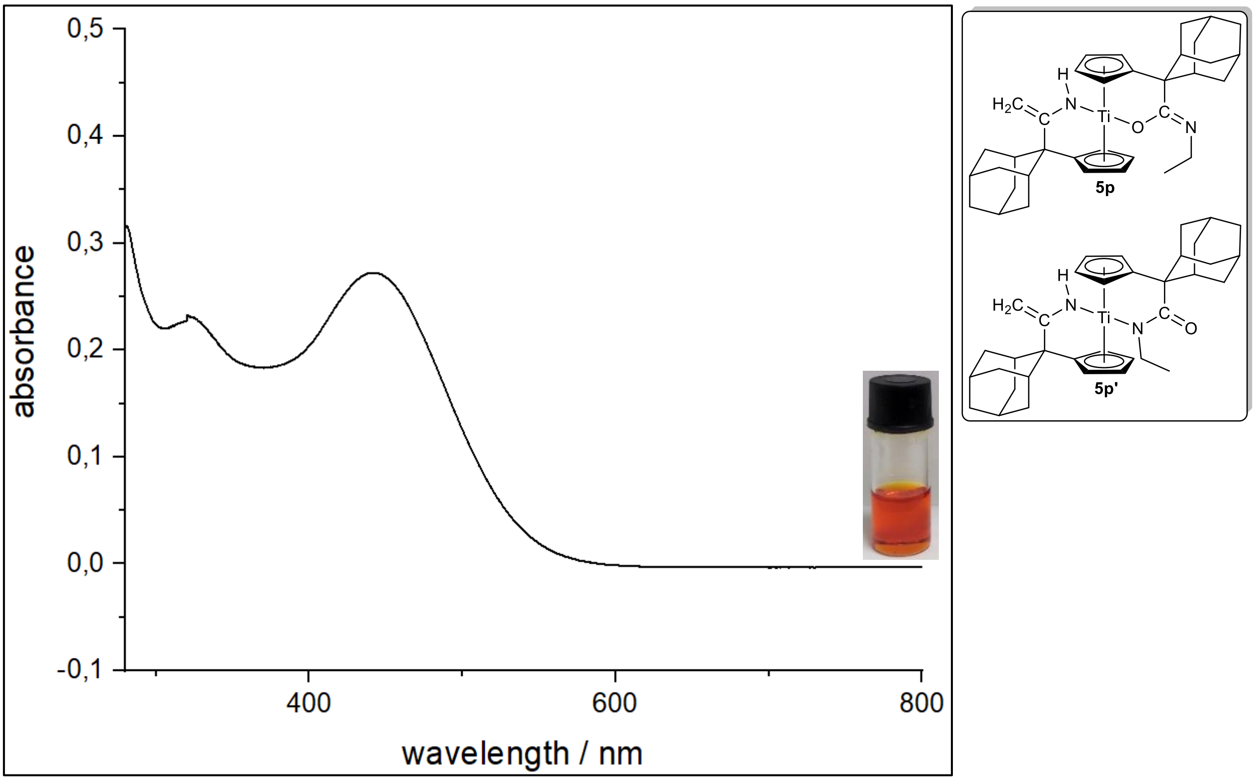

Figure S113: UV/vis spectrum of **5p/5p'**.

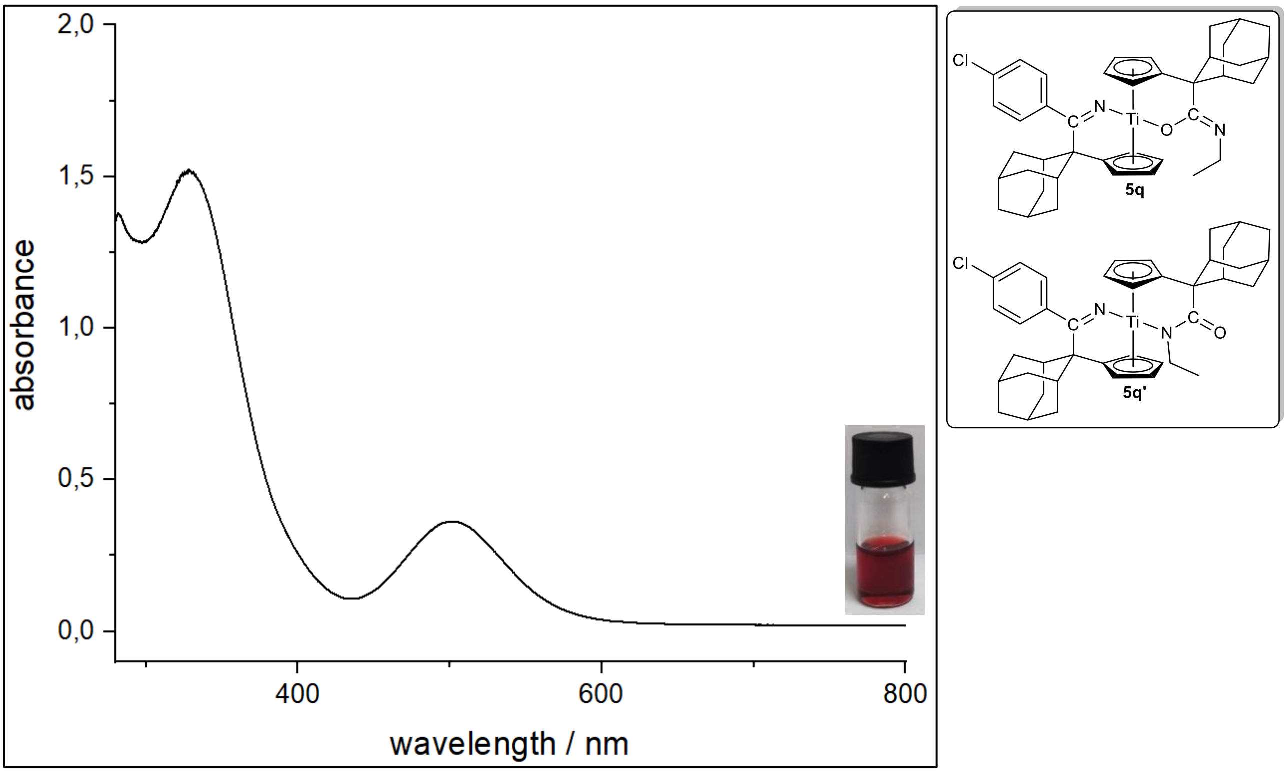

Figure S114: UV/vis spectrum of **5q/5q'**.

Crystallographic Data

Single crystal X-ray data were measured on a Bruker AXS D8 Venture diffractometer (multilayer optics, Mo-Kα and Cu-Kα radiation with λ = 0.71073 Å and 1.54178 Å respectively, Kappa 4-circle goniometer, Photon III C14 CPAD detector). All crystals were measured at a temperature of 100 K. Absorption corrections using equivalent reflections were performed with the program SADABS.[3] The crystal of **1d** was a non-merohedral twin, for which the absorption correction was performed with the program TWINABS.[4] All structures were solved with the program SHELXS[5] and refined with SHELXL[6] using the OLEX2[7] GUI.

All non-H atoms were refined using anisotropic atomic displacement parameters (ADPs). H atoms bonded to C were located in the difference Fourier maps and placed on idealized geometric positions with idealized atomic displacement parameters using the riding model. H atoms bonded to N were refined freely.

3c and **4h** contain disordered solvent molecules, some of which were refined using isotropic ADPs or restraints on the anisotropic ADPs (RIGU instruction within SHELXL), as well as constraints on the geometry (AFIX 66) and isotropic ADPs (EADP) of one toluene phenyl group in **4h**.

In **4h**, **4k**, **4m**, and **5p** the inserted isocyanate groups are disordered. Here, the minor sites were refined using isotropic ADPs.

4l and **5o'** exhibit whole molecule disorder, where the minor sites (sof = 0.106 and 0.161 respectively) were restrained to have the same geometry as the respective major sites (SAME) with isotropic ADPs constrained to be the same (EADP).

The crystallographic data can be obtained free of charge from https://www.ccdc.cam.ac.uk/structures/ quoting the CCDC numbers 2422767-2422786.

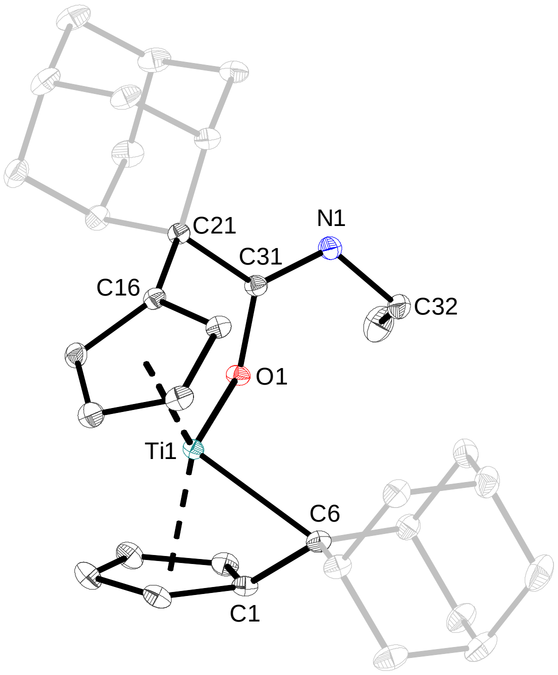

Figure S115: Molecular structure of **1a**. Thermal ellipsoids drawn at the 50% probability level. Hydrogen atoms and co-crystallized toluene are omitted for clarity. Selected bond lengths [Å] and angles [°]: Ti1–O1 1.9350(4), Ti1–C6 2.3714(5), O1–C31 1.3516(6), C1–C6 1.4420(7), C16–C21 1.5229(7), C21–C31 1.5439(7), C31–N1 1.2752(7), C32–N1 1.4662(7); O1–Ti1–C6 104.649(18), Ti1–O1–C31 122.59(3), C16–C21–C31 101.91(4), O1–C31–C21 112.68(4), O1–C31–N1 123.90(5), C21–C31–N1 123.39(4), C31–N1–C32 117.32(5).

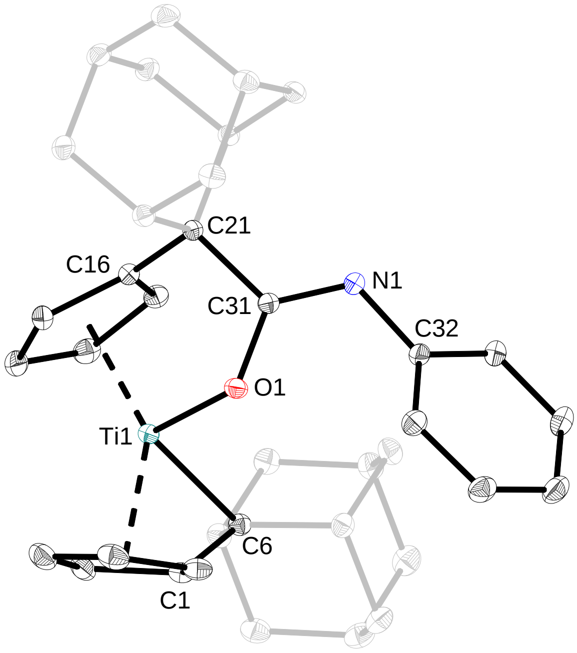

Figure S116: Molecular structure of **1c**. Thermal ellipsoids drawn at the 50% probability level. Hydrogen atoms and solvent molecules are omitted for clarity. Selected bond lengths [Å] and angles [°]: Ti1–O1 1.9343(4), Ti1–C6 2.3756(5), C1–C6 1.4422(7), C16–C21 1.5258(6), O1–C31 1.3327(6), C21–C31 1.5414(6), C31–N1 1.2845(6), C32–N1 1.4124(6); O1–Ti1–C6 103.372(16), Ti1–O1–C31 121.76(3), O1–C31–C21 113.14(4), O1–C31–N1 123.77(4), C21–C31–N1 123.04(4), C31–N1–C32 119.57(4).

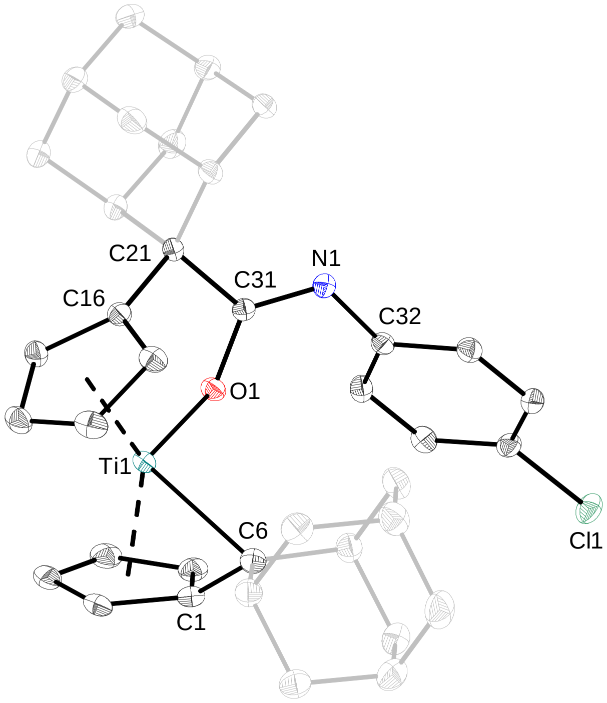

Figure S117: Molecular structure of **1d**. Thermal ellipsoids drawn at the 50% probability level. Hydrogen atoms and additional crystallographic independent molecules are omitted for clarity. Selected bond lengths [Å] and angles [°]: Ti1–O1 1.9245(10), Ti1–C6 2.4029(15), C1–C6 1.4387(19), C16–C21 1.5238(19), O1–C31 1.3338(16), C21–C31 1.5411(18), C31–N1 1.2814(16), C32–N1 1.4123(18); O1–Ti1–C6 102.69(5), Ti1–O1–C31 123.24(8), O1–C31–C21 113.41(4), O1–C31–N1 123.09(12), C21–C31–N1 123.50(12), C31–N1–C32 119.34(11).

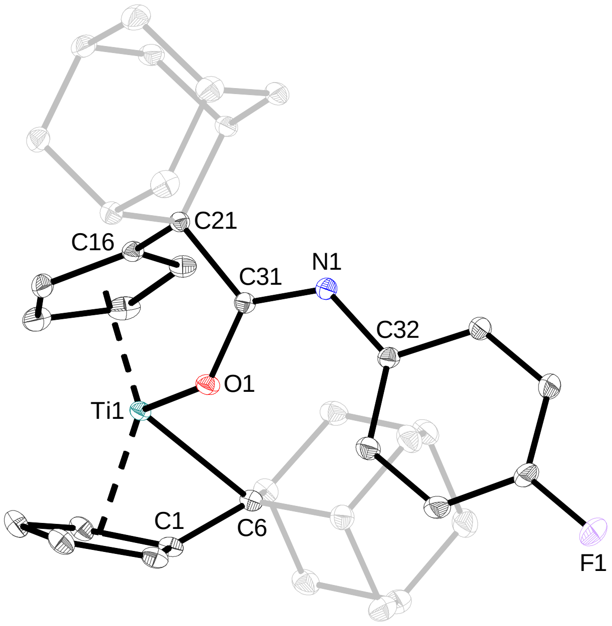

Figure S118: Molecular structure of **1e**. Thermal ellipsoids drawn at the 50% probability level. Hydrogen atoms are omitted for clarity. Selected bond lengths [Å] and angles [°]: Ti1–O1 1.9437(4), Ti1–C6 2.3976(6), C1–C6 1.4373(8), C16–C21 1.5212(8), O1–C31 1.3381(7), C21–C31 1.5436(8), C31–N1 1.2802(7), C32–N1 1.4059(8); O1–Ti1–C6 100.49(2), Ti1–O1–C31 122.54(4), O1–C31–C21 113.45(5), O1–C31–N1 125.71(5), C21–C31–N1 124.91(4), C31–N1–C32 124.91(5).

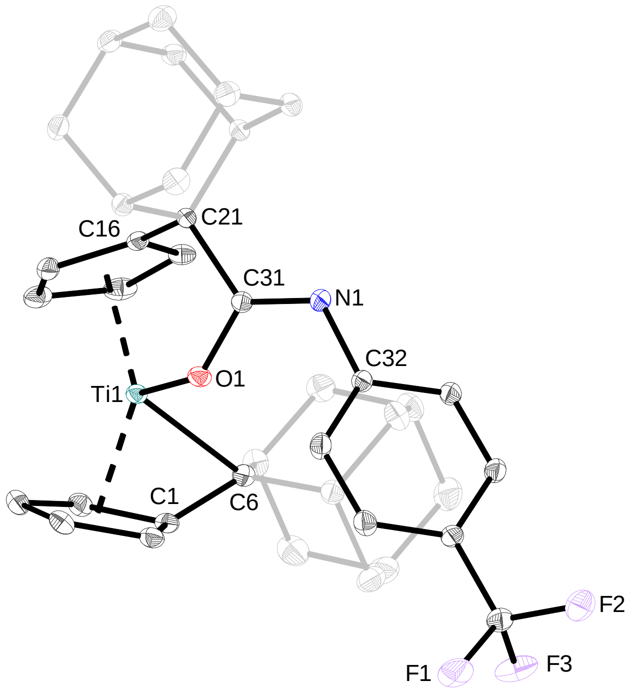

Figure S119: Molecular structure of **1f**. Thermal ellipsoids drawn at the 50% probability level. Hydrogen atoms are omitted for clarity. Selected bond lengths [Å] and angles [°]: Ti1–O1 1.9317(8), Ti1–C6 2.3829(11), C1–C6 1.4403(15), C16–C21 1.5230(14), O1–C31 1.3340(13), C21–C31 1.5420(14), C31–N1 1.2848(13), C32–N1 1.4110(14); O1–Ti1–C6 100.89(4), Ti1–O1–C31 123.58(7), O1–C31–C21 113.17(8), O1–C31–N1 123.36(9), C21–C31–N1 123.47(9), C31–N1–C32 118.89(9).

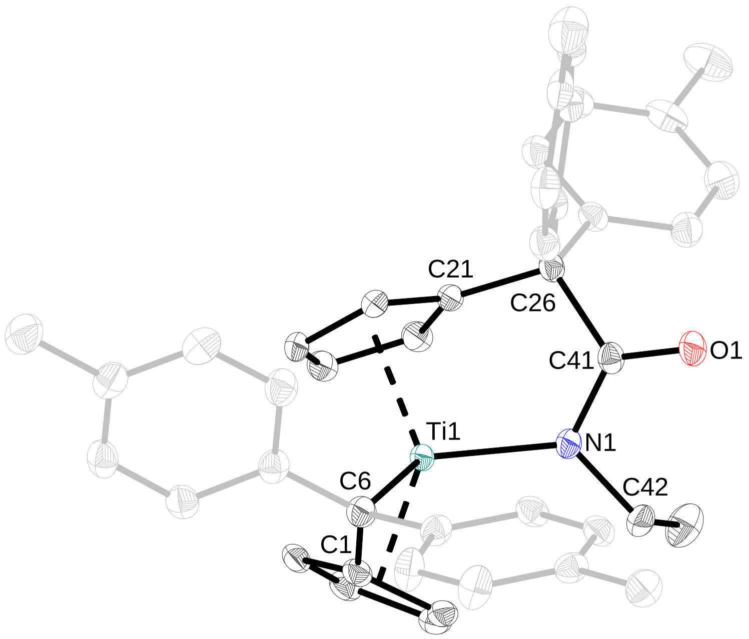

Figure S120: Molecular structure of **2a**. Thermal ellipsoids drawn at the 50% probability level. Hydrogen atoms are omitted for clarity. Selected bond lengths [Å] and angles [°]: Ti1–N1 2.0741(10), Ti1–C6 2.5589(13), C1–C6 1.4340(18), C21–C26 1.5176(16), N1–C41 1.3725(16), N1–C42 1.4864(15), C26–C41 1.5540(16), C41–O1 1.2315(15); N1–Ti1–C6 112.88(4), Ti1–N1–C41 120.68(8), N1–C41–C26 117.16(10), C26–C41–O1 118.33(11), N1–C41–O1 124.51 (11).

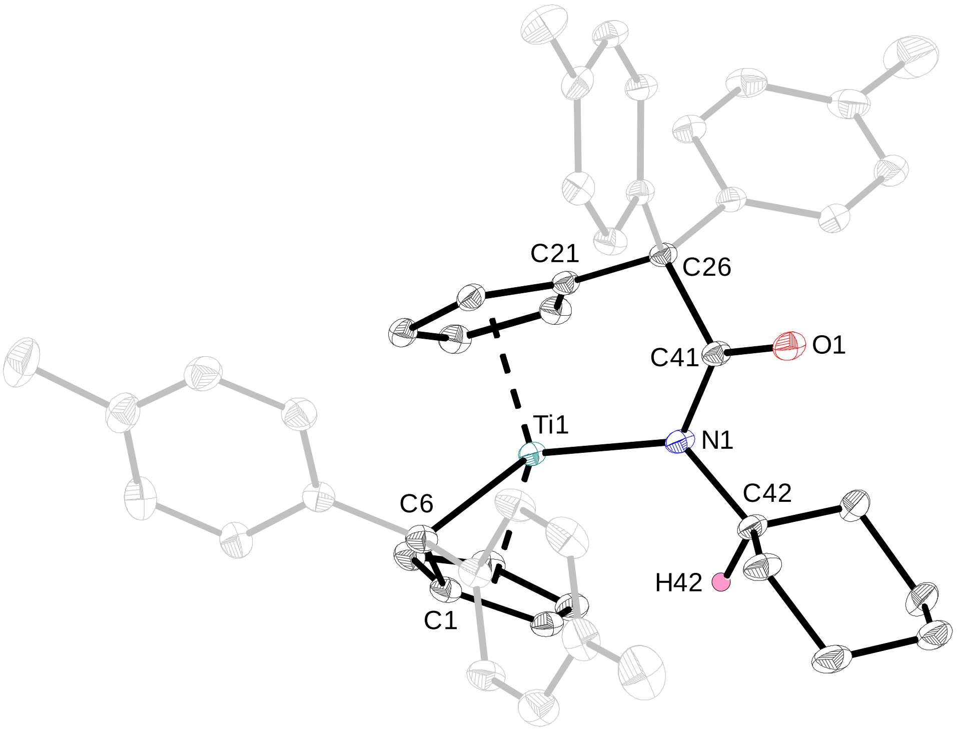

Figure S121: Molecular structure of **2b**. Thermal ellipsoids drawn at the 50% probability level. Hydrogen atoms (except H42) and solvent molecules are omitted for clarity. Selected bond lengths [Å] and angles [°]: Ti1–N1 2.0908(8), Ti1–C6 2.4726(10), C1–C6 1.4419(13), C21–C26 1.5144(12), N1–C41 1.3715(11), N1–C42 1.5016(11), C26–C41 1.5564(12), C41–O1 1.2355(11); N1–Ti1–C6 112.56(3), Ti1–N1–C41 121.75(6), N1–C41–C26 116.89(7), C26–C41–O1 117.34(8), N1–C41–O1 125.74 (8).

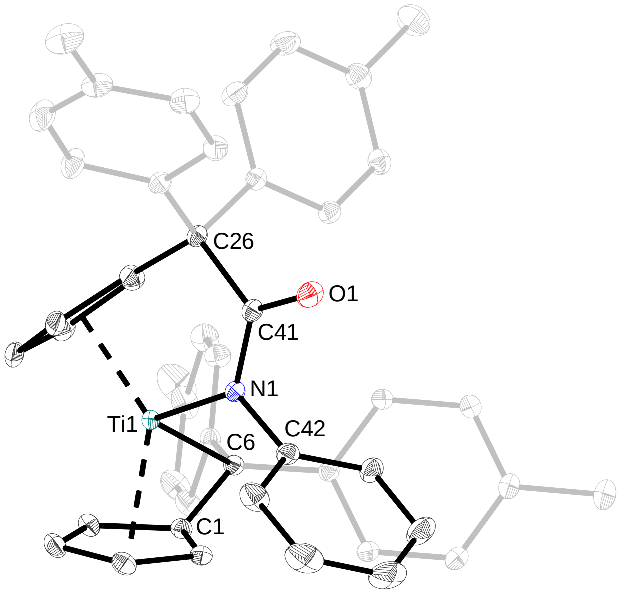

Figure S122: Molecular structure of **2c**. Thermal ellipsoids drawn at the 50% probability level. Hydrogen atoms and solvent molecules are omitted for clarity. Selected bond lengths [Å] and angles [°]: Ti1–N1 2.0778(7), Ti1–C6 2.4207(8), C1–C6 1.4452(11), C21–C26 1.5138(11), N1–C41 1.3769(11), N1–C42 1.4499(11), C26–C41 1.5567(11), C41–O1 1.2279(10); N1–Ti1–C6 108.27(3), Ti1–N1–C41 125.25(5), N1–C41–C26 116.58(7), C26–C41–O1 119.63(7), N1–C41–O1 123.75 (8).

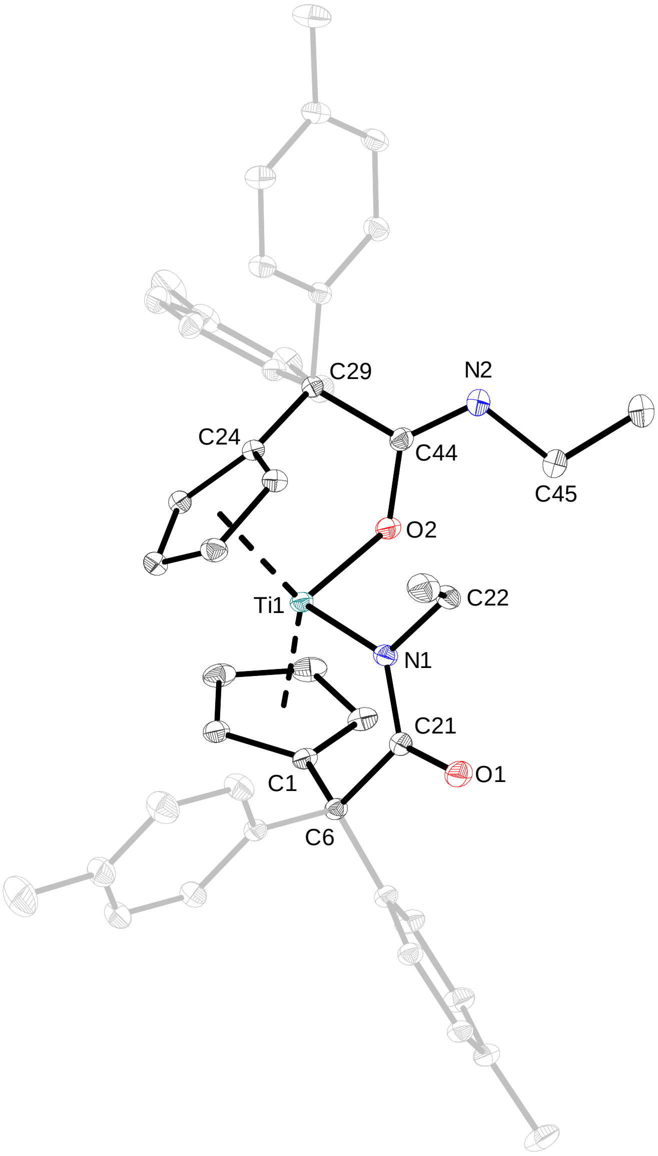

Figure S123: Molecular structure of **3a**. Thermal ellipsoids drawn at the 50% probability level. Hydrogen atoms and solvent molecules are omitted for clarity. Selected bond lengths [Å] and angles [°]: Ti1–O2 1.9171(7), Ti1–N1 2.0750(8), C1–C6 1.5124(12), C24–C29 1.5174(12), N1–C21 1.3573(12), N1–C22 1.4780(12), O2–C44 1.3471(11), O1–C21 1.2350(11), N2–C44 1.2649(12), N2–C45 1.4752(14), C29–C44 1.5442(13), C6–C21 1.5552(13); O2–Ti1–N1 97.88(3), Ti1–O2–C44 127.26(6), Ti1–N1–C21 126.34(6), O2–C44–C29 112.29(8), O2–C44–N2 123.82(9), N1–C21–C6 114.61(8), N1–C21–O1 124.98(9).

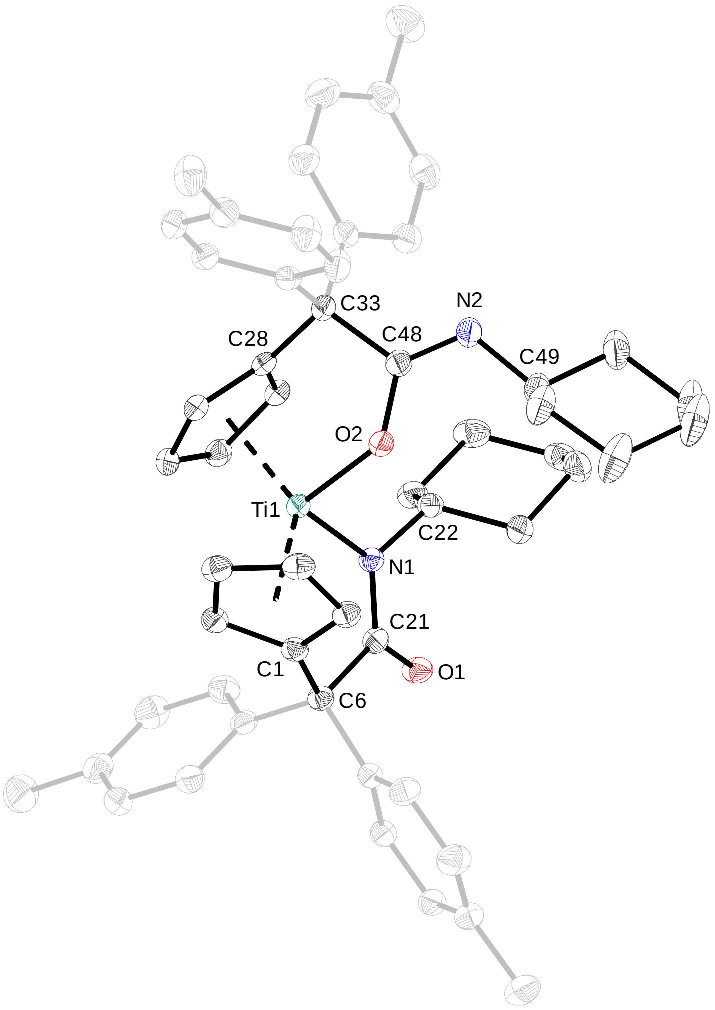

Figure S124: Molecular structure of **3b**. Thermal ellipsoids drawn at the 50% probability level. Hydrogen atoms, solvent molecules and additional crystallographic independent molecules are omitted for clarity. Selected bond lengths [Å] and angles [°]: Ti1–O2 1.9201(12), Ti1–N1 2.0784(14), C1–C6 1.515(2), C28–C33 1.528(2), N1–C21 1.356(2), N1–C22 1.487(2), O2–C48 1.344(2), O1–C21 1.233(2), N2–C48 1.266(2), N2–C49 1.470(2), C33–C48 1.554(2), C6–C21 1.562(2); O2–Ti1–N1 97.99(6), Ti1–O2–C48 130.39(11), Ti1–N1–C21 127.20(12), O2–C48–C33 113.99(15), O2–C48–N2 124.83(16), N1–C21–C6 115.16(14), N1–C21–O1 125.78(16).

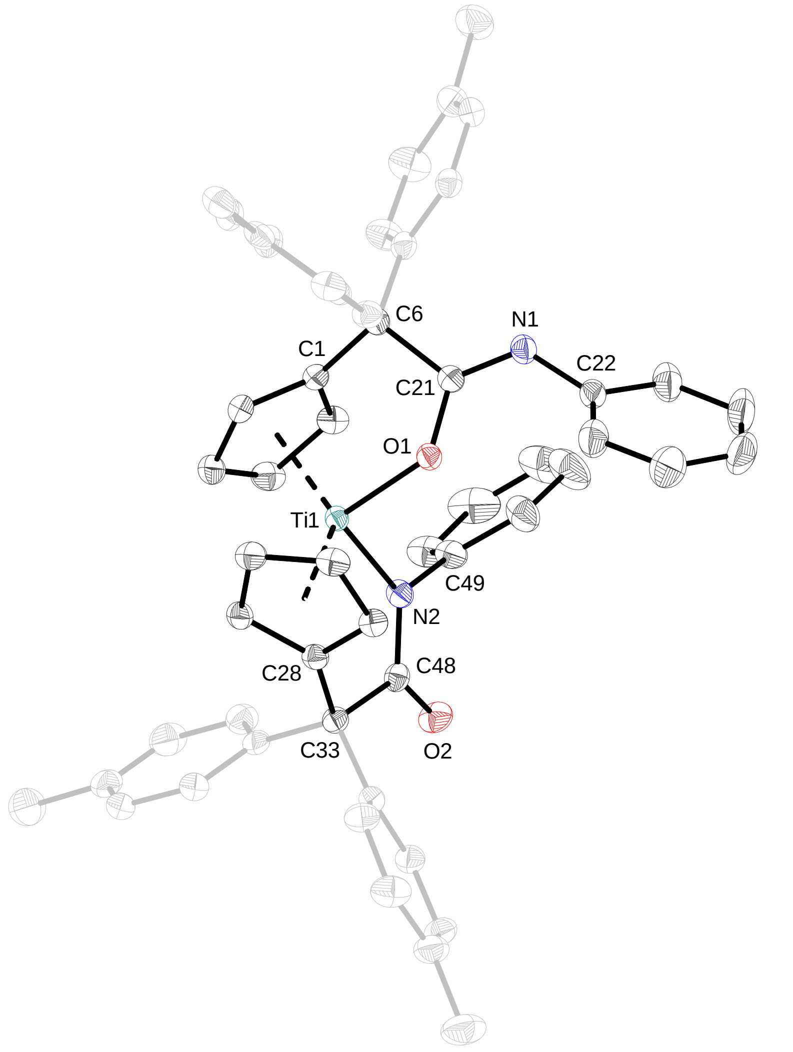

Figure S125: Molecular structure of **3c**. Thermal ellipsoids drawn at the 50% probability level. Hydrogen atoms, solvent molecules and additional crystallographic independent molecules are omitted for clarity. Selected bond lengths [Å] and angles [°]: Ti1–O1 1.9506(9), Ti1–N2 2.0876(11), C1–C6 1.5191(18), C28–C33 1.5158(18), N1–C21 1.2694(18), N1–C22 1.4133(19), O2–C48 1.2276(17), O1–C21 1.3311(16), N2–C48 1.3586(18), N2–C49 1.4399(18), C33–C48 1.5591(18), C6–C21 1.5514(19); N2–Ti1–O1 99.98(4), Ti1–N2–C48 128.08(9), Ti1–O1–C21 128.14(9), N2–C48–C33 114.43(11), O2–C48–N2 124.15(13), N1–C21–C6 119.87(12), N1–C21–O1 126.42(13).

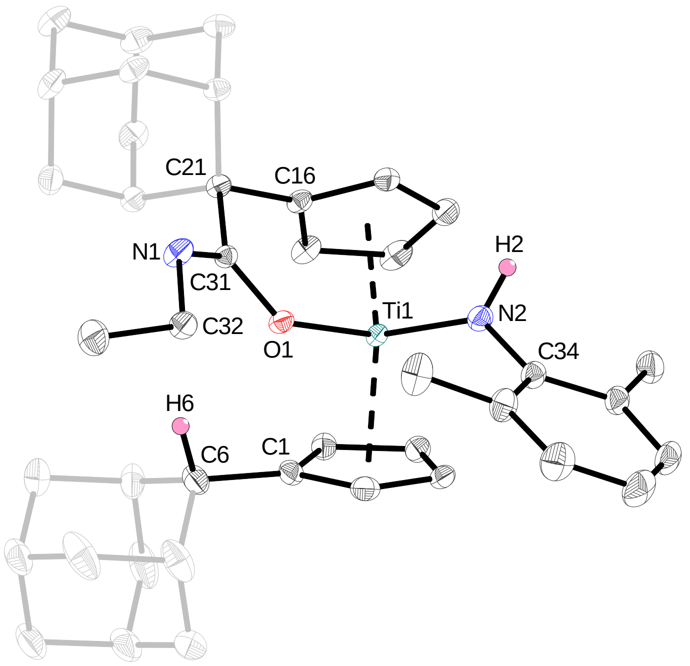

Figure S126: Molecular structure of complex **4h**. Thermal ellipsoids drawn at the 50% probability level. Hydrogen atoms (except for H2 and H6), solvent molecules and disorders are omitted for clarity. Selected bond lengths [Å] and angles [°]: Ti1–O1 1.9380(10), Ti1–N2 1.9891(11), C1–C6 1.5106(17), C16–C21 1.5169(16), C21–C31 1.5582(16), N2–C34 1.4163(16), O1–C31 1.3310(15), C31–N1 1.2743(16), N1–C32 1.461(2); N2–Ti1–O1 100.61(5), Ti1–N2–C34 134.70(9), Ti1–O1–C31 133.07(9), N1–C31–O1 123.81(11), N1–C31–C21 121.56(11).

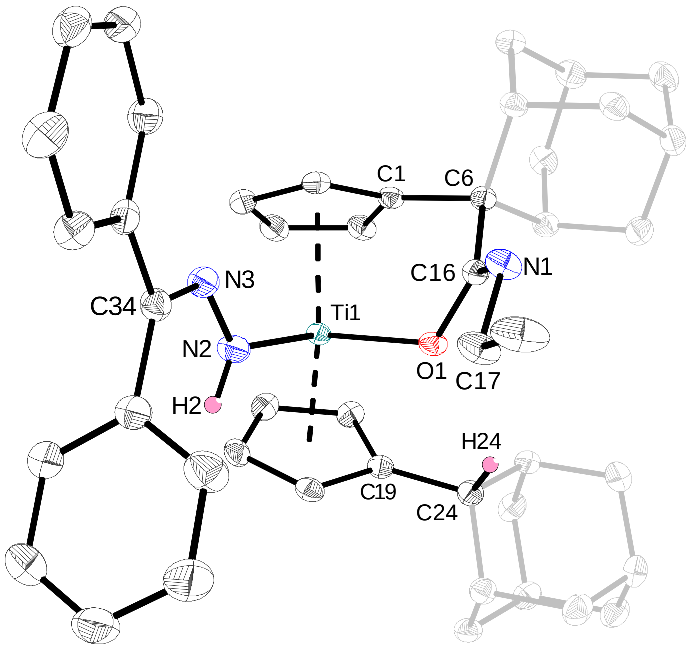

Figure S127: Molecular structure of complex **4i**. Thermal ellipsoids drawn at the 50% probability level. Hydrogen atoms (except for H2 and H24) are omitted for clarity. Selected bond lengths [Å] and angles [°]: Ti1–O1 1.9436(8), Ti1–N2 2.0063(11), N2–N3 1.3564(15), N3–C34 1.2966(17), C1–C6 1.5208(16), C19–C24 1.5053(16), O1–C16 1.3410(14), C16–N1 1.2724(16), N1–C17 1.4729(16), C6–C16 1.5462(16); N2–Ti1–O1 100.18(4), Ti1–N2–N3 127.52(8), Ti1–O1–C16 121.75(7), N1–C16–O1 123.82(11), N1–C16–C6 124.16(11), N2–N3–C34 120.63(11).

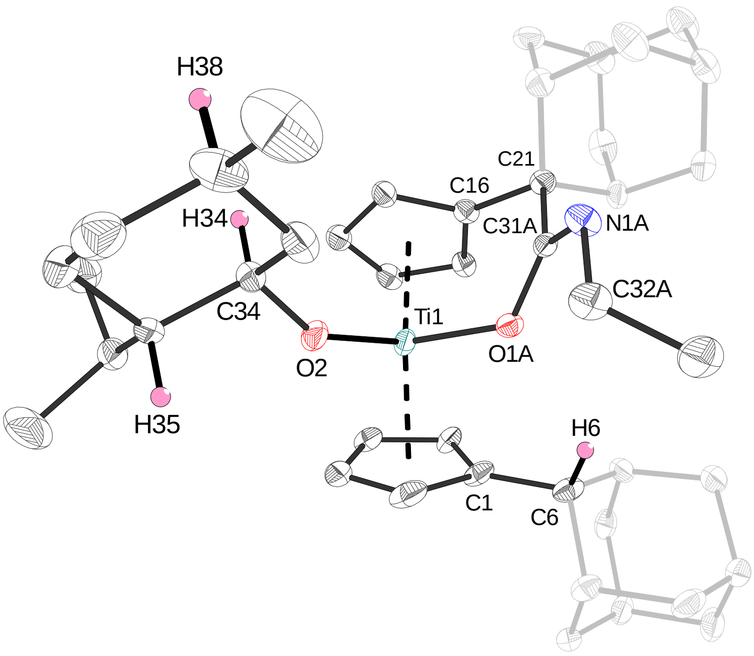

Figure S128: Molecular structure of complex **4k** (*S*-conformer). Thermal ellipsoids drawn at the 50% probability level. Hydrogen atoms (except for H6, H34, H35, H38) and disorders are omitted for clarity. Selected bond lengths [Å] and angles [°]: Ti1–O2 1.838(2), Ti1–O1A 1.975(3), C1–C6 1.502(4), C16–C21 1.513(4), O2–C34 1.411(3), O1A–C31A 1.343(5), C31A–N1A 1.258(5), C32A–N1A 1.469(4), C21–C31A 1.578(4); O1A–Ti1–O2 99.39(12), Ti1–O2–C34 147.85(18), Ti1–O1A–C31A 126.9(2), N1A–C31A–O1A 124.8(3), N1A–C31A–C21 122.7(3).

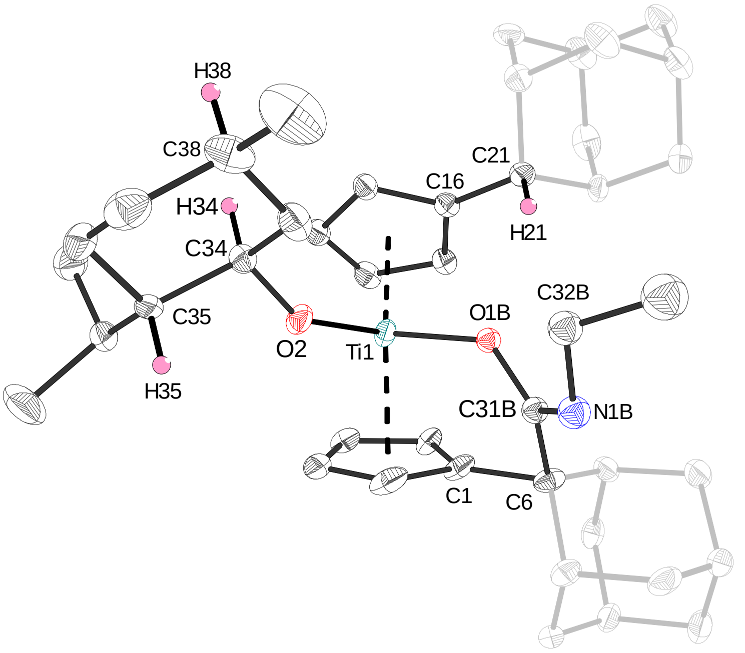

Figure S129: Molecular structure of complex **4k** (*R*-conformer). Thermal ellipsoids drawn at the 50% probability level. Hydrogen atoms (except for H21, H34, H35, H38) and disorders are omitted for clarity. Selected bond lengths [Å] and angles [°]: Ti1–O2 1.838(2), Ti1–O1B 1.837(12), C1–C6 1.502(4), C16–C21 1.513(4), C6–C31B 1.586(14), O2–C34 1.411(3), O1B–C31B 1.365(18), C31B–N1B 1.266(19), C32B–N1B 1.47(2); O1B–Ti1–O2 106.9(4), Ti1–O2–C34 147.85(18), Ti1–O1B–C31B 131.8(11), N1B–C31B–O1B 121.8(13), N1B–C31B–C6 123.5(12).

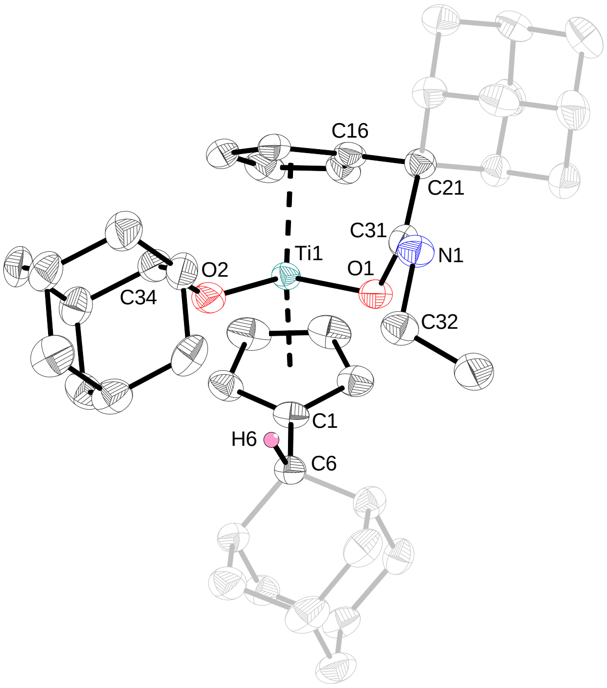

Figure S130: Molecular structure of complex **4l**. Thermal ellipsoids drawn at the 50% probability level. Hydrogen atoms (except for H6) and disorders are omitted for clarity. Selected bond lengths [Å] and angles [°]: Ti1–O2 1.855(2), Ti1–O1 1.946(2), C1–C6 1.516(4), C16–C21 1.525(5), C21–C31 1.540(4), O2–C34 1.407(4), O1–C31 1.343(4), C31–N1 1.265(4), C32–N1 1.472(4); O2–Ti1–O1 99.59(11), Ti1–O2–C34 144.8(2), Ti1–O1–C31 122.64(18), N1–C31–O1 124.8(3), N1–C31–C21 123.1(3).

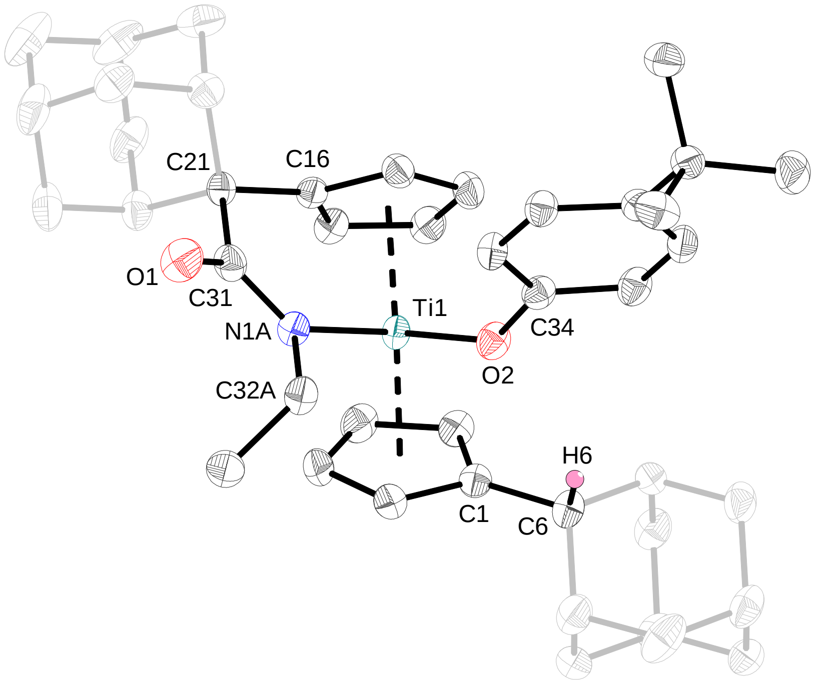

Figure S131: Molecular structure of complex **4m'**. Thermal ellipsoids drawn at the 50% probability level. Hydrogen atoms (except for H6), solvent molecules and disorders are omitted for clarity. Selected bond lengths [Å] and angles [°]: Ti1–O2 1.8830(12), Ti1–N1A 2.0753(19), C1–C6 1.509(2), C16–C21 1.513(2), N1A–C32A 1.481(3), N1A–C31 1.348(3), C31–O1 1.243(2), C21–C31 1.554(2), O2–C34 1.343(2); O2–Ti1–N1A 94.91(8), Ti1–O2–C34 142.05(11), Ti1–N1A–C31 128.15(14), N1A–C31–O1 122.51(18), N1A–C31–C21 115.92(16).

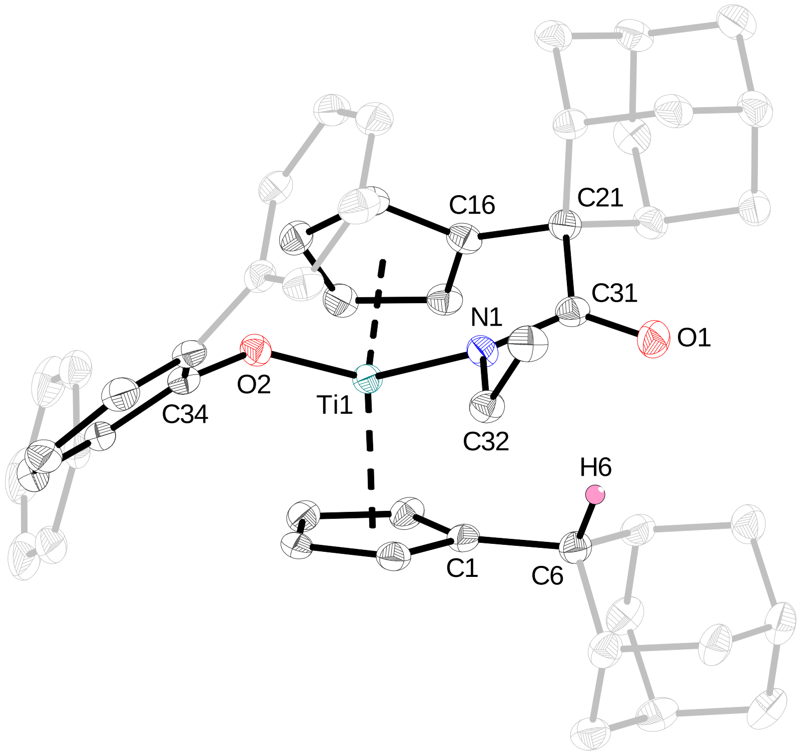

Figure S132: Molecular structure of complex **4n'**. Thermal ellipsoids drawn at the 50% probability level. Hydrogen atoms (except for H6) and solvent molecules are omitted for clarity. Selected bond lengths [Å] and angles [°]: Ti1–O2 1.9064(17), Ti1–N1 2.051(2), C1–C6 1.510(3), C16–C21 1.513(3), N1–C32 1.480(3), N1–C31 1.371(3), C31–O1 1.237(3), C21–C31 1.544(4), O2–C34 1.344(3); O2–Ti1–N1 100.29(8), Ti1–O2–C34 150.43(16), Ti1–N1–C31 123.64(16), N1–C31–O1 123.3(2), N1–C31–C21 114.5(2).

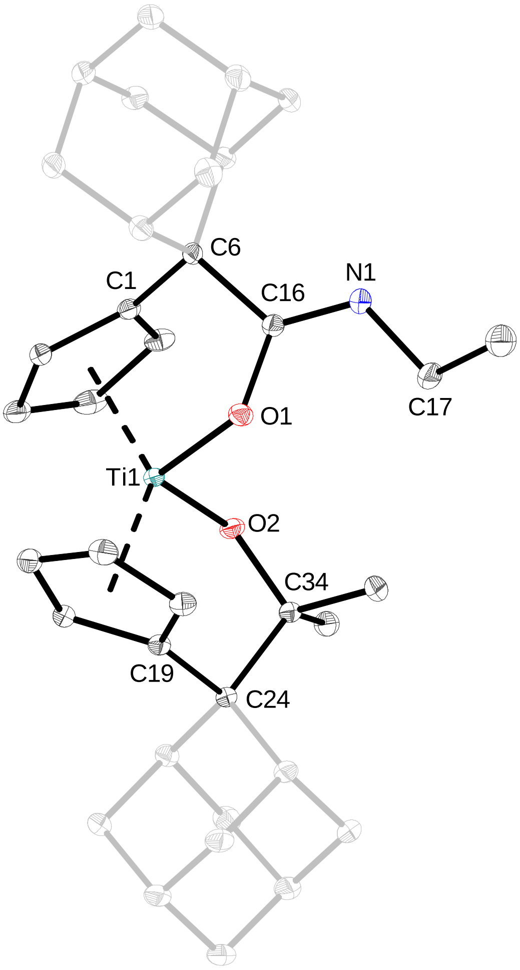

Figure S133: Molecular structure of complex **5o**. Thermal ellipsoids drawn at the 50% probability level. Hydrogen atoms are omitted for clarity. Selected bond lengths [Å] and angles [°]: Ti1–O2 1.8635(4), Ti1–O1 1.9408(4), O2–C34 1.4335(6), C1–C6 1.5182(9), C19–C24 1.5228(9), C34–C24 1.6178(7), O1–C16 1.3457(6), C16–N1 1.2733(7), C6–C16 1.5494(7); O2–Ti1–O1 104.34(2), Ti1–O2–C34 128.93(3), Ti1–O1–C16 124.87(3), O1–C16–N1 124.49(5), O1–C16–C6 112.24(4), O2–C34–C24 107.72(4).

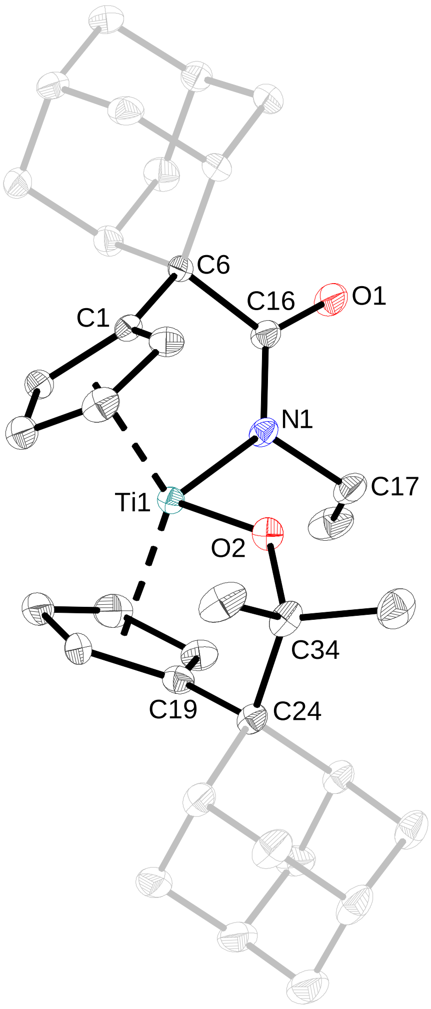

Figure S134: Molecular structure of complex **5o'**. Thermal ellipsoids drawn at the 50% probability level. Hydrogen atoms are omitted for clarity. Selected bond lengths [Å] and angles [°]: Ti1–O2 1.8459(17), Ti1–N1 2.086(2), O2–C34 1.427(3), C1–C6 1.507(3), C6–C16 1.538(4), C19–C24 1.518(4), C24–C34 1.624(4), O1–C16 1.241(3), C16–N1 1.361(3), N1–C17 1.487(3); O2–Ti1–N1 97.92(9), Ti1–O2–C34 134.58(19), Ti1–N1–C16 128.74(17), O1–C16–N1 123.4(2), O1–C16–C6 120.9(2), O2–C34–C24 108.5(2).

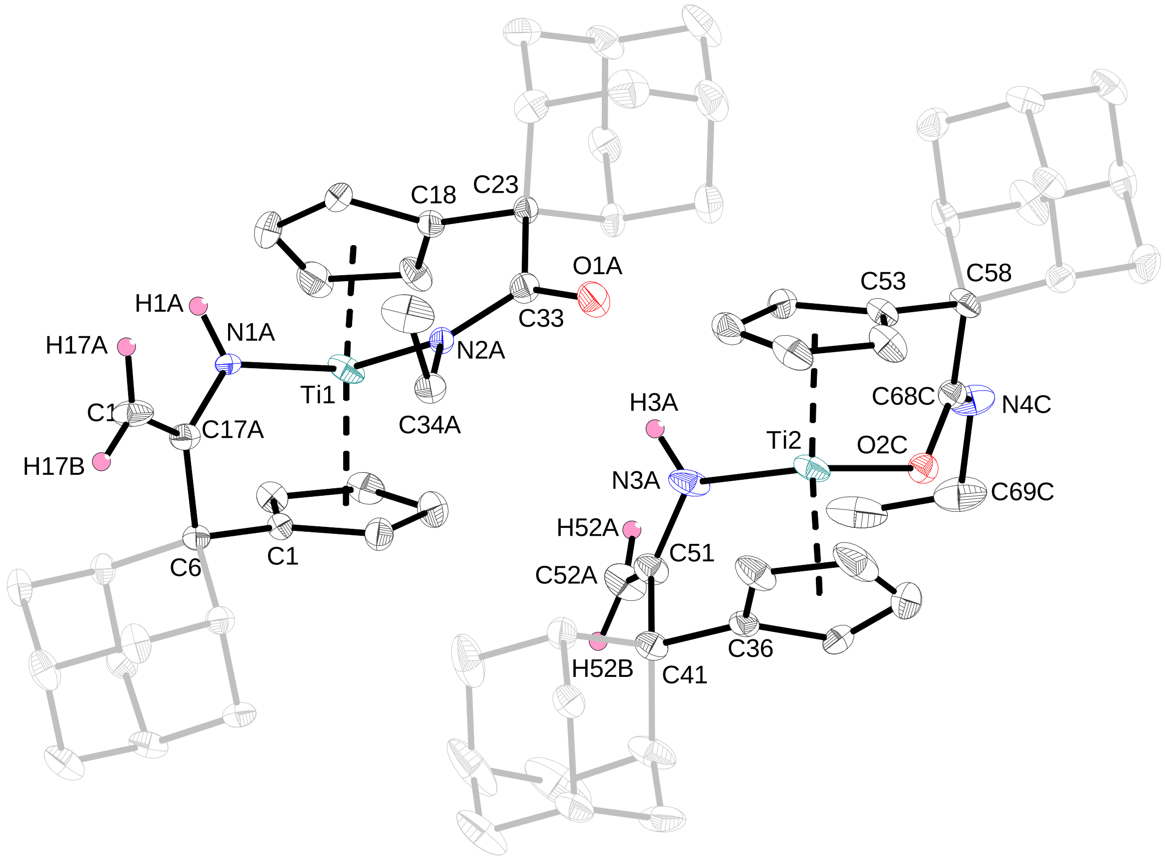

Figure S135: Molecular structure of complex **5p** (left) and **5p'** (right). Thermal ellipsoids drawn at the 50% probability level. Hydrogen atoms (except for H1A, H3A, H17A, H17B, H52A, H52B) and disorders are omitted for clarity. Selected bond lengths [Å] and angles [°]: Ti1–N2A 2.041(3), Ti1–N1A 1.984(3), C1–C6 1.527(3), C6–C16 1.549(3), C16–C17A 1.384(5), C18–C23 1.518(4), C23–C33 1.557(3), N2A–C33 1.365(4), N2A–C34A 1.477(4), C33–O1A 1.232(4), N1A–C16 1.377(4), Ti2–O2C 1.954(6), Ti2–N3A 1.979(3), C36–C41 1.515(4), C41–C51 1.550(3), C51–C52A 1.352(4), C53–C58 1.519(4), C58–C68C 1.516(8), O2C–C68C 1.331(10), C68C–N4C 1.454(9), N4C–C69C 1.454(9), N3A–C51 1.380(4); N1A–Ti1–N2A 96.19(12), Ti1–N2A–C33 125.79(19), Ti1–N1A–C16 128.94(19), N1A–C16–C17A 118.9(3), N1A–C16–C6 113.2(2), N2A–C33–C23 116.0(2), N3A–Ti2–O2C 111.29(25), Ti2–O2C–C68C 125.6(6), Ti2–N3A–C51 125.4(2), N3A–C51–C52A 121.8(3), N3A–C51–C41 110.6(2), O2C–C68C–C58 112.1(6).

Table S1: Crystal Structure Data for Compounds **1a**, **1c**, **1d** and **1e**.

	**1a**	**1c**	**1d**	**1e**
CCDC	2422778	2422782	2422767	2422779
Lab-ID	TBF12	TBF16	MAP163	MAP294
empirical formula	$C_{36.50}H_{45}NOTi$	$C_{43}H_{41}D_6NOTi$	$C_{37}H_{40}ClNOTi$	$C_{37}H_{40}FNOTi$
Fw	561.63	647.75	598.05	581.60
Color	green blue	green blue	green	green
Habit	block	block	rod	block
Wavelength, Å	0.71073	0.71073	1.54178	0.71073
cryst dimens, mm	0.14 x 0.10 x 0.07	0.15 x 0.13 x 0.11	0.11 x 0.05 x 0.05	0.12 x 0.10 x 0.06
cryst syst	triclinic	triclinic	monoclinic	monoclinic
space group	*P*-1	*P*-1	$P2_1/c$	$P2_1/c$
a, Å	9.7368(4)	10.8031(9)	12.6891(3)	16.5415(6)
b, Å	10.2428(4)	12.5543(10)	35.0821(9)	12.6422(4)
c, Å	14.9192(6)	12.6432(10)	13.0894(3)	13.6678(5)
α, deg	88-8342(14)	95.061(3)	90	90
β, deg	83.8240(14)	110.921(3)	91.2550(8)	103.7475(14)
γ, deg	79.1093(13)	92.957(3)	90	90
V, $Å^3$	1452.64(10)	1589.1(2)	5825.5(2)	2776.34(17)
Z	2	2	8	4
ρ_{caclcd}, g cm^{-3}	1.284	1.354	1.364	1.391
μ, mm^{-1}	0.325	0.307	3.566	0.348
T, K	100(2)	100(2)	100(2)	100(2)
λ range, deg	1.373 – 40.249	1.635 – 40.249	2.519 – 74.488	1.267 – 40.249
no. of rflns collected	151776	193624	22209	196846
no. of indep rflns (R(int))	18285 0.0380	20011 0.0344	22209 N/A	17444 0.0464
no. of rflns with $I>2\sigma(I)$	16630	18590	21143	15324
abs cor	semi-empirical	semi-empirical	semi-empirical	semi-empirical
max, min transmission	1.0000, 0.9476	1.0000, 0.9451	1.00000, 0.836588	1.0000, 0.9219
final R indices [$I>2\sigma(I)$]	R1 = 0.0297 wR2 = 0.0813	R1 = 0.0274 wR2 = 0.0811	R1 = 0.0289 wR2 = 0.0762	R1 = 0.0318 wR2 = 0.0850
R indices (all data)	R1 = 0.0341 wR2 = 0.0836	R1 = 0.0302 wR2 = 0.0829	R1 = 0.0309 wR2 = 0.0782	R1 = 0.0383 wR2 = 0.0888
GOF on F^2	1.044	1.080	1.038	1.042
largest diff peak / hole (e.$Å^{-3}$)	0.531 / -0.437	0.566 / -0.420	0.347 / -0.296	0.617 / -0.473

Table S2: Crystal Structure Data for Compounds **1f**, **2a**, **2b** and **2c**.

	**1f**	**2a**	**2b**	**2c**
CCDC	2422776	2422768	2422777	2422775
Lab-ID	MAP295	MAP311	MAP312	TBF18
empirical formula	$C_{38}H_{40}F_3NOTi$	$C_{43}H_{41}NOTi$	$C_{53}H_{47}D_6NOTi$	$C_{50}H_{41}D_3NOTi$
fw	631.61	635.67	773.90	725.78
color	green	yellow green	green	green blue
Habit	plate	rod	plate	plate
Wavelength, Å	0.71073	1.54178	0.71073	0.71073
cryst dimens, mm	0.12 x 0.05 x 0.02	0.12 x 0.04 x 0.03	0.14 x 0.12 x 0.05	0.12 x 0.08 x 0.02
cryst syst	monoclinic	triclinic	triclinic	triclinic
space group	$P2_1/c$	*P*-1	*P*-1	*P*-1
a, Å	16.9955(7)	8.4384(2)	8.7847(6)	8.6443(3)
b, Å	12.7519(5)	11.2814(3)	13.7484(10)	14.0684(5)
c, Å	14.1569(5)	17.9950(4)	19.0277(12)	16.1548(5)
α, deg	90	80.5530(11)	71.031(2)	81.6436(13)
β, deg	100.0455(14)	84.9926(10)	87.856(2)	85.1597(14)
γ, deg	90	87.1649(10)	75.292(3)	83.0431(13)
V, Å^3	3021.1(2)	1682.31(7)	2099.5(3)	1924.98(11)
Z	4	2	2	2
ρ_{caclcd}, g cm^{-3}	1.389	1.255	1.224	1.252
μ, mm^{-1}	0.335	2.410	0.243	0.261
T, K	100(2)	100(2)	100(2)	100(2)
λ range, deg	2.008 – 34.971	2.497 – 74.487	1.620 – 34.971	1.277 – 34.971
no. of rflns collected	148197	40146	164710	130481
no. of indep rflns (R(int))	13283 0.0605	6829 0.0319	18457 0.0464	16881 0.0455
no. of rflns with $I>2\sigma(I)$	11410	6498	16113	14836
abs cor	semi-empirical	semi-empirical	semi-empirical	semi-empirical
max, min transmission	1.0000, 0.9094	1.0000, 0.9140	1.0000, 0.9033	1.0000, 0.9367
final R indices [$I>2\sigma(I)$]	R1 = 0.0456 wR2 = 0.1031	R1 = 0.0300 wR2 = 0.0788	R1 = 0.0461 wR2 = 0.1099	R1 = 0.0395 wR2 = 0.0997
R indices (all data)	R1 = 0.0562 wR2 = 0.1079	R1 = 0.0319 wR2 = 0.0803	R1 = 0.0551 wR2 = 0.1141	R1 = 0.0457 wR2 = 0.1036
GOF on F^2	1.120	1.044	1.121	1.063
largest diff peak / hole (e.Å^{-3})	0.593 / -0.615	0.291 / -0.321	0.588 / -0.550	0.555 / -0.383

Table S3: Crystal Structure Data for Compounds **3a**, **3b**, **3c** and **4h**.

	**3a**	**3b**	**3c**	**4h**
CCDC	2422784	2422772	2422786	2422774
Lab-ID	TBF31	MAP306	MAP304	MAP264
empirical formula	$C_{58}H_{46}D_{12}N_2O_2Ti$	$C_{61}H_{66}N_2O_2Ti$	$C_{66.25}H_{60}N_2O_2Ti$	$C_{54.67}H_{70}N_2OTi$
fw	875.03	907.05	964.06	819.00
color	yellow	yellow	yellow	yellow
Habit	block	plate	block	block
Wavelength, Å	0.71073	1.54178	1.54178	0.71073
cryst dimens, mm	0.14 x 0.10 x 0.08	0.08 x 0.05 x 0.01	0.11 x 0.09 x 0.04	0.14 x 0.10 x 0.06
cryst syst	orthorhombic	triclinic	triclinic	triclinic
space group	*Pbca*	*P*-1	*P*-1	*P*-1
a, Å	15.2088(4)	16.3755(4)	11.0485(3)	10.4173(5)
b, Å	20.8806(6)	16.8155(4)	16.5903(5)	14.5949(8)
c, Å	29.7446(9)	18.8207(4)	28.4308(9)	15.4449(8)
α, deg	90	90.1510(14)	89.0549(15)	75.395(2)
β, deg	90	96.7271(14)	80.2939(15)	84.140(2)
γ, deg	90	104.8089(13)	87.9372(16)	83.987(2)
V, $Å^3$	9446.0(5)	4973.0(2)	5133.1(3)	2252.9(2)
Z	8	4	4	2
ρ_{caclcd}, g cm^{-3}	1.231	1.212	1.247	1.207
μ, mm^{-1}	0.226	1.800	1.781	0.231
T, K	100(2)	100(2)	100(2)	100(2)
λ range, deg	1.369 – 34.970	2.365 – 66.589	2.665 – 74.476	1.447 – 32.032
no. of rflns collected	535998	91247	151358	111373
no. of indep rflns (R(int))	20755 0.0522	17498 0.0615	20851 0.0419	15680 0.0362
no. of rflns with $I>2\sigma(I)$	18767	14408	18589	14080
abs cor	semi-empirical	semi-empirical	semi-empirical	semi-empirical
max, min transmission	1.0000, 0.9349	1.0000 and 0.8934	1.0000 and 0.9073	1.0000, 0.9313
final R indices [$I>2\sigma(I)$]	R1 = 0.0494 wR2 = 0.1207	R1 = 0.0395 wR2 = 0.0930	R1 = 0.0365 wR2 = 0.0951	R1 = 0.0518 wR2 = 0.1323
R indices (all data)	R1 = 0.0554 wR2 = 0.1241	R1 = 0.0531 wR2 = 0.1001	R1 = 0.0420 wR2 = 0.0988	R1 = 0.0583 wR2 = 0.1362
GOF on F^2	1.132	0.997	1.055	1.119
largest diff peak / hole (e.$Å^{-3}$)	0.779 / -0.614	0.530 / -0.480	0.487 / -0.336	0.913 / -0.445

Table S4: Crystal Structure Data for Compounds **4i**, **4k**, **4l** and **4m**.

	**4i**	**4k**	**4l**	**4m**
CCDC	2422769	2422771	2422770	2422780
Lab-ID	MAP265	MAP258	MAP316	TBF21
empirical formula	$C_{46}H_{53}N_3OTi$	$C_{43}H_{61}NO_2Ti$	$C_{43}H_{57}NO_2Ti$	$C_{49}H_{69}NO_2Ti$
fw	711.81	671.82	667.79	751.95
color	yellow orange	yellow	yellow	yellow
Habit	plate	plate	rod	plate
Wavelength, Å	1.54178	0.71073	1.54178	1.54178
cryst dimens, mm	0.08 x 0.08 x 0.02	0.09 x 0.08 x 0.04	0.07 x 0.025 x 0.025	0.08 x 0.08 x 0.025
cryst syst	monoclinic	triclinic	monoclinic	orthorhombic
space group	$P2_1/n$	*P*1	$P2_1/n$	*Pbca*
a, Å	14.1846(3)	9.6799(3)	10.3016(3)	17.4548(5)
b, Å	20.1042(5)	10.4384(3)	19.9035(7)	19.1379(6)
c, Å	14.2288(3)	17.9604(6)	16.7159(6)	24.5961(8)
α, deg	90	96.3074(13)	90	90
β, deg	116.0079(8)	98.1156(14)	90.121(2)	90
γ, deg	90	93.0896(13)	90	90
V, Å^3	3646.72(14)	1781.27(10)	3427.4(2)	8216.3(4)
Z	4	2	4	8
ρ_{caclcd}, g cm^{-3}	1.296	1.253	1.294	1.216
μ, mm^{-1}	2.292	0.278	2.402	2.058
T, K	100(2)	100(2)	100(2)	100(2)
λ range, deg	3.668 – 74.484	1.968 – 30.032	2.220 – 66.592	3.594 – 74.490
no. of rflns collected	69683	97736	60235	172984
no. of indep rflns (R(int))	7462 0.0318	20867 0.0370	6045 0.0807	8390 0.0592
no. of rflns with $I>2\sigma(I)$	7132	19584	5542	7281
abs cor	semi-empirical	semi-empirical	semi-empirical	semi-empirical
max, min transmission	1.0000, 0.8959	1.0000, 0.9596	1.0000, 0.9028	1.0000, 0.8924
final R indices [$I>2\sigma(I)$]	R1 = 0.0316 wR2 = 0.0859	R1 = 0.0432 wR2 = 0.1035	R1 = 0.0440 wR2 = 0.1133	R1 = 0.0440 wR2 = 0.1147
R indices (all data)	R1 = 0.0330 wR2 = 0.0871	R1 = 0.0469 wR2 = 0.1060	R1 = 0.0495 wR2 = 0.1176	R1 = 0.0516 wR2 = 0.1206
GOF on F^2	1.023	1.036	1.028	1.021
largest diff peak / hole (e.Å^{-3})	0.337 / -0.252	0.725 / -0.470	0.273 / -0.484	0.703 / -0.320

Table S5: Crystal Structure Data for Compounds **4n**, **5o**, **5o’** and **5p**.

	**4n**	**5o**	**5o’**	**5p**
CCDC	2422773	2422785	2422781	2422783
Lab-ID	TBF23	TBF28C	TBF28Y	MAP274
empirical formula	$C_{69}H_{55}D_{18}NO_2Ti$	$C_{36}H_{47}NO_2Ti$	$C_{36}H_{47}NO_2Ti$	$C_{35}H_{44}N_2OTi$
fw	1014.29	573.64	573.64	556.62
color	yellow	colorless	yellow	orange
Habit	needle	block	block	block
Wavelength, Å	1.54178	0.71073	0.71073	0.71073
cryst dimens, mm	0.20 x 0.02 x 0.01	0.17 x 0.15 x 0.14	0.19 x 0.16 x 0.14	0.15 x 0.11 x 0.10
cryst syst	orthorhombic	monoclinic	monoclinic	monoclinic
space group	*Pbca*	$P2_1/n$	*Pc*	*Ia*
a, Å	26.6768(11)	11.0708(5)	10.5181(6)	13.5258(5)
b, Å	10.9416(6)	14.5375(7)	15.3581(9)	16.4908(5)
c, Å	35.7018(17)	17.6362(9)	17.6541(11)	24.8930(10)
α, deg	90	90	90	90
β, deg	90	93.2028(18)	95.764(2)	92.1763(11)
γ, deg	90	90	90	90
V, $Å^3$	10420.9(9)	2834.0(2)	2837.4(3)	5548.4(4)
Z	8	4	4	8
ρ_{caclcd}, g cm^{-3}	1.293	1.344	1.343	1.333
μ, mm^{-1}	1.760	0.337	0.337	0.341
T, K	100(2)	100(2)	100(2)	100(2)
λ range, deg	2.475 – 66.590	1.817 – 40.249	1.326 – 34.971	1.482 – 34.971
no. of rflns collected	94812	227899	201832	147922
no. of indep rflns (R(int))	9193 0.1695	17835 0.0382	24900 0.0456	23288 0.0390
no. of rflns with $I>2\sigma(I)$	6225	16348	22938	21685
abs cor	semi-empirical	semi-empirical	semi-empirical	semi-empirical
max, min transmission	1.0000, 0.8571	1.0000, 0.9379	1.0000, 0.9074	1.0000, 0.9437
final R indices [$I>2\sigma(I)$]	R1 = 0.0491 wR2 = 0.0995	R1 = 0.0292 wR2 = 0.0328	R1 = 0.0467 wR2 = 0.1207	R1 = 0.0525 wR2 = 0.1232
R indices (all data)	R1 = 0.0884 wR2 = 0.1155	R1 = 0.0328 wR2 = 0.0808	R1 = 0.0517 wR2 = 0.1245	R1 = 0.0573 wR2 = 0.1258
GOF on F^2	1.003	1.059	1.049	1.095
largest diff peak / hole (e.$Å^{-3}$)	0.240 / -0.360	0.594 / -0.426	1.345 / -0.910	1.260 / -1.310

Computational Part

The DFT (density functional theory) calculations were performed with the B3LYP/Def2-TZVP level of theory.[8] The optimized ground state geometries and the transition states were calculated in toluene at room temperature using the SMD solvation model.[9]

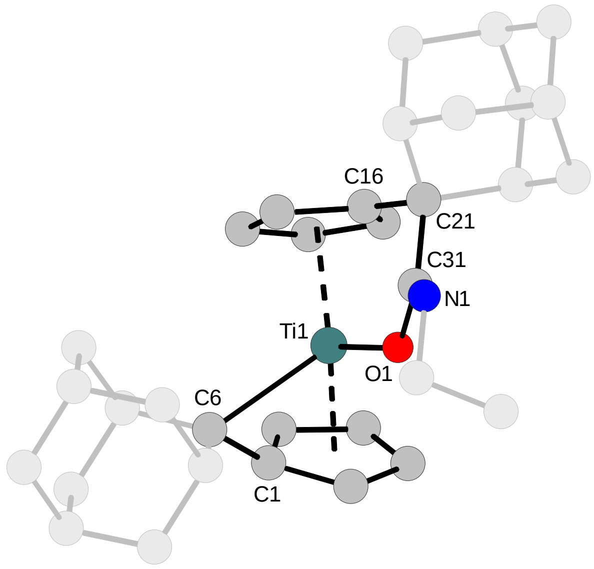

Figure S136. Ground state optimized geometry of complex **1a**, calculated in toluene at the B3LYP/Def2-TZVP. Selected bond lengths (Å): Ti1–O1 1.92, Ti1–C6 2.42, C1–C6 1.43, C16–C21 1.52, C21–C31 1.56, C31–O1 1.34, C31–N1 1.27.

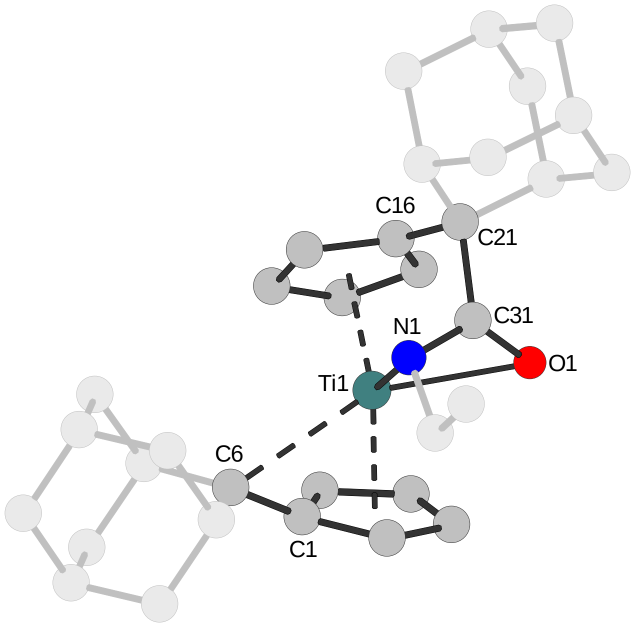

Figure S137. Transition state optimized geometry of complex **1a**, calculated in toluene at the B3LYP/Def2-TZVP level of theory. Selected bond lengths (Å): Ti1–O1 2.62, Ti1–N1 2.39, Ti1–C6 2.70, C1–C6 1.41, C16–C21 1.51, C21–C31 1.57, C31–O1 1.26, C31–N1 1.35.

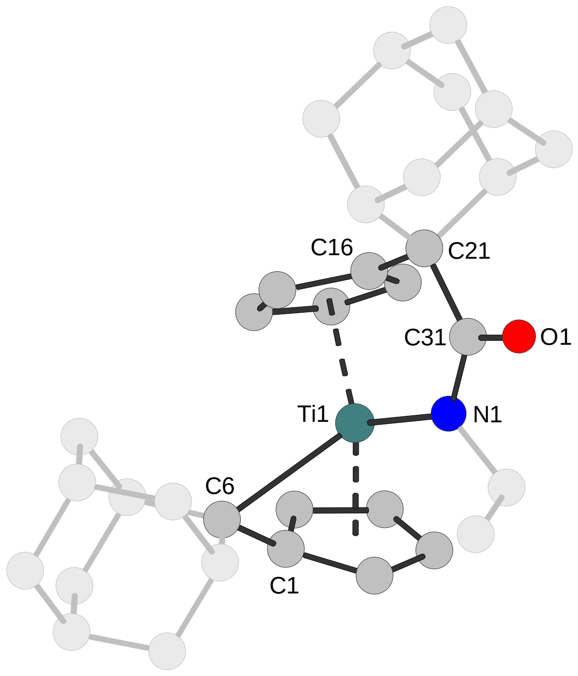

Figure S138. Ground state optimized geometry of complex **1a'**, calculated in toluene at the B3LYP/Def2-TZVP. Selected bond lengths (Å): Ti1–N1 2.09, Ti1–C6 2.53, C1–C6 1.42, C16–C21 1.51, C21–C31 1.55, C31–O1 1.23, C31–N1 1.38.

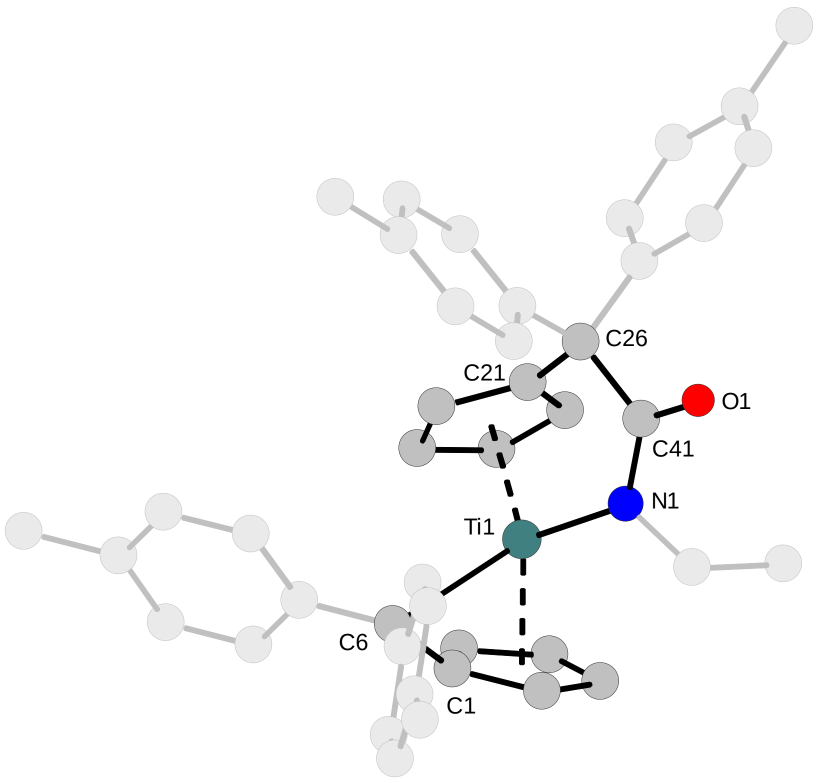

Figure S139. Ground state optimized geometry of complex **2a**, calculated in toluene at the B3LYP/Def2-TZVP. Selected bond lengths (Å): Ti1–N1 2.09, Ti1–C6 2.51, C1–C6 1.43, C21–C26 1.51, C26–C41 1.57, C41–O1 1.22, C41–N1 1.37.

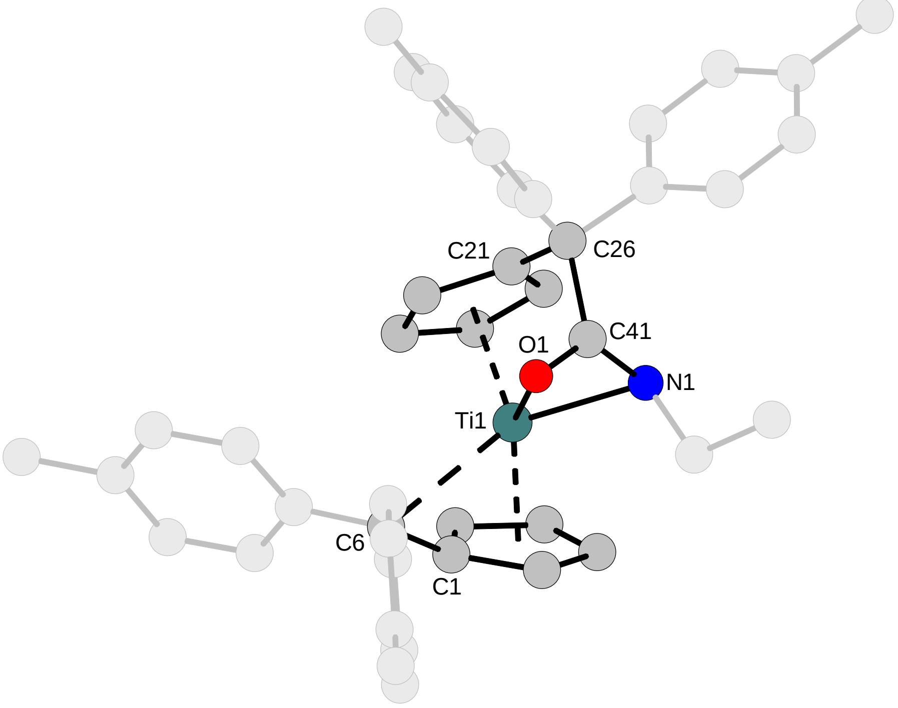

Figure S140. Transition state optimized geometry of complex **2a**, calculated in toluene at the B3LYP/Def2-TZVP level of theory. Selected bond lengths (Å): Ti1–O1 2.67, Ti1–N1 2.31, Ti1–C6 2.64, C1–C6 1.42, C21–C26 1.52, C26–C41 1.57, C41–O1 1.25, C41–N1 1.35.

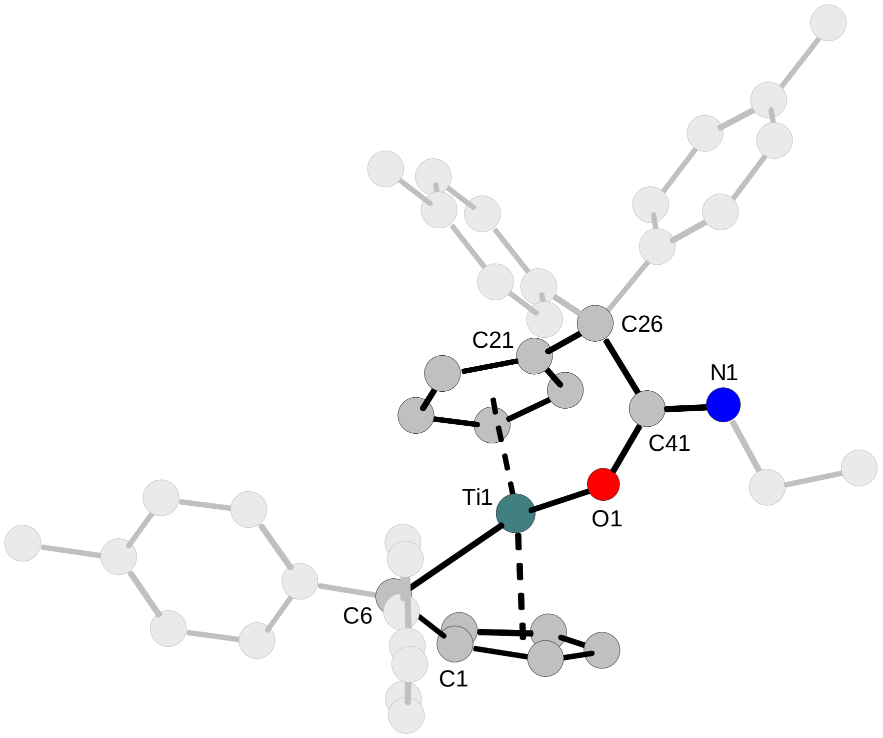

Figure S141. Ground state optimized geometry of complex **2a'**, calculated in toluene at the B3LYP/Def2-TZVP. Selected bond lengths (Å): Ti1–O1 1.93, Ti1–C6 2.46, C1–C6 1.44, C21–C26 1.52, C21–C41 1.57, C41–O1 1.34, C41–N1 1.26.

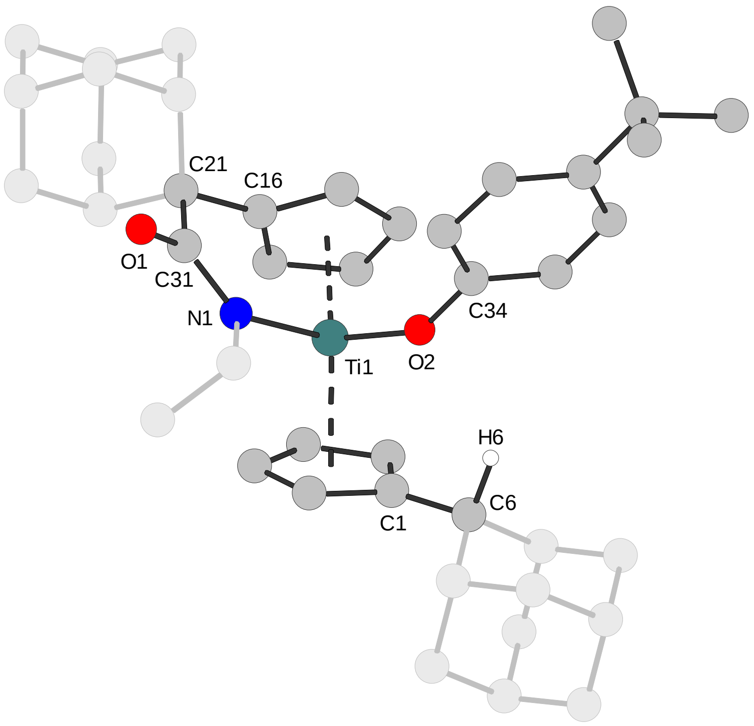

Figure S142. Ground state optimized geometry of complex **4m'**, calculated in toluene at the B3LYP/Def2-TZVP. Selected bond lengths (Å): Ti1–O2 1.88, Ti1–N1 2.06, C1–C6 1.51, C21–C26 1.51, C21–C31 1.56, C31–O1 1.23, C31–N1 1.37, O2–C34 1.34.

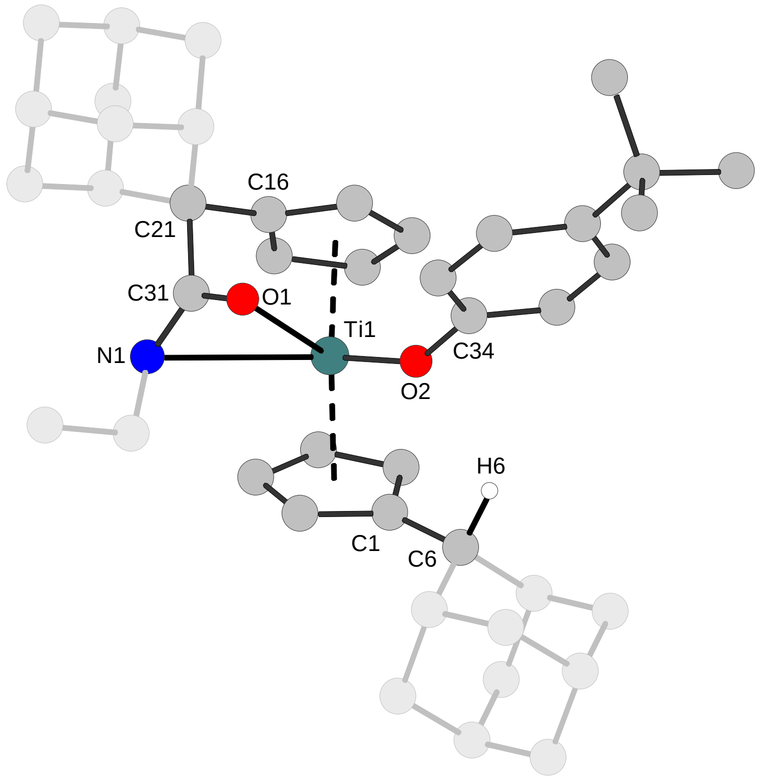

Figure S143. Transition state optimized geometry of complex **4m'**, calculated in toluene at the B3LYP/Def2-TZVP level of theory. Selected bond lengths (Å): Ti1–O1 2.76, Ti1–N1 2.90, Ti1–O2 1.87, C1–C6 1.50, C16–C21 1.51, C21–C31 1.58, C31–O1 1.26, C31–N1 1.32, O2–C34 1.34.

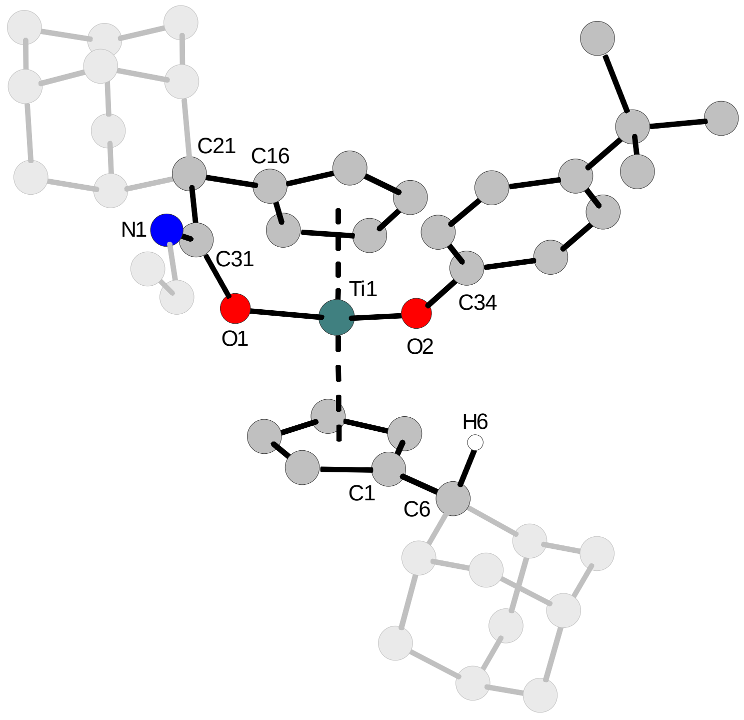

Figure S144. Ground state optimized geometry of complex **4m**, calculated in toluene at the B3LYP/Def2-TZVP. Selected bond lengths (Å): Ti1–O1 1.93, Ti1–O2 1.88, C1–C6 1.51, C16–C21 1.52, C21–C31 1.55, C31–O1 1.34, C31–N1 1.27, O2–C34 1.34.

References

[1] Manßen, M.; Lauterbach, N.; Schmidtmann, M.; Saak, W.; Doye, S.; Beckhaus, R.; Efficient Access to Titanaaziridines by C–H Activation of N-Methylanilines at Ambient Temperature. *Angew. Chem. Int. Ed.* **2015**, (54), 4383-4387. DOI: 10.1002/anie.201500796.

[2] Diekmann, M.; Bockstiegel, G.; Lützen, A.; Friedemann, M.; Saak, W.; Haase, D.; Beckhaus, R.; Chiral Bis(η^5:η^1-pentafulvene)titanium Complexes, *Organometallics* **2006**, (25), 339-348. DOI: 10.1021/om050815m.

[3] Krause, L.; Herbst-Irmer, R.; Sheldrick, G. M.; Stalke, D.; Comparison of silver and molybdenum microfocus X-ray sources for single-crystal structure determination, *J. Appl. Crystallogr.* **2015**, (48), 3-10. DOI: 10.1107/S1600576714022985.

[4] Sheldrick, G. M.; TWINABS 2012/1, Bruker, Madison, Wisconsin, USA, 2012.

[5] Sheldrick, G.; A short history of SHELX. *Acta Cryst. A* **2008**, (64), 112-122. DOI: 10.1107/S0108767307043930.

[6] Sheldrick, G.; Crystal structure refinement with SHELXL. *Acta Cryst. C* **2015**, (71), 3-8. DOI: 10.1107/S2053229614024218.

[7] O. V. Dolomanov, L. J. Bourhis, R. J. Gildea, J. A. K. Howard, H. Puschmann, OLEX2: a complete structure solution, refinement and analysis program, *J. Appl. Cryst.* **2009**, (42), 339-341. DOI: 10.1107/S0021889808042726.

[8] Weigend, F.; Ahlrichs, R.; Balanced basis sets of split valence, triple zeta valence and quadruple zeta valence quality for H to Rn: Design and assessment of accuracy, *Phys. Chem. Chem. Phys.* **2005**, *7*, 3297-3305. DOI: 10.1039/B508541A.

[9] Marenich, A.; Cramer, C.; Truhlar, D.; Universal Solvation Model Based on Solute Electron Density and on a Continuum Model of the Solvent Defined by the Bulk Dielectric Constant and Atomic Surface Tensions, *J. Phys. Chem. B* **2009***, 113*, (18), 6378–6396. DOI: 10.1021/jp810292n.
